# Quantitative Subcellular Proteome and Secretome Profiling of Influenza A Virus-Infected Human Primary Macrophages

**DOI:** 10.1371/journal.ppat.1001340

**Published:** 2011-05-12

**Authors:** Niina Lietzén, Tiina Öhman, Johanna Rintahaka, Ilkka Julkunen, Tero Aittokallio, Sampsa Matikainen, Tuula A. Nyman

**Affiliations:** 1 Institute of Biotechnology, University of Helsinki, Helsinki, Finland; 2 Finnish Institute of Occupational Health, Helsinki, Finland; 3 National Institute for Health and Welfare, Helsinki, Finland; 4 Department of Mathematics, University of Turku, Turku, Finland; Yale University School of Medicine, United States of America

## Abstract

Influenza A viruses are important pathogens that cause acute respiratory diseases and annual epidemics in humans. Macrophages recognize influenza A virus infection with their pattern recognition receptors, and are involved in the activation of proper innate immune response. Here, we have used high-throughput subcellular proteomics combined with bioinformatics to provide a global view of host cellular events that are activated in response to influenza A virus infection in human primary macrophages. We show that viral infection regulates the expression and/or subcellular localization of more than one thousand host proteins at early phases of infection. Our data reveals that there are dramatic changes in mitochondrial and nuclear proteomes in response to infection. We show that a rapid cytoplasmic leakage of lysosomal proteins, including cathepsins, followed by their secretion, contributes to inflammasome activation and apoptosis seen in the infected macrophages. Also, our results demonstrate that P2X_7_ receptor and src tyrosine kinase activity are essential for inflammasome activation during influenza A virus infection. Finally, we show that influenza A virus infection is associated with robust secretion of different danger-associated molecular patterns (DAMPs) suggesting an important role for DAMPs in host response to influenza A virus infection. In conclusion, our high-throughput quantitative proteomics study provides important new insight into host-response against influenza A virus infection in human primary macrophages.

## Author Summary

Influenza A viruses are negative-stranded RNA viruses that are capable of infecting a variety of avian and mammalian species. These viruses are responsible for the annual epidemics that cause severe illnesses in millions of people worldwide. The initial innate immune responses to influenza A viruses have to restrict virus spread before the adaptive immune responses fully develop. Macrophages are the key players of innate immune system and they have a central role in the activation of host response during viral infections. However, the host factors that are involved in the activation of innate immune response during influenza A virus infection are incompletely understood. Here, we have characterized in detail the nuclear, mitochondrial and cytoplasmic proteomes, as well as the secretome from influenza A virus-infected human primary macrophages to get a global view of host factors that are affected by the infection. Our approach allowed us to identify several novel host factors that contribute to innate immune system during influenza A virus infections. These include lysomal proteases cathepsins, P2X_7_ receptor, src family tyrosine kinases as well as several danger-associated molecular patterns.

## Introduction

Influenza A viruses are negative-stranded RNA viruses that are capable of infecting a variety of avian and mammalian species. These viruses are responsible for the annual epidemics that cause severe illnesses in millions of people worldwide. Influenza A virus and secondary bacterial infections can cause lethal pneumonia and encephalopathy especially in elder people. Host defence against influenza A virus infection is initiated by the innate immune system. The principal effector cells involved in innate immunity are macrophages, and dendritic cells (DC) that kill microbes through phagocytosis, present antigens to T cells, and produce cytokines. These innate immune responses are essential for the development of later adaptive immune responses, which provide specific cell-mediated and humoral protection, and are often necessary for a complete clearance of infection.

In viral infections innate immune responses are initiated when viruses or their genetic material is recognized by cellular pattern recognition receptors (PRRs) of the innate immune system [1]. This recognition results in an interferon (IFN)-α/β-mediated antiviral response, as well as activation of pro-inflammatory response and programmed cell death, apoptosis, of infected cells. PRRs involved in activation of antiviral response include Toll-like receptors (TLRs) and Retinoic acid-inducible gene I-like receptors (RLRs) and the coordinated activation of TLRs and RLRs results in proper activation of antiviral response during influenza A virus infection [2], [3]. Influenza A virus infection of human macrophages also results in production of pro-inflammatory cytokines IL-1β and IL-18 [4], [5]. Both of these cytokines have to be cleaved by cysteine protease caspase-1 to generate the secreted, biologically active forms of these cytokines [6]. Caspase-1 is in turn activated in a cytosolic protein complex called inflammasome [7]. The inflammasomes consist of an adapter molecule called apoptosis-associated speck-like protein containing a caspase recruitment domain (ASC) and a PRR that belongs to either the nucleotide-binding and oligomerization domain like receptors (NLRs) or the PYHIN receptor gene family. Caspase-1 activating structures include NLRP3, NOD2/NLRP1, NOD2/NLRP3, IPAF/NAIP5, and AIM2 inflammasomes [8], [9]. Recent studies in experimental mice models have shown a critical role of NLRP3 inflammasome in the host response against influenza A virus infection [10]–[12]. These studies highlight the importance of IL-1β and IL-18 as mediators of host response to influenza A virus infection. However, the exact mechanism of inflammasome activation during viral infection is not known.

Proteomics combined with bioinformatics has emerged as an important tool to extract detailed information of cellular signaling mechanisms [13]. With modern mass spectrometry (MS) -based approaches it is possible to identify and quantify thousands of proteins from cellular samples, as illustrated by Luber et al. who studied subset-specific viral recognition in dendritic cells [14]. Most of the large scale quantitative proteomics experiments, however, have focused on studying changes in protein abundances in whole cell lysates or individual organelles [14], [15]. The use of subcellular proteomics provides a deeper insight into cellular events as protein abundancies can be studied on the level of different subcellular compartments and also protein translocations between different cell parts can be detected [16], [17]. Moreover, pathway and network analyses can provide mechanistic insights by subsequently linking the proteins found to be differentially regulated to the underlying cellular functions and other key players known to be involved in these events. Here, we have used quantitative subcellular proteomics combined with bioinformatics to provide a global view of host-pathogen-interactions during influenza A virus infection of human primary macrophages. We show that viral infection regulates the expression and/or subcellular localization of more than one thousand host proteins at early phases of infection.

## Results

### Quantitative subcellular proteome and secretome analysis of influenza A virus-infected macrophages

**Figure 1:**
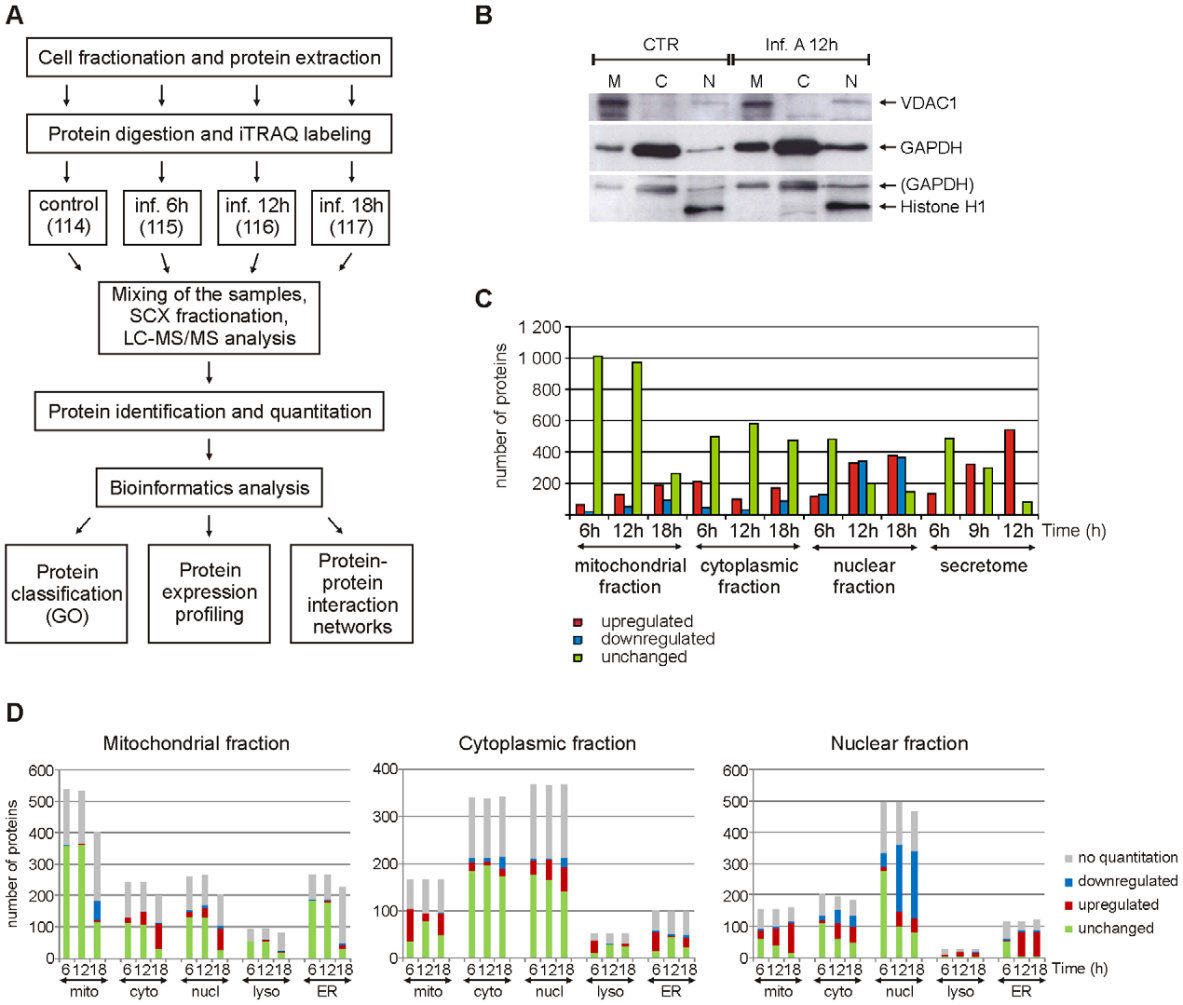
Quantitative subcellular proteome and secretome analysis of influenza A virus-infected human primary macrophages. A. Workflow of the experiment. GO = gene ontology. B. Western blot analysis of control and influenza A virus-infected cells from mitochondrial (M), cytoplasmic (C) and nuclear (N) fractions using mitochondrial, cytoplasmic and nuclear markers (voltage-dependent anion-selective channel protein 1 (VDAC1), glyceraldehyde-3-phosphate dehydrogenase (GAPDH) and histone H1, respectively). C. Numbers of reliably quantified and differentially regulated proteins (fold difference ≥1,5 or ≤0,67 for intracellular fractions and ≥3 for secretome) in each subcellular fraction and secretome at different timepoints. D. Gene ontology-based classification of all the proteins identified from intracellular fractions. Mitochondrial, cytoplasmic, nuclear, lysosomal and endoplasmic reticulum (ER) annotations for the identified proteins are shown.

For subcellular proteome and secretome analysis (Fig. 1A) human primary macrophages were first infected with influenza A virus for 6 h, 12 h and 18 h (intracellular fractions) or 6 h, 9 h and 12 h (secretome). After this the cells were fractionated into mitochondrial, cytoplasmic and nuclear fractions, and macrophage growth media were collected for secretome analysis. The enrichment of mitochondrial, cytoplasmic and nuclear proteins into different fractions was confirmed with Western blots (Fig. 1B). Protein identification and quantification from each subcellular fraction and secretome was done using 4plex iTRAQ (isobaric tag for relative and absolute quantitation) labeling combined with liquid chromatography-tandem mass spectrometry (LC-MS/MS) analysis (Fig. 1A). A total of eight iTRAQ sample sets were analysed, including two biological replicates of mitochondrial, cytoplasmic and nuclear cell fractions as well as secretomes. Additionally, each iTRAQ sample set was analysed twice with LC-MS/MS to improve the quality of protein identifications and quantifications. Based on the LC-MS/MS data we identified 1999, 1423, 1230 and 627 proteins from the mitochondrial, cytoplasmic and nuclear cell fractions and secretome, respectively, with false discovery rates (FDR) of the sample sets varying from 0.5 to 1.6%. More than one thousand proteins were identified from more than one cell fractions and altogether we identified 3477 distinct proteins. From the identified proteins, we reliably quantified 2466 proteins, and of these proteins 1321 were differentially expressed in the intracellular fractions (fold difference ≥1.5 or ≤0.67) and 544 in the secretome (fold difference ≥3) as a result of infection (Fig. 1C, Tables S1 and S2). To confirm iTRAQ quantification results, Western blot analyses were performed for a selected set of proteins (Fig. S1A–D).

**Figure 2:**
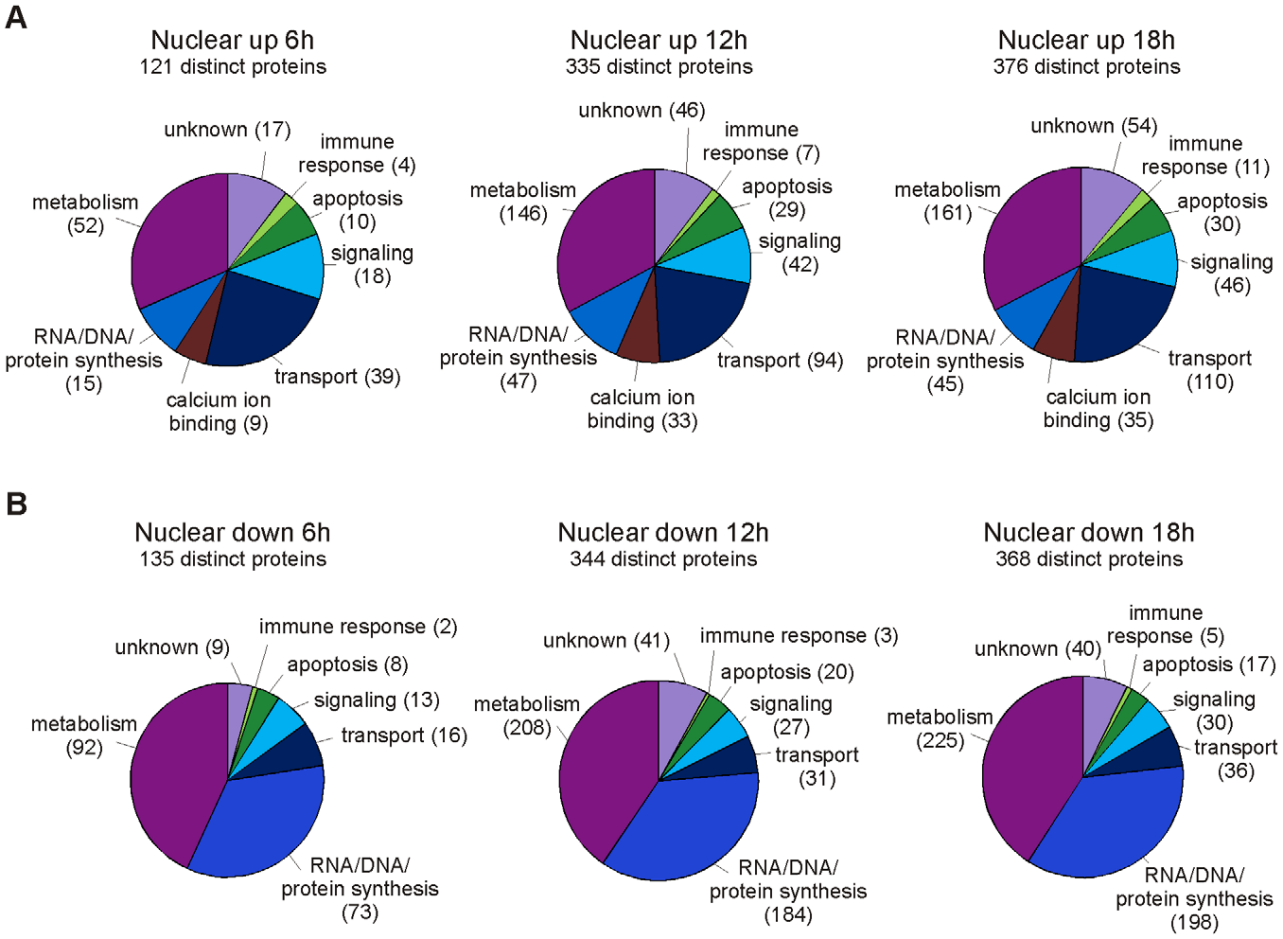
Functional classification of differentially regulated proteins in the nuclear fraction at different timenpoints. Classification of proteins that are A. upregulated and B. downregulated in the nuclear fractions of influenza A virus-infected macrophages. The numbers of proteins related with each category are shown in brackets.

To get an overview of our proteomic data the identified and quantified proteins were analysed further using different bioinformatics tools. The proteins were first classified using the gene ontology (GO) annotations of their known subcellular locations (Fig. 1D) and biological functions (Fig. 2, Fig. S2). Most of the known mitochondrial proteins identified in this study were found from the mitochondrial fraction. In addition to mitochondrial proteins, most of the identified lysosomal and endoplasmic reticulum (ER) proteins were located in the mitochondrial fraction implying that these subcellular organelles are also enriched in the mitochondrial fraction. Proteins with a known cytosolic localization were most abundant in the cytoplasmic fraction. Many cytosolic proteins, however, also have a nuclear annotation which can be seen in our data as a large number of ‘nuclear’ proteins in the cytoplasmic fraction. In the nuclear fraction, proteins with a nuclear annotation were clearly the most abundant.

### The nuclear proteome changes dramatically upon influenza A virus infection

A large number of differentially expressed proteins were identified from the nuclear cell fraction at 12 and 18 h post-infection (Fig. 1C). In the nuclear fraction 335 and 376 proteins were overexpressed, and 344 and 368 proteins underexpressed at 12 and 18 h post-infection, respectively. The overexpressed proteins contain several mitochondrial, ER, Golgi and cytosolic proteins. Functionally the overexpressed proteins form a heterogeneous group of proteins including several metabolism, gene expression, calcium ion binding, transport and signaling related proteins (Fig. 2A). Here, proteins related with gene expression are mainly nuclear or ribosomal proteins whereas proteins related with metabolism, transport, signaling and calcium ion binding originate from several different subcellular compartments. Proteins that are underexpressed in the nuclear fraction at 12 and 18 h post-infection are mainly known nuclear proteins. Almost 200 of them are involved in gene expression, especially in nucleotide metabolism and mRNA processing (Fig. 2B). Host mRNA splicing machinery has been suggested to be important for influenza virus gene expression [18], and interestingly, 40 proteins related with nuclear mRNA splicing were underexpressed in our data. Finally, our proteomics data showed that the amount of several nuclear histones increased clearly in the cytoplasmic fraction at 12 and 18 h post-infection implying that there are major changes in the nuclear architecture after influenza A virus infection.

### Several apoptosis-related proteins and interferon-inducible proteins are regulated in response to influenza A virus infection

**Figure 3:**
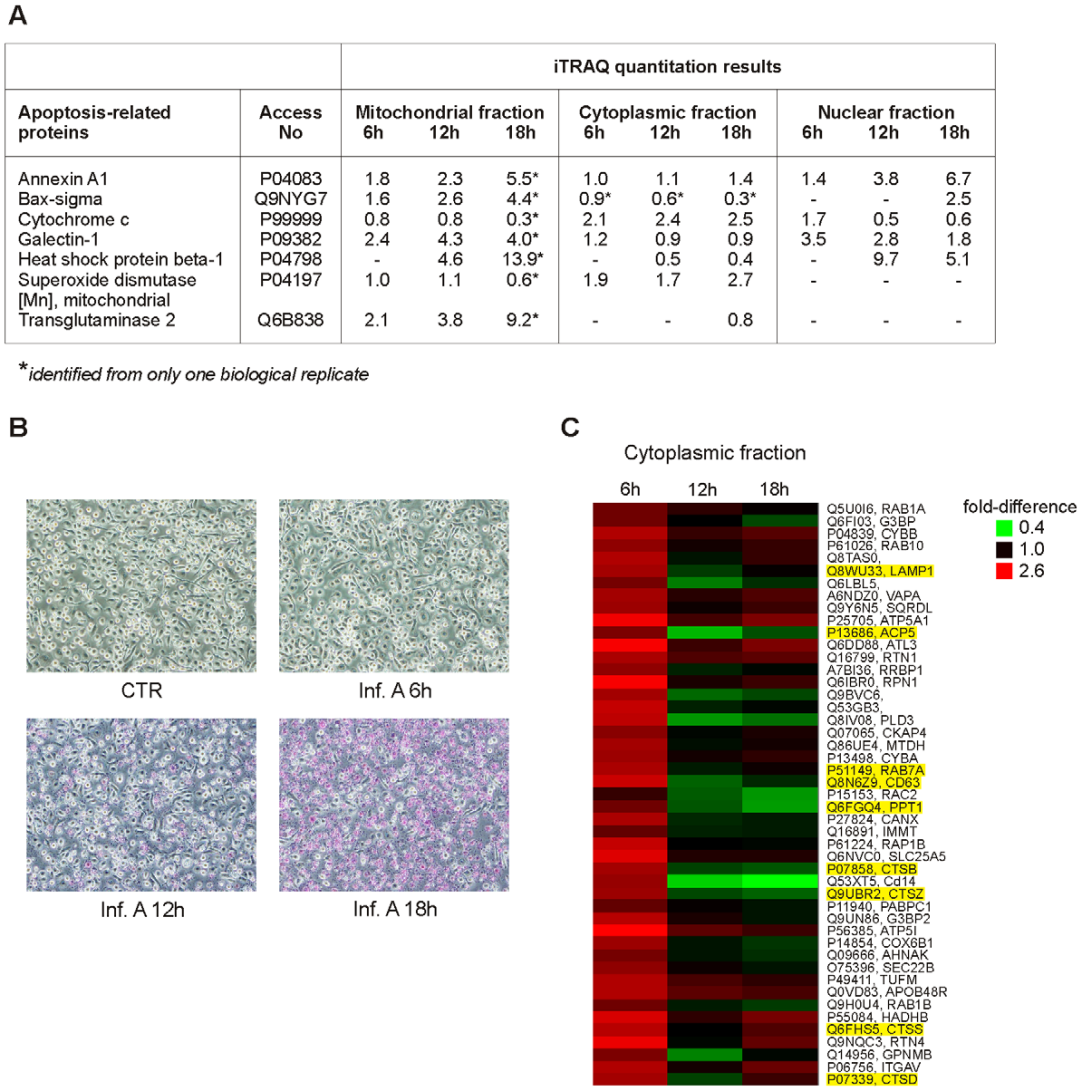
Influenza A virus infection results in apoptosis and increasing amount of lysosomal proteins in cytoplasm. A. Examples of differentially expressed apoptosis-related proteins in intracellular fractions. B. Control and influenza A virus-infected macrophages were stained with APOPercentage Apoptosis Assay. Apoptotic cells are stained as purples. C. Lysosomal proteins as well as mitochondrial proteins are overexpressed in the cytoplasmic fraction already at 6 h post-infection. Lysosomal proteins are highlighted as yellow.

The iTRAQ quantitation results and gene ontology analyses show that the number of differentially expressed apoptosis-related proteins increases in macrophages as the infection proceeds (Fig. 2, Fig. 3A, Fig. S2). Our proteomics data showed, for example, that the amount of Bax decreases in the cytoplasmic fraction and increases in the mitochondrial fraction, and the amount of cytochrome c decreases in the mitochondrial fraction and increases in the cytoplasmic fraction upon infection (Fig. 3A). These data together with Western blot analysis of cytochrome c (Fig. S1) are well in accordance with classical signs of mitochondrial apoptosis: translocation of Bax onto the mitochondria and leakage of cytochrome c into the cytoplasm. APOPercentage apoptosis assay was used to confirm the progression of apoptotic events in influenza A virus infected macrophages (Fig. 3B). No apoptotic cells were detected from control samples or influenza A virus infected samples at 6 h post-infection. At 12 h after the infection 19% of the cells were apoptotic, and at 18 h after the infection already 73% of the cells were apoptotic demonstrating the rapid progression of apoptosis at later timepoints.

It is known that influenza A virus infection triggers the production of IFN-α/β which is followed by the activation of IFN-inducible genes. Accordingly, a clear increase in the expression of several interferon-inducible proteins was seen in the cytoplasmic fraction of influenza A virus infected macrophages (Table S1). These proteins included three members of the interferon-inducible protein with tetratricopeptide repeat (IFIT) family, a group of proteins functioning as antiviral sensors that inhibit protein synthesis through translational arrest. Finally, the expression of several influenza A virus proteins such as hemagglutinin (HA), neuraminidase (NA) and nucleoprotein (NP) was clearly increased in the cytoplasmic fraction of the infected cells (Table S1).

### The level of mitochondrial and lysosomal proteins increases rapidly in the cytoplasmic fraction of influenza A virus-infected macrophages

Several proteins with mitochondrial, lysosomal and ER annotations were upregulated in the cytoplasmic fraction already at 6 h post-infection indicating rapid changes in these compartments upon infection (Fig. 1D). Based on clustering analysis, these proteins belong to a group where the amount of protein in the cytoplasmic fraction increases rapidly at 6 h post-infection and starts to decrease at later time points after infection (Fig. 3C). Surprisingly, the mitochondrial proteins in this group contain several components of the inner mitochondrial membrane including components of the electron transport chain and oxidative phosphorylation. Lysosomal proteins belonging to this cluster contain for example lysosomal proteases, cathepsins (Fig. 3C).

### Secretome characterisation of influenza A virus-infected macrophages

To complete our high-throughput proteomics analysis, we performed secretome characterization of influenza A virus-infected macrophages. Influenza A virus infection of human macrophages clearly induced protein secretion already at 6 h post-infection, and more robust protein secretion was seen at 9 h and 12 h time-points (Fig. 1C, Table S1). Our data shows that influenza A virus infection of macrophages activates both conventional as well as unconventional protein secretion [19]. Examples of the conventionally secreted proteins include e.g. C-C motif chemokine 3, C-C motif chemokine 24, complement C1q subcomponent subunit B, epididymal secretory protein E1, macrophage colony-stimulating factor 1, macrophage metalloelastase, matrix metalloproteinase-9, metalloproteinase inhibitor 2, and plasminogen activator inhibitor 1. The unconventionally secreted proteins include several danger-associated molecular pattern molecules (DAMPs) like amyloid beta A4 protein, annexins, galectins, heat shock proteins, high-mobility group box proteins (HMGBs), and S100 proteins (Table 1). Enhanced secretion of amyloid beta A4 protein, galectin-3, HMGB1, and S100-A9 protein in response to influenza A virus infection was verified by Western blotting (Fig. S1D). In addition to DAMPs, secretion of several other proteins known to be secreted through unconventional secretory pathway was enhanced. These include cystatins A and B, macrophage migration inhibitory factor, and thioredoxin. Interestingly, influenza A virus infection of macrophages also activated the secretion of Ras-related proteins Rab-1A, Rab-2b, Rab-10, and Rab11, as well as several components of vacuolar ATPases (Table 1). Furthermore, enhanced secretion of several lysosomal proteases, cathepsins, was seen in cell culture supernatants of influenza A virus-infected macrophages.

**Table 1:** iTRAQ data reveals increased secretion of several danger signal proteins, ras-related proteins, V-type proton ATPases and cathepsins in the influenza A virus infected macrophages.

Protein name	Access No	Secretome		
		6 h	9 h	12 h
**Danger signal proteins**				
Amyloid beta A4 protein	Q5IS80	2.1	1.8	2.8
Galectin-3	P17931	2.3	2.5	4.9
High mobility group protein B1	P09429	2.1	5.6	8.7
High mobility group protein B2	P26583	2.9	5.1	11.2
Protein S100-A8	P05109	1.8	2.0	3.3
Protein S100-A9	P06702	2.1	2.2	4.1
**Ras-related proteins**				
Ras-related protein Rab-10	P61026	-	5.8	12.6
Ras-related protein Rab-11A	P62491	-	3.9	5.5
Ras-related protein Rab-1A	P62820	-	-	4.5
Ras-related protein Rab-2	P61225	-	-	14.9
**V-type proton ATPases**				
V-type proton ATPase catalytic subunit A	P38606	4.3	4.4	7.8
V-type proton ATPase subunit B, brain isoform	P21281	-	-	11.9
V-type proton ATPase subunit C	P21283	5.2	5.4	7.3
V-type proton ATPase subunit D	Q9Y5K8	-	-	4.8
V-type proton ATPase subunit E 1	P36543	2.8	4.3	8.6
V-type proton ATPase subunit G 1	O75348	2.1	3.7	7.6
**Cathepsins**				
Cathepsin B	P07858	1.1	1.1	2.2
Cathepsin D	P07339	2.4	2.8	6.7
Cathepsin H	P09668	2.2	2.7	5.7
Cathepsin L2	O60911	3.3*	3.6*	9.8*
Cathepsin S	P25774	1.5	1.6	3.3
Cathepsin Z	Q9UBR2	1.5	1.5	3.0

Fold differences for the proteins are calculated as an average of both biological replicates.

*identified from only one biological replicate.

### Cathepsins are essential for influenza A virus-induced inflammasome activation

**Figure 4:**
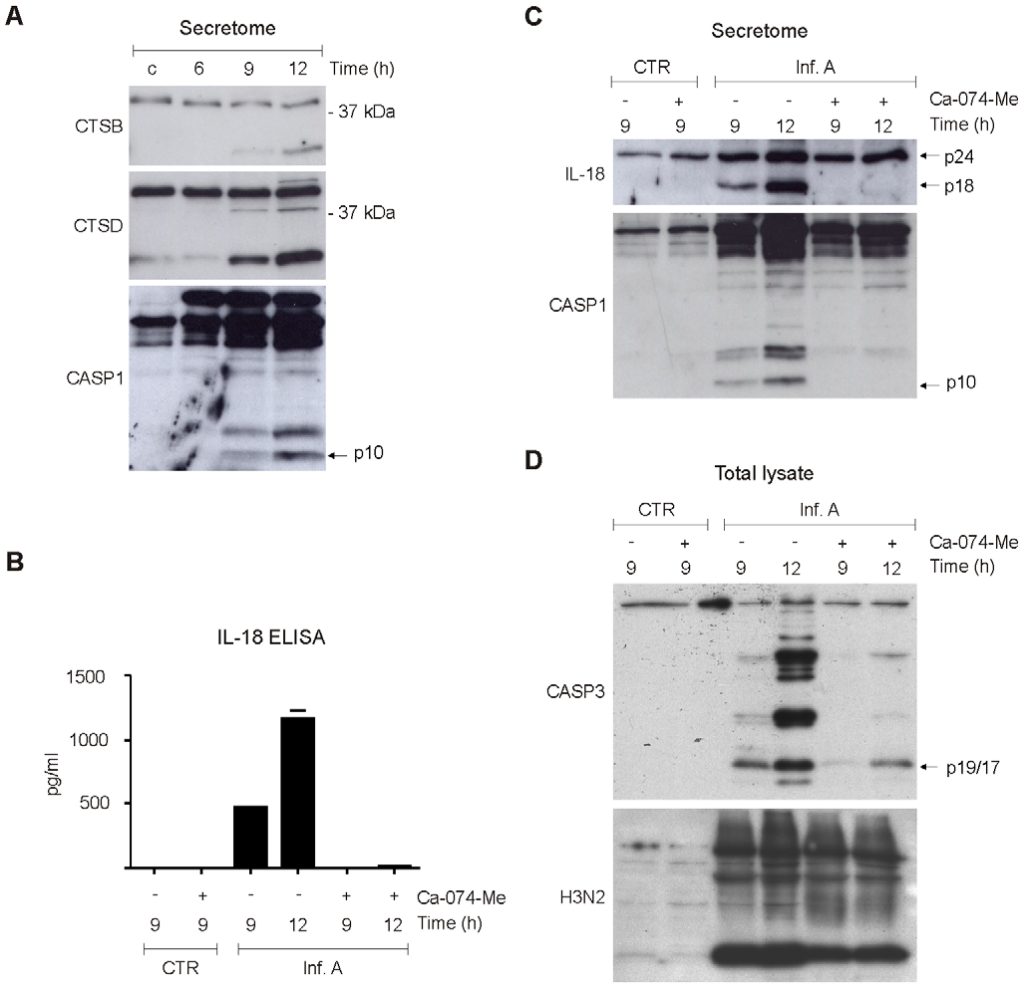
Cathepsins are essential for influenza A virus-induced inflammasome activation. A. Human macrophages were left untreated or infected with influenza A virus for 6, 9, or 12 h, after which concentrated cell culture media was prepared for Western blot analysis with anti-cathepsin B, anti-cathepsin D, anti-caspase-1 Abs. B–C. Human macrophages were infected with influenza A virus in the presence and absence of 40 µM Ca-074 Me, after which the cell culture media was collected and analysed with IL-18 ELISA, and Western blot analysis with anti-IL-18 and caspase-1 specific Abs. D. Human macrophages were infected with influenza A virus in the presence and absence of Ca-074 Me (40 µM). After this total protein lysates were prepared and analyzed by Western blotting with anti-caspase-3 p19/17 and anti-H3N2 specific Abs.

Cytoplasmic leakage of cathepsins has been associated with the activation of NLRP3 inflammasome-associated caspase-1 [20]. Our proteome analysis showed a rapid increase in the amount of cathepsins in the cytoplasm as a result of virus infection. In addition, secretome analysis showed that there is a major increase in the secretion of cathepsins and their regulators in macrophages infected with influenza A virus. To characterize the role of cathepsins in influenza A virus-induced inflammasome activation we performed Western blot analysis which showed that mature 25 kDa cathepsin B and 34 kDa cathepsin D are secreted simultaneously with biologically active p10 form of caspase-1 in response to influenza A virus infection (Fig. 4A). Next, macrophages were infected in the presence and absence of cathepsin B inhibitors, Ca-074-Me and z-FA-fmk, after which IL-18 secretion was analysed by ELISA. Both Ca-074-Me (Fig. 4B) and z-FA-fmk (Fig. S3A) almost completely abolished IL-18 secretion in response to influenza A virus (Udorn/72) infection. This result was confirmed by Western blot analysis of concentrated cell culture supernatants which showed that the appearance of biologically active form of IL-18, IL-18 p18, is abolished in macrophages that have been infected with influenza A virus in the presence of Ca-074-Me (Fig. 4C). In accordance with these results, we did not detect any caspase-1 p10 in cell culture supernatants after Ca-074-Me treatment (Fig. 4C). In addition, Ca-074-Me clearly inhibited IL-18 secretion in response to another influenza A H3N2 strain, Beijing 353/89 (Fig. S3B). Collectively, our data shows that cathepsin B activity is essential for inflammasome activation in response to influenza A virus infection.

### Influenza A virus-induced apoptosis is dependent on cathepsin B

In addition to inflammasome activation, cytoplasmic leakage of cathepsins is associated with cell death. Activation of caspase-3 is the hallmark of programmed cell death, apoptosis, and we have previously shown that caspase-3 is activated in human macrophages during influenza A virus infection [5]. To study the role of cathepsin B in influenza A virus-induced activation of apoptosis, macrophages were infected in the presence and absence of cathepsin B inhibitor, Ca-074-Me, and caspase-3 activation was analysed by Western blotting. Like caspase-1, caspase-3 is a latent zymogen, which is processed upon activation into smaller polypeptide chains: p17 and p12, which in turn form the bioactive enzyme. Influenza A virus (Udorn/72) infection clearly activated the formation of caspase-3 p17/p19 (Fig. 4D). Furthermore, Ca-074-Me inhibited the formation caspase-3 p17/p19 in response to infuenza A viruses Udorn/72 (Fig. 4D) and Beijing 353/89 (Fig. S3C) confirming the role of cathepsins in the activation of apoptosis during infection. This effect was not dependent on diminished viral replication since Ca-074-Me treatment had only little effect on influenza A virus protein expression (Fig. 4D).

### Influenza A virus-induced inflammasome activation is dependent on P2X_7_ receptor and src tyrosine kinase activity

**Figure 5:**
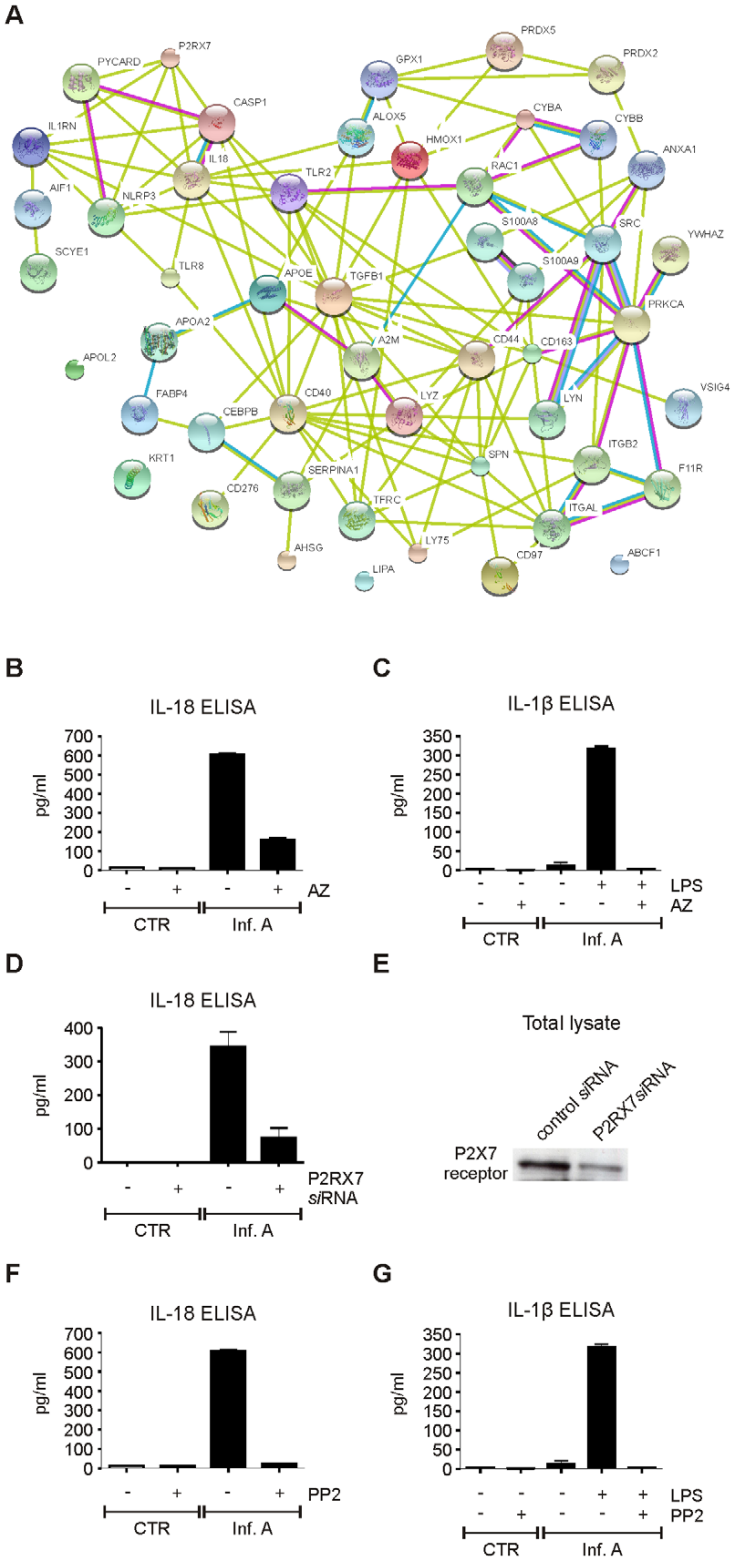
Influenza A virus-induced inflammasome activation is dependent on P2X_7_ receptor and src tyrosine kinase activity. A. Protein-protein interaction network of all inflammatory proteins identified from intracellular fractions. B. Human macrophages were infected with influenza A virus for 9 h in the presence and absence of AZ11645373 (1 µM), after which the cell culture media was collected and analyzed with IL-18 ELISA. C. Human macrophages were primed with LPS for 18 h, after which the macrophages were infected with influenza A virus for 9 h in the presence and absence of AZ11645373. After this cell culture supernatants were analyzed with IL-1β ELISA. D. Human macrophages were treated with control *si*RNA and P2X_7_ receptor specific *si*RNAs for 24 h after which the cells were left unstimulated or infected with influenza A virus for 9 h. IL-18 secretion was analyzed from cell culture supernatants with IL-18 ELISA. E. P2X_7_ receptor expression was analyzed by Western blotting with anti-P2X_7_ specific Abs. F. Macrophages were infected with influenza A virus for 9 h in the presence and absence of PP2 (5 µM), after which the cell culture media was collected and analyzed with IL-18 ELISA. G. Macrophages were primed with LPS for 18 h, after which the macrophages were left untreated or treated with PP2 and infected with influenza A virus for 9 h. The cell culture supernatants were analyzed with IL-1β ELISA.

Several proteins related with inflammatory response were identified from influenza A virus-infected macrophages. Thus, we created a network based on known protein-protein interactions between all the inflammation-related proteins identified from the intracellular fractions of the infected cells (Fig. 5A). NLPR3 was added to our protein interaction network since it has been shown to be essential for the activation of inflammatory response during influenza A virus infection. Protein interaction network showed that purinenergic P2X_7_ receptor is directly linked to NLRP3. P2X_7_ receptor has been shown to be involved in NLRP3 activation in response to extracellular ATP [21], [22], and host tissue damage during influenza A virus infection may result in extracellular leakage of ATP. Therefore we studied the role P2X_7_ receptor in virus-induced inflammasome activation. Specific inhibitor for P2X_7_ receptor, AZ11645373, clearly diminished IL-18 secretion in infected macrophages (Fig. 5B). In addition to IL-18, NLRP3 inflammasome regulates the secretion of IL-1β. We have previously shown that influenza A virus infection is not able to induce production of proIL-1β in human macrophages [23]. Therefore we stimulated macrophages with LPS for 18 h to activate proIL-1β production, after which the macrophages were infected with influenza A virus for 9 h in the presence and absence of AZ11645373. This P2X_7_ receptor inhibitor almost completely abrogated influenza A virus-induced IL-1β secretion (Fig. 5C). In addition to pharmacological inhibition, we used small interfering (si)RNA approach to study the role of P2X_7_ receptor in influenza A virus-induced inflammasome activation. Human macrophages were treated with control *si*RNA and P2X_7_ receptor specific *si*RNAs for 24 h after which the cells were left unstimulated or infected with influenza A virus for 9 h. Silencing of P2X_7_ receptor clearly reduced influenza A virus-induced IL-18 secretion (Fig. 5D) and Western blot analysis confirmed that P2X_7_ receptor protein expression was decreased in P2X_7_ receptor *si*RNA-treated macrophages (Fig. 5E). In conclusion, our results show that P2X7 receptor is essential for influenza A virus-induced inflammasome activation.

The inflammation-related proteins identified included also two NADPH oxidase subunits, gp91phox (CYBB) and p22phox (CYBA). These proteins have been linked to NLRP3 inflammasome activation through reactive oxygen species (ROS) formation in response to various stimuli [24]–[26]. Interestingly, also src tyrosine kinase is known to interact with CYBB and CYBA [27]. To study the role of tyrosine phosphorylation in influenza A virus-induced inflammasome activation we infected human macrophages in the presence and absence of PP2 which is a highly specific inhibitor of src tyrosine kinases. PP2 completely inhibited infuenza A virus Udorn/72- and Beijing 353/89-induced IL-18 secretion (Fig. 5F and Fig. S3D, respectively). Furthermore, PP2 also abrogated IL-1β secretion in response to influenza A virus infection (Fig. 5G) demonstrating that influenza A virus-induced inflammasome activation is dependent on src family tyrosine kinase activity.

## Discussion

Influenza A virus genome contains eight pieces of segmented negative-sense RNA which encode 11 proteins. Therefore the virus has to exploit host cell factors to promote its replication and suppress antiviral response. The function of viral proteins during influenza A virus infections is rather well characterized. However, the host response activated by viral infection is less well understood. The development of genome-wide screening techniques such as RNAi has resulted in the identification of hundreds of new host factors that are involved in influenza A virus replication in different cell lines [28]–[33]. The cellular responses to influenza infection as well as other respiratory viruses have also been studied using proteomics in human cell lines with both two-dimensional gel electrophoresis (2-DE) based approach [34]–[36] and newer MS-based strategies [37]–[39]. These studies have shown that similar cellular pathways are activated in response to different viruses. However, these studies have also pointed out that the cellular responses to influenza infection are in part cell-type specific and more studies using human primary cells are needed to elucidate the host response in detail.

We have previously characterized host-response to influenza infection in human primary macrophages using traditional 2-DE based proteomics and shown that actin and RIG-I/MAVS signaling components translocate onto mitochondria upon infection [40]. However, 2-DE based proteomics suffers from certain drawbacks, most importantly it shows systematic bias against certain protein groups including membrane proteins as well as very big/small proteins and proteins with extreme pIs. Also, it requires a lot of manual work and is therefore not suitable for high-throughput studies. Here, we have used unbiased high-throughput subcellular proteomics approach to analyse the host response of human primary macrophages during influenza A virus infection in a global manner. We provide evidence that the expression and/or subcellular localization of more than one thousand host proteins is affected at the early phases of infection. Up- and downregulation of proteins in all subcellular fractions reflects the dynamic interplay between these compartments and highlights the importance of subcellular proteome characterization as many of these changes cannot be seen at total proteome level.

To defend against virus infection, the host activates antiviral machinery. Interferons and IFN-inducible genes (ISGs) are the central components of this response. We detected the upregulation of many IFN-inducible proteins in the cytoplasmic fraction of influenza A virus-infected macrophages (Table S1). Interestingly, these proteins included three proteins of the IFIT family. It was very recently shown by *si*RNA approach that interferon-inducible transmembrane proteins (IFITM) restrict an early step of influenza A virus replication in A549 lung epithelial cells [29]. With our proteomics approach we did not detect any inducible expression of IFITM proteins in human macrophages in response to influenza A virus infection. It may be that IFITMs and IFITs function in antiviral response in tissue specific manner and similar to IFITMs, IFIT proteins may be a novel family of antiviral restriction factors that mediate cellular innate immunity to influenza A viruses.

Influenza A virus infection begins with the binding of viral hemagglutinin to sialyated host plasma membrane glycoprotein. Following endocytosis, viral particles are trafficked through early endosomes to late endosomes/lysosomes. In these compartments the conformation of HA is changed resulting in fusion of host-viral membranes and entry of viral ribonucleoproteins into the cytosol. We detected major changes in subcellular localization of Ras-related small GTPases and vacuolar ATPases, which are involved in the regulation of endosomal recycling pathway and have recently been shown to be essential for influenza A virus replication [28]. Furthermore, it was shown that small molecule inhibitor of vacuolar ATPase can antagonize influenza A virus replication probably by inhibiting the entry of viral ribonucleoproteins to the cytosol. Interestingly, our secretome analysis showed that several Ras-related proteins, Rab-10, Rab-11A, Rab-1A, and Rap-2b as well as components of vacuolar ATPases, V-type proton ATPases, are rapidly secreted in response to influenza A virus infection. The results suggest that these proteins are involved in protein secretion during influenza A virus infection.

We have previously shown that inflammasome-associated caspase-1 is activated in human macrophages in response to influenza A virus infection resulting in the secretion of pro-inflammatory cytokines [4], [5]. Subsequently, it has been shown that the inflammasome structure that activates caspase-1 during influenza A virus infection is NLRP3 [10]–[12], [41], [42]. Our present results show that influenza A virus infection of human macrophages is associated with endolysosomal leakage of cathepsins to the cytosol. This was followed by the activation of inflammasome-associated caspase-1 and secretion of IL-18, caspase-1, and mature forms of cathepsins. Furthermore, cathepsin specific inhibitor Ca-074-Me completely inhibited secretion of IL-18 and caspase-1 demonstrating that inflammasome activation during influenza A virus infection is completely dependent on cathepsin activity. These results indicate that lysosomal proteases, cathepsins, are an essential part of pro-inflammatory innate immune response during viral infections.

NLRP3 activators are chemically and structurally different suggesting that they are not directly recognized by the NLRP3 inflammasome. It is more likely that they activate inflammasome indirectly by inducing changes in endogenous proteins that are recognized as danger signals. In addition to cathepsin activity, potassium efflux and ROS production are the common features associated with NLRP3 inflammasome activation. It was very recently shown that thioredoxin-interacting protein links oxidative stress to inflammasome activation [43]. Our proteomic data shows that there is re-localization of mitochondrial proteins to the cytoplasm already at 6 h after influenza A virus infection. These proteins contained several components of the inner mitochondrial membrane including proteins involved in electron transport chain and oxidative phosphorylation. The results suggest that there is a substantial change and/or damage in mitochondrial structure at early phases of influenza A virus infection which may contribute to reactive oxygen species production during infection. This is likely to contribute to the inflammasome activation and apoptosis seen in influenza A virus-infected macrophages. Our current results clearly show that src tyrosine kinase activity is essential for inflammasome activation in response to influenza A virus infection: src kinase specific inhibitors PP2 (Fig. 5 F and G) and src kinase inhibitor II (data not shown) abolished influenza A virus-induced secretion of IL-1β and IL-18. It has also been recently shown that src tyrosine kinase Lyn plays a critical role in the activation of NLRP3 inflammasome in response to malarial hemozoin [44]. Src tyrosine kinases are responsible for the regulation of ROS formation through NADPH oxidase complex [27]. Activation of src tyrosine kinases results in phosphorylation of its substrates Tks4 and Tks5. These proteins act as molecular organizers that specifically activate NADPH oxidases resulting in ROS formation [45]. In conclusion, our results demonstrate a novel link between src tyrosine kinase activity, ROS formation, and inflammasome activation during viral infections.

Apoptosis is an innate immune response by which infected and other harmful cells are eliminated from the inflamed tissue. In this way intracellular danger signals are not released to extracellular space and inflammation is not further enhanced. We have previously shown that influenza A virus infection of human macrophages is associated with activation of caspase-3 [5], [23] which is the hallmark of apoptosis. Lysosomal proteases cathepsins are known to act in concert with caspases in apoptotic cell death [46]. Our current data shows that influenza A virus infection of human macrophages is associated with rapid cytoplasmic upregulation of cathepsins indicating lysosomal rupture upon infection. Furthermore, we show that cathepsins act upstream of caspase-3 and that their activity is essential for progression of apoptosis.

Most proteins are secreted through conventional protein secretion pathway. These proteins contain signal peptides that direct their transport to the plasma membrane through the ER-Golgi pathway. In our experiments, many classically secreted proteins were detected in cell culture supernatants of macrophages in response to influenza A virus infection. In addition to classical protein secretion, activated immune cells secrete proteins lacking signal peptides through unconventional secretory pathway [47]. DAMPs are nuclear or cytosolic proteins with defined intracellular functions [48]. They are usually hidden in intact cells and released during tissue damage through unconventional protein secretion pathway. Our quantitative high-throughput secretome analysis revealed secretion of several DAMPs in response to influenza A virus infection. These included 50 kDa cleavage product of amyloid precursor protein, HMGBs, galectins, and S100 proteins. Amyloid precursor protein is processed by α-, β- and γ-secretases to generate inflammatory amyloid β peptide [49]. Fibrillogenic amyloid β peptide 42 (Aβ_42_) is a known activator of NLRP3 inflammasome in migroglial cells [50] and it is involved in the pathogenesis of Alzheimer's disease [51]. We were not able to detect secretion of Aβ_42_ by ELISA in influenza A virus-infected macrophages (data not shown). However, the secretome data showed that amyloid precursor protein is expressed, processed, and secreted in response to influenza A virus infection. In addition, subcellular proteomics data demonstrates that nicastrin and preselinin, which are components of γ-secretases are expressed in human macrophages. This finding suggests that β-amyloid protein and its processing machinery have a specific function in antiviral response.

Taniguchi and coworkers have shown that HMGBs function as universal sentinels for nucleic acid mediated innate immune response [52]. HMGBs operate upstream of cytoplasmic RLRs and endosomal TLRs and they are essential for nucleic acid-induced activation of innate immune response. In our experiments both HMGB1 and HMGB2 were detected in cell culture supernatants of influenza A virus-infected macrophages. These results suggest that HMGBs have an important role in the activation of innate immune response to influenza A virus infection. Our proteomics approach showed also increased secretion of galectin-3 in response to influenza A virus infection. This finding is especially interesting since galectins are known to bind extracellularly carbohydrate structures and the envelope of influenza A virus contains two glycoproteins, hemagglutinin and neuraminidase. Hemagglutinin is required for influenza A virus entry, and it can be speculated that the extracellular galectins restrict viral entry. It is easy to envision that secreted galectins may have also other functions in antiviral response since there is ample evidence about the role of galectins in innate immunity to bacterial and fungal infections [53]. Clearly, future studies are required to characterize the importance of galectins in viral infections.

**Figure 6:**
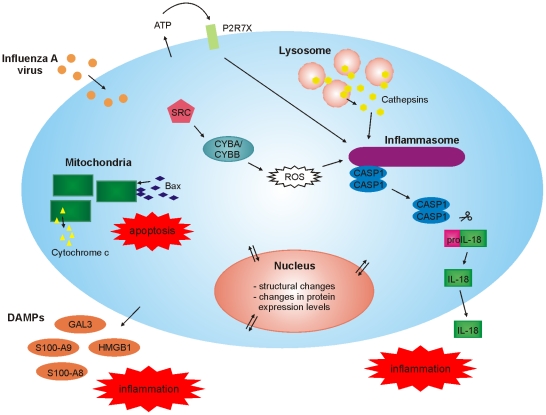
High-throughput, quantitative subcellular proteomics provides a global view of host-response in macrophages upon viral infection.

In conclusion, our high-throughput, unbiased quantitative proteomics study provides important new insight into host-response against influenza A virus infection in human primary macrophages (Fig. 6). First, our data shows that there are dramatic changes in mitochondrial and nuclear proteomes in response to infection. Secondly, our data demonstrates that there is rapid cytoplasmic leakage of lysosomal proteins, including cathepsins, upon infection which contributes to inflammasome activation and apoptosis seen in infected macrophages. Thirdly, our data demonstrates that P2X_7_ receptor and src tyrosine kinase activity are essential for inflammasome activation during influenza A virus infection. Finally, we show that influenza A virus infection is associated with robust secretion of different DAMPs suggesting an important role for DAMPs in antiviral response.

## Materials and Methods

### Cell stimulation and subcellular fractionation

Human primary macrophages were obtained from leukocyte-rich buffy coats from healthy blood donors (Finnish Red Cross Blood Transfusion Service, Helsinki, Finland). Monocytes were isolated as described previously [4] and differentiated into macrophages by maintenance in Macrophage-SFM medium (GIBCO) supplemented with 10 ng/ml GM-CSF (Biosource International) and antibiotics. After 7 days of culture, the resulting macrophages were used in experiments. Macrophages were infected with influenza A virus in complete Macrophage-SFM medium. The studied cells were lysed or fractionated into mitochondrial, cytoplasmic and nuclear fractions. Mitochondrial and cytoplasmic fractions were isolated by Qproteome Mitochondria Isolation Kit (Qiagen) and the cytoplasmic fractions were further purified with 2-D Clean-Up Kit (GE Healthcare). Nuclear fractions were isolated by Qproteome Nuclear Protein Isolation Kit (Qiagen) and the insoluble and soluble nuclear protein fractions were combined before analysis. About 1×10^7^ cells were used for all isolations. For secretome analyses, the cells grown in complete Macrophage-SFM medium were washed tree times with PBS after which the cells were stimulated in RPMI growth media supplemented with 1 mM HEPES, L-Glutamine and antibiotics (GIBCO). The growth media were collected and concentrated in Amicon Ultra centrifugal filter devices (Millipore Corporation, Billerica, MA).

### Virus stocks and infections

Human pathogenic H3N2 influenza A virus strains, Udorn/72 and Beijing/353/89, were cultured in embryonated hen eggs and stored at −70°C. The hemagglutination titer of both influenza virus strains was 256, as measured by standard methods. In infection experiments, virus dose of 2.56 hemagglutination U/ml was used. The experiments were performed with strain Udorn/72 unless otherwise stated. The protein amount of cell lysates was analysed by SDS-PAGE followed by silver-staining and Western blotting to confirm that viral infection did not decrease the total protein expression level in our experiments (Fig. S1E).

### iTRAQ labeling and mass spectrometry

Mitochondrial, cytoplasmic or nuclear fractions or secretomes of uninfected control cells and influenza A virus infected cells at given timepoints were labeled with 4plex iTRAQ. The samples were first dissolved into 43 µl of iTRAQ dissolution buffer and 2 µl of each sample was run into an SDS-PAGE gel. For the intracellular fractions, equal protein amounts of each sample were taken for the iTRAQ analyses based on the silver stained gels. For secretome analyses, the whole samples were labeled. Protein alkylation, trypsin digestion and labeling of the resulting peptides were done according to manufacturer's instructions (AB Sciex). After labeling, the samples were pooled, dried and dissolved into 20 mM KH_2_PO_4_ (pH 3). Labeled peptides were fractionated by strong cation exchange chromatography (SCX) on an Ettan HPLC system (Amersham Biosciences). Each SCX fraction containing labeled peptides was analysed twice with nano-LC-ESI-MS/MS using Ultimate 3000 nano-LC (Dionex) and QSTAR Elite hybrid quadrupole time-of-flight mass spectrometer (AB Sciex) with nano-ESI ionization (approximately 22 SCX fractions for intracellular samples and 13 fractions for secretome). MS data were acquired automatically using Analyst QS 2.0 software. Information-dependent acquisition method consisted of a 0.5 s TOF-MS survey scan of m/z 400–1400. From every survey scan two most abundant ions with charge states +2 to +4 were selected for product ion scans. Once an ion was selected for MS/MS fragmentation, it was put on an exclusion list for 60 s.

### Data-analysis

Protein identification and relative quantitation was performed using ProteinPilot 2.0.1 software (AB Sciex). Data files from both technical replicates of an iTRAQ sample set were processed together. The search database was a self-built combination of Uniprot human protein sequences and Uniprot ssRNA negative-strand viruses sequences (both form the release 55.0, 02/08). The search criteria were: cysteine alkylation with MMTS, trypsin digestion, biological modifications allowed, thorough search and detected protein threshold of 95% confidence (Unused ProtScore >1,3). Additionally, automatic bias correction was used for intracellular fractions to correct for uneven protein loading. ProteinPilot identification and quantitation results were also manually checked: for each identified protein at least two unique peptides with good quality MS/MS data were required, and MS/MS spectra with all reporter ion peak heights below 10 counts were manually removed from quantitation results. False discovery rates were calculated using a concatenated normal and reversed sequence database and a previously reported method [54].

Proteins identified from each subcellular fraction were classified based on their Gene Ontology annotations using GeneTrail [55]. Additionally, k-means clustering analysis was performed for the differentially regulated proteins in each subcellular fraction using Chipster, an open source data analysis tool (http://chipster.sourceforge.net). Clustering was done based on the relative quantitation results from the iTRAQ experiments, and a suitable number of clusters for each subcellular fraction was determined by studying the cluster profiles. Protein-protein interaction networks for a selected group of proteins was created using String [56].

### Western blotting

Isolated mitochondrial, cytoplasmic and nuclear fractions from influenza A virus infected and untreated macrophages were dissolved in Laemmli sample buffer. Equal amount of protein from each sample was loaded into an SDS-PAGE gel and transferred onto PVDF-membrane. Membranes were blocked with 5% nonfat milk, stained with different Abs overnight and detected by ECL. The primary antibodies used in this study were cathepsin B (Calbiochem), caspase-3 and HSP90 (Cell Signaling), IFIT3 (BD Transduction Laboratories), actin, annexin A1, β-Amyloid, APOE, caspase-1, cathepsin D, cathepsin Z, cytochrome c, galectin-3, GAPDH, histone H1, HMGB1, LAMP-1, P2X7 receptor, S100-A9 and VDAC1 (Santa Cruz Biotechnology Inc.). IL-18 and influenza A virus (H3N2) antibodies have been previously described [4], [40].

### ELISA

The IL-1β and IL-18 cytokine concentrations were determined by ELISA according to manufacturer's instructions. Human IL-1β and IL-18 ELISAs were purchased from Diaclone and Bender Medsystems, respectively.

### Cathepsin B inhibition experiments

The cells grown in complete Macrophage-SFM medium were washed tree times with PBS and the media was changed to RPMI growth media supplemented with 1 mM HEPES, L-Glutamine and antibiotics. The RPMI media has lower content of initial media proteins than Macrophage-SFM medium. The cathepsin B inhibitors, Ca-074 Me (Calbiochem) and z-FA-fmk (Calbiochem) were added 0.5 h before influenza A virus infection and used at final concentration of 40 µM and 50 µM, respectively.

### P2X_7_ receptor and src tyrosine kinase inhibition experiments

The P2X_7_ receptor inhibitor AZ11645373 and src tyrosine kinase inhibitor PP2 were purchased from Sigma, and they were added to macrophages 0.5 h before infection with influenza A virus. AZ11645373 and PP2 were used at final concentration of 1 µM and 5 µM, respectively.

### Small interference RNA (siRNA) experiments

After five days of cell culture in 12-well plates, macrophages were transfected with 200 nM non-targeting control *si*RNA (AllStars Negative Control siRNA, Qiagen, Hilden, Germany) and with 100 nM of each of two different P2X_7_ receptor siRNAs (Hs_P2RX7_1, Hs_P2RX7_2; Qiagen) by using HiPerFect Transfection Reagent (Qiagen) according to the manufacturer's instruction. After 4 h of *si*RNA treatment, fresh macrophage media was added to the cells. On the following day, the cells were left unstimulated or infected with influenza A virus for 9 h, after which the cell culture supernatants were collected and total proteins were isolated for ELISA and Western blot analyses, respectively.

### APOPercentage apoptosis assay

The percentage of apoptotic cells was assayed with APOPercentage Apoptosis Assay according to manufacturer's instructions (Biocolor Life Science Assays). Photographs were taken with an Olympus DP70 Digital microscope camera connected to an Olympus IX71 light microscope using DP Controller (version 2.2.1.227) and DP Manager (version 2.2.1.195) softwares. The stained (apoptotic) and unstained cells were manually counted, and the percentage of apoptotic cells was calculated.

## Supporting Information

Figure S1 Western blot analysis of selected proteins identified in the iTRAQ experiments. A–D. Comparison of iTRAQ quantitation results (in brackets) and Western blot analyses for a selected set of proteins. E. Equal amount of proteins in total cell lysates prepared from control cells and influenza A virus-infected cells was verified by Western blot analysis anti-actin, anti-GAPDH, and anti-HSP90 Abs and by silver staining.
(0.32 MB PDF)

Figure S2 Functional classification of differentially regulated proteins in mitochondrial and cytoplasmic fractions at different timenpoints. Classification of up- and downregulated proteins in A–B. mitochondrial and C–D. cytoplasmic fractions, respectively. The numbers of proteins related with each category are shown in brackets.
(0.02 MB PDF)

Figure S3 Additional validation of influenza A virus-induced inflammasome activation and apoptosis. A. Human macrophages were infected with influenza A virus for 9 h in the presence and absence of z-FA-fmk. After this cell culture supernatants were collected and IL-18 secretion was analyzed with ELISA. B–C. Human macrophages were infected with influenza A virus (Beijing 353/89) for 18 h in the presence and absence of Ca-074 Me (40 µM). After this B. IL-18 secretion was analyzed with ELISA or C. total cell lysates were prepared and Western blot analysis was performed with anti-caspase 3 Abs. D. Human macrophages were infected with influenza A virus (Beijing 353/89) for 18 h in the presence and absence of PP2 (5 µM). After this IL-18 secretion was analyzed with ELISA.
(0.06 MB PDF)

Table S1 Protein identification and relative quantitation results from the iTRAQ experiments. A. intracellular fractions and B. secretome. Results from both biological replicates are shown.
(0.58 MB PDF)

Table S2 Protein identification and quantitation results from ProteinPilot for all the analyzed samples.
(1.86 MB XLS)
