# Supplementary figures and images for: Quantitative Subcellular Proteome and Secretome Profiling of Influenza A Virus-Infected Human Primary Macrophages
Source: PLoS Pathog. 2011 May 12;7(5):e1001340. doi: 10.1371/journal.ppat.1001340 (PMC3093355; doi:10.1371/journal.ppat.1001340)

**A**

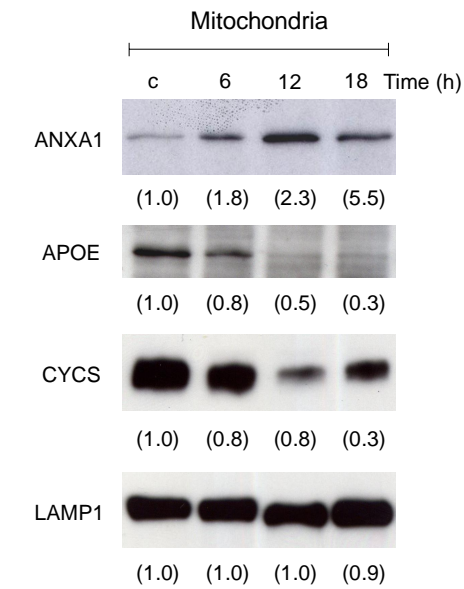

**B**

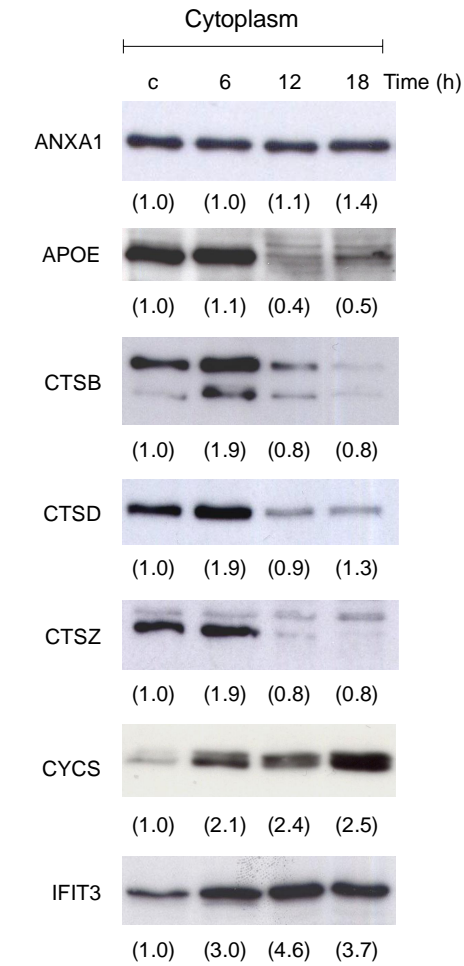

**C**

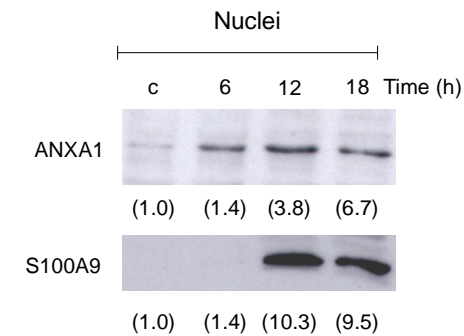

**D**

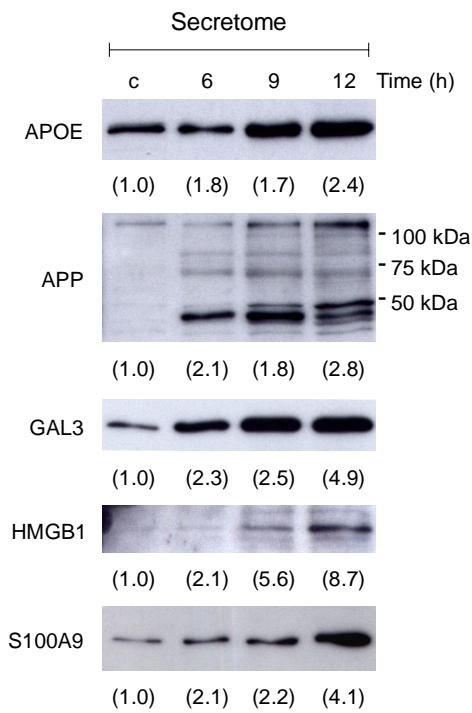

**E**

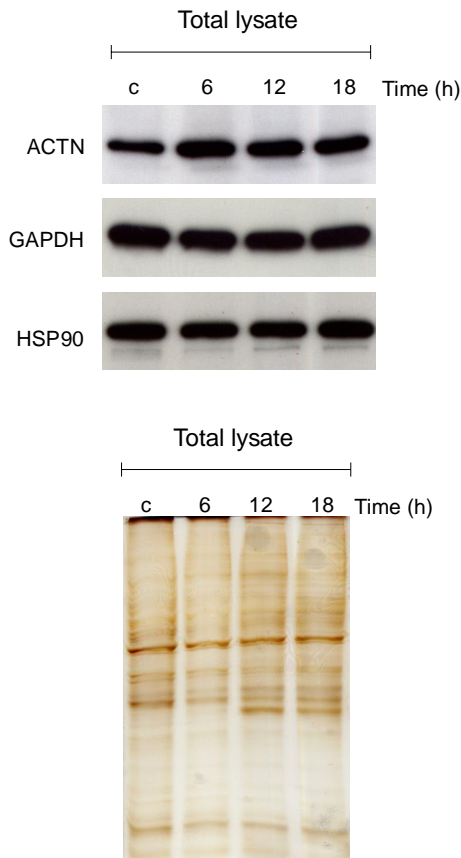

Supplement: Figure S1 — Western blot analysis of selected proteins identified in the iTRAQ experiments. A–D. Comparison of iTRAQ quantitation results (in brackets) and Western blot analyses for a selected set of proteins. E. Equal amount of proteins in total cell lysates prepared from control cells and influenza A virus-infected cells was verified by Western blot analysis anti-actin, anti-GAPDH, and anti-HSP90 Abs and by silver staining. (0.32 MB PDF) [file ppat.1001340.s001.pdf]

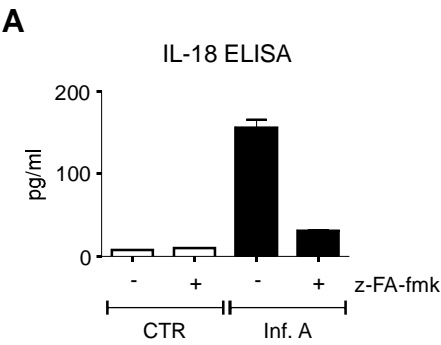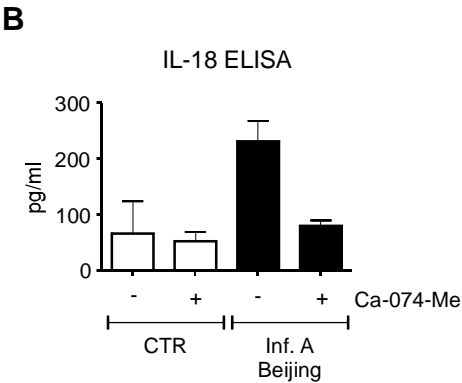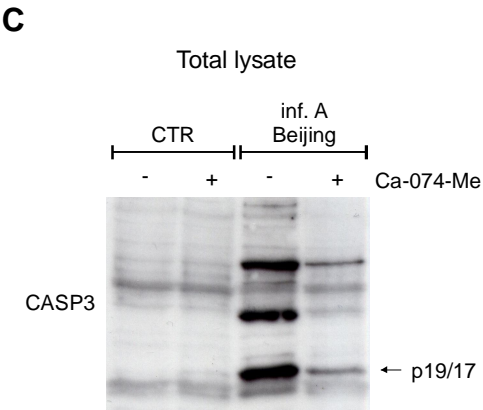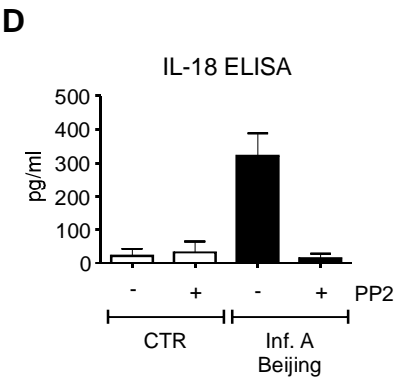

Supplement: Figure S3 — Additional validation of influenza A virus-induced inflammasome activation and apoptosis. A. Human macrophages were infected with influenza A virus for 9 h in the presence and absence of z-FA-fmk. After this cell culture supernatants were collected and IL-18 secretion was analyzed with ELISA. B–C. Human macrophages were infected with influenza A virus (Beijing 353/89) for 18 h in the presence and absence of Ca-074 Me (40 µM). After this B. IL-18 secretion was analyzed with ELISA or C. total cell lysates were prepared and Western blot analysis was performed with anti-caspase 3 Abs. D. Human macrophages were infected with influenza A virus (Beijing 353/89) for 18 h in the presence and absence of PP2 (5 µM). After this IL-18 secretion was analyzed with ELISA. (0.06 MB PDF) [file ppat.1001340.s003.pdf]
