# Supplementary material for: Quantitative Subcellular Proteome and Secretome Profiling of Influenza A Virus-Infected Human Primary Macrophages
Source: PLoS Pathog. 2011 May 12;7(5):e1001340. doi: 10.1371/journal.ppat.1001340 (PMC3093355; doi:10.1371/journal.ppat.1001340)
Supplement: Figure S2 — Functional classification of differentially regulated proteins in mitochondrial and cytoplasmic fractions at different timenpoints. Classification of up- and downregulated proteins in A–B. mitochondrial and C–D. cytoplasmic fractions, respectively. The numbers of proteins related with each category are shown in brackets. (0.02 MB PDF) [file ppat.1001340.s002.pdf]

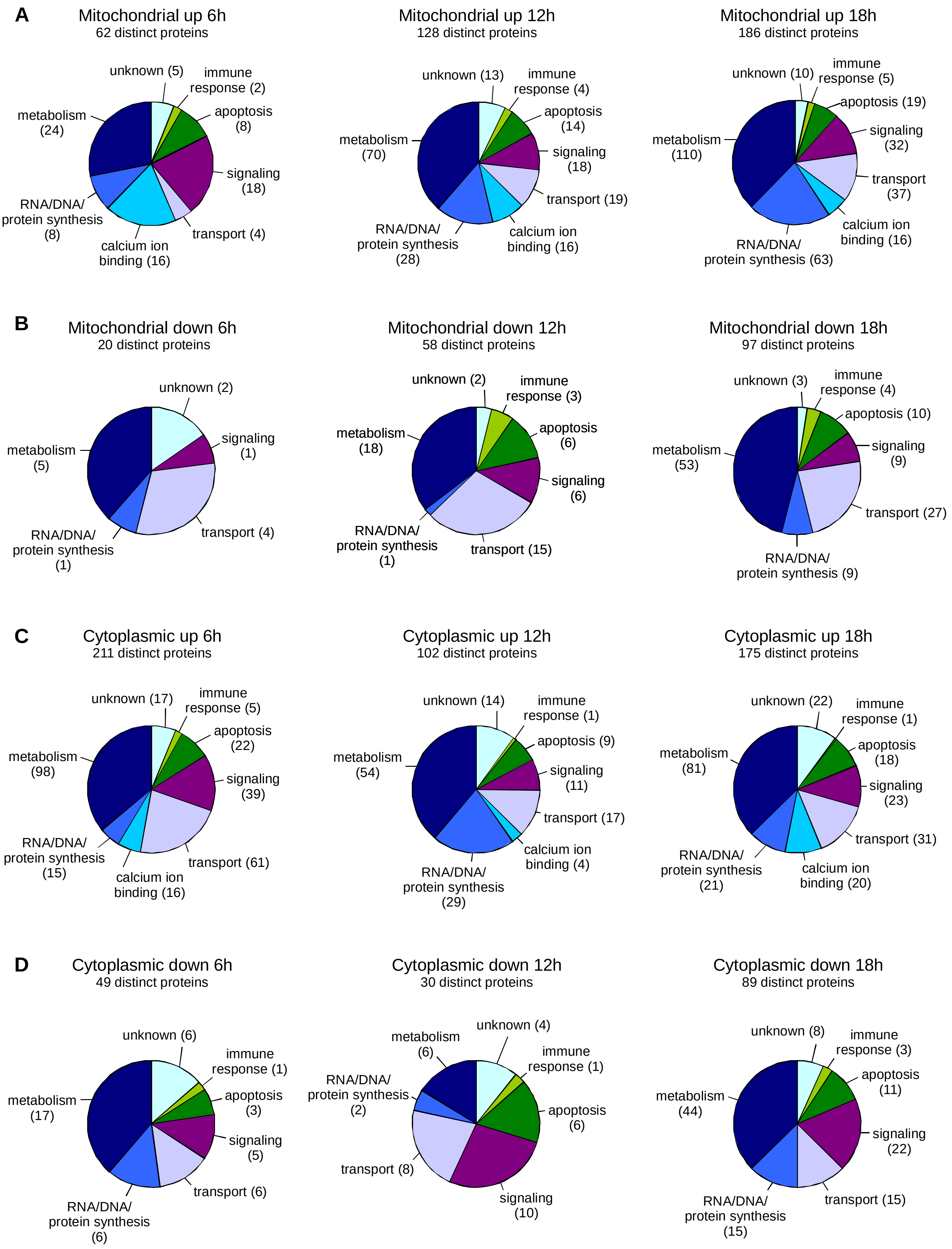

A
Mitochondrial up 6h
62 distinct proteins
unknown (5)
immune response (2)
apoptosis (8)
signaling (18)
transport (4)
calcium ion binding (16)
RNA/DNA/ protein synthesis (8)
metabolism (24)
Mitochondrial up 12h
128 distinct proteins
unknown (13)
immune response (4)
apoptosis (14)
signaling (18)
transport (19)
calcium ion binding (16)
RNA/DNA/ protein synthesis (28)
metabolism (70)
Mitochondrial up 18h
186 distinct proteins
unknown (10)
immune response (5)
apoptosis (19)
signaling (32)
transport (37)
calcium ion binding (16)
RNA/DNA/ protein synthesis (63)
metabolism (110)
B
Mitochondrial down 6h
20 distinct proteins
unknown (2)
signaling (1)
transport (4)
RNA/DNA/ protein synthesis (1)
metabolism (5)
Mitochondrial down 12h
58 distinct proteins
unknown (2)
immune response (3)
apoptosis (6)
signaling (6)
transport (15)
RNA/DNA/ protein synthesis (1)
metabolism (18)
Mitochondrial down 18h
97 distinct proteins
unknown (3)
immune response (4)
apoptosis (10)
signaling (9)
transport (27)
RNA/DNA/ protein synthesis (9)
metabolism (53)
C
Cytoplasmic up 6h
211 distinct proteins
unknown (17)
immune response (5)
apoptosis (22)
signaling (39)
transport (61)
calcium ion binding (16)
RNA/DNA/ protein synthesis (15)
metabolism (98)
Cytoplasmic up 12h
102 distinct proteins
unknown (14)
immune response (1)
apoptosis (9)
signaling (11)
transport (17)
calcium ion binding (4)
RNA/DNA/ protein synthesis (29)
metabolism (54)
Cytoplasmic up 18h
175 distinct proteins
unknown (22)
immune response (1)
apoptosis (18)
signaling (23)
transport (31)
calcium ion binding (20)
RNA/DNA/ protein synthesis (21)
metabolism (81)
D
Cytoplasmic down 6h
49 distinct proteins
unknown (6)
immune response (1)
apoptosis (3)
signaling (5)
transport (6)
RNA/DNA/ protein synthesis (6)
metabolism (17)
Cytoplasmic down 12h
30 distinct proteins
unknown (4)
immune response (1)
apoptosis (6)
signaling (10)
transport (8)
RNA/DNA/ protein synthesis (2)
metabolism (6)
Cytoplasmic down 18h
89 distinct proteins
unknown (8)
immune response (3)
apoptosis (11)
signaling (22)
transport (15)
RNA/DNA/ protein synthesis (15)
metabolism (44)
