# Supplementary material for: Quantitative Subcellular Proteome and Secretome Profiling of Influenza A Virus-Infected Human Primary Macrophages
Source: PLoS Pathog. 2011 May 12;7(5):e1001340. doi: 10.1371/journal.ppat.1001340 (PMC3093355; doi:10.1371/journal.ppat.1001340)
Supplement: Table S1 — Protein identification and relative quantitation results from the iTRAQ experiments. A. intracellular fractions and B. secretome. Results from both biological replicates are shown. (0.58 MB PDF) [file ppat.1001340.s004.pdf]

Table S1. Protein identification and relative quantitation results from the iTRAQ experiments. A. intracellular fractions and B. secretome. Results from both biological replicates are shown

A. Intracellular fractions

Protein name	SwissProt access	mitochondrial fraction			cytoplasmic fraction			nuclear fraction		
		6h (iTRAQ 115:114)	12h (iTRAQ 116:114)	18h (iTRAQ 117:114)	6h (iTRAQ 115:114)	12h (iTRAQ 116:114)	18h (iTRAQ 117:114)	6h (iTRAQ 115:114	12h (iTRAQ 116:114)	18h (iTRAQ 117:114)
[Pyruvate dehydrogenase [lipoamide]] kinase isozyme 1, mitochondrial precursor	Q15118|PDK1_HUMAN	0,67	**0,69**							
[Pyruvate dehydrogenase [lipoamide]] kinase isozyme 3, mitochondrial precursor	Q15120|PDK3_HUMAN	0,94	0,88							
14 kDa phosphohistidine phosphatase	Q9NRX4|PHP14_HUMAN				0,92	1,00	0,88			
14-3-3 protein beta/alpha	P31946|1433B_HUMAN	0,00	0,00		0,85<br>**0,72**	1,02<br>0,90	0,98<br>0,66	1,00<br>1,40	0,87<br>1,04	0,63<br>0,91
14-3-3 protein epsilon	P62258|1433E_HUMAN	0,00	0,00		0,82<br>0,95	1,06<br>0,89	0,99<br>0,92	0,00	0,00	0,00
14-3-3 protein eta	Q04917|1433F_HUMAN				0,91<br>**0,80**	0,98<br>0,91	0,80<br>0,96			
14-3-3 protein gamma	P61981|1433G_HUMAN	1,70<br>1,43	2,91<br>1,31	4,03	**0,86**<br>**0,70**	**0,83**<br>0,89	0,87<br>0,96	1,19<br>0,00	1,14<br>0,00	**0,56**<br>0,00
14-3-3 protein theta	P27348|1433T_HUMAN				1,02<br>**0,68**	1,16<br>0,83	1,14<br>**0,65**			
14-3-3 protein zeta/delta	P63104|1433Z_HUMAN	1,51<br>0,00	1,64<br>0,00	1,92	**0,85**<br>0,74	0,91<br>0,99	1,07<br>0,94	1,56	1,44	0,87
15 kDa selenoprotein precursor	O60613|SEP15_HUMAN	0,79	1,00	0,95						
1-acylglycerophosphocholine O-acyltransferase 1	Q8NF37|PCAT1_HUMAN	0,00	0,00		0,00	0,00	0,00			
1-acyl-sn-glycerol-3-phosphate acyltransferase epsilon	Q9NUQ2|PLCE_HUMAN	0,00<br>0,92	0,00<br>0,81	0,00						
1-acyl-sn-glycerol-3-phosphate acyltransferase theta	Q53EU6|PLCH_HUMAN	0,70	0,93	1,07						
1-O-acylceramide synthase precursor	Q8NCC3|LYPA3_HUMAN				1,24	0,93	0,86			
1-phosphatidylinositol-4,5-bisphosphate phosphodiesterase beta-2	Q00722|PLCB2_HUMAN							0,85	0,98	0,81
2',3'-cyclic-nucleotide 3'-phosphodiesterase	P09543|CN37_HUMAN	1,18<br>1,11	1,24<br>1,09	1,28						
2,4-dienoyl CoA reductase 2, peroxisomal	Q4VXZ8|Q4VXZ8_HUMAN	0,91	1,04							
2,4-dienoyl-CoA reductase, mitochondrial precursor	Q16698|DECR_HUMAN	0,98<br>0,96	0,93<br>1,01	**0,74**	**1,91**<br>2,14	**1,47**<br>**1,35**	**2,48**<br>**2,06**	0,87<br>**0,58**	**1,83**<br>**1,37**	**1,78**<br>**1,37**
2',5'-phosphodiesterase	Q6L8Q7|PDE25_HUMAN	1,03<br>1,11	1,29<br>1,04	1,40						
24-dehydrocholesterol reductase precursor	Q15392|DHC24_HUMAN	1,05<br>0,96	1,26<br>1,04	1,24						
24-kDa subunit of complex I (Fragment)	Q9UEH5|Q9UEH5_HUMAN	1,08	0,78	0,99						
26S protease regulatory subunit 6A	P17980|PRS6A_HUMAN				0,86<br>0,72	1,02<br>0,97	0,86<br>**0,74**			
26S protease regulatory subunit 6B	P43686|PRS6B_HUMAN				0,92	0,92	0,79			
26S protease regulatory subunit S10B	P62333|PRS10_HUMAN				**0,78**<br>0,94	**0,81**<br>0,90	**0,73**<br>0,82			
26S proteasome non-ATPase regulatory subunit 1	Q99460|PSMD1_HUMAN				0,89	1,06	0,89			
26S proteasome non-ATPase regulatory subunit 12	O00232|PSD12_HUMAN				1,18	1,13	0,80			
26S proteasome non-ATPase regulatory subunit 6	Q15008|PSMD6_HUMAN				0,70<br>0,91	0,67<br>0,84	0,58<br>0,73			
26S proteasome non-ATPase regulatory subunit 7	P51665|PSD7_HUMAN	0,97	**2,68**	**5,75**	0,86<br>0,82	1,05<br>**0,85**	0,81<br>**0,79**	0,94	**1,92**	**1,50**
26S proteasome non-ATPase regulatory subunit 9	O00233|PSMD9_HUMAN				**0,75**	0,76	0,43			
28 kDa heat- and acid-stable phosphoprotein	Q13442|HAP28_HUMAN				**0,62**	0,88	0,68	0,93<br>1,18	1,06<br>1,29	0,61<br>1,08
28S ribosomal protein S15, mitochondrial precursor	P82914|RT15_HUMAN	0,88<br>1,00	0,81<br>**0,83**	0,83						
28S ribosomal protein S16, mitochondrial precursor	Q9Y3D3|RT16_HUMAN	1,07<br>1,04	1,09<br>1,01	0,96						
28S ribosomal protein S17, mitochondrial precursor	Q9Y2R5|RT17_HUMAN	1,12	1,05							
28S ribosomal protein S18a, mitochondrial precursor	Q9NVS2|RT18A_HUMAN	0,94	1,04							
28S ribosomal protein S18b, mitochondrial precursor	Q9Y676|RT18B_HUMAN	0,84	0,88							
28S ribosomal protein S24, mitochondrial precursor	Q96EL2|RT24_HUMAN	1,22<br>**0,87**	1,09<br>0,96	0,93						
28S ribosomal protein S26, mitochondrial precursor	Q9BYN8|RT26_HUMAN	0,94<br>0,95	0,85<br>**0,85**	0,86						
28S ribosomal protein S35, mitochondrial precursor	P82673|RT35_HUMAN	1,55<br>0,99	1,36<br>1,00	1,25						
28S ribosomal protein S7, mitochondrial precursor	Q9Y2R9|RT07_HUMAN	0,88<br>0,92	0,95<br>0,90	0,80						
2-aminoethanethiol dioxygenase	Q96SZ5|AEDO_HUMAN	0,94	0,99		0,89	1,03	0,73			
2-hydroxyacyl-CoA lyase 1	Q9UJ83|HACL1_HUMAN	1,05	**1,28**							
2-oxoglutarate dehydrogenase E1 component, mitochondrial precursor	Q02218|ODO1_HUMAN	0,96<br>**0,94**	**0,88**<br>1,02	**0,80**	0,00	0,00	0,00			
3 beta-hydroxysteroid dehydrogenase type 7	Q9H2F3|3BHS7_HUMAN	0,00<br>0,92	0,00<br>1,15	0,00						
3,2-trans-enoyl-CoA isomerase, mitochondrial precursor	P42126|D3D2_HUMAN	0,93<br>**0,84**	1,00<br>**0,83**	0,61						
39S ribosomal protein 54, mitochondrial precursor	Q6P161|RM54_HUMAN	0,86	**0,86**	0,70						
39S ribosomal protein L1, mitochondrial precursor	Q9BYD6|RM01_HUMAN	0,91	0,85	0,81						
39S ribosomal protein L10, mitochondrial precursor	Q7Z7H8|RM10_HUMAN	1,07	0,97							
39S ribosomal protein L11, mitochondrial precursor	Q9Y3B7|RM11_HUMAN	0,88<br>1,07	0,95<br>**0,85**	0,83						
39S ribosomal protein L12, mitochondrial precursor	P52815|RM12_HUMAN	0,91<br>0,98	0,92<br>0,99	**0,75**	**1,97**	**1,54**	**2,50**	0,92<br>**1,66**	**2,28**<br>**3,27**	**1,81**<br>**4,26**
39S ribosomal protein L13, mitochondrial	Q9BYD1|RM13_HUMAN	1,14<br>0,87	1,49<br>0,92	7,18						
39S ribosomal protein L14, mitochondrial precursor	Q6P1L8|RM14_HUMAN	1,11	1,01							
39S ribosomal protein L15, mitochondrial precursor	Q9P015|RM15_HUMAN	0,82<br>1,04	**0,70**<br>0,94	**0,67**						
39S ribosomal protein L16, mitochondrial precursor	Q9NX20|RM16_HUMAN	0,83<br>1,05	0,79<br>0,94	0,80						
39S ribosomal protein L17, mitochondrial precursor	Q9NRX2|RM17_HUMAN	1,17<br>**0,80**	1,08<br>0,87	0,78						
39S ribosomal protein L18, mitochondrial precursor	Q9H0U6|RM18_HUMAN	0,97<br>**0,69**	0,92<br>0,87	0,85						
39S ribosomal protein L19, mitochondrial precursor	P49406|RM19_HUMAN	0,89<br>0,98	0,82<br>0,93	**0,72**						

39S ribosomal protein L2, mitochondrial precursor	Q5T653|RM02_HUMAN	1,19<br>0,93	1,05<br>0,95	0,97						
39S ribosomal protein L20, mitochondrial precursor	Q9BYC9|RM20_HUMAN	1,16<br>0,86	1,08<br>0,89	1,00						
39S ribosomal protein L21, mitochondrial precursor	Q7Z2W9|RM21_HUMAN	0,96	0,99							
39S ribosomal protein L22, mitochondrial precursor	Q9NWU5|RM22_HUMAN	0,89	0,91							
39S ribosomal protein L24, mitochondrial precursor	Q96A35|RM24_HUMAN	0,99<br>0,98	**0,84**<br>1,01	**0,64**						
39S ribosomal protein L28, mitochondrial precursor	Q13084|RM28_HUMAN	0,84	0,88	0,78						
39S ribosomal protein L30, mitochondrial precursor	Q8TCC3|RM30_HUMAN	0,98	0,91	0,92						
39S ribosomal protein L32, mitochondrial precursor	Q9BYC8|RM32_HUMAN	1,02<br>1,00	1,03<br>1,04	0,77						
39S ribosomal protein L34, mitochondrial precursor	Q9BQ48|RM34_HUMAN	1,09	0,88							
39S ribosomal protein L35, mitochondrial precursor	Q9NZE8|RM35_HUMAN	1,20	1,02							
39S ribosomal protein L37, mitochondrial precursor	Q9BZE1|RM37_HUMAN	0,89<br>0,95	**0,72**<br>1,00	**0,69**						
39S ribosomal protein L38, mitochondrial precursor	Q96DV4|RM38_HUMAN	0,87<br>0,96	0,88<br>0,91	**0,74**						
39S ribosomal protein L40, mitochondrial precursor	Q9NQ50|RM40_HUMAN	0,79<br>1,07	1,18<br>0,93	1,07						
39S ribosomal protein L41, mitochondrial precursor	Q8IXM3|RM41_HUMAN	0,76	0,97							
39S ribosomal protein L43, mitochondrial precursor	Q8N983|RM43_HUMAN	0,95	0,93	0,81						
39S ribosomal protein L44, mitochondrial precursor	Q9H9J2|RM44_HUMAN	0,77<br>0,94	0,86<br>1,09	1,04						
39S ribosomal protein L45, mitochondrial precursor	Q9BRJ2|RM45_HUMAN	1,12<br>1,05	0,98<br>0,93	1,02						
39S ribosomal protein L46, mitochondrial precursor	Q9H2W6|RM46_HUMAN	0,98<br>0,94	0,99<br>1,00	0,88						
39S ribosomal protein L47, mitochondrial precursor	Q9HD33|RM47_HUMAN	**0,76**	**0,78**	0,81						
39S ribosomal protein L48, mitochondrial precursor	Q96GC5|RM48_HUMAN	0,00<br>0,83	0,00<br>0,97	0,00						
39S ribosomal protein L51, mitochondrial precursor	Q4U2R6|RM51_HUMAN	1,03	0,92							
39S ribosomal protein L53, mitochondrial precursor	Q96EL3|RM53_HUMAN	0,91	**0,83**		2,90	1,69	1,51			
39S ribosomal protein L55, mitochondrial precursor	Q7Z7F7|RM55_HUMAN	1,09	1,02							
39S ribosomal protein L9, mitochondrial precursor	Q9BYD2|RM09_HUMAN	0,92<br>1,02	1,02<br>0,92	0,84						
3-hydroxyacyl-CoA dehydrogenase type-2	Q99714|HCD2_HUMAN	1,01<br>0,97	0,87<br>0,92	**0,73**						
3-hydroxyisobutyryl-CoA hydrolase, mitochondrial precursor	Q6NVY1|HIBCH_HUMAN	0,94<br>**1,07**	0,84<br>1,01	**0,57**	0,00	0,00	0,00			
3-ketoacyl-CoA thiolase, mitochondrial	P42765|THIM_HUMAN	**0,91**	**0,84**	**0,68**	**2,24**<br>**1,78**	**1,75**<br>1,19	**3,26**<br>**1,90**	1,10<br>**0,62**	1,16<br>1,00	**2,10**<br>**1,22**
3-ketoacyl-CoA thiolase, peroxisomal precursor	P09110|THIK_HUMAN	1,01<br>0,96	0,86<br>**0,82**	**0,82**	0,00<br>2,31	0,00<br>0,96	0,00<br>1,69			
3-mercaptopyruvate sulfurtransferase variant (Fragment)	Q59HD5|Q59HD5_HUMAN				0,88	1,09	0,79			
3-oxoacyl-[acyl-carrier-protein] synthase, mitochondrial precursor	Q9NWU1|OXSM_HUMAN	1,22	1,16							
3-oxoacyl-CoA thiolase (Fragment)	Q8NCW8|Q8NCW8_HUMAN							1,49<br>0,66	3,37<br>1,47	**5,11**<br>1,52
40S ribosomal protein S10	P46783|RS10_HUMAN				**1,16**<br>1,02	**0,89**<br>**0,88**	0,98<br>1,00	**0,87**<br>**1,42**	**1,24**<br>**1,95**	**0,73**<br>**1,49**
40S ribosomal protein S11	P62280|RS11_HUMAN	1,06	**1,75**	**5,16**	**1,23**<br>**1,29**	0,95<br>**1,18**	0,97<br>1,08	0,94<br>**0,40**	**0,63**<br>**0,44**	**0,62**<br>**0,46**
40S ribosomal protein S12	Q76M58|Q76M58_HUMAN	0,91	0,62	1,59	**1,17**	0,97	**1,15**	**0,58**<br>0,70	0,62<br>1,37	**0,42**<br>0,62
40S ribosomal protein S13	P62277|RS13_HUMAN	0,90	**1,23**	**2,09**	**1,22**<br>**1,18**	**0,88**<br>0,92	0,96<br>1,07	0,96<br>**1,39**	**0,61**<br>**0,54**	**0,39**<br>**0,53**
40S ribosomal protein S14	P62263|RS14_HUMAN	1,34<br>1,96	1,97<br>1,65	**4,85**	**1,32**<br>**1,34**	0,95<br>0,99	1,15<br>1,08	**0,74**<br>**0,79**	1,02<br>0,99	**0,59**<br>0,91
40S ribosomal protein S15	P62841|RS15_HUMAN	0,98	1,31	**3,10**	1,03	0,89	1,06			
40S ribosomal protein S15a	P62244|RS15A_HUMAN	1,27	**1,59**	**3,12**	**1,22**<br>0,91	0,91<br>0,82	1,03<br>1,08	0,76	1,08	**0,63**
40S ribosomal protein S16	P62249|RS16_HUMAN	0,89	**1,32**	**2,47**	**1,48**<br>**1,42**	1,12<br>1,01	**1,45**<br>1,29	0,99<br>**1,62**	0,92<br>0,80	**0,51**<br>0,81
40S ribosomal protein S17	P08708|RS17_HUMAN				1,45	0,90	1,10	0,80<br>1,03	0,60<br>1,04	0,63<br>1,02
40S ribosomal protein S2	P15880|RS2_HUMAN	1,20	1,44	**2,70**				0,93	**0,77**	**0,74**
40S ribosomal protein S20	P60866|RS20_HUMAN	1,70	1,63	4,63	1,23<br>1,40	0,85<br>0,80	0,98<br>1,06	0,88<br>**0,70**	0,87<br>0,67	**0,61**<br>**0,56**
40S ribosomal protein S23	P62266|RS23_HUMAN	0,73	1,19	1,95	1,10<br>1,47	0,90<br>1,13	0,98<br>1,33	1,03<br>**0,59**	**0,63**<br>**0,62**	**0,79**<br>**0,71**
40S ribosomal protein S24	Q7Z3D1|Q7Z3D1_HUMAN	0,00	0,00	0,00	0,88	0,98	0,96	**0,58**	0,96	0,56
40S ribosomal protein S25	P62851|RS25_HUMAN	0,88	**1,68**	**4,42**	1,20<br>0,98	1,04<br>0,95	1,14<br>1,05	0,94<br>**1,24**	**1,22**<br>**1,73**	1,00<br>**1,87**
40S ribosomal protein S26	P62854|RS26_HUMAN	0,71	1,45	**3,43**	1,11<br>**1,41**	0,82<br>0,91	0,94<br>0,75	0,82	**0,60**	**0,64**
40S ribosomal protein S27-like protein	Q71UM5|RS27L_HUMAN	0,00	0,00	0,00						
40S ribosomal protein S28	P62857|RS28_HUMAN				0,98	**0,74**	0,98	**0,76**<br>1,15	**0,71**<br>0,96	**0,35**<br>**0,59**
40S ribosomal protein S3	P23396|RS3_HUMAN	1,12<br>1,21	1,32<br>1,33	**2,48**	**1,44**<br>1,27	1,15<br>1,03	1,27<br>1,03	0,92<br>**0,55**	0,83<br>**0,68**	**0,67**<br>**0,56**
40S ribosomal protein S30	P62861|RS30_HUMAN				**0,00**	**0,00**	**0,00**			
40S ribosomal protein S4, X isoform	P62701|RS4X_HUMAN				**1,28**	0,93	1,00			
40S ribosomal protein S5	P46782|RS5_HUMAN	1,12	**1,42**	**3,66**	**1,23**<br>**1,19**	0,89<br>0,90	1,05<br>1,08	0,90<br>0,94	0,93<br>0,99	**0,68**<br>**0,75**
40S ribosomal protein S6	P62753|RS6_HUMAN							1,00	1,03	**1,21**
40S ribosomal protein S7	P62081|RS7_HUMAN	1,05	**1,73**	**3,77**	**1,23**<br>1,43	1,01<br>0,93	1,20<br>1,39	**0,74**<br>**1,36**	**1,80**<br>**1,38**	0,89<br>1,15
40S ribosomal protein S9	P46781|RS9_HUMAN	1,07<br>**0,79**	**1,47**<br>**1,37**	**2,88**	**1,27**<br>**1,54**	**0,84**<br>1,18	**0,81**<br>1,40	0,92<br>**0,85**	**0,70**<br>**0,52**	**0,69**<br>**0,64**
40S ribosomal protein SA	P08865|RSSA_HUMAN				**1,49**<br>1,10	1,11<br>**0,78**	**1,34**<br>0,93	1,10<br>**1,38**	**0,62**<br>0,77	**0,50**<br>0,56
45 kDa calcium-binding protein precursor	Q9BRK5|CAB45_HUMAN	**1,40**<br>**1,24**	0,79<br>0,89	**0,67**						
4F2 cell-surface antigen heavy chain	P08195|4F2_HUMAN	**1,30**<br>**1,13**	0,98<br>1,05	1,05	**1,58**<br>7,73	1,07<br>3,36	**1,45**			
4-trimethylaminobutyraldehyde dehydrogenase	P49189|AL9A1_HUMAN	1,04<br>0,99	1,01<br>0,96	0,82	1,02<br>1,04	0,91<br>**1,37**	1,00<br>1,24			
5'-3' exoribonuclease 2	Q9H0D6|XRN2_HUMAN							0,86	0,13	0,10

					0,00	0,00	0,00	0,91	0,51	**0,30**
5-formyltetrahydrofolate cyclo-ligase	P49914|MTHFS_HUMAN									
		1,07	0,93							
5-oxoprolinase	O14841|OPLA_HUMAN									
					1,20		0,65			
6,8 kDa mitochondrial proteolipid	P56378|68MP_HUMAN									
		0,83	1,05							
60 kDa heat shock protein, mitochondrial precursor	P10809|CH60_HUMAN	**0,89**	0,95	**0,83**	**2,14**	**1,46**	**3,30**	**0,72**	**2,18**	**4,72**
		1,01	1,01		1,96	1,37	2,19	0,94	**1,77**	**4,05**
60S acidic ribosomal protein P0	P05388|RLA0_HUMAN	0,87	**1,19**	**1,52**						
		1,09	**1,16**							
60S acidic ribosomal protein P0 (Fragment)	Q53HK9|Q53HK9_HUMAN				1,12	**0,79**	1,00	1,00	**1,60**	**1,53**
					0,99	**0,71**	**0,78**	1,14	**1,28**	**1,53**
60S ribosomal protein L10	P27635|RL10_HUMAN	0,89	**1,41**	**2,35**						
								**0,49**	**0,52**	**0,67**
60S ribosomal protein L10a	P62906|RL10A_HUMAN	0,94	1,08	**1,41**	1,01	**0,76**	0,88	0,94	**1,54**	**1,21**
		1,14	**1,23**		0,93	**0,82**	0,73	**1,28**	**1,24**	**1,69**
60S ribosomal protein L12	P30050|RL12_HUMAN							**0,85**	**1,51**	**1,26**
60S ribosomal protein L13	Q6NZ55|Q6NZ55_HUMAN	1,20	1,27	2,37	1,00	**0,71**	**0,51**	1,04	**1,59**	0,91
		0,00	0,00	0,00	1,65	0,95	0,75			
60S ribosomal protein L13	Q567Q8|Q567Q8_HUMAN	0,69	0,98	1,29						
		0,00	0,00	0,00	1,48	0,87	**0,53**	1,17	1,28	1,22
60S ribosomal protein L13a	P40429|RL13A_HUMAN									
		**1,52**	1,09							
60S ribosomal protein L14	P50914|RL14_HUMAN							0,00	0,00	0,00
60S ribosomal protein L17	P18621|RL17_HUMAN	**0,85**	**1,42**	**2,53**				**0,89**	**0,65**	**0,62**
		1,17	1,49		0,97	**0,86**	**0,71**			
60S ribosomal protein L18	Q07020|RL18_HUMAN	1,13	1,20	**1,64**				1,11	**1,72**	**1,55**
		1,04	**1,20**		1,23	0,99	1,04	**0,58**	**0,83**	1,14
60S ribosomal protein L18a	Q02543|RL18A_HUMAN	1,09	1,13	**1,44**						
		**1,15**	**1,10**							
60S ribosomal protein L19	P84098|RL19_HUMAN	**0,74**	0,91	**1,17**	1,02	**0,78**	**0,71**	0,98	**0,75**	**0,75**
								1,00	**0,58**	**0,76**
60S ribosomal protein L21	P46778|RL21_HUMAN	0,81	**1,29**	**2,06**	1,04	**0,79**	**0,69**	0,96	0,99	**0,75**
		2,34	1,51					**0,55**	**0,69**	0,77
60S ribosomal protein L22	P35268|RL22_HUMAN							**0,72**	**1,67**	**1,39**
		**1,70**	1,63							
60S ribosomal protein L23	P62829|RL23_HUMAN									
								**0,59**	**0,48**	**0,37**
60S ribosomal protein L23a	P62750|RL23A_HUMAN	0,94	**1,35**	**2,03**	0,93	**0,85**	0,94	**0,90**	**1,65**	**1,50**
					0,86	0,99	1,17	**1,18**	**2,12**	**2,13**
60S ribosomal protein L24	P83731|RL24_HUMAN	0,95	1,25	**2,31**	0,89	**0,75**	**0,69**	0,95	0,95	0,82
					1,26	1,06	1,02	0,98	0,96	1,10
60S ribosomal protein L26	P61254|RL26_HUMAN				1,06	**0,84**	**0,82**	**0,88**	1,11	**0,63**
		1,75	1,27					1,06	**0,81**	**0,82**
60S ribosomal protein L26-like 1	Q9UNX3|RL26L_HUMAN	0,86	1,23	**2,20**						
					1,11	1,02	0,98			
60S ribosomal protein L27	P61353|RL27_HUMAN	1,06	1,04	**1,40**	**1,27**	0,84	0,88	0,81	**1,47**	0,88
		**1,41**	1,17		1,16	1,04	1,17	1,07	1,14	1,14
60S ribosomal protein L27a	P46776|RL27A_HUMAN									
					1,17	0,74	0,65	0,80	0,49	0,91
60S ribosomal protein L28	P46779|RL28_HUMAN	0,84	**1,31**	**2,11**	0,98	0,92	**0,69**	**1,19**	1,01	0,94
		1,47	1,18		1,28	1,18	0,92	**0,38**	**0,58**	**0,61**
60S ribosomal protein L3	P39023|RL3_HUMAN									
		**1,19**	**1,39**							
60S ribosomal protein L30	P62888|RL30_HUMAN	1,03	1,10	**1,41**	1,32	1,00	**1,36**	**0,84**	**1,68**	1,06
		**1,52**	**1,55**		**1,49**	1,01	1,02	1,06	**1,46**	**1,51**
60S ribosomal protein L31	P62899|RL31_HUMAN	**0,81**	1,16	**1,51**	1,06	**0,81**	**0,79**	**0,85**	**1,49**	0,95
		**1,40**	1,47		**1,30**	0,97	1,09	**1,66**	**1,50**	**1,75**
60S ribosomal protein L32	P62910|RL32_HUMAN	0,00	0,00	0,00				0,91	1,29	0,70
60S ribosomal protein L34	P49207|RL34_HUMAN	0,87	1,21	**2,76**	0,96	0,95	1,02	0,90	**2,39**	**2,24**
					1,10	1,11	0,98	**0,35**	1,16	1,20
60S ribosomal protein L35	P42766|RL35_HUMAN	0,77	1,47	2,77	0,00	0,00	0,00	0,86	**0,75**	**0,53**
		1,15	1,21							
60S ribosomal protein L35a	P18077|RL35A_HUMAN	1,15	**1,24**	**1,53**	1,09	0,91	0,69	**1,45**	**1,87**	**1,37**
		1,04	**1,20**					**0,40**	**0,68**	**0,67**
60S ribosomal protein L36	Q9Y3U8|RL36_HUMAN	1,11	**1,33**	**1,92**	0,97	**0,65**	**0,69**	0,89	**1,41**	0,96
		**1,37**	**1,21**		0,87	0,86	0,97	1,12	1,11	**1,49**
60S ribosomal protein L36a-like	Q969Q0|RL36L_HUMAN				1,20	0,94	0,88	0,93	0,94	0,80
								**0,59**	0,61	**0,67**
60S ribosomal protein L38	P63173|RL38_HUMAN							0,96	**0,41**	0,20
					0,00	0,00	0,00	0,00	0,00	0,00
60S ribosomal protein L4	P36578|RL4_HUMAN									
		**1,52**	1,04							
60S ribosomal protein L6	Q9HBB3|Q9HBB3_HUMAN	0,96	1,14	**1,27**	1,00	**0,81**	**0,78**	1,00	**1,53**	**1,26**
		**1,39**	**1,16**		**1,29**	1,04	1,07	**0,52**	1,03	1,08
60S ribosomal protein L7	P18124|RL7_HUMAN	**0,90**	0,98	**1,26**						
					**1,17**	0,94	1,02			
60S ribosomal protein L8	P62917|RL8_HUMAN	0,95	1,13	**1,40**	**1,15**	**0,85**	0,94	1,15	**1,48**	**1,26**
		**1,35**	**1,25**		**1,29**	0,83	1,00	**0,74**	**1,18**	1,09
60S ribosome subunit biogenesis protein NIP7 homolog	Q9Y221|NIP7_HUMAN							1,20	0,94	0,85
6-phosphofructokinase, liver type	P17858|K6PL_HUMAN	1,15	**3,18**	**5,67**	**0,64**	**0,75**	**0,62**			
					0,92	**0,81**	**0,66**			
6-phosphogluconate dehydrogenase, decarboxylating	P52209|6PGD_HUMAN	1,18	**1,80**	**3,03**	**0,83**	0,97	**0,92**	1,64	2,30	**5,94**
		**1,59**	**1,67**		**0,83**	1,04	0,91			
6-phosphogluconolactonase	O95336|6PGL_HUMAN									
					0,80	0,99	0,53			
70 kDa SHP-1L protein	Q9UK67|Q9UK67_HUMAN	1,34	**2,85**	**5,75**	**0,85**	0,92	**0,67**			
					0,97	**0,84**	0,66	**0,49**	0,96	**0,61**
78 kDa glucose-regulated protein precursor	P11021|GRP78_HUMAN	**0,92**	1,00	1,00	**1,78**	**1,08**	**2,03**	0,90	**3,22**	**5,22**
		**0,95**	**1,18**		1,94	1,28	1,61	**0,88**	**2,70**	**4,83**
7-dehydrocholesterol reductase	Q9UBM7|DHCR7_HUMAN	1,01	1,16	1,07						
		**0,85**	**1,12**							
ABC-transporter	Q96PT8|Q96PT8_HUMAN	0,96	1,11	**1,23**						
		1,12	**1,16**							
Abhydrolase domain-containing protein 10, mitochondrial precursor	Q9NUJ1|ABHDA_HUMAN	**0,78**	**0,86**	**0,68**						
		0,98	0,97							
Abhydrolase domain-containing protein 11	Q8NFV4|ABHDB_HUMAN	1,04	0,89	0,69						
		0,87	**0,85**							
Abhydrolase domain-containing protein 12	Q8N2K0|ABD12_HUMAN	0,99	1,16	1,54				1,23	**1,83**	**2,90**
		0,82	0,98					0,51	1,38	2,47
Abhydrolase domain-containing protein 2	P08910|ABHD2_HUMAN	0,78	**0,41**	0,55						
Acetolactate synthase-like protein	A1L0T0|ILVBL_HUMAN	1,05	**1,27**	**1,45**						
		0,92	**1,16**							
Acetyl-CoA acetyltransferase, cytosolic	Q9BWD1|THIC_HUMAN				**0,86**	**0,79**	**0,80**			
					0,90	0,97	**0,82**			
Acetyl-CoA acetyltransferase, mitochondrial precursor	P24752|THIL_HUMAN	**0,83**	**0,76**	**0,66**						
		**1,12**	**0,89**		1,56	1,32	1,74			
	Q9NUB1|ACS2L_HUMAN	0,00	0,00	0,00						
Acetyl-coenzyme A synthetase 2-like, mitochondrial precursor										
Acid ceramidase precursor	Q13510|ASAH1_HUMAN	1,02	1,00	0,86						
		1,03	1,09		**1,53**	0,99	1,12			
Acid phosphatase 1 isoform c variant (Fragment)	Q59EH3|Q59EH3_HUMAN									
					0,50	0,94	0,30			

Acidic leucine-rich nuclear phosphoprotein 32 family member B	Q92688|AN32B_HUMAN				1,11<br>0,79	0,96<br>0,91	0,75<br>0,73			
Acidic ribosomal phosphoprotein P1	Q7Z612|Q7Z612_HUMAN	1,02	1,02							
ACLY variant protein (Fragment)	Q4LE36|Q4LE36_HUMAN	1,04	2,05	6,42	**0,81**<br>0,97	1,05<br>**1,17**	**0,87**<br>1,06			
Aconitase 1, soluble	Q5VZA7|Q5VZA7_HUMAN				**0,86**	0,92	**0,75**			
Aconitate hydratase, mitochondrial precursor	Q99798|ACON_HUMAN	0,97<br>**0,93**	0,94<br>**0,91**	**0,66**	**1,74**<br>2,19	**1,55**<br>1,75	**2,38**<br>2,25			
ACTA2 protein (Fragment)	Q13707|Q13707_HUMAN	0,00	0,00		0,95	0,83	0,85	0,62	**0,81**	0,66
Actin related protein 2/3 complex	Q2LE71|Q2LE71_HUMAN	**2,32**	**2,31**	**4,79**	0,94<br>0,93	**0,89**<br>0,93	1,00<br>0,83	1,04<br>**1,40**	**1,76**<br>**1,35**	**1,39**<br>**1,19**
Actin, beta (Fragment)	Q8WVW5|Q8WVW5_HUMAN	**1,44**	**1,82**	**4,53**				**0,91**	**0,62**	0,90
Actin, beta (Fragment)	Q96HG5|Q96HG5_HUMAN	**1,36**	**1,48**							
Actinin alpha 1 isoform b	Q1HE25|Q1HE25_HUMAN	**1,68**<br>**2,27**	1,28<br>1,29	**2,08**	**0,84**<br>0,93	**1,23**<br>**1,22**	**1,53**<br>**1,24**	**0,41**	**0,31**	0,25
Actin-like protein (Fragment)	Q562Y8|Q562Y8_HUMAN				0,00	0,00	0,00			
Actin-like protein (Fragment)	Q562Z4|Q562Z4_HUMAN				0,00	0,00	0,00			
Actin-like protein (Fragment)	Q562Z6|Q562Z6_HUMAN				0,00	0,00	0,00			
Actin-related protein 2	P61160|ARP2_HUMAN	**2,08**<br>**1,57**	**1,84**<br>**1,47**	**3,46**	0,93<br>0,99	0,96<br>1,01	**1,17**<br>0,98	1,03<br>0,88	1,18<br>1,18	**1,45**<br>1,16
Actin-related protein 2/3 complex subunit 1B	O15143|ARC1B_HUMAN	**1,84**<br>**1,37**	**1,88**<br>1,35	**4,12**	1,02<br>1,12	1,13<br>1,26	1,10<br>1,07	1,12<br>**0,35**	1,08<br>1,08	1,69<br>0,98
Actin-related protein 2/3 complex subunit 2	O15144|ARPC2_HUMAN	1,37<br>1,23	1,24<br>1,21	1,92	1,06<br>1,14	**1,15**<br>1,12	**1,25**<br>**1,67**	**0,86**<br>1,16	**1,52**<br>**1,27**	1,21<br>1,19
Actin-related protein 2/3 complex subunit 4	P59998|ARPC4_HUMAN	0,84<br>**1,39**	0,81<br>1,28	1,29	**1,23**<br>**0,79**	**1,28**<br>0,97	**1,65**<br>**1,49**	0,98<br>1,43	**1,70**<br>1,13	1,25<br>0,89
Actin-related protein 2/3 complex subunit 5	O15511|ARPC5_HUMAN	**1,82**<br>**1,51**	**1,93**<br>1,21	**3,48**	**0,84**<br>**0,77**	**0,90**<br>0,92	1,04<br>0,93	0,99<br>**1,56**	**1,87**<br>**1,63**	**1,47**<br>**1,42**
Actin-related protein 2/3 complex subunit 5-like protein	Q9BPX5|ARP5L_HUMAN							0,86	**2,01**	**1,53**
Activated RNA polymerase II transcription cofactor 4 variant (Fragment)	Q59G24|Q59G24_HUMAN	1,35	1,58		0,72<br>**0,69**	0,73<br>0,88	0,67<br>1,07	**1,17**	**2,54**	**2,73**
Activator of 90 kDa heat shock protein ATPase homolog 1	O95433|AHSA1_HUMAN				1,12<br>0,00	1,15<br>0,00	1,05<br>0,00			
Activin receptor interacting protein 5	Q49SH3|Q49SH3_HUMAN	0,99<br>1,02	0,90<br>1,06	0,89				0,91<br>2,13	1,46<br>2,13	2,09<br>4,21
Activity-dependent neuroprotector homeobox protein	Q9H2P0|ADNP_HUMAN							0,00	0,00	0,00
Acyl carrier protein, mitochondrial precursor	O14561|ACPM_HUMAN	1,05<br>1,09	1,14<br>1,12	**0,74**	1,13	**1,82**	1,75	1,03	1,26	**1,68**
Acylamino-acid-releasing enzyme	P13798|ACPH_HUMAN				1,04<br>0,74	1,19<br>1,46	1,12<br>1,01			
Acyl-CoA dehydrogenase family member 9, mitochondrial precursor	Q9H845|ACAD9_HUMAN	**0,84**<br>0,98	**0,82**<br>1,02	**0,77**						
Acyl-CoA synthetase family member 2, mitochondrial precursor	Q96CM8|ACSF2_HUMAN	1,21<br>0,92	1,25<br>**0,86**	1,05						
Acyl-CoA synthetase family member 3, mitochondrial precursor	Q4G176|ACSF3_HUMAN	1,12<br>1,00	0,86<br>0,94	0,79						
Acyl-CoA synthetase long-chain family member 4	Q5JWV8|Q5JWV8_HUMAN	1,06	0,98							
Acyl-CoA synthetase short-chain family member 1	Q7Z5G3|Q7Z5G3_HUMAN	1,01	**0,89**							
Acyl-CoA:lysophosphatidylglycerol acyltransferase 1	Q92604|LGAT1_HUMAN	0,90	1,05							
Acyl-CoA-binding domain-containing protein 5	Q5T8D3|ACBD5_HUMAN	0,00	**0,00**							
Acyl-CoA-binding protein	P07108|ACBP_HUMAN	0,99	1,12		**0,77**<br>0,54	0,95<br>**0,72**	**0,83**<br>**0,68**			
Acyl-Coenzyme A dehydrogenase, C-4 to C-12 straight chain	Q5T4U5|Q5T4U5_HUMAN	**0,89**<br>**0,91**	**0,85**<br>**0,94**	**0,80**	0,96<br>1,52	1,26<br>1,07	1,53<br>1,38			
Acyl-Coenzyme A dehydrogenase, short/branched chain	Q5SQN6|Q5SQN6_HUMAN	0,96<br>1,00	0,83<br>0,90	**0,66**						
Acyl-Coenzyme A dehydrogenase, very long chain variant (Fragment)	Q53HR2|Q53HR2_HUMAN				2,14<br>**2,56**	1,48<br>1,25	1,80<br>1,47	0,86<br>0,76	**2,88**<br>**2,63**	**5,11**<br>**2,81**
Acyl-coenzyme A oxidase 1, peroxisomal	Q15067|ACOX1_HUMAN	0,92	0,97							
Acyl-coenzyme A oxidase 3, peroxisomal	O15254|ACOX3_HUMAN	0,91<br>1,08	0,88<br>1,00	**0,76**						
Acyl-coenzyme A thioesterase 9	Q9Y305|ACOT9_HUMAN	1,00<br>1,03	1,24<br>1,04	0,82						
Acylglycerol kinase, mitochondrial precursor	Q53H12|AGK_HUMAN	0,85<br>0,94	0,88<br>0,99	**0,74**						
ADAM 10 precursor	O14672|ADA10_HUMAN	1,00<br>0,96	0,65<br>0,96	0,74						
Adaptin ear-binding coat-associated protein 2	Q9NVZ3|NECP2_HUMAN				0,89	0,89	0,82			
Adaptor-related protein complex 2, alpha 2 subunit variant (Fragment)	Q53ET1|Q53ET1_HUMAN	1,06	**0,82**							
Adenine phosphoribosyltransferase	P07741|APT_HUMAN	0,99	**4,66**	**11,01**	0,93<br>**0,74**	0,99<br>0,89	0,91<br>**0,68**			
Adenosine 3'-phospho 5'-phosphosulfate transporter 1	Q8TB61|S35B2_HUMAN	**0,52**<br>1,03	0,64<br>1,00	0,84						
Adenosine deaminase, RNA-specific isoform ADAR-a variant (Fragment)	Q59EC0|Q59EC0_HUMAN							0,82<br>**0,32**	**0,30**<br>**0,32**	**0,27**<br>**0,21**
Adenosine kinase	Q86U79|Q86U79_HUMAN	0,97	**0,84**		0,79<br>0,93	0,97<br>1,16	0,87<br>0,78			
Adenosine monophosphate deaminase 2	Q5T694|Q5T694_HUMAN				0,96<br>**0,79**	0,98<br>1,02	1,25<br>1,26			
Adenosylhomocysteinase	P23526|SAHH_HUMAN				1,11	**1,25**	1,18			
Adenylate cyclase type 7	P51828|ADCY7_HUMAN	1,04	0,93							
Adenylate kinase 2	Q5EB54|Q5EB54_HUMAN	0,80	**0,63**	**0,25**	**1,61**<br>**1,67**	**2,00**<br>**2,33**	**1,83**<br>**1,78**			
Adenylate kinase isoenzyme 4, mitochondrial	P27144|KAD4_HUMAN	0,00<br>1,06	0,00<br>0,92	0,00						
Adenylosuccinate lyase	P30566|PUR8_HUMAN				0,92	0,80	1,20			
Adenylosuccinate synthetase isozyme 2	P30520|PURA2_HUMAN				0,83	0,91	0,92			
Adenylyl cyclase-associated protein	Q5T0S2|Q5T0S2_HUMAN	1,26	1,00	2,15	0,94	1,10	**1,33**			
Adenylyl cyclase-associated protein 1	Q01518|CAP1_HUMAN	1,55	1,34		**0,89**	1,06	**1,21**			
Adipocyte plasma membrane-associated protein	Q9HDC9|APMAP_HUMAN	0,88<br>**0,94**	1,05<br>**1,16**	1,10						
ADP,ATP carrier protein, liver isoform T2 variant (Fragment)	Q59EI9|Q59EI9_HUMAN	0,88<br>0,97	0,97<br>0,86	**0,74**						
ADP/ATP translocase 2	P05141|ADT2_HUMAN	0,92<br>1,08	**0,89**<br>0,89	**0,80**				**1,43**	**1,99**	**6,16**
ADP-dependent glucokinase	Q9BRR6|ADPGK_HUMAN	0,81	0,93	0,90						

		0,95	0,97							
ADP-ribose pyrophosphatase, mitochondrial precursor	Q9BW91|NUDT9_HUMAN	1,07	0,99							
ADP-ribosyl cyclase 2 precursor	Q10588|BST1_HUMAN							**1,36**	**0,77**	0,86
ADP-ribosylation factor 3	P61204|ARF3_HUMAN	1,21<br>**1,48**	**1,30**<br>**1,26**	**2,58**	1,22<br>0,88	**1,41**<br>0,96	**1,31**<br>0,57	1,38	**3,85**	**5,11**
ADP-ribosylation factor 4	P18085|ARF4_HUMAN	0,77	2,38	**5,40**						
ADP-ribosylation factor 5	P84085|ARF5_HUMAN				0,78	1,08	0,69			
ADP-ribosylation factor GTPase-activating protein 2	Q8N6H7|ARFG2_HUMAN				0,56	**0,66**	0,37			
ADP-ribosylation factor-like protein 15	Q9NXU5|ARL15_HUMAN	1,25	1,13							
ADP-ribosylation factor-like protein 6-interacting protein 1	Q15041|AR6P1_HUMAN	**0,77**	1,09	**1,07**						
ADP-ribosylation factor-like protein 6-interacting protein 4	Q66PJ3|AR6P4_HUMAN							0,00	0,00	0,00
ADP-ribosylation factor-like protein 8A	Q96BM9|ARL8A_HUMAN	1,35<br>1,65	1,62<br>1,14	1,58						
ADP-ribosylation factor-like protein 8B	Q9NVJ2|ARL8B_HUMAN	1,06<br>1,18	**1,22**<br>1,04	1,09						
ADP-ribosylation factor-like 3	Q5JSM2|Q5JSM2_HUMAN				0,72	0,91	0,61			
Adrenodoxin, mitochondrial precursor	P10109|ADX_HUMAN	**0,78**<br>0,96	**0,61**<br>**0,86**	**0,41**	**2,04**<br>**1,60**	**1,44**<br>1,01	**1,85**<br>**1,64**	0,00	0,00	0,00
Adrenodoxin-like protein, mitochondrial precursor	Q6P4F2|ADXL_HUMAN	0,59	0,68							
Aflatoxin B1 aldehyde reductase member 2	O43488|ARK72_HUMAN	0,94<br>1,00	0,94<br>1,01	0,85	0,93<br>0,93	1,03<br>0,90	**1,25**<br>0,82			
Aging-associated gene 6 protein	Q2TSD3|Q2TSD3_HUMAN	4,20	7,49	11,67	**1,39**<br>**1,30**	1,12<br>0,99	0,95<br>1,05			
Aging-associated gene 7 protein								0,00	0,00	0,00
Aging-associated protein 2	Q2TU77|Q2TU77_HUMAN				**0,81**<br>**0,60**	**0,88**<br>**0,76**	0,84<br>0,85			
AH receptor-interacting protein	O00170|AIP_HUMAN				0,99	1,03	0,91			
AHCY protein	Q1RMG2|Q1RMG2_HUMAN				0,82	1,05	1,10			
AKAP8 protein	Q8NE02|Q8NE02_HUMAN							0,94	1,04	1,08
A-kinase anchor protein 13	Q12802|AKP13_HUMAN				0,93	1,03	0,88			
AKR1A1 protein	Q6IAZ4|Q6IAZ4_HUMAN				0,85<br>**0,89**	0,91<br>**0,89**	0,81<br>**0,76**			
AKR1B1 protein	Q6FGA4|Q6FGA4_HUMAN	1,04	1,01		0,95<br>0,95	1,01<br>**1,30**	1,01<br>0,74			
Alanyl-tRNA synthetase, cytoplasmic	P49588|SYAC_HUMAN				**0,88**<br>0,92	**0,82**<br>1,03	**0,77**<br>0,81			
Alcohol dehydrogenase 5 (Class III), chi polypeptide	Q6IRT1|Q6IRT1_HUMAN				0,88	0,96	0,95			
Aldehyde dehydrogenase 1 family, member A1	Q5SYR1|Q5SYR1_HUMAN				0,00	0,00	0,00			
Aldehyde dehydrogenase 18 family, member A1	Q5T567|Q5T567_HUMAN	0,96<br>1,01	0,86<br>1,00	**0,69**						
Aldehyde dehydrogenase 3B1	P43353|AL3B1_HUMAN	1,26<br>0,99	0,83<br>**0,86**	0,88						
Aldehyde dehydrogenase 4 family, member A1	Q5JNV6|Q5JNV6_HUMAN	0,95<br>1,01	0,89<br>**0,89**	**0,75**						
Aldehyde dehydrogenase 6A1 variant (Fragment)	Q53H94|Q53H94_HUMAN	1,01	0,94							
Aldehyde dehydrogenase family 16 member A1	Q8IZ83|A16A1_HUMAN				1,03<br>0,96	1,02<br>0,91	1,16<br>1,19			
Aldehyde dehydrogenase X, mitochondrial precursor	P30837|AL1B1_HUMAN	1,05	1,07							
ALDH3A2 protein	Q6I9T3|Q6I9T3_HUMAN	0,93<br>1,02	1,10<br>1,07	1,01						
Aldose 1-epimerase	Q96C23|GALM_HUMAN				0,99<br>0,00	0,96<br>0,00	0,90<br>0,00			
Alkyldihydroxyacetonephosphate synthase, peroxisomal precursor	O00116|ADAS_HUMAN	1,05<br>0,94	1,00<br>1,00	**0,86**				0,65	**1,80**	**2,56**
Allograft inflammatory factor 1	Q4V347|Q4V347_HUMAN				0,92<br>**0,71**	1,05<br>**0,72**	0,91<br>**0,64**	**1,49**	**0,44**	**0,40**
Allograft inflammatory factor 1 (Fragment)	Q5STX8|Q5STX8_HUMAN							**0,72**	**0,19**	**0,15**
All-trans-retinol 13,14-reductase precursor	Q6NUM9|RETST_HUMAN	0,91<br>0,99	0,66<br>1,07	0,57						
Alpha subunit of GsGTP binding protein (Fragment)	Q14455|Q14455_HUMAN	1,09	0,77	0,99						
Alpha-1,6-mannosyl-glycoprotein 2-beta-N-acetylglucosaminyltransferase	Q10469|MGAT2_HUMAN	1,11<br>1,15	0,71<br>0,84	**0,64**						
Alpha-1-antitrypsin precursor	P01009|A1AT_HUMAN				**1,79**	1,17	**1,64**			
Alpha-2-HS-glycoprotein precursor	P02765|FETUA_HUMAN	1,30	1,37	2,24						
Alpha-2-macroglobulin precursor	P01023|A2MG_HUMAN	0,87<br>0,70	0,58<br>0,59	0,89						
Alpha-2-macroglobulin receptor-associated protein precursor	P30533|AMRP_HUMAN	1,03<br>1,02	0,94<br>**1,15**	0,92	1,66	0,90	1,50	**1,54**<br>**1,49**	**6,37**<br>**4,93**	**7,92**<br>**6,72**
Alpha-actinin-4	O43707|ACTN4_HUMAN	1,62	**1,23**		0,90<br>0,86	**1,29**<br>**1,25**	**1,50**<br>**1,37**	0,56	0,14	**0,11**
Alpha-centractin	P61163|ACTZ_HUMAN				0,88<br>0,91	0,91<br>1,16	**1,28**<br>**1,40**			
Alpha-enolase	P06733|ENOA_HUMAN				**0,84**<br>**0,87**	0,96<br>0,99	**0,93**<br>**0,84**			
Alpha-galactosidase A (Fragment)	Q6LER7|Q6LER7_HUMAN	1,04<br>0,99	1,20<br>0,96	1,03						
Alpha-KG-E2	Q7LDY7|Q7LDY7_HUMAN	0,97<br>0,95	**0,88**<br>1,07	**0,77**	1,74<br>**2,03**	0,95<br>1,19	1,69<br>**1,59**	**1,48**<br>**1,55**	**2,92**<br>1,48	**5,18**<br>**3,62**
Alpha-L-iduronidase precursor	P35475|IDUA_HUMAN	1,36	0,77	1,64				0,00	0,00	0,00
Alpha-mannosidase 2	Q16706|MA2A1_HUMAN	1,13<br>**1,18**	**0,45**<br>1,02	**0,64**						
Alpha-N-acetylgalactosaminidase precursor	P17050|NAGAB_HUMAN	**1,48**<br>0,88	**1,89**<br>0,95	**1,49**	2,19	1,36	1,66			
Alpha-N-acetylglucosaminidase precursor	P54802|ANAG_HUMAN	0,94	**0,85**							
Alpha-N-acetylglucosaminidase	Q14769|Q14769_HUMAN	1,33	1,71	1,38	**1,81**	1,27	2,26			
Alpha-soluble NSF attachment protein	P54920|SNAA_HUMAN	0,88<br>0,97	1,01<br>0,92	**0,83**	1,13<br>1,15	1,10<br>1,04	**1,44**<br>0,88			
Alpha-taxilin	P40222|TXLNA_HUMAN				0,72	**0,50**	0,57	0,89	0,85	0,83
Amine oxidase [flavin-containing] B	P27338|AOFB_HUMAN	**0,87**	1,01							
Aminopeptidase B	Q9H4A4|AMPB_HUMAN				0,52	0,69	0,74			

Amplified in osteosarcoma	Q9BW99|Q9BW99_HUMAN	0,83	0,79	0,95						
Amyloid-like protein 2 precursor	Q06481|APLP2_HUMAN	0,73	0,33	0,45						
Anamorsin	Q6FI81|CPIN1_HUMAN				0,95	0,91	0,83			
Angiotensin-converting enzyme, somatic isoform precursor	P12821|ACE_HUMAN	**1,35**<br>1,14	1,06<br>0,95	1,10						
Anion exchanger 2 type a variant (Fragment)	Q59GF1|Q59GF1_HUMAN	1,21	0,87	0,98						
ANKRD26-like family C member 1A	Q6S8J3|A26CA_HUMAN							1,87	1,28	1,21
ANKRD26-like family C member 1B	A5A3E0|A26CB_HUMAN				0,00	0,00	0,00			
Ankyrin repeat and SAM domain-containing protein 1A	Q92625|ANS1A_HUMAN	0,00<br>0,00	0,00<br>0,00	0,00						
Ankyrin repeat domain-containing protein 35	Q8N283|ANR35_HUMAN	0,00	0,00							
Ankyrin-2	Q01484|ANK2_HUMAN	0,90	1,06							
Annexin A1	P04083|ANXA1_HUMAN	**1,95**<br>**1,66**	**2,78**<br>**1,88**	**5,45**	**1,11**<br>0,95	**1,08**<br>**1,06**	**1,46**<br>1,28	**1,66**<br>**1,20**	**4,13**<br>**3,37**	**8,09**<br>**5,21**
Annexin A11	Q5T0G8|Q5T0G8_HUMAN	**1,31**	1,08	**1,47**						
Annexin A11 variant (Fragment)	Q59EP1|Q59EP1_HUMAN				**1,28**	1,00	**1,38**			
Annexin A2	P07355|ANXA2_HUMAN	**1,53**<br>1,87	**1,50**<br>**1,32**	**2,52**	**1,50**<br>**1,29**	**1,17**<br>0,82	**1,85**<br>**1,18**	**1,39**<br>**0,82**	**1,23**<br>**1,07**	1,70<br>**1,24**
Annexin A5	P08758|ANXA5_HUMAN	**2,42**<br>**1,83**	**3,26**<br>**1,95**	**6,40**	1,06<br>0,95	1,02<br>**1,11**	**1,91**<br>1,74	**1,98**<br>0,00	**3,03**<br>0,00	**3,31**<br>0,00
Annexin A6	P08133|ANXA6_HUMAN	**1,70**	**1,69**							
Annexin A7	Q5T0M7|Q5T0M7_HUMAN	1,31	1,27	1,97	**1,14**<br>**1,28**	**1,14**<br>**1,20**	**1,86**<br>**1,67**			
Annexin VII isoform 1 variant (Fragment)	Q53HM8|Q53HM8_HUMAN	**1,74**	1,52							
Antigen KI-67	P46013|KI67_HUMAN							0,81<br>1,04	0,67<br>1,00	0,58<br>0,88
ANXA11 protein (Fragment)	Q6ICS0|Q6ICS0_HUMAN	**1,29**	**1,24**		**1,32**	**0,88**	**1,42**	**3,16**<br>0,77	1,20<br>1,15	**2,01**<br>1,04
ANXA4 protein	Q6P452|Q6P452_HUMAN	**1,22**<br>**1,39**	**1,20**<br>**1,18**	**1,37**	1,25	1,04	1,47			
ANXA4 protein (Fragment)	Q6LES2|Q6LES2_HUMAN				**1,28**	1,07	**1,91**			
AP-1 complex subunit gamma-1	O43747|AP1G1_HUMAN	1,10	1,00		1,24	1,03	1,25			
AP1B1 protein	Q86X54|Q86X54_HUMAN	1,03<br>1,15	0,81<br>0,86	0,85	1,20<br>1,48	1,40<br>1,60	1,51<br>1,86			
AP-2 complex subunit alpha-1	O95782|AP2A1_HUMAN	0,99<br>1,04	**0,62**<br>**0,80**	**0,67**				**0,43**	**0,55**	**0,50**
AP-2 complex subunit mu-1	Q96CW1|AP2M1_HUMAN	1,12<br>**1,18**	**0,62**<br>**0,86**	**0,59**						
AP-3 complex subunit delta-1	O14617|AP3D1_HUMAN							1,26	1,38	1,67
AP-3 complex subunit sigma-1	Q92572|AP3S1_HUMAN				**0,48**	0,67	0,42	1,16	4,58	4,83
APEX nuclease (Multifunctional DNA repair enzyme) 1	Q5TZP7|Q5TZP7_HUMAN				0,82<br>**0,68**	1,01<br>1,04	**0,79**<br>0,86	1,10<br>**0,57**	**0,57**<br>**0,58**	**0,60**<br>**0,46**
Apolipoprotein A-II precursor	P02652|APOA2_HUMAN	0,93	1,21	**2,59**						
Apolipoprotein B-100 receptor	Q0VD83|AB48R_HUMAN	1,47	1,45		**1,99**<br>1,41	**1,48**<br>1,10	**1,38**<br>0,98			
Apolipoprotein C-I precursor	P02654|APOC1_HUMAN	0,99<br>0,97	0,72<br>1,98	0,99						
Apolipoprotein E precursor	P02649|APOE_HUMAN	**0,76**<br>**0,81**	**0,26**<br>**0,68**	**0,30**	0,96<br>1,21	0,44<br>0,38	0,63<br>**0,40**	1,12<br>1,31	**1,57**<br>1,22	**1,46**<br>1,40
Apolipoprotein L2 variant (Fragment)	Q59GW9|Q59GW9_HUMAN				**1,66**	**1,16**	1,01			
Apolipoprotein O precursor	Q9BUR5|APOO_HUMAN	0,00<br>0,91	0,00<br>**0,83**	0,00				**0,70**<br>1,57	0,93<br>1,00	**1,31**<br>1,53
Apolipoprotein O-like precursor	Q6UXV4|APOOL_HUMAN	0,97	1,07	0,89						
Apolipoprotein-L2	Q9BQE5|APOL2_HUMAN	0,91<br>**1,16**	1,19<br>**1,12**	**1,56**	**1,38**	1,07	0,89	0,80	2,04	1,46
Apoptosis inhibitor 5	Q9BZZ5|API5_HUMAN				1,21	1,83	1,11	1,00<br>0,84	**0,30**<br>**0,51**	**0,25**<br>**0,48**
Apoptosis-associated speck-like protein containing a CARD	Q9ULZ3|ASC_HUMAN	0,00	0,00	0,00	**0,68**<br>0,55	0,84<br>**0,76**	0,86<br>**0,58**			
Apoptosis-inducing factor 1, mitochondrial precursor	O95831|AIFM1_HUMAN	**0,84**<br>**0,93**	0,92<br>**0,93**	**0,67**	1,79	1,36	1,32	0,76	0,97	1,26
Apoptotic chromatin condensation inducer in the nucleus	Q9UKV3|ACINU_HUMAN							0,94<br>0,96	**0,48**<br>**0,81**	**0,25**<br>**0,55**
Arachidonate 5-lipoxygenase	P09917|LOX5_HUMAN	1,09<br>**0,81**	1,21<br>1,02	**1,63**	0,86	0,83	0,41	1,26	**7,91**	**9,09**
Arachidonate 5-lipoxygenase-activating protein	P20292|AL5AP_HUMAN	1,13<br>**0,76**	**1,29**<br>0,94	1,24						
ARF GTPase-activating protein GIT2	Q14161|GIT2_HUMAN	0,99	1,42	2,64	0,00	0,00	0,00			
ARF6 protein	Q6FH17|Q6FH17_HUMAN	0,00	0,00	0,00						
Arginine and glutamate-rich protein 1	Q9NWB6|ARGL1_HUMAN							1,12<br>**1,23**	**0,38**<br>**0,71**	**0,27**<br>0,69
Arginine/serine-rich coiled-coil protein 1	Q96IZ7|RSRC1_HUMAN							0,00<br>0,00	0,00<br>0,00	0,00<br>0,00
Arginine/serine-rich coiled-coil protein 2	Q7L4I2|RSRC2_HUMAN							0,91	0,45	0,25
Arginine/serine-rich splicing factor 6 variant (Fragment)	Q59GY3|Q59GY3_HUMAN							**1,21**	**0,47**	**0,44**
Arginine/serine-rich splicing factor 7 type B	Q564D3|Q564D3_HUMAN							**0,85**<br>1,14	**0,30**<br>**0,67**	**0,18**<br>**0,66**
Arginyl-tRNA synthetase, cytoplasmic	P54136|SYRC_HUMAN			16,96	**0,73**<br>0,85	0,87<br>0,98	**0,73**<br>0,85			
ARHA protein (Fragment)	Q9BVT0|Q9BVT0_HUMAN							1,28	2,29	**4,06**
ARHG protein (Fragment)	Q6ICQ8|Q6ICQ8_HUMAN	1,10<br>**1,14**	1,05<br>0,97	1,22	1,45	1,04	0,94	1,58	2,47	3,90
ARHGDIA protein	Q6IBM5|Q6IBM5_HUMAN				0,94<br>0,93	1,02<br>1,12	1,01<br>1,07			
ARHGEF1 protein	Q6NX52|Q6NX52_HUMAN				0,77	0,73	**0,47**			
Armadillo repeat-containing protein 1	Q9NVT9|ARMC1_HUMAN	1,04	0,98							
Armadillo repeat-containing protein 10	Q8N2F6|ARM10_HUMAN	1,50	**1,40**							
Armadillo repeat-containing X-linked protein 3	Q9UH62|ARMX3_HUMAN	1,08	1,15							
ARP3 actin-related protein 3 homolog variant (Fragment)	Q9Y2Q5|MAPIP_HUMAN	1,42	1,24	1,39	0,92<br>1,05	**0,83**<br>1,04	1,03<br>1,10	1,15	**1,61**	**1,88**
Arrestin beta 2 isoform 1 variant (Fragment)	Q59EM5|Q59EM5_HUMAN									

		1,01	0,86							
Arsenite-resistance protein 2	Q9BXP5|ARS2_HUMAN							1,04	**0,39**	**0,33**
Arsenate resistance protein ARS2	A4D2E6|A4D2E6_HUMAN							 **0,78**	 **0,45**	 **0,35**
Aryl hydrocarbon receptor interacting protein	A0SZW6|A0SZW6_HUMAN				 0,76	 0,97	 0,79			
Arylacetamide deacetylase-like 1	Q6PIU2|ADCL1_HUMAN	0,97<br>**0,91**	**1,10**<br>**1,15**	1,01						
Arylsulfatase B precursor	P15848|ARSB_HUMAN	1,20<br>1,05	**1,62**<br>0,92	1,14	1,78<br>1,61	0,89<br>0,83	1,56<br>1,14			
Asparaginyl-tRNA synthetase variant (Fragment)	Q53GU6|Q53GU6_HUMAN				**0,68**	0,96	**0,71**			
Aspartate aminotransferase, mitochondrial precursor	P00505|AATM_HUMAN	0,93<br>**0,87**	**0,82**<br>**0,87**	**0,60**	**1,59**<br>**1,89**	**1,38**<br>**1,38**	**2,14**<br>**2,21**			
Aspartyl/asparaginyl beta-hydroxylase	Q12797|ASPH_HUMAN	0,98<br>1,02	1,02<br>1,09	0,95				0,94<br>0,88	1,39<br>**2,19**	1,73<br>**3,30**
Aspartyl-tRNA synthetase, cytoplasmic	P14868|SYDC_HUMAN	 0,00	 0,00		0,80<br>1,69	0,98<br>1,50	**0,80**<br>1,88			
Ataxin-2-like protein	Q8WWM7|ATX2L_HUMAN				1,07<br>1,04	0,89<br>0,85	0,81<br>0,84			
ATF3 protein (Fragment)	Q6ICQ9|Q6ICQ9_HUMAN							0,78<br>0,93	0,54<br>0,45	0,62<br>0,18
Atlastin-3	Q6DD88|ATLA3_HUMAN	1,02<br>0,98	**1,31**<br>**1,17**	**1,44**	**2,58**	**1,30**	**1,66**			
ATP synthase F0 subunit 6	A6YWX8|A6YWX8_HUMAN	1,00<br>1,10	1,08<br>**0,63**	0,71						
ATP synthase gamma chain	Q6I9V2|Q6I9V2_HUMAN	 0,97	 1,01							
ATP synthase gamma chain (Fragment)	Q8TAS0|Q8TAS0_HUMAN	**0,70**	**0,85**	**0,78**	**2,15**<br>1,86	1,06<br>0,92	1,28<br>1,32	0,88	2,10	3,12
ATP synthase lipid-binding protein, mitochondrial precursor	Q06055|AT5G2_HUMAN	 1,06	 **0,91**						**4,09**	**12,31**
ATP synthase mitochondrial F1 complex assembly factor 2, mitochondrial precursor	Q8N5M1|ATPF2_HUMAN	0,00<br>0,88	0,00<br>0,88	0,00						
ATP synthase protein 8	A0S8D6|A0S8D6_HUMAN	0,00<br>0,90	0,00<br>1,06	0,00						
ATP synthase subunit a	Q4GDK0|Q4GDK0_HUMAN	 1,04	 0,92							
ATP synthase subunit alpha, mitochondrial precursor	P25705|ATPA_HUMAN	1,00<br>1,01	0,97<br>1,02	**0,84**	**2,45**<br>2,61	**1,37**<br>1,34	**1,75**<br>1,58	1,11<br>0,86	**2,53**<br>**2,08**	**7,44**<br>**4,21**
ATP synthase subunit beta, mitochondrial precursor	P06576|ATPB_HUMAN	0,98<br>**0,96**	0,95<br>0,97	**0,80**	**1,91**<br>2,11	1,12<br>**1,16**	**1,55**<br>1,11	0,76<br>0,97	**3,41**<br>**1,85**	**7,94**<br>**4,75**
ATP synthase subunit d, mitochondrial	O75947|ATP5H_HUMAN	0,93<br>**1,06**	0,94<br>**1,05**	**0,79**	 **1,37**	 0,87	 0,93	1,11	**2,72**	**6,29**
ATP synthase subunit e, mitochondrial	P56385|ATP5I_HUMAN	1,06<br>0,96	1,00<br>0,97	0,89	 **2,62**	 **1,48**	 **1,33**	1,03	7,53	15,51
ATP synthase subunit g, mitochondrial	O75964|ATP5L_HUMAN	0,90<br>1,04	0,89<br>1,08	**0,81**	 **2,17**	 0,99	 **0,86**	0,75	2,41	**4,68**
ATP synthase subunit O, mitochondrial precursor	P48047|ATPO_HUMAN	0,96<br>0,94	0,94<br>**0,87**	**0,82**	 2,30	 **1,29**	 1,17	0,87<br>1,31	**2,53**<br>2,13	**5,49**<br>**4,47**
ATP synthase subunit s, mitochondrial precursor	Q99766|ATP5S_HUMAN	 0,96	 1,08							
ATP synthase subunit s-like protein	Q9NW81|AT5SL_HUMAN	 1,11	 0,91							
ATP synthase, H+ transporting, mitochondrial F0 complex, subunit b isoform 1 variant (Fragment)	Q53GB3|Q53GB3_HUMAN				**1,78**<br>**2,53**	0,85<br>1,04	0,97<br>1,14	1,41	**5,46**	**13,09**
ATP synthase, H+ transporting, mitochondrial F0 complex, subunit B1	Q5QNZ2|Q5QNZ2_HUMAN							 1,57	 **2,96**	 **9,05**
ATP synthase, H+ transporting, mitochondrial F0 complex, subunit F6	Q6NZ59|Q6NZ59_HUMAN	0,88	**0,85**	**0,70**	 **1,87**	 1,02	 1,31	0,86<br>1,09	3,89<br>**3,53**	**12,85**<br>**10,20**
ATP1B1 protein (Fragment)	Q6LEU2|Q6LEU2_HUMAN	**1,73**<br>1,06	**1,30**<br>**1,17**	1,32						
ATP5D protein	Q6FG90|Q6FG90_HUMAN	0,96<br>1,05	0,93<br>1,03	**0,70**	 2,66	 **1,44**	 **1,53**	0,78<br>**1,95**	**3,76**<br>**3,71**	**6,39**<br>**7,97**
ATP5G3 protein (Fragment)	Q6LEU9|Q6LEU9_HUMAN	**0,66**	**0,84**	**0,85**	**2,64**<br>**3,24**	1,26<br>**1,87**	1,31<br>2,02	 0,59	 **2,85**	 **6,52**
ATP5J protein	Q6IB54|Q6IB54_HUMAN	 1,07	 **1,10**							
ATP5J2 protein	Q6IBB3|Q6IBB3_HUMAN	 1,24	 1,27							
ATP6V1G1 protein	Q6IB33|Q6IB33_HUMAN	**1,48**<br>0,98	**1,99**<br>1,08	**1,71**	1,09<br>0,84	**0,74**<br>**0,65**	0,92<br>0,79	**1,73**<br>**1,68**	**4,15**<br>**3,99**	**6,58**<br>**6,37**
ATPase family, AAA domain containing 3A (Fragment)	Q5SV24|Q5SV24_HUMAN				 0,00	 **0,00**	 0,00			
ATPase family AAA domain-containing protein 1	Q8NBU5|ATAD1_HUMAN	0,75	0,78	0,65						
ATPase family AAA domain-containing protein 3A	Q9NVI7|ATD3A_HUMAN	0,94<br>0,98	0,95<br>0,97	**0,82**				**0,80**<br>1,08	**0,56**<br>**0,57**	**0,55**<br>**0,72**
ATPase family AAA domain-containing protein 3B	Q5T9A4|ATD3B_HUMAN	 0,89	 1,01							
ATPase inhibitor, mitochondrial precursor	Q9UII2|ATIF1_HUMAN	0,87<br>**1,21**	**0,87**<br>**1,20**	**0,75**	1,91<br>**1,61**	1,24<br>1,09	**1,86**<br>1,19	 1,62	 2,14	 **3,51**
ATPase, Ca++ transporting, plasma membrane 4	B1APW6|B1APW6_HUMAN	4,79	3,58							
ATPase, H+ transporting, lysosomal 31kDa, V1 subunit E isoform 1	Q53Y06|Q53Y06_HUMAN	**1,31**<br>**0,89**	**1,82**<br>0,98	**1,68**	1,12<br>1,12	**0,82**<br>0,95	0,93<br>1,11	**1,50**<br>**1,49**	**3,72**<br>**2,92**	**5,80**<br>**4,51**
ATP-binding cassette sub-family B member 10, mitochondrial precursor	Q9NRK6|ABCBA_HUMAN	1,24<br>**0,86**	1,09<br>0,90	1,09						
ATP-binding cassette sub-family B member 7, mitochondrial precursor	O75027|ABCB7_HUMAN	1,15<br>**0,86**	1,20<br>0,96	1,20						
ATP-binding cassette sub-family B member 8, mitochondrial precursor	Q9NUT2|ABCB8_HUMAN	1,13<br>1,05	0,80<br>1,00	0,90						
ATP-binding cassette sub-family D member 1	P33897|ABCD1_HUMAN	1,03<br>**0,85**	0,91<br>0,89	0,81						
ATP-binding cassette sub-family D member 3	P28288|ABCD3_HUMAN	1,11<br>0,98	1,04<br>1,12	0,98						
ATP-binding cassette sub-family E member 1	P61221|ABCE1_HUMAN	0,62		2,81	**0,83**	1,00	0,97			
ATP-binding cassette sub-family F member 1	Q8NE71|ABCF1_HUMAN							 0,00	 0,00	 0,00
ATP-binding cassette, sub-family C, member 3 isoform MRP3 variant (Fragment)	Q59H05|Q59H05_HUMAN	 1,06	 0,98							
ATP-dependent Clp protease ATP-binding subunit clpX-like, mitochondrial precursor	O76031|CLPX_HUMAN	0,92<br>**0,83**	0,66<br>0,83	0,57						
ATP-dependent DNA helicase 2 subunit 1	P12956|KU70_HUMAN				0,99<br>0,99	**1,63**<br>**1,50**	**1,72**<br>1,23	**1,38**<br>**0,64**	**1,42**<br>**1,38**	**1,96**<br>**1,48**
ATP-dependent DNA helicase 2 subunit 2	P13010|KU86_HUMAN				**1,14**	**1,53**	**1,49**	**1,25**	**1,47**	**2,35**
ATP-dependent metalloprotease YME1L1	Q96TA2|YMEL1_HUMAN	**0,83**<br>1,03	0,85<br>1,10	0,94						
ATP-dependent RNA helicase A	Q08211|DHX9_HUMAN							**1,51**<br>**0,20**	**0,47**<br>**0,32**	**0,68**<br>**0,29**
ATP-dependent RNA helicase DDX1	Q92499|DDX1_HUMAN				 0,00	 0,00	 0,00	1,30	0,24	0,60
ATP-dependent RNA helicase DDX18	Q9NVP1|DDX18_HUMAN							0,73	0,41	0,43

								0,38	0,71	0,48
ATP-dependent RNA helicase DDX19B	Q9UMR2|DD19B_HUMAN				**0,77**	0,90	**0,55**			
ATP-dependent RNA helicase SUPV3L1, mitochondrial precursor	Q8IYB8|SUV3_HUMAN	1,06	1,00							
ATWD578	Q6UW11|Q6UW11_HUMAN	0,90	**0,85**							
Autophagy-related protein 3	Q9NT62|ATG3_HUMAN				0,78	0,82	0,92			
AVLL5809	Q6UWH2|Q6UWH2_HUMAN	1,05	1,05							
B2M protein	Q6IAT8|Q6IAT8_HUMAN	1,08	1,05	1,04	**1,61**<br>1,33	0,92<br>0,87	**1,41**<br>0,99	1,32<br>1,05	**3,48**<br>1,89	**3,68**<br>2,15
B3GAT3 protein (Fragment)	Q5U676|Q5U676_HUMAN	1,01	0,99							
BAK1 protein	Q6I9T6|Q6I9T6_HUMAN	0,95	**0,82**							
Band 4,1-like protein 3	Q9Y2J2|E41L3_HUMAN				1,87	**1,79**	1,83			
Barrier-to-autointegration factor	O75531|BAF_HUMAN	0,89	0,99					**0,90**<br>0,72	**1,28**<br>**1,61**	**1,26**<br>**2,60**
Basic leucine zipper and W2 domain-containing protein 1	Q7L1Q6|BZW1_HUMAN				**0,69**<br>**0,57**	**0,81**<br>**0,68**	**0,66**<br>**0,45**			
Basigin precursor	P35613|BASI_HUMAN							**2,21**	**2,12**	**2,89**
Bax-sigma	Q9NYG7|Q9NYG7_HUMAN	**1,72**<br>**1,54**	**3,38**<br>**1,81**	**4,40**	0,90	0,56	0,34	0,82	1,46	**2,54**
B-cell CLL/lymphoma 7 protein family member B	Q9BQE9|BCL7B_HUMAN							0,00	0,00	0,00
B-cell CLL/lymphoma 7 protein family member C	Q8WUZ0|BCL7C_HUMAN							0,78	**0,15**	**0,11**
B-cell receptor-associated protein 31	P51572|BAP31_HUMAN	**0,90**<br>1,02	**0,82**<br>**1,13**	**0,61**	**1,78**<br>**1,70**	**1,67**<br>**1,41**	**1,78**<br>1,47	1,10	**2,30**	**2,27**
B-cell receptor-associated protein 31 variant (Fragment)	Q53HT6|Q53HT6_HUMAN							**1,21**	**1,70**	**2,26**
BCKDK protein	Q96G95|Q96G95_HUMAN	0,85<br>1,05	0,88<br>1,09	0,77				1,09<br>0,49	1,04<br>1,40	**1,71**<br>1,88
Bcl10-interacting CARD protein	Q96LW7|BINCA_HUMAN	0,95<br>0,97	**0,71**<br>0,89	**0,49**						
BCL2-associated athanogene 3 variant (Fragment)	Q53GY1|Q53GY1_HUMAN				0,43	0,20				
Bcl-2-associated transcription factor 1	Q9NYF8|BCLF1_HUMAN							0,97<br>**1,18**	**0,56**<br>**1,15**	**0,25**<br>**0,67**
Bcl-2-like 13 protein	Q9BXK5|B2L13_HUMAN	1,08<br>1,15	1,01<br>1,17	1,10						
Benzodiazapine receptor	Q53Y59|Q53Y59_HUMAN				1,75	1,57	1,64	0,97	0,98	1,25
Beta actin variant (Fragment)	Q53GK6|Q53GK6_HUMAN				**0,92**<br>**0,92**	**1,10**<br>**1,10**	**0,95**<br>0,92	**0,72**	**0,76**	**0,67**
Beta-1-syntrophin	Q13884|SNTB1_HUMAN	**1,31**	1,14							
Beta-2-glycoprotein 1 precursor	P02749|APOH_HUMAN	**1,19**<br>**0,84**	**1,85**<br>**1,27**	**3,15**	**1,98**<br>**1,39**	**1,89**<br>**1,28**	**3,95**<br>**2,64**	1,32<br>1,19	**5,19**<br>**6,71**	**6,26**<br>**8,13**
Beta-2-microglobulin	Q540F8|Q540F8_HUMAN	1,11	**1,27**							
Beta-centractin	P42025|ACTY_HUMAN				0,00	0,00	0,00			
Beta-galactosidase precursor	P16278|BGAL_HUMAN	1,02	0,93							
Beta-galactoside alpha-2,6-sialyltransferase 1	P15907|SIAT1_HUMAN	1,03	0,92							
Beta-galactosidase (Fragment)	Q53G40|Q53G40_HUMAN	1,17	**1,58**	**1,27**						
Beta-glucuronidase precursor	P08236|BGLR_HUMAN	1,01<br>0,94	**1,26**<br>1,00	**1,16**	**1,27**	1,09	1,25			
Beta-hexosaminidase beta chain precursor	P07686|HEXB_HUMAN	1,11<br>1,00	**1,32**<br>**0,88**	1,05	**1,78**<br>1,58	0,95<br>1,00	**1,54**<br>1,30			
Beta-lactamase-like protein 2	Q53H82|LACB2_HUMAN	1,05<br>0,95	1,65<br>**0,80**	3,15						
BH3-interacting domain death agonist	P55957|BID_HUMAN				0,54<br>0,00	**0,50**<br>0,00	0,31<br>0,00	1,37	**9999,00**<br>5,42	**9999,00**<br>12,61
Bifunctional aminoacyl-tRNA synthetase	P07814|SYEP_HUMAN	1,46	**1,89**	**4,28**	**0,76**<br>0,89	**1,08**<br>**1,19**	**0,83**<br>0,99			
Bifunctional coenzyme A synthase	Q13057|COASY_HUMAN	0,58	0,94		0,00	0,00	0,00			
Bifunctional methylenetetrahydrofolate dehydrogenase/cyclohydrolase, mitochondrial precursor	P13995|MTDC_HUMAN	0,96<br>0,89	0,96<br>**0,88**	0,86				**0,79**<br>0,94	**0,58**<br>**0,70**	**0,31**<br>**0,48**
Bifunctional purine biosynthesis protein PURH	P31939|PUR9_HUMAN				0,00<br>1,29	0,00<br>1,09	0,00<br>0,93			
Biliverdin reductase A precursor	P53004|BIEA_HUMAN				0,97<br>**0,85**	1,09<br>0,93	0,93<br>**0,70**			
Bladder cancer related CD9 variant	Q86TK9|Q86TK9_HUMAN							2,57	2,28	2,07
Bleomycin hydrolase	Q13867|BLMH_HUMAN	0,00	0,00		0,00	0,00	0,00			
BolA-like protein 3	Q53S33|BOLA3_HUMAN	0,96<br>0,90	0,83<br>**0,89**	0,65						
Bone marrow stromal antigen 2 precursor	Q10589|BST2_HUMAN	1,08<br>1,06	**0,72**<br>0,88	0,73						
BORIS-like protein	A0S6W1|A0S6W1_HUMAN							0,61	**0,42**	**0,55**
Brain acid soluble protein 1	P80723|BASP_HUMAN	1,30<br>1,16	1,61<br>1,06	1,77	0,00<br>0,86	0,00<br>1,10	0,00<br>1,31	**2,03**<br>2,26	1,52<br>1,89	1,26<br>2,29
Brain protein 44	O95563|BR44_HUMAN	0,87<br>1,01	0,80<br>0,95	0,69						
Brain protein 44-like protein	Q9Y5U8|BR44L_HUMAN	0,95	1,05	0,83						
Branched chain keto acid dehydrogenase E1, alpha polypeptide variant (Fragment)	Q59EI3|Q59EI3_HUMAN	0,00<br>0,93	0,00<br>0,91	0,00						
Branched-chain-amino-acid aminotransferase, mitochondrial precursor	O15382|BCAT2_HUMAN	0,82<br>1,09	0,81<br>0,96	0,63						
Breast carcinoma-amplified sequence 2	O75934|BCAS2_HUMAN							0,82	**0,34**	0,17
Breast carcinoma amplified sequence 2 variant (Fragment)	Q53HE3|Q53HE3_HUMAN							0,00	0,00	0,00
BRI3-binding protein	Q8WY22|BRI3B_HUMAN	1,03<br>0,89	1,04<br>1,15	1,08				1,11	2,35	2,25
Bridging integrator 2	Q9UBW5|BIN2_HUMAN				**0,39**	0,75	0,57			
BTB/POZ domain-containing protein 14A	Q96BF6|BTBDE_HUMAN	0,00<br>0,00	0,00<br>0,00	0,00	0,73	0,81	1,16	0,00<br>0,00	0,00<br>0,00	0,00<br>0,00
BTB/POZ domain-containing protein KCTD12	Q96CX2|KCD12_HUMAN	0,00	0,00	0,00	1,02<br>0,99	1,65<br>**1,39**	1,18<br>1,28	0,00<br>0,89	0,00<br>1,30	0,00<br>1,48
BZRP protein	Q6ICF9|Q6ICF9_HUMAN							1,54	1,17	1,54
C10orf54 protein (Fragment)	Q2TA85|Q2TA85_HUMAN	1,23	0,93							
C14orf159 protein	Q6IA66|Q6IA66_HUMAN	**1,21**	1,03							

C1orf151 protein (Fragment)	Q96G68|Q96G68_HUMAN	 0,84	 1,04					0,00	0,00	0,00
C1orf212 protein	Q49AP7|Q49AP7_HUMAN	0,00	0,00	0,00						
C1-tetrahydrofolate synthase	Q6UB35|Q6UB35_HUMAN	0,93	1,07	0,96						
C-1-tetrahydrofolate synthase, cytoplasmic	P11586|C1TC_HUMAN				0,80<br>0,68	0,90<br>**0,82**	0,83<br>**0,71**	 0,94	 0,83	 0,80
C5a anaphylatoxin chemotactic receptor	P21730|C5AR_HUMAN	1,16<br>1,02	0,87<br>1,14	1,23	 1,69	 0,81	 1,24			
CAAX prenyl protease 1 homolog	O75844|FACE1_HUMAN	0,92<br>0,98	1,15<br>0,96	1,07						
CAG-ISL 7	Q45RF0|Q45RF0_HUMAN				0,00<br>0,00	0,00<br>0,00	0,00<br>0,00			
Calcium and integrin-binding protein 1	Q99828|CIB1_HUMAN	 0,91	 **0,77**							
Calcium-binding atopy-related autoantigen 1	Q9BPX6|CBAA1_HUMAN	**0,76**<br>**0,92**	**0,73**<br>0,94	**0,63**						
Calcium-binding mitochondrial carrier protein Aralar2	Q9UJS0|CMC2_HUMAN	1,07<br>0,91	0,93<br>1,00	**0,85**						
Calcium-binding mitochondrial carrier protein SCaMC-1	Q6NUK1|SCMC1_HUMAN	0,95<br>0,94	1,09<br>**0,93**	0,98				1,03	1,59	**3,53**
Calcium-binding mitochondrial carrier protein SCaMC-2	Q6KCM7|SCMC2_HUMAN	0,95<br>0,81	0,97<br>0,82	0,99						
Calcium-binding protein 39	Q9Y376|CAB39_HUMAN				1,08<br>0,70	1,15<br>**0,76**	0,87<br>0,72			
Calcium-binding protein p22	Q99653|CHP1_HUMAN	0,93<br>0,93	1,00<br>1,01	1,08	0,99<br>0,00	**0,62**<br>0,00	0,73<br>0,00	1,89	**4,85**	**6,08**
Calcium-independent phospholipase A2-gamma	Q9NP80|PLPL8_HUMAN	0,96<br>1,00	0,49<br>1,06	0,66						
Calcium-transporting ATPase type 2C member 1	P98194|AT2C1_HUMAN	0,00<br>0,95	0,00<br>0,92	0,00						
Calcyclin-binding protein	Q9HB71|CYBP_HUMAN				0,82	**0,81**	0,93			
CALM3 protein	Q9BRL5|Q9BRL5_HUMAN	**1,50**	1,14	0,94				**1,30**	**2,32**	**2,73**
Calmodulin	P62158|CALM_HUMAN	 **1,56**	 1,00		1,05<br>0,72	**1,10**<br>**0,73**	**1,51**<br>1,06	 **1,80**	 **3,92**	 **4,60**
Calnexin precursor	P27824|CALX_HUMAN	**0,91**<br>**0,93**	1,05<br>**1,24**	1,09	**1,90**<br>1,96	0,89<br>0,99	0,94<br>1,02	1,04<br>1,03	**8,46**<br>**5,91**	**15,14**<br>**10,31**
Calpain 2, large [catalytic] subunit variant (Fragment)	Q59EF6|Q59EF6_HUMAN				0,85<br>1,36	0,95<br>2,38	1,02<br>2,94			
Calpain small subunit 1	P04632|CPNS1_HUMAN				**0,79**<br>**0,82**	**0,81**<br>0,73	0,77<br>0,76			
Calpastatin	Q7Z4K0|Q7Z4K0_HUMAN				0,00<br>**0,66**	0,00<br>0,75	0,00<br>0,79			
Calpastatin isoform a variant (Fragment)	Q59HE3|Q59HE3_HUMAN				**0,66**	0,77	**0,69**	 1,27	 **1,89**	 1,49
Calponin-2	Q99439|CNN2_HUMAN				 **0,70**	 0,98	 0,77			
CALR protein	Q6IAT4|Q6IAT4_HUMAN	**0,85**<br>0,96	0,96<br>**1,20**	**0,88**	**1,79**<br>1,86	**1,16**<br>**1,25**	**2,26**<br>1,74	0,98<br>1,20	**4,64**<br>**3,08**	**6,21**<br>**4,38**
Calumenin precursor	O43852|CALU_HUMAN	1,11<br>0,96	0,97<br>1,04	0,91	**2,08**	**1,57**	**2,46**	1,86	2,30	2,39
cAMP responsive element modulator	Q59FC7|Q59FC7_HUMAN							0,00	0,00	0,00
cAMP-dependent protein kinase catalytic subunit alpha	P17612|KAPCA_HUMAN	0,00	0,00	0,00	0,86<br>0,00	1,13<br>0,00	0,94<br>0,00			
cAMP-dependent protein kinase type I-alpha regulatory subunit	P10644|KAP0_HUMAN				 0,77	 **0,79**	 0,74			
cAMP-dependent protein kinase type II-alpha regulatory subunit	P13861|KAP2_HUMAN				 1,28	 0,92	 1,08			
CAPN1 protein	Q6DHV4|Q6DHV4_HUMAN				1,08<br>1,04	1,08<br>1,10	1,01<br>1,17			
Capping protein (Actin filament) muscle Z-line, alpha 2 variant (Fragment)	Q53GE2|Q53GE2_HUMAN	**1,48**	1,24	**2,16**						
Capping protein (Actin filament) muscle Z-line, beta	Q32Q68|Q32Q68_HUMAN	**1,27**<br>**1,09**	1,16<br>1,08	**1,88**	1,02<br>1,06	**1,11**<br>1,13	**1,12**<br>1,07	1,15	1,32	**1,69**
Capping protein (Actin filament) muscle Z-line, beta	B1AK88|B1AK88_HUMAN							 0,64	 1,48	 1,13
Caprin-1	Q14444|CAPR1_HUMAN				1,57<br>1,10	1,38<br>0,82	1,64<br>0,82	**0,74**<br>0,99	**0,39**<br>**0,70**	**0,30**<br>**0,62**
Capz-interacting protein	Q6JBY9|CPZIP_HUMAN				0,88	0,76	0,78			
Carbonic anhydrase 2	P00918|CAH2_HUMAN				**0,73**<br>**0,85**	1,04<br>1,13	1,01<br>1,11			
Carbonyl reductase [NADPH] 1	P16152|CBR1_HUMAN	 0,97	 0,92		0,64<br>0,63	1,11<br>0,75	1,25<br>0,80			
Carbonyl reductase 4	Q8N4T8|CBR4_HUMAN	 0,89	 0,99							
Carboxypeptidase D precursor	O75976|CBPD_HUMAN	 0,93	 0,90							
Carboxypeptidase M precursor	P14384|CBPM_HUMAN	1,14<br>**1,15**	0,86<br>1,07	0,89	 0,00	 0,00	 0,00	**2,16**<br>**1,40**	0,98<br>0,95	1,04<br>0,91
Carnitine acetyltransferase	Q5T952|Q5T952_HUMAN	1,03	1,04	0,67						
Carnitine O-acetyltransferase	P43155|CACP_HUMAN	 1,07	 1,01							
Carnitine O-palmitoyltransferase 2, mitochondrial precursor	P23786|CPT2_HUMAN	1,01<br>**0,90**	0,96<br>**0,96**	**0,86**						
Carnitine O-palmitoyltransferase I, liver isoform	P50416|CPT1A_HUMAN	1,06<br>0,97	1,07<br>1,11	0,97					4,89	16,40
Carnitine O-palmitoyltransferase I, muscle isoform	Q92523|CPT1B_HUMAN	0,78	0,87	1,30						
Carrier family 6 , member 8 variant (Fragment)	Q59EV6|Q59EV6_HUMAN	**1,24**<br>0,95	**1,43**<br>**0,90**	1,06	 1,89	 **1,10**	 **1,49**			
Casein kinase 2, beta polypeptide	Q5SRQ6|Q5SRQ6_HUMAN							**1,34**	**2,35**	**2,42**
Caseinolytic peptidase B protein homolog	Q9H078|CLPB_HUMAN	**0,88**<br>**0,93**	**0,61**<br>**0,89**	**0,47**	 **1,36**	 0,97	 0,97	0,72	0,29	0,28
Caspase-1 precursor	P29466|CASP1_HUMAN				 **0,70**	 **0,58**	 0,42			
CAST protein (Fragment)	Q05DE8|Q05DE8_HUMAN				 0,32	 0,62	 0,49	0,87	0,90	**0,50**
Cat eye syndrome critical region protein 1 precursor	Q9NZK5|CECR1_HUMAN	1,07<br>0,96	**1,30**<br>**0,89**	**1,19**	**1,66**<br>**2,11**	1,19<br>1,18	**1,64**<br>**1,64**	0,93<br>0,76	1,07<br>**1,78**	**1,56**<br>**1,92**
Cat eye syndrome critical region protein 5 precursor	Q9BXW7|CECR5_HUMAN	0,93<br>**0,86**	1,04<br>**0,89**	0,74						
Catalase	P04040|CATA_HUMAN	0,95<br>**0,91**	0,95<br>1,03	**0,75**	1,00<br>**1,25**	**0,87**<br>0,99	**1,11**<br>1,03			
Catechol O-methyltransferase	P21964|COMT_HUMAN	0,82<br>1,03	1,07<br>**1,18**	0,97	 **1,59**	 0,92	 0,94	1,10	1,99	2,92
Catechol-O-methyltransferase domain-containing protein 1	Q86VU5|CMTD1_HUMAN	0,95	0,76	1,06						
Cathelicidin antimicrobial peptide precursor	P49913|CAMP_HUMAN	1,04<br>0,91	0,82<br>0,89	**1,35**	 1,42	 0,96	 **2,56**			
Cathepsin B precursor	P07858|CATB_HUMAN	0,97<br>**0,89**	1,00<br>**0,79**	**0,76**	**1,93**<br>**1,86**	**0,84**<br>0,85	**0,84**<br>**0,77**			
Cathepsin D precursor	P07339|CATD_HUMAN	**0,91**	**0,80**	**0,66**	**1,91**	**0,87**	**1,38**	1,25	1,38	**1,50**

		**0,91**	**0,78**		1,86	**0,84**	**1,26**	1,15	0,87	0,93
Cathepsin H precursor	P09668|CATH_HUMAN	0,95<br>1,06	1,22<br>0,91	0,95	**1,64**<br>1,78	1,02<br>**1,17**	**1,29**<br>**1,41**			
Cathepsin L1 precursor	P07711|CATL1_HUMAN	0,99<br>1,12	**1,49**<br>1,02	1,20						
Cathepsin L2 precursor	O60911|CATL2_HUMAN				1,34	1,05	1,29	1,22	**1,34**	**1,52**
Cathepsin Z precursor	Q9UBR2|CATZ_HUMAN	0,95<br>**0,87**	0,77<br>**0,81**	**0,67**	**1,77**<br>1,94	**0,82**<br>**0,83**	**0,70**<br>0,83			
Cation chloride cotransporter 6	Q9BYI0|Q9BYI0_HUMAN	1,09<br>1,06	0,91<br>0,95	1,07						
Cation-independent mannose-6-phosphate receptor precursor	P11717|MPRI_HUMAN	0,98<br>**0,86**	**0,70**<br>**0,74**	0,81	0,00	0,00	0,00	0,00	0,00	0,00
CBX5 protein	Q6I9T7|Q6I9T7_HUMAN							**0,79**<br>1,09	**0,31**<br>0,65	**0,21**<br>**0,57**
CCAAT/enhancer-binding protein alpha	P49715|CEBPA_HUMAN							0,78	0,43	0,39
CCAAT/enhancer-binding protein beta	P17676|CEBPB_HUMAN							0,66<br>0,83	**0,05**<br>**0,19**	**0,04**<br>**0,13**
CD14 antigen	P08571|CD14_HUMAN	**1,38**<br>**1,21**	**0,44**<br>**0,63**	**0,36**	1,75<br>**1,87**	0,49<br>**0,53**	0,43<br>**0,44**	**1,02**<br>**1,22**	**0,09**<br>**0,20**	**0,11**<br>**0,20**
CD166 antigen precursor	Q13740|CD166_HUMAN	**1,25**<br>1,12	0,92<br>**1,23**	0,93						
CD2 antigen (P50), sheep red blood cell receptor variant (Fragment)	Q53F96|Q53F96_HUMAN							0,00	0,00	0,00
CD2 antigen cytoplasmic tail-binding protein 2	O95400|CD2B2_HUMAN							1,11	0,36	0,18
CD209 protein	Q2TB19|Q2TB19_HUMAN	1,10	0,86	0,86						
CD276 antigen precursor	Q5ZPR3|CD276_HUMAN	1,21	0,89	1,04						
CD44 antigen precursor	P16070|CD44_HUMAN				1,42	0,99	**1,33**	**1,70**	**1,54**	**1,52**
CD44 molecule (Fragment)	Q9H5A7|Q9H5A7_HUMAN	**1,25**<br>**1,34**	**1,14**<br>**1,29**	**1,74**	**1,63**	1,09	**1,70**	**1,47**	1,16	1,07
CD48 protein	Q8MGR0|Q8MGR0_HUMAN							0,00<br>0,00	0,00<br>0,00	0,00<br>0,00
CD58 protein	Q9BRW0|Q9BRW0_HUMAN	1,28<br>1,07	1,10<br>**1,10**	1,17	0,91	1,13	0,84			
CD59 protein	Q6FHM9|Q6FHM9_HUMAN	1,21<br>1,05	0,78<br>1,16	0,80	1,70	0,97	1,65	**1,84**<br>**1,95**	0,94<br>**1,36**	1,14<br>**1,71**
CD74/ROS fusion protein	A9YLN4|A9YLN4_HUMAN							0,77	**2,99**	**3,54**
CD9 antigen	P21926|CD9_HUMAN	**1,44**<br>**1,34**	0,95<br>**1,28**	1,19	2,07<br>1,73	1,21<br>**0,77**	**1,46**<br>1,09			
CD97 antigen precursor	P48960|CD97_HUMAN	1,13	0,99							
CD99 protein (Fragment)	Q6ICV7|Q6ICV7_HUMAN	0,00<br>**1,25**	0,00<br>0,87	0,00	1,69	0,83	1,13			
CDC37 protein	Q6FG59|Q6FG59_HUMAN				**0,81**<br>0,69	0,98<br>0,82	**0,86**<br>0,70			
CDGSH iron sulfur domain-containing protein 1	Q9NZ45|CISD1_HUMAN	0,98	0,92							
CDGSH iron sulfur domain-containing protein 2	Q8N5K1|CISD2_HUMAN	0,97<br>1,03	0,99<br>**1,14**	1,03				1,13<br>1,02	**3,81**<br>2,63	**4,82**<br>4,19
CDK5 regulatory subunit-associated protein 3	Q96JB5|CK5P3_HUMAN	**0,72**	1,04	**1,47**				1,28	**9,63**	**8,70**
CDKN2A-interacting protein	Q9NXV6|CARF_HUMAN							0,72	0,07	0,03
cDNA FLJ11160 fis, clone PLACE1007014, weakly similar to 36 KD NUCLEOLAR PROTEIN HNP36	Q9NUS9|Q9NUS9_HUMAN	0,89	**0,53**							
cDNA FLJ13286 fis, clone OVARC1001154, highly similar to Homo sapiens clone 24720 epithelin 1 and 2 mRNA	Q9H8S1|Q9H8S1_HUMAN							0,00	0,00	0,00
cDNA FLJ14249 fis, clone OVARC1001200, weakly similar to Mus musculus HS1 binding protein 3	Q9H7U7|Q9H7U7_HUMAN				0,86	0,70	0,47			
cDNA FLJ16759 fis, clone BRACE3046450, highly similar to Interleukin-16	Q6ZMQ7|Q6ZMQ7_HUMAN				**0,00**	**0,00**	**0,00**	1,04	**0,42**	**0,35**
CDNA FLJ25678 fis, clone TST04067, highly similar to PURINE NUCLEOSIDE PHOSPHORYLASE	Q8N7G1|Q8N7G1_HUMAN				0,69<br>0,84	1,01<br>0,90	1,12<br>0,85			
CDNA FLJ26554 fis, clone LNF01773, highly similar to Galactokinase	Q6ZP37|Q6ZP37_HUMAN	0,88	0,99	1,93	**0,63**<br>1,08	0,74<br>1,27	**0,45**<br>0,94			
cDNA FLJ31051 fis, clone HSYRA2000605, weakly similar to MYOSIN HEAVY CHAIN, CLONE 203	Q96ND2|Q96ND2_HUMAN	1,02<br>1,02	1,14<br>1,15	1,18				1,07<br>1,30	**2,32**<br>1,76	**4,45**<br>**2,86**
cDNA FLJ31747 fis, clone NT2RI2007377, highly similar to RNA-BINDING PROTEIN EWS	Q96MX4|Q96MX4_HUMAN				2,27<br>1,03	8,53<br>1,11	17,85<br>0,87	1,26<br>1,14	0,69<br>0,94	0,65<br>1,23
cDNA FLJ34327 fis, clone FEBRA2009022, highly similar to TRANSLATION INITIATION FACTOR EIF-2B EPSILON	Q541Z1|Q541Z1_HUMAN				0,83	0,89	0,85			
cDNA FLJ34453 fis, clone HLUNG2002429, highly similar to Homo sapiens cartilage-associated protein	Q8NB01|Q8NB01_HUMAN	0,54<br>1,02	0,99<br>1,23	0,65						
cDNA FLJ34850 fis, clone NT2NE2011758, highly similar to NADH-UBIQUINONE OXIDOREDUCTASE 20 kDa SUBUNIT								0,00	0,00	0,00
cDNA FLJ35689 fis, clone SPLEN2019379, highly similar to INTERFERON-REGULATED RESISTANCE GTP-BINDING	Q8NAA8|Q8NAA8_HUMAN	1,76	**4,84**	**11,52**						
cDNA FLJ36887 fis, clone BNGH42005504, highly similar to 26S PROTEASOME REGULATORY SUBUNIT S3	Q8N9M2|Q8N9M2_HUMAN				0,62<br>1,02	0,83<br>1,25	0,64<br>1,02	0,00	0,00	0,00
cDNA FLJ37649 fis, clone BRHIP2000534, moderately similar to GAMMA-INTERFERON-INDUCIBLE PROTEIN IFI-16	Q8N9E5|Q8N9E5_HUMAN							0,00	0,00	0,00
cDNA FLJ38696 fis, clone KIDNE2001931, highly similar to HETEROGENEOUS NUCLEAR RIBONUCLEOPROTEIN G	Q8N8Y7|Q8N8Y7_HUMAN				1,74<br>1,18	**4,75**<br>2,48	**3,00**<br>1,68			
cDNA FLJ43203 fis, clone FEBRA2008468, highly similar to LYSOSOMAL ACID LIPASE/CHOLESTERYL ESTER	Q6ZUY8|Q6ZUY8_HUMAN	**0,64**<br>0,79	**0,53**<br>0,83	0,60						
cDNA FLJ44592 fis, clone BLADE2002310, highly similar to Homo sapiens SH3-domain binding protein 1 (Fragment)	Q6ZTJ5|Q6ZTJ5_HUMAN							0,79	0,73	**0,85**
cDNA FLJ45505 fis, clone BRTHA2020642, weakly similar to DRR1 protein	Q6ZSI4|Q6ZSI4_HUMAN				1,43	1,88	1,92			
cDNA FLJ46155 fis, clone TESTI4001517, moderately similar to Keratin, type I cytoskeletal 18	Q6ZRR8|Q6ZRR8_HUMAN	0,70	0,94	3,38				**31,81**		**64,02**
cDNA FLJ46672 fis, clone TRACH3009008, highly similar to Thioredoxin reductase	Q6ZR44|Q6ZR44_HUMAN				**0,79**<br>1,10	1,09<br>1,27	0,95<br>1,33			
cDNA FLJ75087, highly similar to Homo sapiens sarcosine dehydrogenase (SARDH), mRNA	A8K596|A8K596_HUMAN	1,06	1,01							
cDNA FLJ75299, highly similar to Xenopus laevis proteasome (prosome, macropain) 26S subunit, ATPase 3, mRNA	A8K781|A8K781_HUMAN			9999,00						
cDNA FLJ75460, highly similar to Homo sapiens phenylalanine-tRNA synthetase-like, beta subunit, mRNA	A8K666|A8K666_HUMAN				0,92	1,01	1,19			
cDNA FLJ75516, highly similar to Xenopus tropicalis ubiquitin C, mRNA	A8K674|A8K674_HUMAN	**0,93**	**1,09**							
cDNA FLJ75700, highly similar to Homo sapiens complement component 1, q subcomponent binding protein (C1QBP),	A8K651|A8K651_HUMAN	0,94	0,65	0,70						
cDNA FLJ75713, highly similar to Homo sapiens kynurenine 3-monooxygenase	A8K693|A8K693_HUMAN	1,01<br>**1,15**	0,93<br>1,10	0,93						
cDNA FLJ75774, highly similar to Homo sapiens lectin, mannose-binding 2 (LMAN2), mRNA	A8K7T4|A8K7T4_HUMAN	1,00	**1,11**							
cDNA FLJ75881, highly similar to Homo sapiens transferrin receptor	A8K6Q8|A8K6Q8_HUMAN	1,10<br>0,97	0,68<br>**0,94**	0,62	1,36	1,09	1,30			
cDNA FLJ75886, highly similar to Homo sapiens acyl-Coenzyme A oxidase 1, palmitoyl (ACOX1), transcript variant	A8K6X8|A8K6X8_HUMAN	1,00	0,90	**0,79**						
cDNA FLJ75945, highly similar to Homo sapiens heterogeneous nuclear ribonucleoprotein A2/B1 (HNRPA2B1),	A8K064|A8K064_HUMAN							0,00	0,00	0,00

cDNA FLJ76028, highly similar to Homo sapiens regulator of G-protein signalling 10 (RGS10), transcript variant 1, mRNA	A8K408|A8K408_HUMAN				0,50	0,61	0,56			
cDNA FLJ76053, highly similar to Homo sapiens Ras-GTPase activating protein SH3 domain-binding protein 2 (G3BP2),	A8K6V7|A8K6V7_HUMAN							0,63	0,34	**0,29**
cDNA FLJ76079, highly similar to Homo sapiens lymphocyte-specific protein 1 (LSP1), mRNA	A8K2L4|A8K2L4_HUMAN							**1,54**	**0,77**	**0,51**
cDNA FLJ76144, highly similar to Homo sapiens NADH dehydrogenase (ubiquinone) 1 alpha subcomplex, 10, 42kDa	A8K413|A8K413_HUMAN	0,95	0,93	**0,71**						
cDNA FLJ76156, highly similar to Homo sapiens aspartyl-tRNA synthetase 2 (DARS2), mRNA	A8K4A8|A8K4A8_HUMAN	0,99<br>1,03	0,93<br>0,91	**0,86**						
cDNA FLJ76183	A8K514|A8K514_HUMAN									9999,00
cDNA FLJ76254, highly similar to Homo sapiens gamma-glutamyl hydrolase (GGH), mRNA	A8K335|A8K335_HUMAN	1,02	0,99							
cDNA FLJ76270, highly similar to Homo sapiens platelet/endothelial cell adhesion molecule	A8K3S7|A8K3S7_HUMAN	1,10	0,97							
cDNA FLJ76298, highly similar to Homo sapiens serpin peptidase inhibitor, clade B (ovalbumin), member 1	A8K5L2|A8K5L2_HUMAN				0,98	0,96	**0,84**			
cDNA FLJ76462, highly similar to Homo sapiens heterogeneous nuclear ribonucleoprotein D	A8K9J2|A8K9J2_HUMAN							**1,15**	**0,54**	**0,56**
cDNA FLJ76490, highly similar to Homo sapiens ancient ubiquitous protein 1 (AUP1), transcript variant 2, mRNA	A8KAQ6|A8KAQ6_HUMAN	1,01	1,08							
cDNA FLJ76536, highly similar to Homo sapiens TYRO protein tyrosine kinase binding protein (TYROBP), transcript variant 2,	A8K2X0|A8K2X0_HUMAN	1,03	1,05	0,93						
cDNA FLJ76608, highly similar to Homo sapiens transmembrane protein 85, mRNA	A8K3A9|A8K3A9_HUMAN							0,00	0,00	0,00
cDNA FLJ76697	A8K0M6|A8K0M6_HUMAN	0,82	0,81							
cDNA FLJ76812, highly similar to Homo sapiens IQ motif containing GTPase activating protein 2 (IQGAP2), mRNA	A8K4V1|A8K4V1_HUMAN				1,24	1,00	1,02			
cDNA FLJ76817, highly similar to Homo sapiens non-POU domain containing, octamer-binding (NONO), mRNA	A8K525|A8K525_HUMAN	0,63	0,99	2,64	1,18	2,15	1,42	0,94<br>1,01	**0,23**<br>**0,37**	**0,12**<br>**0,31**
cDNA FLJ76820	A8K544|A8K544_HUMAN	0,99	1,00	0,64						
cDNA FLJ76859, highly similar to Homo sapiens splicing factor, arginine/serine-rich 4 (SFRS4), mRNA	A8K644|A8K644_HUMAN				2,37	**2,36**	1,83	**1,17**	**0,69**	**0,66**
cDNA FLJ76867, highly similar to Homo sapiens mannosidase, alpha, class 2B, member 1 (MAN2B1), mRNA	A8K6A7|A8K6A7_HUMAN				**1,68**	1,06	**1,78**			
cDNA FLJ77100	A8K4E9|A8K4E9_HUMAN	1,03	1,01							
cDNA FLJ77138, highly similar to Homo sapiens golgi SNAP receptor complex member 1 (GOSR1), transcript variant 1,	A8K5R6|A8K5R6_HUMAN	1,21	0,82	0,89						
cDNA FLJ77144, highly similar to Homo sapiens mitochondrial ribosome recycling factor (MRRF), transcript variant 1, mRNA	A8K6D8|A8K6D8_HUMAN	0,00	0,00	0,00						
cDNA FLJ77173, highly similar to Homo sapiens carboxylesterase 1	A8K844|A8K844_HUMAN	**0,93**	**1,15**							
cDNA FLJ77183	A8K8K3|A8K8K3_HUMAN	1,08	0,88							
cDNA FLJ77222, highly similar to Homo sapiens UDP-N-acetyl-alpha-D-galactosamine:polypeptide N-	A8KAJ7|A8KAJ7_HUMAN	1,06	0,88							
cDNA FLJ77226, highly similar to Homo sapiens p21 activated kinase 1B (PAK1B) mRNA	A8KAN3|A8KAN3_HUMAN				0,41	0,49	0,62			
cDNA FLJ77282, highly similar to Homo sapiens SEH1-like (SEH1L), mRNA	A8K5B1|A8K5B1_HUMAN							0,00	0,00	0,00
cDNA FLJ77404, highly similar to Homo sapiens small nuclear ribonucleoprotein 70kDa polypeptide	A8KAQ5|A8KAQ5_HUMAN							1,07	**0,34**	**0,30**
cDNA FLJ77459, highly similar to Homo sapiens interleukin enhancer binding factor 3, 90kDa, mRNA	A8K6F2|A8K6F2_HUMAN							0,97<br>**0,43**	1,02<br>**0,69**	0,99<br>0,59
cDNA FLJ77470, highly similar to Homo sapiens egf-like module containing, mucin-like, hormone receptor-like 2	A8K6W1|A8K6W1_HUMAN	0,00<br>0,95	0,00<br>1,00	0,00						
cDNA FLJ77498, highly similar to Homo sapiens nucleobindin 2 (NUCB2), mRNA	A8K642|A8K642_HUMAN	1,18	1,03							
cDNA FLJ77562, highly similar to Homo sapiens STEAP family member 3 (STEAP3), transcript variant 1, mRNA	A8K6E3|A8K6E3_HUMAN	0,88	0,91							
cDNA FLJ77574, highly similar to Homo sapiens iduronidase, alpha-L-(IDUA), mRNA	A8K701|A8K701_HUMAN	0,91	**0,86**							
cDNA FLJ77615, highly similar to Homo sapiens nucleolar complex associated 3 homolog	A8K905|A8K905_HUMAN							0,90	0,59	0,55
cDNA FLJ77650	A8K1I1|A8K1I1_HUMAN	1,23	1,05							
cDNA FLJ77654, highly similar to Homo sapiens guanine nucleotide binding protein (G protein), beta polypeptide 4	A8K3F6|A8K3F6_HUMAN	1,13	1,45							
cDNA FLJ77670, highly similar to Homo sapiens ribosomal protein S15a (RPS15A), mRNA	A8K7H3|A8K7H3_HUMAN	0,87	**1,26**							
cDNA FLJ77708, highly similar to Homo sapiens coatomer protein complex, subunit gamma (COPG), mRNA	A8K6M8|A8K6M8_HUMAN				0,85	1,04	1,10			
cDNA FLJ77725	A8KAH5|A8KAH5_HUMAN				0,72	0,60	0,78			
cDNA FLJ77757	A8K968|A8K968_HUMAN	**2,09**	**1,74**							
cDNA FLJ77763, highly similar to Homo sapiens prolactin regulatory element binding (PREB), mRNA	A8K813|A8K813_HUMAN	1,77	1,52	1,36						
cDNA FLJ78010, highly similar to Homo sapiens methylmalonyl Coenzyme A mutase, mRNA	A8K953|A8K953_HUMAN	0,00	0,00	0,00						
cDNA FLJ78048, highly similar to Homo sapiens torsin A interacting protein 1, mRNA	A8K630|A8K630_HUMAN							**1,16**	**0,68**	0,77
cDNA FLJ78144	A8K7K8|A8K7K8_HUMAN				0,37	0,55	0,44			
cDNA FLJ78173, highly similar to Homo sapiens hexokinase 1 (HK1) mRNA	A8K7J7|A8K7J7_HUMAN	**1,16**	**0,92**							
cDNA FLJ78212, highly similar to Human prolidase (imidodipeptidase) mRNA	A8K416|A8K416_HUMAN				0,98	0,93	1,02			
cDNA FLJ78242, highly similar to Homo sapiens signal sequence receptor, alpha (translocon-associated protein	A8K685|A8K685_HUMAN							1,50	**3,20**	**4,61**
cDNA FLJ78246, highly similar to Homo sapiens splicing factor 3a, subunit 3, 60kDa (SF3A3), mRNA	A8K566|A8K566_HUMAN							0,91<br>**0,73**	**0,31**<br>**0,41**	**0,25**<br>**0,30**
cDNA FLJ78303, highly similar to Homo sapiens CECR1 protein mRNA	A8K9H4|A8K9H4_HUMAN	0,00	0,00	0,00						
cDNA FLJ78321	A8K0V6|A8K0V6_HUMAN	**1,15**	1,00	0,94						
cDNA FLJ78356, highly similar to Homo sapiens PIMT isozyme I	A8K109|A8K109_HUMAN	0,00	0,00	0,00	0,98<br>**0,79**	1,08<br>0,88	1,03<br>**0,82**			
cDNA FLJ78366, highly similar to Homo sapiens leucine-rich PPR-motif containing (LRPPRC), mRNA	A8K1V1|A8K1V1_HUMAN	**0,93**<br>**0,94**	**0,93**<br>0,97	**0,74**				0,00	0,00	0,00
cDNA FLJ78417, highly similar to Homo sapiens low density lipoprotein receptor-related protein associated protein 1	A8K8F6|A8K8F6_HUMAN				1,36	0,89	1,35			
cDNA FLJ78433, highly similar to Homo sapiens chaperonin containing TCP1, subunit 5	A8K2X8|A8K2X8_HUMAN				**0,70**<br>**0,73**	0,99<br>0,92	1,09<br>0,99		8,60	18,84
cDNA FLJ78475, highly similar to Homo sapiens glycerol-3-phosphate dehydrogenase 2	A8K1Z2|A8K1Z2_HUMAN	**0,89**	**0,89**	**0,76**						
cDNA FLJ78481, highly similar to Homo sapiens adaptor-related protein complex 2, beta 1 subunit, mRNA	A8K916|A8K916_HUMAN	1,07<br>1,14	**0,69**<br>1,06	**0,73**						
cDNA FLJ78516	A8K2W3|A8K2W3_HUMAN				0,00	0,00	0,00			
cDNA FLJ78520, highly similar to Homo sapiens histone 1, H2bn (HIST1H2BN), mRNA	A8K9J7|A8K9J7_HUMAN							0,96<br>1,15	0,69<br>0,89	0,71<br>1,04

cDNA FLJ78528, highly similar to Homo sapiens vacuolar protein sorting 4B	A8K4G7|A8K4G7_HUMAN				**0,56**	**0,59**	**0,36**			
cDNA FLJ78533, highly similar to Homo sapiens dynamin 2 (DNM2), transcript variant 4, mRNA	A8K1B6|A8K1B6_HUMAN				1,03	**1,17**	0,93			
cDNA FLJ78591, highly similar to Homo sapiens ribosomal protein S3A (RPS3A), mRNA	A8K4W0|A8K4W0_HUMAN	1,10	1,58							
cDNA FLJ78601, highly similar to Homo sapiens glycoprotein (transmembrane) nmb (GPNMB), transcript variant 2, mRNA	A8K7R3|A8K7R3_HUMAN	1,07	0,98	**0,80**				**1,45**	**1,55**	**1,72**
cDNA FLJ78614, highly similar to Homo sapiens eukaryotic translation initiation factor 4A, isoform 1 (EIF4A1), mRNA	A8K088|A8K088_HUMAN	1,02	1,66							
cDNA FLJ78639	A8K6Q6|A8K6Q6_HUMAN	0,97	0,87							
cDNA FLJ78645, highly similar to Homo sapiens DEAH (Asp-Glu-Ala-His) box polypeptide 30 (DHX30), transcript variant 2,	A8K5F1|A8K5F1_HUMAN	0,97	0,88	0,94						
cDNA FLJ78673, highly similar to Homo sapiens NOD9 protein (NOD9), transcript variant 1, mRNA	A8K6Q1|A8K6Q1_HUMAN	**1,20**	0,99							
cDNA FLJ78677, highly similar to Homo sapiens splicing factor 3b, subunit 3, 130kDa (SF3B3), mRNA	A8K6V3|A8K6V3_HUMAN							**1,44**<br>**0,31**	0,51<br>**0,44**	0,79<br>**0,42**
cDNA FLJ78679, highly similar to Homo sapiens DEAD (Asp-Glu-Ala-Asp) box polypeptide 46 (DDX46), mRNA	A8K6X3|A8K6X3_HUMAN							1,07	**0,44**	**0,38**
cDNA FLJ90197 fis, clone MAMMA1001418	Q8NCK4|Q8NCK4_HUMAN	1,10	0,78	0,84						
cDNA FLJ90278 fis, clone NT2RP1000325, highly similar to Phosphate carrier protein, mitochondrialprecursor	Q8NCF7|Q8NCF7_HUMAN	**0,94**<br>0,94	**0,91**<br>0,95	**0,78**	2,50	**1,51**	1,37	**1,37**<br>0,71	**3,70**<br>**2,53**	**6,22**<br>**2,77**
cDNA FLJ90803 fis, clone Y79AA1000270, highly similar to Vacuolar ATP synthase subunit S1	Q8NBT4|Q8NBT4_HUMAN	0,98<br>**0,87**	0,75<br>0,87	0,76						
cDNA, FLJ79483, highly similar to Homo sapiens guanylate binding protein family, member 6 (GBP6), mRNA	B0AZP3|B0AZP3_HUMAN							0,50	0,13	0,16
CDNA: FLJ20897 fis, clone ADKA03573	Q9H7G6|Q9H7G6_HUMAN							0,87<br>1,21	**1,70**<br>1,54	**1,62**<br>**1,56**
CDNA: FLJ22728 fis, clone HSI15617, (Fragment)	Q9H600|Q9H600_HUMAN							0,00	0,00	0,00
CDP-diacylglycerol--inositol 3-phosphatidyltransferase isoform 1 variant (Fragment)	Q53HA5|Q53HA5_HUMAN	0,98<br>**0,73**	1,30<br>0,81	1,38						
Cell cycle control protein 50A	Q9NV96|CC50A_HUMAN	1,06	0,70	0,62						
Cell division control protein 42 homolog precursor	Q6ICS0|Q6ICS0_HUMAN	1,02<br>**1,24**	1,00<br>**1,28**	1,01	0,84<br>1,13	0,80<br>1,06	0,72<br>0,92	0,98<br>**0,45**	0,92<br>0,76	**2,01**<br>0,95
Cell division cycle 5-like protein	Q99459|CDC5L_HUMAN							**0,73**<br>0,97	**0,28**<br>**0,50**	**0,17**<br>**0,43**
Cell division cycle and apoptosis regulator protein 1	Q8IX12|CCAR1_HUMAN							1,00<br>0,89	0,66<br>0,89	0,68<br>0,87
Cell growth-inhibiting protein 15	Q08ES5|Q08ES5_HUMAN				1,12	0,98	0,65	1,18<br>**0,46**	1,12<br>**0,57**	1,33<br>0,89
Cell growth-inhibiting protein 34	Q08ES8|Q08ES8_HUMAN	**0,84**<br>**1,23**	**1,20**<br>**1,26**	**1,89**						
Cell growth-regulating nucleolar protein	Q9NX58|LYAR_HUMAN							**0,80**<br>0,84	**0,49**<br>**0,61**	**0,49**<br>**0,46**
Cell proliferation-inducing protein 47	Q08ET0|Q08ET0_HUMAN	**0,84**<br>0,92	**0,81**<br>0,99	**0,77**						
Cell surface glycoprotein	Q29971|Q29971_HUMAN				**1,86**	0,86	1,44			
Cellular retinoic acid-binding protein 2	P29373|RABP2_HUMAN				0,91<br>**0,76**	**1,17**<br>1,11	1,12<br>1,23			
Centrin-2	P41208|CETN2_HUMAN							0,90	**0,36**	**0,22**
Centrosomal protein 170kDa	A6H8X9|A6H8X9_HUMAN				0,00	0,00	0,00			
Centrosomal protein of 170 kDa	Q5SW79|CE170_HUMAN				1,40	0,94	0,93			
Cerebral protein 1	O43159|HUCE1_HUMAN							0,00	0,00	0,00
Ceroid-lipofuscinosis neuronal protein 5	O75503|CLN5_HUMAN	0,88	1,19	1,22						
Cervical EMMPRIN	Q54A51|Q54A51_HUMAN	**1,39**<br>1,02	1,00<br>**1,08**	1,05	**1,80**<br>**1,43**	1,08<br>0,90	**1,52**<br>1,23			
CES2 protein (Fragment)	Q6IPK9|Q6IPK9_HUMAN							0,90	**0,58**	0,80
CGG triplet repeat-binding protein 1	Q9UFW8|CGBP1_HUMAN							1,05	0,30	**0,27**
Chaperone activity of bc1 complex-like, mitochondrial precursor	Q8NI60|ADCK3_HUMAN	**1,25**	1,00							
Chaperonin containing TCP1, subunit 3	Q5SZY0|Q5SZY0_HUMAN	0,86	1,08	**2,10**	**0,84**<br>0,83	0,98<br>1,09	1,07<br>1,24			
Chaperonin containing TCP1, subunit 6A isoform a variant (Fragment)	Q59ET3|Q59ET3_HUMAN	1,25	**1,51**	3,99	0,64	0,89	0,89			
Charged multivesicular body protein 1a	Q9HD42|CHM1A_HUMAN							**1,27**<br>1,05	**2,41**<br>**2,00**	**2,25**<br>**2,63**
Charged multivesicular body protein 1b	Q7LBR1|CHM1B_HUMAN							1,02	1,26	0,97
Charged multivesicular body protein 2a	O43633|CHM2A_HUMAN				1,06	0,69	**0,62**	**1,25**<br>1,64	**1,71**<br>**1,86**	**1,42**<br>1,77
Charged multivesicular body protein 2b	Q9UQN3|CHM2B_HUMAN				0,88	0,82	0,93			
Charged multivesicular body protein 4b	Q9H444|CHM4B_HUMAN	0,00<br>0,76	0,00<br>0,94	0,00	0,92<br>0,65	**0,75**<br>**0,66**	0,80<br>0,60	**1,64**<br>0,87	**3,72**<br>2,91	**3,25**<br>3,65
Charged multivesicular body protein 5	Q9NZZ3|CHMP5_HUMAN				0,91	**0,71**	**0,67**	1,37	**2,22**	1,64
Chitinase domain-containing protein 1 precursor	Q9BWS9|CHID1_HUMAN	**0,82**<br>0,92	**0,83**<br>1,00	**0,74**				0,99<br>0,97	**1,49**<br>**1,46**	**1,84**<br>**1,97**
Chitinase-3-like protein 1 precursor	P36222|CH3L1_HUMAN	0,68<br>0,83	**0,21**<br>**0,74**	**0,19**				**0,80**	**0,35**	**0,49**
Chitobiase, di-N-acetyl-	Q5VX51|Q5VX51_HUMAN	0,88<br>1,02	**1,46**<br>0,90	1,11						
Chitobiosyldiphosphodolichol beta-mannosyltransferase	Q9BT22|ALG1_HUMAN	1,11<br>1,01	**1,36**<br>1,05	1,24						
Chloride channel CLIC-like protein 1 precursor	Q96S66|CLCC1_HUMAN	1,01	1,02	1,14						
Chloride channel protein 7	P51798|CLCN7_HUMAN	1,01<br>**0,85**	1,07<br>0,90	0,88						
Chloride intracellular channel 1 protein	O00299|CLIC1_HUMAN	1,17	1,26		0,84<br>0,85	**0,77**<br>**0,80**	0,68<br>**0,69**			
Chloride intracellular channel protein 4	Q9Y696|CLIC4_HUMAN	1,21	0,99		**0,77**<br>0,89	0,91<br>0,84	0,79<br>**0,72**			
Choline/ethanolaminephosphotransferase 1	Q9Y6K0|CEPT1_HUMAN	0,00<br>0,87	0,00<br>1,24	0,00						
Chondrosarcoma-associated protein 2	Q9UK43|Q9UK43_HUMAN							**0,78**<br>0,86	**0,30**<br>**0,52**	**0,18**<br>**0,35**
Chromatin modifying protein 4A	Q14D22|Q14D22_HUMAN				0,42	0,49	0,45			
Chromobox protein homolog 3	Q13185|CBX3_HUMAN				**1,45**<br>0,58	1,59<br>0,97	1,19<br>1,15	0,81<br>0,80	**0,31**<br>**0,59**	**0,20**<br>**0,48**
Chromodomain-helicase-DNA-binding protein 4	Q14839|CHD4_HUMAN							1,02<br>**0,60**	0,71<br>**0,47**	0,66<br>**0,47**
Cisplatin resistance-associated overexpressed protein	O95232|CROP_HUMAN							**0,88**<br>1,17	**0,32**<br>**0,65**	**0,23**<br>**0,65**
Citrate lyase subunit beta-like protein, mitochondrial precursor	Q8N0X4|CLYBL_HUMAN	0,81<br>1,03	0,89<br>0,94	0,79						
Citrate synthase, mitochondrial precursor	O75390|CISY_HUMAN	**0,83**	**0,83**	**0,61**	**1,78**	**1,57**	**2,58**	1,08	**2,66**	**3,70**

		1,04	1,02		1,79	1,36	1,74	**0,76**	**2,10**	**2,47**
C-jun-amino-terminal kinase-interacting protein 4	O60271|JIP4_HUMAN				1,54	1,02	1,88			
CKLF-like MARVEL transmembrane domain-containing protein 3	Q96MX0|CKLF3_HUMAN	1,22<br>0,90	0,79<br>1,01	1,19						
CKLF-like MARVEL transmembrane domain-containing protein 6	Q9NX76|CKLF6_HUMAN	1,62<br>**1,26**	1,35<br>0,94							
Clathrin heavy chain 1	Q00610|CLH1_HUMAN	**1,22**<br>**1,66**	**1,56**<br>**1,85**	**2,45**	**1,27**<br>1,44	**1,68**<br>1,77	**1,87**<br>1,98	**1,28**<br>**0,43**	1,10<br>1,05	**2,26**<br>**1,32**
Clathrin light chain A	P09496|CLCA_HUMAN	1,08	1,53	3,15	1,20<br>**0,80**	1,14<br>1,08	1,44<br>**1,30**	1,01<br>**1,44**	**1,36**<br>**1,68**	**1,55**<br>**1,93**
Clathrin, light polypeptide	Q53Y37|Q53Y37_HUMAN				1,46<br>0,91	**1,37**<br>1,03	**1,98**<br>1,25	1,02<br>**1,38**	**1,23**<br>**1,81**	**1,33**<br>**1,91**
Cleavage and polyadenylation specificity factor subunit 5	O43809|CPSF5_HUMAN				**1,36**	2,38	2,01	**1,24**<br>**1,27**	**0,78**<br>0,88	**0,48**<br>**0,79**
Cleavage and polyadenylation specificity factor subunit 6	Q16630|CPSF6_HUMAN				0,00	0,00	0,00	1,06<br>**1,49**	**0,77**<br>**1,95**	**0,48**<br>**1,61**
Cleavage and polyadenylation specificity factor subunit 7	Q8N684|CPSF7_HUMAN				0,00	0,00	0,00	0,74<br>1,04	**0,31**<br>0,70	**0,21**<br>0,47
Cleavage stimulation factor 64 kDa subunit	P33240|CSTF2_HUMAN				0,00	0,00	0,00	1,02<br>1,40	**0,27**<br>0,75	**0,17**<br>**0,36**
Cleft lip and palate transmembrane protein 1	O96005|CLPT1_HUMAN	0,98<br>0,87	0,94<br>1,03	1,05	0,00<br>0,00	0,00<br>0,00	0,00<br>0,00	0,00	0,00	0,00
CMRF35-like molecule 1 precursor	Q8TDQ1|CLM1_HUMAN	0,00	0,00							
C-Myc-binding protein	Q99417|MYCBP_HUMAN							0,81<br>1,42	**2,01**<br>5,18	**1,75**<br>5,30
Coactosin-like protein	Q14019|COTL1_HUMAN	0,00<br>**2,25**	0,00<br>**1,94**	0,00	0,92<br>**0,86**	1,03<br>0,98	0,98<br>1,02	1,15	**0,32**	**0,26**
Coatomer subunit beta	P53618|COPB_HUMAN	1,39	1,91	**4,86**	0,72<br>0,63	0,98<br>0,96	0,95<br>1,17			
Coatomer subunit gamma	Q9Y678|COPG_HUMAN				**0,66**	**0,92**	0,90			
Cob(I)yrinic acid a,c-diamide adenosyltransferase, mitochondrial precursor	Q96EY8|MMAB_HUMAN	0,93<br>1,11	0,89<br>0,96	**0,62**	8,19<br>2,05	3,93<br>1,42	6,13<br>1,98	1,88	**2,80**	**3,76**
Co-chaperone protein HscB, mitochondrial precursor	Q8IWL3|HSC20_HUMAN	1,03	0,95							
Coenzyme Q3 homolog, methyltransferase	Q5T061|Q5T061_HUMAN	0,00	1,23							
Coenzyme Q7 homolog, ubiquinone	Q9BTT7|Q9BTT7_HUMAN	0,92	**0,80**							
Cofilin-1	P23528|COF1_HUMAN	**1,88**<br>**2,11**	**1,63**<br>**1,64**	**2,18**	**0,85**<br>0,68	1,02<br>0,82	0,99<br>0,75	0,94<br>**1,45**	**0,66**<br>**0,87**	0,90<br>**0,78**
Cohesin subunit SA-1	Q8WVM7|STAG1_HUMAN							0,81	1,49	1,38
Coiled-coil domain containing 6	Q6GSG7|Q6GSG7_HUMAN				0,94	0,78	0,72			
Coiled-coil domain-containing protein 109A	Q8NE86|C109A_HUMAN	0,89<br>0,92	0,93<br>0,98	0,80						
Coiled-coil domain-containing protein 109B	Q9NWR8|C109B_HUMAN	1,03	0,83							
Coiled-coil domain-containing protein 115	Q96NT0|CC115_HUMAN	1,14	1,13	0,99						
Coiled-coil domain-containing protein 124	Q96CT7|CC124_HUMAN				0,99<br>**0,60**	0,69<br>**0,68**	0,53<br>**0,53**	**1,08**<br>1,18	0,94<br>1,25	0,55<br>0,91
Coiled-coil domain-containing protein 44	Q9BSH4|CCD44_HUMAN	1,03	1,04							
Coiled-coil domain-containing protein 47 precursor	Q96A33|CCD47_HUMAN	0,93<br>**0,75**	1,22<br>0,99	1,53				0,83<br>1,03	**2,39**<br>2,01	**3,16**<br>**2,48**
Coiled-coil domain-containing protein 51	Q96ER9|CCD51_HUMAN	0,94<br>0,98	0,92<br>**0,86**	**0,69**						
Coiled-coil domain-containing protein 56	Q9Y2R0|CCD56_HUMAN	1,00<br>0,93	**1,08**<br>0,99	0,71				0,94<br>1,83	1,04<br>2,53	1,05<br>3,92
Coiled-coil domain-containing protein 58	Q4VC31|CCD58_HUMAN	0,00<br>0,87	0,00<br>**0,87**	0,00						
Coiled-coil domain-containing protein 72	Q9Y2S6|CCD72_HUMAN				0,82<br>**0,52**	0,82<br>0,79	0,86<br>0,79	**0,75**<br>0,86	**0,44**<br>**0,69**	**0,32**<br>**0,66**
Coiled-coil domain-containing protein 86	Q9H6F5|CCD86_HUMAN							**1,23**<br>1,27	0,82<br>1,22	**0,64**<br>1,46
Coiled-coil domain-containing protein 90A, mitochondrial precursor	Q96AQ8|CC90A_HUMAN	1,17	1,14							
Coiled-coil domain-containing protein 90B, mitochondrial precursor	Q9GZT6|CC90B_HUMAN	1,15<br>0,94	0,86<br>0,84	0,76						
Coiled-coil domain-containing protein 93	Q567U6|CCD93_HUMAN	0,84	0,89	1,22	0,00	0,00	0,00			
Coiled-coil-helix-coiled-coil-helix domain-containing protein 3, mitochondrial precursor	Q9NX63|CHCH3_HUMAN	**0,84**<br>1,01	**0,88**<br>**1,08**	**0,74**	**1,65**	0,99	0,94	0,99<br>**0,78**	1,00<br>1,17	1,01<br>**1,38**
Coiled-coil-helix-coiled-coil-helix domain-containing protein 5	Q9BSY4|CHCH5_HUMAN	0,00	0,00							
Coiled-coil-helix-coiled-coil-helix domain-containing protein 6	Q9BRQ6|CHCH6_HUMAN	0,91	1,14							
Coiled-coil-helix-coiled-coil-helix domain-containing protein 8	Q9NYJ1|CHCH8_HUMAN	0,00	0,00							
Cold inducible RNA binding protein	Q53XX5|Q53XX5_HUMAN							**1,39**<br>1,78	0,50<br>0,58	**0,32**<br>0,66
Collagen alpha-1(XI) chain precursor	P12107|COBA1_HUMAN	1,07	1,07							
Collagen type IV alpha-3-binding protein	Q9Y5P4|C43BP_HUMAN				0,95	0,88	0,65			
Collectin-12	Q5KU26|COL12_HUMAN	0,88	0,75							
Complement component 1 Q subcomponent-binding protein, mitochondrial precursor	Q07021|C1QBP_HUMAN	0,99	1,00					0,00	0,00	0,00
Complex I intermediate-associated protein 30, mitochondrial precursor	Q9Y375|CIA30_HUMAN	1,01<br>1,02	0,88<br>0,99	0,71						
COMT protein	Q9BWC7|Q9BWC7_HUMAN	0,71<br>0,96	1,58<br>1,09	1,33	**1,55**	0,93	0,90			
COP9 signalosome complex subunit 4	Q9BT78|CSN4_HUMAN				0,84	0,94	0,77			
Copine-2	Q96FN4|CPNE2_HUMAN				0,00	0,00	0,00			
Copine-3	O75131|CPNE3_HUMAN	1,69<br>1,23	1,24<br>1,07	1,54	1,56	1,30	1,52			
Copper transport protein ATOX1	O00244|ATOX1_HUMAN				0,00	0,00	0,00			
Coproporphyrinogen oxidase variant (Fragment)	Q53F08|Q53F08_HUMAN	**0,69**<br>0,92	**0,35**<br>**0,76**	**0,35**						
Core histone macro-H2A,1	O75367|H2AY_HUMAN	0,88<br>**0,51**	**0,38**<br>**0,64**	1,02	**2,01**	**3,81**	**6,46**	**0,88**<br>0,98	**0,55**<br>**0,54**	**0,64**<br>**0,88**
Coronin 7	Q17RK4|Q17RK4_HUMAN				1,05<br>1,48	0,93<br>0,94	0,81<br>1,06			
Coronin, actin binding protein, 1C variant (Fragment)	Q59EA2|Q59EA2_HUMAN				**1,51**<br>**1,61**	**1,64**<br>**1,68**	**1,83**<br>**1,99**	1,20	**0,60**	0,98
Coronin-1A	P31146|COR1A_HUMAN	0,00<br>1,40	0,00<br>1,16	0,00	1,04<br>1,07	**1,25**<br>1,49	**1,32**<br>**1,65**	**1,43**<br>**0,34**	0,80<br>**0,75**	1,13<br>**0,68**
Coronin-1B	Q9BR76|COR1B_HUMAN				0,96<br>1,19	1,18<br>**1,26**	1,03<br>**1,36**	**0,31**	**0,42**	**0,33**
Coronin-1C_i3 protein	A7MAP1|A7MAP1_HUMAN	**1,52**<br>**1,29**	1,02<br>0,94	**1,28**				**0,33**	**0,59**	**0,43**

Corticosteroid 11-beta-dehydrogenase isozyme 1	P28845|DHI1_HUMAN	0,92<br>0,84	**1,24**<br>0,96	1,20	1,24	1,16	0,96			
COX5B protein (Fragment)	Q6FHJ9|Q6FHJ9_HUMAN				1,69	1,02	0,98			
COX7A2L protein	Q6FGA0|Q6FGA0_HUMAN	0,90<br>0,90	**0,79**<br>0,90	**0,81**				1,12<br>0,00	0,93<br>0,00	**1,70**<br>0,00
Crk-like protein	P46109|CRKL_HUMAN				0,00	0,00	0,00			
CSDA protein	Q96B76|Q96B76_HUMAN				0,00	0,00	0,00			
CSTA protein	Q6IB90|Q6IB90_HUMAN				0,91	0,96	**0,78**			
CTAGE family, member 5 isoform 1 variant (Fragment)	Q59FD2|Q59FD2_HUMAN	0,95<br>1,04	1,19<br>1,05	1,21						
C-terminal binding protein 1	Q7Z2Q5|Q7Z2Q5_HUMAN				0,00	0,00	0,00	0,00	0,00	0,00
Cathepsin G precursor	P08311|CATG_HUMAN	0,98	1,19							
Cathepsin S precursor	P25774|CATS_HUMAN	1,00<br>0,97	**1,15**<br>**0,82**	**0,74**	**1,96**<br>2,09	1,05<br>1,11	**1,29**<br>**1,56**	0,99<br>**0,65**	0,60<br>1,45	0,68<br>**1,64**
CTTN protein	Q8N707|Q8N707_HUMAN							0,91	**0,23**	**0,23**
C-type lectin domain family 5 member A	Q9NY25|CLC5A_HUMAN	1,79<br>1,33	2,85<br>1,08	3,20	0,00	0,00	0,00			
Cullin-4B	Q13620|CUL4B_HUMAN				1,15	1,15	**1,36**			
Cullin-associated NEDD8-dissociated protein 1	Q86VP6|CAND1_HUMAN				0,97<br>0,91	1,05<br>0,89	**1,14**<br>0,99			
CYB5 protein	Q6IB14|Q6IB14_HUMAN	0,87	1,17	0,86						
Cystatin B	P04080|CYTB_HUMAN	1,01<br>1,10	1,43<br>**1,25**	1,66	0,92<br>**0,77**	1,01<br>**0,75**	0,94<br>1,10	0,95<br>2,17	**2,00**<br>1,90	**2,05**<br>1,84
Cystatin-C precursor	P01034|CYTC_HUMAN	1,08	0,89							
Cysteine and glycine-rich protein 1	Q5U0J2|Q5U0J2_HUMAN							1,12	**0,32**	**0,26**
Cysteine desulfurase, mitochondrial precursor	Q9Y697|NFS1_HUMAN	1,08	1,02							
Cysteine-rich protein 1	Q53XY7|Q53XY7_HUMAN				0,87	1,06	1,01			
Cytidine deaminase	P32320|CDD_HUMAN				0,88	0,97	0,85			
Cytochrome b-245 heavy chain	P04839|CY24B_HUMAN	**1,30**<br>1,04	1,05<br>**1,14**	1,10	**1,98**<br>2,55	**1,29**<br>**1,29**	**1,40**<br>**1,51**	**2,21**<br>**0,53**	**2,06**<br>**1,89**	**4,37**<br>**2,06**
Cytochrome b-245 light chain	P13498|CY24A_HUMAN	**1,23**<br>**1,12**	1,10<br>0,91	**1,41**	**1,79**<br>**1,96**	1,05<br>1,25	**1,28**<br>1,23	**2,38**<br>**1,19**	**3,14**<br>**1,77**	**3,40**<br>**1,93**
Cytochrome b-5 isoform 1 variant (Fragment)	Q59F44|Q59F44_HUMAN	1,06	1,10							
Cytochrome b5 type B precursor	O43169|CYB5B_HUMAN	1,05	1,21	0,94						
Cytochrome b561 domain-containing protein 2	O14569|C56D2_HUMAN	0,91	1,07							
Cytochrome b-c1 complex subunit 1, mitochondrial precursor	P31930|QCR1_HUMAN	0,89<br>**0,93**	**0,81**<br>**0,92**	**0,67**						
Cytochrome b-c1 complex subunit 2, mitochondrial precursor	P22695|QCR2_HUMAN	**0,85**<br>0,96	**0,86**<br>0,98	**0,84**	**1,81**	1,09	0,99	0,97<br>1,34	**0,75**<br>**0,67**	**1,49**<br>1,24
Cytochrome b-c1 complex subunit 6, mitochondrial precursor	P07919|QCR6_HUMAN	1,02<br>**1,17**	1,04<br>**1,16**	**0,80**						
Cytochrome b-c1 complex subunit 8	O14949|QCR8_HUMAN	0,99<br>0,94	0,91<br>**0,84**	**0,74**				1,04<br>**3,09**	**0,82**<br>0,71	**1,17**<br>**1,70**
Cytochrome b-c1 complex subunit 9	Q9UDW1|QCR9_HUMAN	**0,98**<br>0,97	0,95<br>0,95	**0,76**						
Cytochrome b-c1 complex subunit Rieske, mitochondrial precursor	P47985|UCRI_HUMAN	1,02<br>0,97	1,06<br>0,96	0,88				1,14	0,88	1,34
Cytochrome c	P99999|CYC_HUMAN	**0,74**<br>**0,79**	**0,68**<br>**0,88**	**0,30**	**1,98**<br>**2,23**	**2,45**<br>**2,43**	**2,46**<br>**2,44**	1,06<br>**1,65**	**0,54**<br>0,85	**0,41**<br>**0,68**
Cytochrome c oxidase assembly protein COX15 homolog	Q7KZN9|COX15_HUMAN	0,97<br>0,95	0,98<br>**0,87**	0,86						
Cytochrome c oxidase assembly protein COX19	Q49B96|COX19_HUMAN	1,34	0,82							
Cytochrome c oxidase copper chaperone	Q14061|COX17_HUMAN	1,04	0,90							
Cytochrome c oxidase polypeptide 8A, mitochondrial	P10176|COX82_HUMAN	**0,78**	0,95					1,12<br>**3,31**	1,11<br>1,62	1,47<br>2,14
Cytochrome c oxidase polypeptide VIa-liver, mitochondrial precursor	P12074|CX6A1_HUMAN	0,00	0,00							
Cytochrome c oxidase polypeptide VIc precursor	P09669|COX6C_HUMAN	1,01<br>**1,08**	0,92<br>**1,07**	**0,79**				**1,26**<br>**2,32**	1,03<br>0,97	**1,38**<br>**1,72**
Cytochrome c oxidase polypeptide VIIa-liver/heart, mitochondrial precursor	P14406|CX7A2_HUMAN							**2,24**	1,15	**1,65**
Cytochrome c oxidase subunit 2	Q4GQP0|Q4GQP0_HUMAN	**1,13**<br>0,98	1,01<br>0,99	**0,89**	1,70	1,00	0,89	**1,21**<br>**2,12**	0,97<br>0,86	**1,62**<br>**1,67**
Cytochrome c oxidase subunit 7C, mitochondrial precursor	P15954|COX7C_HUMAN	0,92<br>1,12	0,94<br>**1,14**	0,74				**1,42**<br>**2,09**	1,00<br>1,25	**1,55**<br>**2,28**
Cytochrome c oxidase subunit IV isoform 1	Q6P666|Q6P666_HUMAN	**0,87**<br>1,04	0,96<br>1,03	**0,82**	**2,16**	1,09	1,09	**1,29**<br>**2,54**	0,96<br>**1,46**	**1,61**<br>**2,32**
Cytochrome c oxidase subunit Va	Q8TB65|Q8TB65_HUMAN	0,98<br>0,99	**0,88**<br>1,05	**0,68**	0,00<br>**1,82**	0,00<br>1,09	0,00<br>1,17	**1,19**<br>**1,96**	0,90<br>1,23	**1,41**<br>**2,02**
Cytochrome c oxidase subunit Vb	Q53YB7|Q53YB7_HUMAN	**0,71**<br>0,98	**0,79**<br>0,99	**0,71**	**1,98**	0,98	0,95	1,25<br>**2,15**	1,01<br>1,21	**1,63**<br>**2,18**
Cytochrome c oxidase subunit VIb isoform 1	P14854|CX6B1_HUMAN	1,00<br>0,98	**0,91**<br>1,04	**0,77**	**1,72**<br>**1,99**	0,96<br>1,04	**0,84**<br>0,94	**1,25**<br>**2,30**	**0,66**<br>1,07	0,98<br>**1,64**
Cytochrome c oxidase subunit VIIa polypeptide 2	Q496I0|Q496I0_HUMAN	1,10<br>0,88	0,97<br>**0,79**	1,14				**1,43**	1,09	1,25
Cytochrome c-type heme lyase	P53701|CCHL_HUMAN	0,94	1,04							
Cytochrome c1, heme protein, mitochondrial precursor	P08574|CY1_HUMAN	0,90	0,86	0,87						
Cytochrome P450 20A1	Q6UW02|CP20A_HUMAN	1,00	1,08							
Cytochrome P450 27, mitochondrial precursor	Q02318|CP27A_HUMAN	**0,87**<br>0,98	**0,65**<br>**0,91**	**0,47**	0,00<br>3,12	0,00<br>0,96	0,00<br>0,81	1,45<br>0,68	**2,67**<br>**2,78**	**4,04**<br>**3,77**
Cytochrome P450 2S1	Q96SQ9|CP2S1_HUMAN	0,94<br>0,85	0,95<br>0,99	0,95						
Cytochrome P450 51A1	Q16850|CP51A_HUMAN	**0,81**	**0,75**	**0,74**						
Cytochrome P450, family 1, subfamily B, polypeptide 1	Q5TZW8|Q5TZW8_HUMAN	0,87<br>0,87	**0,78**<br>0,43	**0,63**						
Cytokine receptor-like factor 3	Q8IUI8|CRLF3_HUMAN				0,00	0,00	0,00			
Cytoplasmic dynein 1 heavy chain 1	Q14204|DYHC1_HUMAN	0,89	1,13	1,53	0,90	0,98<br>0,91	**0,80**<br>0,81	1,07	0,74	1,14
Cytoplasmic dynein intermediate chain 2C	Q7Z4X1|Q7Z4X1_HUMAN	0,00	0,00	0,00	0,97<br>0,87	0,93<br>1,14	0,85<br>0,93			
Cytoplasmic FMR1-interacting protein 1	Q7L576|CYFP1_HUMAN	0,86<br>1,14	1,05<br>**1,36**	1,38	1,13	1,17	1,16	0,81	0,98	0,92
Cytoskeleton-associated protein 4	Q07065|CKAP4_HUMAN	0,97<br>0,98	1,04<br>**1,14**	1,04	**1,85**<br>**1,61**	0,96<br>1,12	1,16<br>1,21	**0,72**<br>**1,21**	**2,68**<br>**2,76**	**3,30**<br>**3,84**
Cytoskeleton-associated protein 5	Q14008|CKAP5_HUMAN				0,96	1,02	1,16	0,93	**1,13**	1,11
Cytosol aminopeptidase	P28838|AMPL_HUMAN	1,02<br>1,01	1,02<br>1,07	**1,53**	0,97<br>0,90	1,14<br>1,07	**1,18**<br>0,98			

Cytosolic non-specific dipeptidase	Q96KP4|CNDP2_HUMAN				**0,81**<br>**0,90**	**0,84**<br>1,05	**0,85**<br>0,92			
DAP protein (Fragment)	Q6FGC3|Q6FGC3_HUMAN				0,00<br>0,00	0,00<br>0,00	0,00<br>0,00			
DAZ-associated protein 1	Q96EP5|DAZP1_HUMAN							1,34	1,49	0,61
D-beta-hydroxybutyrate dehydrogenase, mitochondrial precursor	Q02338|BDH_HUMAN	0,86<br>0,99	0,94<br>1,02	**0,73**						
DDX48 protein	Q6IBQ2|Q6IBQ2_HUMAN							0,92<br>**0,46**	0,26<br>**0,27**	**0,27**<br>**0,28**
DEAD (Asp-Glu-Ala-Asp) box polypeptide 17	B1AHM0|B1AHM0_HUMAN							0,93<br>0,67	**0,25**<br>**0,40**	**0,21**<br>**0,35**
DEAD/H (Asp-Glu-Ala-Asp/His) box polypeptide 3 variant (Fragment)	Q59GX6|Q59GX6_HUMAN	0,84	**1,46**	1,90	0,74<br>0,89	0,77<br>0,88	**0,55**<br>0,82	1,30<br>**0,65**	0,27<br>**0,58**	0,22<br>**0,45**
Death-inducer obliterator 1	Q9BTC0|DIDO1_HUMAN							0,82<br>1,21	0,45<br>**0,64**	0,44<br>0,67
Death-inducing protein	Q6IC98|DEAIN_HUMAN	 0,00	 0,00							
Dedicator of cytokinesis protein 2	Q92608|DOCK2_HUMAN	 1,05	 1,04		0,00	0,00	0,00			
Dedicator of cytokinesis protein 8	Q8NF50|DOCK8_HUMAN	0,91<br>**0,83**	0,97<br>0,99	**1,25**	1,04	1,43	1,33			
Dehydrogenase/reductase SDR family member 1	Q96LJ7|DHRS1_HUMAN	 **1,20**	 1,05							
Dehydrogenase/reductase SDR family member 4	Q9BTZ2|DHRS4_HUMAN	0,99<br>**0,90**	0,89<br>0,90	0,95						
Dehydrogenase/reductase SDR family member 7 precursor	Q9Y394|DHRS7_HUMAN	0,99<br>**0,89**	**1,22**<br>1,03	**1,23**						
Dehydrogenase/reductase SDR family member 7B	Q6IAN0|DRS7B_HUMAN	0,00<br>0,87	0,00<br>0,94	0,00						
Dehydrogenase/reductase SDR family member 9 precursor	Q9BPW9|DHRS9_HUMAN	**0,63**<br>0,85	0,90<br>1,06	1,06						
Delta(3,5)-Delta(2,4)-dienoyl-CoA isomerase, mitochondrial precursor	Q13011|ECH1_HUMAN	**0,79**<br>0,96	**0,84**<br>**0,87**	**0,64**	**2,44**<br>2,25	**1,94**<br>**1,57**	**4,15**<br>2,52	**0,80**<br>0,77	**1,60**<br>1,39	**1,95**<br>1,81
Density-regulated protein	O43583|DENR_HUMAN							0,90	0,89	0,70
Deoxynucleotidyltransferase terminal-interacting protein 2	Q5QJE6|TDIF2_HUMAN							0,94	0,80	**0,45**
Deoxyribonuclease-2-alpha precursor	O00115|DNS2A_HUMAN	1,04<br>1,03	1,07<br>**0,79**	0,71	 **1,46**	 0,79	 **1,24**			
Deoxyuridine 5'-triphosphate nucleotidohydrolase, mitochondrial precursor	P33316|DUT_HUMAN	 1,04	 0,90							
Derlin-1	Q9BUN8|DERL1_HUMAN	1,04	0,96	1,04						
Desmocollin-2 precursor	Q02487|DSC2_HUMAN							1,00	**0,50**	0,67
Desmoplakin	P15924|DESP_HUMAN				 **1,37**	 **1,50**	 1,50			
Destrin	P60981|DEST_HUMAN				0,88	1,11	1,02			
Diablo homolog, mitochondrial precursor	Q9NR28|DBLOH_HUMAN	0,88<br>**0,90**	0,76<br>**0,83**	0,57						
Diacylglycerol O-acyltransferase 1	O75907|DGAT1_HUMAN	0,00	0,00	0,00						
Diaphanous 1 variant (Fragment)	Q59FH8|Q59FH8_HUMAN				 0,81	 0,90	 0,88			
Dihydrolipoamide branched chain transacylase E2	Q5VVL8|Q5VVL8_HUMAN	0,94	0,83	**0,70**						
Dihydrolipoamide S-acetyltransferase	Q86YI5|Q86YI5_HUMAN	**0,86**	**0,85**	**0,72**						
Dihydrolipoamide S-acetyltransferase (E2 component of pyruvate dehydrogenase complex) variant (Fragment)	Q53EP3|Q53EP3_HUMAN	 1,02	 1,08		 3,25	 1,79	 3,56			
Dihydrolipoyl dehydrogenase, mitochondrial precursor	P09622|DLDH_HUMAN	**0,86**<br>1,01	**0,86**<br>0,95	**0,73**	**2,16**<br>1,87	**1,59**<br>1,39	**2,59**<br>2,14	0,94<br>0,97	**2,41**<br>**1,89**	**5,15**<br>**4,00**
Dihydroorotate dehydrogenase, mitochondrial precursor	Q02127|PYRD_HUMAN	0,74<br>0,90	0,77<br>**0,84**	0,47						
Dihydropteridine reductase	P09417|DHPR_HUMAN	0,49<br>1,09	0,35<br>1,07	0,30						
Dihydropyrimidinase-like 2	Q86U75|Q86U75_HUMAN	1,02<br>0,88	1,41<br>**1,23**	1,77				 0,89	 **3,08**	 **4,38**
Dihydropyrimidinase-like 2 variant (Fragment)	Q59GB4|Q59GB4_HUMAN				**0,87**<br>0,86	**0,87**<br>1,08	**0,81**<br>0,75			
Dipeptidyl peptidase 9	Q86TI2|DPP9_HUMAN				0,00	0,00	0,00			
Dipeptidyl-peptidase 1 precursor	P53634|CATC_HUMAN	1,16<br>**0,82**	**1,68**<br>0,92	1,03	0,42	0,77	0,52			
Dipeptidyl-peptidase 2 precursor	Q9UHL4|DPP2_HUMAN	 1,19	 1,21							
Dipeptidyl-peptidase 3	Q9NY33|DPP3_HUMAN				**0,87**<br>0,97	0,94<br>0,92	0,94<br>0,95			
Diphosphomevalonate decarboxylase	P53602|ERG19_HUMAN				0,97<br>0,98	1,07<br>1,14	**0,75**<br>0,81			
Disabled homolog 2	P98082|DAB2_HUMAN	0,78<br>**1,40**	0,47<br>0,82	0,57	0,76<br>**0,47**	0,63<br>**0,54**	0,44<br>**0,45**	0,69	**0,33**	**0,45**
Disrupted in renal carcinoma protein 2	Q96SL1|DIRC2_HUMAN	0,00<br>0,00	0,00<br>0,97	0,00						
DNA (cytosine-5)-methyltransferase 1	P26358|DNMT1_HUMAN							0,00<br>0,89	0,00<br>0,34	0,00<br>**0,35**
DNA damage-binding protein 1	Q16531|DDB1_HUMAN				 1,23	 1,84	 1,17			
DNA topoisomerase 1	P11387|TOP1_HUMAN							0,98<br>**0,57**	**0,48**<br>**0,61**	**0,87**<br>**0,81**
DNA topoisomerase 2 (Fragment)	Q71UH4|Q71UH4_HUMAN							0,80<br>**0,42**	**0,53**<br>**0,59**	0,54<br>**0,57**
DNA-dependent protein kinase catalytic subunit	P78527|PRKDC_HUMAN							**1,75**<br>**0,35**	0,64<br>**0,48**	**0,70**<br>**0,40**
DNA-directed RNA polymerase, mitochondrial precursor	O00411|RPOM_HUMAN	 0,94	 0,92							
DNA-directed RNA polymerase I subunit RPA34	O15446|RPA34_HUMAN							 1,06	 0,84	 0,60
DNA-directed RNA polymerase II subunit RPB2	P30876|RPB2_HUMAN							 0,00	 0,00	 0,00
DNA-directed RNA polymerase II subunit RPB4	O15514|RPB4_HUMAN							0,92	**0,41**	**0,22**
DNA-directed RNA polymerases I, II, and III subunit RPABC1	P19388|RPAB1_HUMAN							0,96<br>0,91	**0,42**<br>**0,33**	**0,31**<br>**0,45**
DNA-directed RNA polymerases I, II, and III subunit RPABC3	P52434|RPAB3_HUMAN							 0,00	 0,00	 0,00
DnaJ (Hsp40) homolog, subfamily A, member 1	Q5T7Q0|Q5T7Q0_HUMAN	 1,05	 1,12							
DnaJ domain-containing protein RME-8	Q3L0T1|Q3L0T1_HUMAN	 0,90	 0,98							
DnaJ homolog subfamily A member 2	O60884|DNJA2_HUMAN				 0,69	 0,76	 **0,62**			
DnaJ homolog subfamily A member 3, mitochondrial precursor	Q96EY1|DNJA3_HUMAN	1,07<br>**1,20**	0,87<br>1,12	0,82						
DnaJ homolog subfamily B member 11 precursor	Q9UBS4|DJB11_HUMAN	1,01<br>1,00	1,11<br>**1,15**	**1,27**				1,17<br>**1,40**	**2,19**<br>**2,69**	**2,31**<br>**3,82**
DnaJ homolog subfamily B member 12	Q9NXW2|DJB12_HUMAN	0,83	0,97	0,97						

		0,78	1,28							
DnaJ homolog subfamily B member 4	A1A5C4|A1A5C4_HUMAN							0,83	**2,48**	**2,21**
DnaJ homolog subfamily C member 10 precursor	Q8IXB1|DJC10_HUMAN	0,87<br>0,98	0,88<br>1,06	1,01						
DnaJ homolog subfamily C member 11	Q9NVH1|DJC11_HUMAN	1,03<br>1,04	0,99<br>1,06	0,90						
DnaJ homolog subfamily C member 13	O75165|DNJCD_HUMAN	0,96	0,99	1,18						
DnaJ homolog subfamily C member 3 precursor	Q13217|DNJC3_HUMAN	**0,91**<br>0,98	1,01<br>**1,07**	1,00	**2,02**<br>1,23	1,24<br>1,09	1,84<br>1,14	1,13<br>0,99	**4,32**<br>**3,76**	**5,55**<br>**5,46**
DnaJ homolog subfamily C member 5	Q9H3Z4|DNJC5_HUMAN	1,10<br>0,98	1,00<br>0,98	1,19	**2,13**	1,22	1,61			
DnaJ homolog subfamily C member 7	Q99615|DNJC7_HUMAN				0,75<br>**0,50**	0,73<br>0,55	0,53<br>**0,40**			
DnaJ homolog subfamily C member 8	O75937|DNJC8_HUMAN				0,90<br>**0,73**	0,99<br>0,91	0,92<br>**0,77**	1,05<br>1,11	**0,55**<br>0,90	**0,27**<br>**0,65**
DNAJB1 protein	Q6FI51|Q6FI51_HUMAN				0,81	1,09	1,04			
DNCL1 protein	Q6FGH9|Q6FGH9_HUMAN	0,85	**1,47**	2,56	1,11	1,08	0,90	**0,81**	**0,37**	**0,20**
DNL-type zinc finger protein	Q5SXM8|DNLZ_HUMAN	1,02	0,95							
DOC-1R protein	Q6IAV4|Q6IAV4_HUMAN							0,84<br>1,31	**0,17**<br>**0,38**	**0,09**<br>**0,35**
Docking protein 2	O60496|DOK2_HUMAN				0,83<br>0,79	0,63<br>0,73	**0,58**<br>0,58			
Docking protein 3	Q7L591|DOK3_HUMAN	0,91	0,98	1,19	0,94<br>**0,67**	0,62<br>0,58	0,66<br>**0,32**			
DOK1 protein	Q2TA81|Q2TA81_HUMAN				0,00<br>0,00	0,00<br>0,00	0,00<br>0,00			
Dolichyl-diphosphooligosaccharide-protein glycosyltransferase	Q5VWA5|Q5VWA5_HUMAN	1,04<br>0,98	1,03<br>1,07	0,93	2,16	**1,30**	1,24	1,03<br>0,74	**1,73**<br>**1,51**	**2,51**<br>**2,17**
Dolichyl-diphosphooligosaccharide--protein glycosyltransferase subunit 2 precursor	P04844|RPN2_HUMAN	1,02<br>0,96	1,03<br>**1,12**	0,94				**0,65**	**1,73**	**2,60**
Dolichyl-diphosphooligosaccharide--protein glycosyltransferase subunit DAD1	P61803|DAD1_HUMAN	1,05<br>**0,82**	0,92<br>0,96	0,90				1,15<br>1,23	**2,89**<br>2,31	**2,80**<br>2,07
Dolichyl-diphosphooligosaccharide--protein glycosyltransferase subunit STT3A	P46977|STT3A_HUMAN	0,97<br>**0,88**	1,04<br>1,05	0,95						18,16
Dolichyl-diphosphooligosaccharide--protein glycosyltransferase subunit STT3B	Q8TCJ2|STT3B_HUMAN	0,99<br>**0,86**	1,05<br>**1,23**	1,03				0,23	**2,38**	**3,39**
Dolichyl-phosphate beta-glucosyltransferase	Q9Y673|ALG5_HUMAN	**0,87**	1,10							
Dolichyl-phosphate mannosyltransferase polypeptide 1, catalytic subunit	Q96HK0|Q96HK0_HUMAN	0,95	1,01	0,89						
Dolichyl-phosphate mannosyltransferase polypeptide 1, catalytic subunit (Fragment)	Q5QPK0|Q5QPK0_HUMAN	1,04	**1,14**							
Dolichyl-phosphate mannosyltransferase polypeptide 3	Q5SR62|Q5SR62_HUMAN	1,12<br>1,01	1,11<br>0,96	1,26	0,00	0,00	0,00			
Dopamine receptor interacting protein 4	Q4W4Y1|Q4W4Y1_HUMAN							0,89	1,63	1,63
Drebrin-like protein	Q9UJU6|DBNL_HUMAN				0,69	0,68	0,56	0,55<br>0,97	**0,24**<br>0,62	**0,23**<br>**0,52**
DTYMK protein (Fragment)	Q6FGU2|Q6FGU2_HUMAN	0,87<br>**1,11**	0,97<br>1,09	**1,24**						
Dual specificity mitogen-activated protein kinase kinase 2	P36507|MP2K2_HUMAN				0,88	0,89	0,76			
Dual specificity protein phosphatase 23	Q9BVJ7|DUS23_HUMAN				0,00	0,00	0,00			
DUSP3 protein	Q8IYJ9|Q8IYJ9_HUMAN				0,00	0,00	0,00			
DUTP pyrophosphatase	Q6NSA3|Q6NSA3_HUMAN				1,04<br>0,90	0,72<br>1,04	1,09<br>1,22			
Dynactin 2 variant (Fragment)	Q53H88|Q53H88_HUMAN				**0,81**<br>**0,68**	0,92<br>0,80	1,04<br>0,87	0,94<br>**1,51**	**1,32**<br>1,05	0,94<br>0,83
Dynactin subunit 3	O75935|DCTN3_HUMAN				0,60	0,73	**0,72**			
Dynamin-2	P50570|DYN2_HUMAN							**0,62**	**0,75**	**0,65**
Dynamin-like 120 kDa protein, mitochondrial precursor	O60313|OPA1_HUMAN	0,89<br>1,02	**0,76**<br>0,98	**0,62**						
Dynamin-like protein DYNIV-11 variant (Fragment)	Q59GN9|Q59GN9_HUMAN				0,98	0,98	1,09			
Dynein heavy chain 12, axonemal	Q8WXK2|DYH12_HUMAN							6,04	4,42	5,43
Dynein heavy chain 5, axonemal	Q8TE73|DYH5_HUMAN							0,94	0,81	0,81
Dynein light chain 2, cytoplasmic	Q96FJ2|DYL2_HUMAN							0,84	**0,48**	**0,32**
Dysferlin_v1	Q0H395|Q0H395_HUMAN	0,95	0,98							
E2-induced gene 5 protein	Q96A26|E2IG5_HUMAN	0,79<br>0,97	0,87<br>1,00	0,98				1,31<br>1,78	1,52<br>1,46	2,25<br>**2,74**
E3 ubiquitin-protein ligase CBL	P22681|CBL_HUMAN				0,00	0,00	0,00			
E3 ubiquitin-protein ligase synoviolin precursor	Q86TM6|SYVN1_HUMAN	0,89<br>0,95	1,23<br>1,11	1,36						
EARS2 protein	Q86YH3|Q86YH3_HUMAN	1,08<br>0,95	0,85<br>0,91	0,67						
EBNA-2 co-activator variant (Fragment)	Q59FF0|Q59FF0_HUMAN							1,28<br>**0,35**	1,01<br>**0,54**	**1,57**<br>**0,45**
EBP protein (Fragment)	Q6FGL3|Q6FGL3_HUMAN	0,94	0,99	0,92				0,00	0,00	0,00
Ectonucleoside triphosphate diphosphohydrolase 1	P49961|ENTP1_HUMAN	1,16<br>1,00	1,06<br>1,12	**1,29**						
EEF1D protein	Q96I38|Q96I38_HUMAN				**0,64**<br>0,73	0,92<br>0,98	**1,25**<br>1,14			
EF-hand domain-containing protein D2	Q96C19|EFHD2_HUMAN	1,68<br>1,08	2,00<br>**1,20**	2,31	0,78<br>0,71	**1,50**<br>1,11	**1,78**<br>1,10	1,52	0,73	0,74
EH domain-binding protein 1-like protein 1	Q8N3D4|EH1L1_HUMAN	1,01	1,06							
EH domain-containing protein 4	Q9H223|EHD4_HUMAN				**0,73**<br>**0,82**	0,74<br>**0,72**	**0,72**<br>0,56			
EIF2S2 protein	Q96I16|Q96I16_HUMAN				0,70	0,62	**0,70**	0,91	**1,57**	**1,65**
EIF3S3 protein	Q6IB98|Q6IB98_HUMAN				**1,21**<br>1,06	1,21<br>1,07	**1,44**<br>1,14			
EIF3S4 protein	Q6IAM0|Q6IAM0_HUMAN				1,25	0,80	1,19	0,60	0,78	0,42
EIF4G1 variant protein (Fragment)	Q4LE58|Q4LE58_HUMAN	0,83	0,92	0,65	**1,15**	**1,16**	0,99	1,08	0,71	0,51
EIF5 protein	Q6IBU0|Q6IBU0_HUMAN				0,92<br>**0,69**	0,88<br>0,73	**0,78**<br>**0,70**			
ELAV (Embryonic lethal, abnormal vision, Drosophila)-like 1	Q53XN6|Q53XN6_HUMAN							**0,85**<br>1,15	**0,18**<br>**0,33**	**0,14**<br>**0,31**
Electron transfer flavoprotein subunit beta	P38117|ETFB_HUMAN	**0,86**<br>0,95	0,99<br>**0,95**	**0,74**	**1,49**<br>1,81	**1,25**<br>1,31	1,43<br>0,88			
Electron transfer flavoprotein ubiquinone oxidoreductase	Q547S8|Q547S8_HUMAN	1,05<br>1,01	0,86<br>0,96	0,81						

Electron-transfer-flavoprotein, alpha polypeptide	Q53XN3|Q53XN3_HUMAN	**0,87**<br>1,01	0,93<br>**0,89**	0,87	**1,97**<br>**1,80**	**1,73**<br>**1,37**	**1,76**<br>**1,42**			
ELMO domain-containing protein 2	Q8IZ81|ELMD2_HUMAN	0,00<br>0,96	0,00<br>1,13	0,00						
Elongation factor 1-alpha	Q6IPT9|Q6IPT9_HUMAN				**0,73**<br>**0,86**	**0,93**<br>1,02	**0,62**<br>0,72	**1,19**	**1,31**	**1,68**
Elongation factor 1-alpha (Fragment)	Q53HQ7|Q53HQ7_HUMAN	**1,37**<br>**1,49**	**3,76**<br>**2,83**	**10,04**				**0,36**	**1,23**	1,03
Elongation factor 1-beta	P24534|EF1B_HUMAN				**0,70**<br>0,54	1,01<br>**0,89**	**1,17**<br>0,96	**0,70**<br>0,91	**1,99**<br>**1,90**	**1,92**<br>**1,54**
Elongation factor 2	P13639|EF2_HUMAN	1,35<br>1,39	**1,88**<br>**1,55**	**4,83**	**0,93**<br>0,95	**1,07**<br>**1,08**	**0,88**<br>**1,10**	**1,57**<br>0,54	**1,95**<br>**1,64**	**3,48**<br>**1,39**
Elongation factor G 1, mitochondrial precursor	Q96RP9|EFG1_HUMAN	0,94<br>0,97	**0,90**<br>0,97	**0,74**						
Elongation factor G isoform	A0AR28|A0AR28_HUMAN	0,94	1,05							
Elongation factor Ts, mitochondrial precursor	P43897|EFTS_HUMAN	0,79<br>0,99	**0,81**<br>0,95	**0,73**						
Elongation factor Tu, mitochondrial precursor	P49411|EFTU_HUMAN	0,98<br>1,05	0,96<br>**0,93**	**0,84**	**1,91**<br>2,11	**1,39**<br>**1,34**	1,21<br>**1,32**	0,00	0,00	0,00
Elongation of very long chain fatty acids protein 1	Q9BW60|ELOV1_HUMAN	0,81	0,58							
Elongation of very long chain fatty acids protein 5	Q9NYP7|ELOV5_HUMAN	0,86	0,68							
EMD protein	Q6FI02|Q6FI02_HUMAN	0,82	1,05	1,16				1,02<br>1,28	0,78<br>0,86	0,67<br>0,78
Ena/VASP-like protein	Q9UI08|EVL_HUMAN	1,81<br>0,98	**2,54**<br>1,10	**9,31**	0,86<br>0,84	1,12<br>1,03	1,08<br>0,92	0,85<br>0,98	0,90<br>1,10	**0,38**<br>**0,63**
Endoglin	Q5T9C0|Q5T9C0_HUMAN	0,00<br>0,89	0,00<br>0,80	0,00						
Endophilin-B1	Q9Y371|SHLB1_HUMAN				0,73	0,88	**0,63**			
Endoplasmic reticulum aminopeptidase 1	Q9NZ08|ERAP1_HUMAN	0,87<br>1,05	1,00<br>**1,13**	**0,87**						
Endoplasmic reticulum aminopeptidase 2	Q6P179|ERAP2_HUMAN	1,07<br>0,99	1,14<br>1,06	0,83						
Endoplasmic reticulum metallopeptidase 1	Q7Z2K6|ERMP1_HUMAN	**0,78**<br>1,05	1,04<br>0,98	0,99						
Endoplasmic reticulum protein ERp29 precursor	P30040|ERP29_HUMAN	0,87<br>1,01	0,97<br>**1,11**	**0,80**	**1,87**<br>**1,54**	1,10<br>1,16	**2,17**<br>**1,52**	**1,34**<br>**1,31**	**2,90**<br>**2,30**	**4,39**<br>**2,92**
Endoplasmic reticulum-Golgi intermediate compartment protein 1	Q969X5|ERGI1_HUMAN	1,18	1,23							
Endoplasmic reticulum-Golgi intermediate compartment protein 3	Q9Y282|ERGI3_HUMAN	**1,41**<br>1,13	**0,84**<br>**1,25**	0,96						
Endothelial differentiation inhibitory protein D18	Q4G5Y3|Q4G5Y3_HUMAN							0,88	0,66	0,57
Endothelial differentiation-related factor 1	O60869|EDF1_HUMAN							0,97<br>1,03	**0,39**<br>**0,57**	**0,29**<br>0,61
Engulfment and cell motility protein 1	Q92556|ELMO1_HUMAN				1,25	1,27	1,04			
Enhancer of rudimentary homolog	P84090|ERH_HUMAN							**0,86**<br>0,97	**0,56**<br>1,04	**0,25**<br>**0,65**
Enolase (Fragment)	Q53FT9|Q53FT9_HUMAN	**1,48**<br>**1,53**	**2,01**<br>**1,58**	**2,01**				0,88	**0,61**	**0,44**
Enoyl Coenzyme A hydratase domain containing 3	Q96DC8|Q96DC8_HUMAN	0,69	0,88							
Enoyl-CoA hydratase, mitochondrial precursor	P30084|ECHM_HUMAN	**0,85**<br>1,05	**0,88**<br>1,04	**0,68**	**1,72**<br>1,62	**1,47**<br>**1,23**	**2,82**<br>1,68			
Enoyl-Coenzyme A, hydratase/3-hydroxyacyl Coenzyme A dehydrogenase	Q58EZ5|Q58EZ5_HUMAN	0,97	1,12							
Eosinophil cationic protein precursor	P12724|ECP_HUMAN	0,00	0,00							
Epidermal growth factor receptor substrate 15-like 1	Q9UBC2|EP15R_HUMAN	1,10	0,92							
Epididymal secretory protein E1 precursor	P61916|NPC2_HUMAN	1,08<br>0,93	**1,41**<br>0,95	1,10	**1,65**<br>**1,37**	1,07<br>0,97	**1,55**<br>**1,55**			
Epoxide hydrolase 1	P07099|HYEP_HUMAN	1,00<br>0,93	1,16<br>**1,18**	1,11						
Equilibrative nucleoside transporter 3	Q9BZD2|S29A3_HUMAN	0,65	0,81	0,55						
ER lipid raft associated 1	B0QZ42|B0QZ42_HUMAN	0,95	1,01							
ER lumen protein retaining receptor	Q6IPC5|Q6IPC5_HUMAN	1,05	0,64							
ER lumen protein retaining receptor 2	P33947|ERD22_HUMAN	0,89	0,80	**0,78**						
Erlin-1	O75477|ERLN1_HUMAN	1,85	**2,51**	2,32						
Erlin-2	O94905|ERLN2_HUMAN	0,91<br>0,95	**1,22**<br>**1,16**	**1,11**						
ERO1-like protein alpha precursor	Q96HE7|ERO1A_HUMAN	0,92	1,03	0,96	1,62	1,17	1,51			
Erythrocyte band 7 integral membrane protein	P27105|STOM_HUMAN	0,97<br>0,91	1,18<br>**0,66**	**1,22**				1,17	**2,04**	2,70
Erythrocyte membrane protein band 4,1-like 2	Q5T4F0|Q5T4F0_HUMAN	0,98<br>1,02	0,91<br>1,22	1,03						
ES1 protein homolog, mitochondrial precursor	P30042|ES1_HUMAN	0,94<br>0,93	0,80<br>0,89	**0,62**	**2,30**<br>**1,74**	**2,08**<br>1,81	**3,19**<br>**1,95**			
Estradiol 17-beta-dehydrogenase 11 precursor	Q8NBQ5|DHB11_HUMAN	1,11<br>1,00	1,12<br>1,12	1,26						
Estradiol 17-beta-dehydrogenase 12	Q53GQ0|DHB12_HUMAN	**0,88**<br>0,91	1,00<br>1,10	1,00	1,86	1,09	0,93	0,98<br>0,78	**3,65**<br>**3,63**	**6,67**<br>**7,03**
Estradiol 17-beta-dehydrogenase 8	Q92506|DHB8_HUMAN	0,92	1,00							
ETF1 protein	Q96CG1|Q96CG1_HUMAN				**0,89**	0,86	0,78			
Ethanolamine-phosphate cytidylyltransferase	Q99447|PCY2_HUMAN				0,00	0,00	0,00			
ETHE1 protein, mitochondrial precursor	O95571|ETHE1_HUMAN	1,03<br>1,00	1,06<br>0,91	0,68	**1,73**	1,14	1,30			
Eukaryotic initiation factor 4A-I	P60842|IF4A1_HUMAN	1,64	**6,42**	**14,83**	0,81<br>0,89	0,90<br>**0,80**	0,62<br>0,85			
Eukaryotic initiation factor 4B	Q8WYK5|Q8WYK5_HUMAN				0,94<br>**0,71**	0,95<br>**0,69**	**0,74**<br>0,69	0,89<br>1,50	**0,30**<br>0,49	**0,20**<br>0,36
Elongation factor 1- gamma	P26641|EF1G_HUMAN	0,00	0,00		**0,85**<br>**0,76**	**1,18**<br>1,11	**1,28**<br>1,08	0,81<br>**0,46**	**1,68**<br>**1,19**	**2,24**<br>1,04
Eukaryotic translation initiation factor 1A, X-chromosomal	P47813|IF1AX_HUMAN							0,97<br>**1,20**	**1,28**<br>**1,42**	1,11<br>**1,41**
Eukaryotic translation initiation factor 2A	Q9BY44|EIF2A_HUMAN							0,56	0,73	0,60
Eukaryotic translation initiation factor 3 subunit A	Q14152|EIF3A_HUMAN							0,89	**0,64**	0,81
Eukaryotic translation initiation factor 3 subunit B	P55884|EIF3B_HUMAN				1,19<br>1,35	1,12<br>1,84	1,11<br>2,21			
Eukaryotic translation initiation factor 3 subunit C	Q99613|EIF3C_HUMAN				0,89<br>1,88	1,05<br>2,39	**1,24**<br>1,85	0,82<br>0,98	0,69<br>0,87	0,68<br>0,65
Eukaryotic translation initiation factor 3 subunit J	O75822|EIF3J_HUMAN				**0,77**<br>**0,52**	0,92<br>**0,81**	1,01<br>0,93	0,73<br>1,04	0,64<br>0,86	0,69<br>0,89
	Q5U0F4|Q5U0F4_HUMAN				1,16	1,21	1,14			

Eukaryotic translation initiation factor 3, subunit 2 beta, 36kDa										
Eukaryotic translation initiation factor 3, subunit A	Q24JU4|Q24JU4_HUMAN				1,03<br>**1,31**	1,10<br>**1,23**	1,36<br>**1,52**			
Eukaryotic translation initiation factor 3, subunit E	Q8WVK4|Q8WVK4_HUMAN				0,85	0,89	0,75			
Eukaryotic translation initiation factor 3, subunit E interacting protein	B0QY89|B0QY89_HUMAN				0,88	0,93	0,95			
Eukaryotic translation initiation factor 4 gamma 1	Q04637|IF4G1_HUMAN				1,01	0,97	0,80	0,88	0,86	0,87
Eukaryotic translation initiation factor 4 gamma, 2 variant (Fragment)	Q59G42|Q59G42_HUMAN				0,75	0,88	0,38			
Eukaryotic translation initiation factor 4E	Q32Q75|Q32Q75_HUMAN							1,08	0,68	0,75
Eukaryotic translation initiation factor 4H	Q15056|IF4H_HUMAN				0,91<br>**0,57**	0,87<br>**0,72**	**0,62**<br>**0,67**	1,10<br>0,89	**0,68**<br>0,63	**0,42**<br>**0,57**
Eukaryotic translation initiation factor 5A-1	P63241|IF5A1_HUMAN	0,94	**2,76**	**7,19**	**0,79**<br>0,57	0,90<br>**0,72**	**0,68**<br>0,55	**1,24**<br>**1,37**	**2,15**<br>**2,19**	**1,44**<br>**1,53**
Eukaryotic translation initiation factor 5B	Q8N5A0|Q8N5A0_HUMAN							1,14<br>1,10	2,31<br>**2,20**	**2,14**<br>**1,96**
EVH1 domain binding protein	Q9NZI9|Q9NZI9_HUMAN				1,08	1,76	1,98			
Evolutionarily conserved signaling intermediate in Toll pathway, mitochondrial precursor	Q9BQ95|ECSIT_HUMAN	0,00<br>0,93	0,00<br>0,93	0,00						
Exosome complex exonuclease MTR3	Q5RKV6|EXOS6_HUMAN							0,00	0,00	0,00
Exportin-1	O14980|XPO1_HUMAN				0,00	0,00	0,00			
Exportin-2	P55060|XPO2_HUMAN				1,28<br>0,85	1,43<br>0,62	1,51<br>0,60			
Extended-synaptotagmin-1	Q9BSJ8|ESYT1_HUMAN	1,00<br>1,03	0,98<br>**1,20**	1,03	**2,03**<br>**2,43**	1,30<br>1,88	**1,40**<br>1,44	2,19<br>**0,70**	**3,25**<br>1,80	4,15<br>2,72
Extracellular signal-related kinase 1c	Q8NHX0|Q8NHX0_HUMAN				1,09	0,89	0,52			
Ezrin	P15311|EZRI_HUMAN	**1,67**	**1,48**		0,95<br>**0,84**	1,04<br>0,99	**1,14**<br>0,94	**1,50**<br>1,04	1,01<br>1,03	1,19<br>**1,12**
Ezrin-radixin-moesin-binding phosphoprotein 50	O14745|NHERF_HUMAN				1,16<br>0,55	0,90<br>0,74	**0,68**<br>**0,63**			
FABP3 protein	Q6IBD7|Q6IBD7_HUMAN				0,92<br>0,83	1,02<br>**0,89**	0,99<br>1,34			
FABP4 protein	Q6IBA1|Q6IBA1_HUMAN				**1,23**<br>**0,77**	**1,65**<br>1,14	1,20<br>1,01			
F-actin-capping protein subunit alpha-1	P52907|CAZA1_HUMAN	0,96	0,96		1,01<br>1,11	1,15<br>1,12	1,10<br>1,13	1,23<br>1,18	**1,94**<br>1,58	1,49<br>1,10
F-actin-capping protein subunit alpha-2	P47755|CAZA2_HUMAN				1,04	0,93	0,89			
FAD-dependent oxidoreductase domain-containing protein 1	Q96CU9|FXRD1_HUMAN	1,03	0,95							
Family with sequence similarity 129, member B	Q5VVW7|Q5VVW7_HUMAN				0,88<br>**0,81**	0,93<br>0,89	1,09<br>0,87			
Family with sequence similarity 39, member B	Q76LA1|Q76LA1_HUMAN							0,87	1,56	2,07
Far upstream element-binding protein 1	Q96AE4|FUBP1_HUMAN				1,08	**1,43**	1,22	**1,54**	**0,53**	**0,51**
Far upstream element-binding protein 2	Q92945|FUBP2_HUMAN				1,07<br>1,06	1,27<br>**1,39**	1,17<br>1,23	**1,16**<br>**0,77**	**0,33**<br>**0,60**	**0,33**<br>**0,53**
Far upstream element-binding protein 3	Q96I24|FUBP3_HUMAN							1,00	0,17	0,12
Farnesyl diphosphate synthase	Q96G29|Q96G29_HUMAN	1,07	0,97		0,82<br>0,88	**0,76**<br>0,91	**0,75**<br>**0,69**			
Fas apoptotic inhibitory molecule 2	Q9BWQ8|FAIM2_HUMAN				0,00<br>1,76	0,00<br>1,17	0,00<br>1,36			
FASN variant protein (Fragment)	Q4LE83|Q4LE83_HUMAN	0,82	**0,70**	0,74	**0,85**<br>1,01	0,99<br>**1,16**	1,00<br>**1,20**			
FAST kinase domain-containing protein 2	Q9NYY8|FAKD2_HUMAN	1,05	1,06							
FAST kinase domain-containing protein 5	Q7L8L6|FAKD5_HUMAN	0,99	0,98							
Fatty acid-binding protein, epidermal	Q01469|FABP5_HUMAN	**1,58**<br>1,55	1,47<br>**1,52**	**1,90**	**0,80**<br>**0,63**	1,03<br>0,91	**0,86**<br>**0,76**			
Fatty acyl-CoA reductase 1	Q8WVX9|FACR1_HUMAN	0,90<br>0,97	0,85<br>1,08	**0,74**						
Fc gamma receptor I	Q92637|Q92637_HUMAN	0,00	0,00	0,00						
Fc of IgG, low affinity IIa, receptor	Q8WW64|Q8WW64_HUMAN	1,02	0,92							
FCH and double SH3 domains protein 2	O94868|FCSD2_HUMAN				0,00<br>0,00	0,00<br>0,00	0,00<br>0,00			
FDFT1 protein	Q6IAX1|Q6IAX1_HUMAN	0,98<br>0,88	1,03<br>0,98	1,05						
FEN1 protein	Q6FHX6|Q6FHX6_HUMAN				0,00	0,00	0,00	0,82	**0,31**	**0,26**
Fermitin family homolog 3	Q86UX7|URP2_HUMAN	**1,38**<br>1,14	**2,08**<br>**1,69**	**4,88**	0,90<br>1,04	1,09<br>1,69	0,92<br>1,47	0,85	1,79	1,50
Ferredoxin reductase	Q6GSK2|Q6GSK2_HUMAN	0,89<br>0,96	0,80<br>0,95	0,60						
Ferritin	Q6NZ44|Q6NZ44_HUMAN	0,82	**0,63**		0,93	0,95	1,04			
Ferritin (Fragment)	Q6NS36|Q6NS36_HUMAN	1,11	1,41	0,62	0,85	1,46	1,10			
Ferritin, light polypeptide	Q8WU07|Q8WU07_HUMAN	1,08	0,93		0,88	**0,88**	1,00			
Ferritin light polypeptide variant	Q6S4P3|Q6S4P3_HUMAN	0,94	1,01	0,83	1,09	**0,91**	**1,11**			
Ferrochelatase	Q7KZA3|Q7KZA3_HUMAN	1,00	1,00							
Ferrochelatase (Fragment)	Q53FU1|Q53FU1_HUMAN	0,96	0,90	0,80						
FH1/FH2 domain-containing protein 1	Q9Y613|FHOD1_HUMAN				1,29	0,84	0,81			
Fibrinogen-like 2 variant (Fragment)	Q53GD2|Q53GD2_HUMAN	0,94<br>0,92	0,91<br>0,89	1,20						
Ficolin (Collagen/fibrinogen domain containing) 1	Q5VYV5|Q5VYV5_HUMAN	1,00	0,99							
Filamin A	P21333|FLNA_HUMAN	**1,49**<br>**1,52**	**1,87**<br>**1,51**	**4,62**	**0,91**<br>1,02	1,01<br>1,04	**0,96**<br>1,00	**1,26**<br>0,24	**0,43**<br>0,28	**0,76**<br>0,26
Filamin B	Q60FE7|Q60FE7_HUMAN				0,89<br>0,89	1,10<br>1,07	1,00<br>1,21			
FK506 binding protein 5	Q5TGM6|Q5TGM6_HUMAN				0,92<br>0,90	1,01<br>1,18	0,85<br>0,92			
FK506-binding protein 15	Q5T1M5|FKB15_HUMAN							0,86<br>0,82	**0,51**<br>0,68	0,57<br>**0,32**
FK506-binding protein 3	Q00688|FKBP3_HUMAN				**0,71**	**0,82**	0,89	**0,86**<br>**1,26**	**0,64**<br>0,97	**0,48**<br>0,90
FK506-binding protein 4	Q02790|FKBP4_HUMAN				**0,78**<br>0,97	0,89<br>0,88	**0,78**<br>0,99			
Flavin reductase	P30043|BLVRB_HUMAN		2,82	6,52	0,83<br>0,80	**0,74**<br>0,82	**0,59**<br>**0,53**			
FLJ00395 protein (Fragment)	Q6ZNI9|Q6ZNI9_HUMAN	1,13	1,12							

FLOT2 protein	Q6FG43|Q6FG43_HUMAN	**1,17**<br>**1,20**	**1,26**<br>**1,19**	**1,29**	1,28	0,84	0,85	1,35<br>0,92	0,95<br>1,20	**2,49**<br>1,27
Flotillin 1	Q5ST80|Q5ST80_HUMAN	0,99<br>**1,09**	**1,28**<br>1,11	1,06						
Formin-like protein 1	O95466|FMNL_HUMAN	1,14<br>1,04	2,14<br>1,07	2,94						
Frataxin, mitochondrial precursor	Q16595|FRDA_HUMAN	1,01	0,94							
Fructose-bisphosphate aldolase A	P04075|ALDOA_HUMAN	**1,37**<br>**1,50**	**2,03**<br>**1,83**	**3,65**	**0,86**<br>0,96	**0,92**<br>0,99	0,97<br>1,00	**1,40**<br>**0,69**	**0,79**<br>0,98	**1,63**<br>**1,36**
Fructose-bisphosphate aldolase C	P09972|ALDOC_HUMAN				0,00	0,00	0,00			
FSCN1 protein (Fragment)	Q96IH1|Q96IH1_HUMAN				1,06	**1,29**	**1,51**			
FtsH homolog	Q9Y2Q2|Q9Y2Q2_HUMAN							0,80<br>1,31	0,80<br>**1,60**	0,74<br>1,40
Full-length cDNA 5-PRIME end of clone CS0DF013YM24 of Fetal brain of Homo sapiens (Human) variant (Fragment)	Q53GF9|Q53GF9_HUMAN	1,20	1,01	1,08				0,93<br>2,05	**6,69**<br>4,64	**10,09**<br>**7,51**
Full-length cDNA clone CS0DC004YJ14 of Neuroblastoma of Homo sapiens (Fragment)	Q86TW1|Q86TW1_HUMAN							0,00	0,00	0,00
Full-length cDNA clone CS0DJ012YD11 of T cells (Jurkat cell line) of Homo sapiens	Q86SZ6|Q86SZ6_HUMAN	0,97	0,91							
Full-length cDNA clone CS0DL010YH08 of B cells (Ramos cell line) of Homo sapiens	Q96QL0|Q96QL0_HUMAN	0,79	0,53	0,85	0,81	0,71	0,76	0,95	1,39	**2,08**
Full-length cDNA clone CS0DL010YH08 of B cells (Ramos cell line) of Homo sapiens	Q53XC0|Q53XC0_HUMAN	1,35	1,24					0,60	1,44	1,45
Full-length cDNA clone CS0DM004YH09 of Fetal liver of Homo sapiens	Q53XB6|Q53XB6_HUMAN	**1,23**	**1,63**		**0,88**	**1,11**	1,08	**1,38**<br>0,56	**4,64**<br>2,91	**7,54**<br>3,18
Fumarate hydratase, mitochondrial precursor	P07954|FUMH_HUMAN	1,01<br>0,95	**0,92**<br>**0,93**	**0,77**	**1,34**<br>1,49	**1,21**<br>**1,27**	**1,92**<br>**1,69**			
Fumarylacetoacetate hydrolase domain-containing protein 1	Q6P587|FAHD1_HUMAN	**0,79**<br>0,94	0,86<br>0,90	**0,50**						
Fumarylacetoacetate hydrolase domain-containing protein 2A	Q96GK7|FAH2A_HUMAN	0,99	1,12							
FUN14 domain containing 2 (Fragment)	Q5HYP8|Q5HYP8_HUMAN	1,02<br>1,07	1,04<br>1,09	0,85					**11,42**	**12,61**
Fusion	Q8TBR3|Q8TBR3_HUMAN	0,85	0,94		0,97	1,01	0,61	**1,58**	0,73	0,66
Fusion protein SYT-SSX2	A4PIW0|A4PIW0_HUMAN							1,15	0,62	0,64
FUS interacting protein (Serine/arginine-rich) 1	Q5JRI0|Q5JRI0_HUMAN							0,74	0,58	0,68
FUS interacting protein (Serine-arginine rich) 1 isoform 2 variant (Fragment)	Q53GD7|Q53GD7_HUMAN							0,83	**0,27**	**0,20**
Fus-like protein (Fragment)	Q13344|Q13344_HUMAN	1,18	1,50	2,30	1,18	1,28	0,61	**1,32**	**0,38**	**0,14**
FWP004	Q5QTS3|Q5QTS3_HUMAN				1,24	1,10	0,91			
G3BP protein	Q6FI03|Q6FI03_HUMAN				1,57<br>1,60	1,06<br>1,08	0,81<br>0,86	0,78	**0,42**	**0,22**
GABARAPL2 protein	Q6FG91|Q6FG91_HUMAN				0,70	0,80	0,64			
Galactocerebrosidase precursor	P54803|GALC_HUMAN	0,91	1,11							
Galactosidase, alpha	Q53Y83|Q53Y83_HUMAN				**1,76**	**0,79**	0,89			
Galectin-1	P09382|LEG1_HUMAN	**2,73**<br>**1,96**	**5,82**<br>**2,78**	**3,97**	**1,19**<br>**1,11**	**0,84**<br>0,88	**0,87**<br>0,94	**4,07**<br>**2,88**	**2,50**<br>**3,06**	**1,78**<br>**1,72**
Galectin-3	P17931|LEG3_HUMAN	1,07<br>**1,36**	0,97<br>1,08	**0,77**	**1,15**	1,02	1,08			
Galectin-9	O00182|LEG9_HUMAN	**1,59**<br>1,07	**1,29**<br>**1,42**	**1,12**	0,00	0,00	0,00	2,09<br>0,66	0,92<br>0,49	1,13<br>0,56
Galectin-9-like protein	Q3B8N2|LEG9L_HUMAN	0,00	0,00							
Galphai2 protein	Q6B6N3|Q6B6N3_HUMAN							1,00	1,21	1,21
Gamma-glutamyl hydrolase precursor	Q92820|GGH_HUMAN	0,68	1,32	0,99	**1,47**	1,41	1,30	0,00<br>1,94	0,00<br>0,63	0,00<br>0,92
Gamma-interferon-inducible lysosomal thiol reductase precursor	P13284|GILT_HUMAN	**1,08**	0,93							
Gamma-interferon-inducible protein Ifi-16	Q16666|IF16_HUMAN				0,00	0,00	0,00	**0,73**<br>**0,45**	**0,44**<br>0,76	0,42<br>0,67
Gamma-parvin	Q9HBI0|PARVG_HUMAN				**0,47**	**0,57**	**0,50**			
Gamma-soluble NSF attachment protein	Q99747|SNAG_HUMAN	0,00<br>0,96	0,00<br>0,97	0,00	0,00	0,00	0,00			
Ganglioside GM2 activator precursor	P17900|SAP3_HUMAN	**0,85**<br>0,93	**0,64**<br>**0,76**	**0,56**	**1,91**<br>**1,33**	**0,77**<br>0,62	0,98<br>0,82			
GatC-like protein	O43716|GATCL_HUMAN	0,82	0,91							
GBAS protein (Fragment)	Q53X96|Q53X96_HUMAN	0,86<br>0,91	**0,81**<br>0,93	**0,72**				1,03	**2,34**	**1,59**
GBP2 protein	Q6IAU2|Q6IAU2_HUMAN				0,90<br>0,00	1,04<br>0,00	0,86<br>0,00			
GCN1-like protein 1	Q92616|GCN1L_HUMAN	0,85	0,83	0,93						
GDH/6PGL endoplasmic bifunctional protein precursor	O95479|G6PE_HUMAN	0,79<br>**0,83**	0,97<br>0,92	0,89						
GDI2 protein	Q6IAT1|Q6IAT1_HUMAN				**0,87**<br>0,93	1,04<br>0,94	0,89<br>0,97			
GDP-fucose protein O-fucosyltransferase 1 precursor	Q9H488|OFUT1_HUMAN	1,06<br>0,81	1,11<br>1,11	1,06						
GDP-L-fucose synthetase	Q13630|FCL_HUMAN				0,92	1,07	0,65			
Gelsolin	P06396|GELS_HUMAN	**1,78**<br>**1,20**	**3,43**<br>**1,97**	**7,23**	**0,82**<br>**0,94**	0,97<br>1,17	**0,95**<br>**1,15**	1,02<br>**0,63**	**2,69**<br>**1,68**	**2,35**<br>**1,60**
GEM-interacting protein	Q9P107|GMIP_HUMAN				0,00	0,00	0,00			
Gene expressed in odontoblast protein	Q6EVH4|Q6EVH4_HUMAN	1,13	1,00							
GH3 domain-containing protein precursor	Q8N2G8|GHDC_HUMAN	**1,42**<br>0,97	1,33<br>1,02	1,39						
Girdin	Q3V6T2|GRDN_HUMAN				1,05	1,09	1,16			
Glia maturation factor gamma	Q8TDZ6|Q8TDZ6_HUMAN				0,85<br>**0,72**	0,94<br>0,89	0,79<br>**0,71**			
Glucose-6-phosphate 1-dehydrogenase	P11413|G6PD_HUMAN	1,20	**2,24**	**5,21**	0,92<br>0,94	1,07<br>**1,36**	1,00<br>1,10			
Glucose-6-phosphate 1-dehydrogenase	A8K8D9|A8K8D9_HUMAN	1,05	1,27							
Glucose-6-phosphate 1-dehydrogenase (Fragment)	Q2VF42|Q2VF42_HUMAN							0,81	**4,95**	**10,79**
Glucose-6-phosphate isomerase	P06744|G6PI_HUMAN				**0,87**<br>**0,88**	0,96<br>0,95	0,96<br>**0,85**			
Glucosidase 2 subunit beta precursor	P14314|GLU2B_HUMAN	1,01<br>0,97	**1,12**<br>**1,14**	1,08	**1,92**<br>1,75	**1,41**<br>1,15	**2,41**<br>1,67	0,81<br>0,00	**2,48**<br>0,00	**4,20**<br>0,00
Glucosidase I	Q58F09|Q58F09_HUMAN	0,94	1,05	0,90				1,09<br>**0,67**	**2,03**<br>**2,72**	**4,03**<br>**3,48**
Glucosylceramidase precursor	P04062|GLCM_HUMAN	1,00	1,06	**0,80**						

		0,94	1,02		**2,76**	1,01	1,20			
Glutamate dehydrogenase 1, mitochondrial precursor	P00367|DHE3_HUMAN	0,98	0,98							
Glutamate dehydrogenase 1 variant (Fragment)	Q53GW3|Q53GW3_HUMAN	**0,90**	**0,87**	**0,77**	**1,99**<br>**2,43**	**1,51**<br>**1,49**	**2,57**<br>**2,20**	**1,37**<br>**0,56**	**3,41**<br>**3,11**	**8,08**<br>**4,76**
Glutaminase kidney isoform, mitochondrial precursor	O94925|GLSK_HUMAN	**0,81**<br>0,93	**0,83**<br>0,95	**0,68**						
Glutamine synthetase	P15104|GLNA_HUMAN				0,82	0,98	0,98			
Glutaminyl-tRNA synthetase variant (Fragment)	Q53HS0|Q53HS0_HUMAN	1,08	1,36	1,56	0,89	**0,86**	0,97			
Glutaredoxin-1	P35754|GLRX1_HUMAN	1,10	1,25		0,90	0,97	0,88			
Glutaredoxin-related protein 5	Q86SX6|GLRX5_HUMAN	0,88<br>0,93	0,83<br>0,88	0,61						
Glutaryl-CoA dehydrogenase, mitochondrial precursor	Q92947|GCDH_HUMAN	0,92<br>1,02	0,97<br>1,02	0,87						
Glutathione peroxidase	Q6NSD4|Q6NSD4_HUMAN				0,00	0,00	0,00			
Glutathione peroxidase 1	P07203|GPX1_HUMAN	1,05<br>**0,89**	1,26<br>0,92	**1,45**						
Glutathione reductase, mitochondrial precursor	P00390|GSHR_HUMAN	0,92<br>0,97	1,20<br>0,99	1,24	0,95<br>1,01	**1,32**<br>1,13	1,22<br>1,22			
Glutathione S-transferase kappa 1	Q9Y2Q3|GSTK1_HUMAN	1,02	**0,92**		**1,85**	1,25	**1,30**	**1,64**<br>1,42	**4,39**<br>**3,45**	**2,99**<br>**3,10**
Glutathione S-transferase P	P09211|GSTP1_HUMAN				0,82<br>**0,70**	0,94<br>0,87	0,84<br>0,89			
Glutathione synthetase	P48637|GSHB_HUMAN				1,10<br>**0,78**	**0,91**<br>0,74	1,01<br>**0,60**			
Glutathione transferase omega-1	P78417|GSTO1_HUMAN	0,00<br>0,00	0,00<br>0,00	0,00	**0,79**<br>**0,71**	0,95<br>0,84	0,86<br>**0,67**			
Glyceraldehyde 3-phosphate dehydrogenase (Fragment)	Q53X65|Q53X65_HUMAN	1,10<br>**1,23**	**2,07**<br>**1,65**	**5,30**	**0,87**<br>0,98	1,01<br>**1,09**	0,99<br>**1,10**	1,07<br>**0,73**	**2,22**<br>**1,71**	**4,19**<br>**2,24**
Glycerol kinase	Q6IQ27|Q6IQ27_HUMAN	0,97<br>1,01	1,14<br>1,02	1,13						
Glycerol-3-phosphate dehydrogenase 1-like protein	Q8N335|GPD1L_HUMAN	1,15	1,13							
Glycerol-3-phosphate dehydrogenase, mitochondrial precursor	P43304|GPDM_HUMAN	0,96	1,03							
Glyceronephosphate O-acyltransferase variant (Fragment)	Q59EC9|Q59EC9_HUMAN	0,99	1,11							
Glycerophosphodiester phosphodiesterase 1	Q9NZC3|GDE1_HUMAN	1,07<br>0,94	1,08<br>1,06	1,23						
Glycogen phosphorylase, liver form	P06737|PYGL_HUMAN				0,86<br>0,80	0,92<br>0,94	0,88<br>0,96			
Glycolipid transfer protein	Q9NZD2|GLTP_HUMAN				0,92	0,94	0,79			
Glycosyltransferase 25 family member 1 precursor	Q8NBJ5|GT251_HUMAN	1,04<br>0,97	1,08<br>**1,12**	1,00	**2,45**	1,28	1,42	1,25<br>**0,61**	**2,21**<br>**1,75**	**3,94**<br>2,91
Glycyl-tRNA synthetase	P41250|SYG_HUMAN	1,03<br>0,99	1,06<br>0,99	1,16	**0,83**<br>0,96	0,89<br>1,04	0,94<br>1,07			
Glypican-4 precursor	O75487|GPC4_HUMAN	0,81	**0,75**							
GNAS complex locus	Q5FWY2|Q5FWY2_HUMAN	**1,28**	1,02							
GNB1 protein (Fragment)	Q1RMY8|Q1RMY8_HUMAN				2,01	1,14	1,36			
GNB2 protein	Q6FHM2|Q6FHM2_HUMAN	1,53<br>**0,85**	1,11<br>0,94	1,09	**1,88**	1,21	1,31			
GNB2L1 protein (Fragment)	Q6FH47|Q6FH47_HUMAN							**1,26**	0,78	1,18
Golgi apparatus protein 1	Q6P9D1|Q6P9D1_HUMAN	0,99	**0,82**					**1,67**	**2,26**	**4,01**
Golgi apparatus protein 1 precursor	Q92896|GSLG1_HUMAN	**1,08**	**0,64**	**0,70**				**1,37**	**2,69**	**4,13**
Golgi autoantigen, golgin subfamily a, 2 (Fragment)	Q5SYX5|Q5SYX5_HUMAN	1,29	0,97	**1,29**						
Golgi integral membrane protein 4	O00461|GOLI4_HUMAN	0,83<br>1,04	0,95<br>0,96	0,93				1,79<br>1,93	2,36<br>1,50	**3,78**<br>3,92
Golgi membrane protein 1	Q8NBJ4|GOLM1_HUMAN	1,26	1,06							
Golgi phosphoprotein 3	Q9H4A6|GOLP3_HUMAN	**1,46**	1,82							
Golgi reassembly-stacking protein 2	Q9H8Y8|GORS2_HUMAN				1,09	1,10	0,96			
Golgi resident protein GCP60	Q9H3P7|GCP60_HUMAN	0,33	0,87	1,54						
Golgi SNAP receptor complex member 1	O95249|GOSR1_HUMAN	1,19	1,00							
Golgi-associated plant pathogenesis-related protein 1	Q9H4G4|GAPR1_HUMAN	1,15<br>0,98	**0,82**<br>0,99	0,82	**1,67**	0,85	0,84	**1,42**<br>**1,35**	**0,84**<br>0,89	**0,74**<br>0,77
Golgin subfamily A member 5	Q8TBA6|GOGA5_HUMAN	1,08		1,04						
Golgin subfamily B member 1	Q14789|GOGB1_HUMAN	0,96<br>**1,19**	0,79<br>1,02	0,96	0,00	0,00	0,00			
Golgi-specific brefeldin A-resistance guanine nucleotide exchange factor 1	Q92538|GBF1_HUMAN	1,39<br>1,03	0,72<br>1,22	1,14						
GPI transamidase component PIG-S	Q96S52|PIGS_HUMAN	0,95<br>1,01	1,13<br>1,05	1,02				1,07	0,20	0,11
GPI transamidase component PIG-T precursor	Q969N2|PIGT_HUMAN	1,01	1,27							
GPI-anchor transamidase precursor	Q92643|GPI8_HUMAN	0,92	0,99							
G-protein coupled receptor 98 precursor	Q8WXG9|GPR98_HUMAN	1,03	0,88	1,15						
Grancalcin	P28676|GRAN_HUMAN				0,00	0,00	0,00			
Graves disease carrier protein	P16260|GDC_HUMAN	0,00	0,00	0,00						
GRB2 protein (Fragment)	Q6ICN0|Q6ICN0_HUMAN				0,86<br>**0,71**	**0,86**<br>0,86	**0,78**<br>**0,71**			
GRHPR protein (Fragment)	Q5M7Z5|Q5M7Z5_HUMAN	0,92	1,01							
G-rich sequence factor 1	Q12849|GRSF1_HUMAN	1,01<br>0,94	**0,68**<br>0,92	**0,55**						
GRIP1-associated protein 1	Q4V328|GRAP1_HUMAN				0,90	0,83	0,65			
Growth arrest and DNA-damage-inducible proteins-interacting protein 1	Q8TAE8|G45IP_HUMAN	**1,21**	0,96							
Growth hormone inducible transmembrane protein	Q5VT94|Q5VT94_HUMAN	1,08<br>0,91	1,01<br>1,03	0,82						
Growth-inhibiting protein 17	Q2TU34|Q2TU34_HUMAN	1,21<br>**1,62**	**1,68**<br>**1,79**	**1,69**	**0,86**<br>0,83	**0,94**<br>**0,91**	**0,94**<br>**0,88**	**1,64**<br>0,80	**3,46**<br>**3,72**	**10,37**<br>**5,75**
GrpE protein homolog 1, mitochondrial precursor	Q9HAV7|GRPE1_HUMAN	**0,81**<br>0,98	**0,84**<br>1,05	**0,60**	**1,69**	**1,44**	**1,90**			
GSPT1 protein	Q96GF2|Q96GF2_HUMAN				0,97	1,12	1,03			
GSTK1 protein	Q6P4H0|Q6P4H0_HUMAN	0,93	0,94	**0,68**						

GSTK1 protein (Fragment)	Q2NLC8|Q2NLC8_HUMAN				**1,53**	1,23	**1,16**			
GSTZ1 protein	Q6IB17|Q6IB17_HUMAN	0,84<br>1,10	0,90<br>0,98	0,89						
GTF2F1 protein	Q6IBK5|Q6IBK5_HUMAN				0,94	0,76	0,65			
GTP:AMP phosphotransferase mitochondrial	Q9UIJ7|KAD3_HUMAN	**0,84**<br>1,05	**0,75**<br>**0,88**	**0,52**	1,63	1,23	1,39			
GTPase activating protein (SH3 domain) binding protein 1	Q32P45|Q32P45_HUMAN							1,03	0,62	0,34
GTPase IMAP family member 1	Q8WWP7|GIMA1_HUMAN	0,93<br>0,95	1,03<br>**1,18**	0,91						
GTPase IMAP family member 4	Q9NUV9|GIMA4_HUMAN				**0,74**<br>0,00	0,95<br>0,00	0,94<br>0,00			
GTPase IMAP family member 8	Q8ND71|Q8ND71_HUMAN							0,00	0,00	0,00
GTPase KRas precursor	P01116|RASK_HUMAN	1,03	1,06							
GTP-binding nuclear protein Ran	P62826|RAN_HUMAN				**0,80**<br>0,94	1,09<br>1,02	0,96<br>1,04			
GTP-binding protein era homolog	O75616|ERAL_HUMAN	1,03<br>0,86	0,63<br>**0,86**	0,69						
GTP-binding protein SAR1a	Q9NR31|SAR1A_HUMAN	0,60<br>1,06	1,62<br>**1,57**	**3,51**	**0,79**<br>**0,81**	0,94<br>1,16	**0,68**<br>0,83			
GTP-binding protein SAR1b	Q9Y6B6|SAR1B_HUMAN	1,22	**1,27**	1,76	0,82	0,80	0,82			
Guanine nucleotide binding protein (G protein), alpha inhibiting activity polypeptide 3	Q5TZX1|Q5TZX1_HUMAN	1,04<br>1,19	1,04<br>1,16	1,02						
Guanine nucleotide binding protein (G protein), beta polypeptide 2-like 1 variant (Fragment)	Q53HU2|Q53HU2_HUMAN				**1,77**	**1,29**	**2,20**			
Guanine nucleotide binding protein (G protein), q polypeptide	Q6NT27|Q6NT27_HUMAN	1,19	1,16							
Guanine nucleotide-binding protein alpha-12 subunit	Q03113|GNA12_HUMAN	1,08	1,05							
Guanine nucleotide-binding protein alpha-13 subunit	Q14344|GNA13_HUMAN	**1,25**<br>1,08	**0,86**<br>0,94	1,09	1,91	0,94	1,25			
Guanine nucleotide-binding protein G(i), alpha-1 subunit	P63096|GNAI1_HUMAN				**1,47**	**0,89**	1,05			
Guanine nucleotide-binding protein G(i), alpha-2 subunit	P04899|GNAI2_HUMAN	1,62<br>1,00	1,33<br>0,96	1,60	0,00	0,00	0,00			
Guanine nucleotide-binding protein G(I)/G(S)/G(O) subunit gamma-10 precursor	P50151|GBG10_HUMAN	1,11	1,00					0,00<br>0,00	0,00<br>0,00	0,00<br>0,00
Guanine nucleotide-binding protein G(I)/G(S)/G(O) subunit gamma-5 precursor	P63218|GBG5_HUMAN	**1,30**<br>0,99	1,23<br>**0,80**	1,13	**1,98**<br>**1,58**	1,03<br>0,89	**1,42**<br>0,99	**1,58**<br>**1,83**	**2,01**<br>1,43	**2,52**<br>**2,82**
Guanine nucleotide-binding protein G(I)/G(S)/G(T) subunit beta-1	P62873|GBB1_HUMAN	1,33<br>1,06	1,08<br>1,12	**1,10**				**1,72**<br>0,55	1,22<br>**1,24**	**3,52**<br>1,53
Guanine nucleotide-binding protein G(s) subunit alpha isoforms XLas	Q5JWF2|GNAS1_HUMAN							1,40	0,84	1,47
GYG1 protein (Fragment)	Q8N5Y3|Q8N5Y3_HUMAN				0,00	0,00	0,00			
H/ACA ribonucleoprotein complex subunit 1	Q9NY12|NOLA1_HUMAN							**1,20**<br>1,13	**0,82**<br>0,83	**0,56**<br>**0,73**
H/ACA ribonucleoprotein complex subunit 2	Q9NX24|NOLA2_HUMAN							0,81<br>0,71	**0,56**<br>1,36	**0,46**<br>0,66
H/ACA ribonucleoprotein complex subunit 3	Q9NPE3|NOLA3_HUMAN							**0,85**<br>1,11	0,93<br>**0,53**	**0,51**<br>**0,60**
H2A histone family member J	Q9NV63|Q9NV63_HUMAN							0,00<br>0,00	0,00<br>0,00	0,00<br>0,00
H2A histone family, member J	Q9BTM1|Q9BTM1_HUMAN							0,00<br>0,00	0,00<br>0,00	0,00<br>0,00
HASCT1	Q9P2X2|Q9P2X2_HUMAN	1,33	**1,49**							
HBeAg-binding protein 2 binding protein A	Q1HDL3|Q1HDL3_HUMAN	0,99	0,91							
HBS1-like protein	Q9Y450|HBS1L_HUMAN				0,61	0,72	0,52			
HCLS1 protein	Q6IBK9|Q6IBK9_HUMAN	1,10<br>1,72	1,03<br>1,87	1,61	0,91<br>0,66	**0,85**<br>0,68	**0,71**<br>0,59	**0,67**<br>**1,61**	**0,15**<br>**0,38**	**0,11**<br>**0,37**
HCLS1-associated protein X-1	O00165|HAX1_HUMAN	0,88	0,84							
Hcp beta-lactamase-like protein C1orf163	Q96BR5|CA163_HUMAN	0,69<br>0,98	0,44<br>0,94	**0,38**						
Heat shock 70 kDa protein 1	P08107|HSP71_HUMAN	1,01	1,14	**2,41**	0,94<br>0,96	**1,11**<br>**1,15**	1,12<br>0,92	**1,53**<br>0,91	1,04<br>1,14	1,04<br>0,96
Heat shock 70kDa protein 1A variant (Fragment)	Q59EJ3|Q59EJ3_HUMAN	**1,19**	1,11							
Heat shock 70kDa protein 4	Q2TAL4|Q2TAL4_HUMAN				**0,84**<br>1,04	0,98<br>1,14	1,05<br>1,15			
Heat shock 70kDa protein 9B	Q1HB43|Q1HB43_HUMAN	**0,90**<br>0,98	**0,81**<br>0,99	**0,69**	**1,71**	**1,41**	**1,83**	**0,72**	**2,24**	**2,99**
Heat shock 70kDa protein 9B variant (Fragment)	Q53H23|Q53H23_HUMAN							0,91	**2,35**	**4,41**
Heat shock cognate 71 kDa protein	P11142|HSP7C_HUMAN	0,89<br>1,04	0,98<br>1,03	**1,62**	0,94<br>1,00	1,09<br>**1,14**	0,98<br>1,02	**1,66**<br>1,05	**0,77**<br>**0,87**	**0,83**<br>**0,71**
Heat shock protein 105 kDa	Q92598|HS105_HUMAN				1,07<br>0,97	1,23<br>1,13	1,21<br>0,94			
Heat shock protein 75 kDa, mitochondrial precursor	Q12931|TRAP1_HUMAN	1,00	1,02							
Heat shock protein 90Ad	Q58FG1|Q58FG1_HUMAN				0,00	0,00	0,00			
Heat shock protein beta-1	P04792|HSPB1_HUMAN	1,87<br>1,17	**5,16**<br>4,11	**13,91**	0,83	**0,52**	**0,41**	3,43	**9,73**	**5,05**
Heat shock protein HSP 90-alpha	P07900|HS90A_HUMAN	1,12<br>1,14	**1,45**<br>1,48	**1,85**	0,92<br>0,93	0,97<br>0,97	**1,12**<br>1,02	1,61	2,19	4,68
Heat shock protein HSP 90-beta	P08238|HS90B_HUMAN	**1,33**<br>1,12	**2,35**<br>**1,68**	**5,64**	**0,92**<br>0,94	1,04<br>1,00	1,05<br>1,05	1,88	2,85	4,71
Hemagglutinin precursor	P19106|HEMA_I72A2	**3,00**<br>**1,86**	**7,29**<br>**7,58**	**7,18**	**1,87**<br>2,18	**7,29**<br>5,35	**9,31**<br>7,90	**6,49**<br>**6,34**	**4,01**<br>**4,87**	**5,97**<br>**5,60**
Hematological and neurological expressed 1-like protein	Q9H910|HN1L_HUMAN				0,98	0,77	0,66			
Hematological and neurological expressed 1 protein	Q9UK76|HN1_HUMAN				1,16	0,92	0,78			
Heme oxygenase (Decycling) 1	Q96DI8|Q96DI8_HUMAN	1,30	0,95		0,00	0,00	0,00	0,00	0,00	0,00
Heme oxygenase (Decyclizing) 2 variant (Fragment)	Q53HF1|Q53HF1_HUMAN				1,58	0,94	0,79			
Heme-binding protein 1	Q9NRV9|HEBP1_HUMAN	1,00	**0,84**		0,95<br>1,00	1,05<br>1,16	1,05<br>0,96			
Hemopexin precursor	P02790|HEMO_HUMAN	1,79	1,79	**3,85**						
Heparan sulfate 2-O-sulfotransferase 1	Q7LGA3|HS2ST_HUMAN	1,10<br>1,03	**0,55**<br>**0,83**	0,53				1,33<br>1,76	1,11<br>1,36	1,48<br>1,96
Heparin-binding protein HBp15	Q7Z4W8|Q7Z4W8_HUMAN	0,92	1,16	1,37	1,24<br>0,97	0,79<br>0,79	1,09<br>0,89	1,18	**2,06**	**2,30**
Hepatoma-derived growth factor	P51858|HDGF_HUMAN				0,92<br>0,66	1,17<br>**0,85**	0,94<br>**0,75**	0,00	0,00	0,00
Hepatoma-derived growth factor-related protein 2	Q7Z4V5|HDGR2_HUMAN				0,00	0,00	0,00	1,06<br>**1,36**	**1,56**<br>**1,72**	1,39<br>**1,59**
Hepatopoietin	Q9UK40|Q9UK40_HUMAN									

		1,06	0,70							
Hepatopoietin PCn127	Q1AHP8|Q1AHP8_HUMAN							1,06	**3,19**	**2,69**
					0,79	1,03	**0,80**			
Heterochromatin protein 1, binding protein 3	Q5SSJ5|Q5SSJ5_HUMAN							1,07	**0,46**	**0,44**
					0,75	0,71	0,43	**0,79**	**0,69**	**0,66**
Heterogeneous nuclear ribonucleoprotein A1	P09651|ROA1_HUMAN	0,57	**1,23**	**2,02**	1,29	**2,42**	**2,02**	**0,80**	**0,24**	**0,14**
		0,95	1,53		0,89	1,58	**1,49**	0,97	**0,28**	**0,29**
Heterogeneous nuclear ribonucleoprotein A3	P51991|ROA3_HUMAN	**0,80**	**1,58**	**2,33**				0,93	**0,32**	**0,19**
		**0,69**	**1,46**		1,19	**3,18**	2,08	0,87	**0,37**	**0,27**
Heterogeneous nuclear ribonucleoprotein AB isoform a variant (Fragment)	Q53F64|Q53F64_HUMAN				1,25	**2,35**	**1,95**	0,99	**0,28**	**0,21**
					0,94	**1,76**	1,31	1,05	**0,36**	**0,33**
Heterogeneous nuclear ribonucleoprotein C-like 1	O60812|HNRCL_HUMAN									
								0,00	0,00	0,00
Heterogeneous nuclear ribonucleoprotein D0	Q14103|HNRPD_HUMAN							0,93	0,39	0,33
Heterogeneous nuclear ribonucleoprotein D-like	O14979|HNRDL_HUMAN							0,93	**0,28**	**0,20**
								0,94	**0,50**	**0,33**
Heterogeneous nuclear ribonucleoprotein F	P52597|HNRPF_HUMAN									
					1,26	1,53	1,09	**0,58**	**0,25**	**0,22**
Heterogeneous nuclear ribonucleoprotein G	P38159|HNRPG_HUMAN							**0,77**	**0,35**	**0,15**
								0,96	**0,45**	**0,36**
Heterogeneous nuclear ribonucleoprotein H	P31943|HNRH1_HUMAN	1,20	**3,45**	**5,85**	**1,99**	**1,66**	1,18	**0,79**	**0,18**	**0,15**
		1,00	**2,57**		**1,51**	1,45	1,01	**0,61**	**0,11**	**0,16**
Heterogeneous nuclear ribonucleoprotein H2	P55795|HNRH2_HUMAN									
								**0,68**	**0,19**	**0,20**
Heterogeneous nuclear ribonucleoprotein H3 isoform a variant (Fragment)	Q53F48|Q53F48_HUMAN							**0,72**	**0,17**	**0,14**
								**1,44**	**0,38**	**0,38**
Heterogeneous nuclear ribonucleoprotein K	P61978|HNRPK_HUMAN	1,38	**3,24**	**5,29**	**1,21**	**1,37**	1,08			
		1,04	1,67		0,95	**1,15**	0,92			
	Q5EC54|Q5EC54_HUMAN							1,03	**0,26**	**0,20**
Heterogeneous nuclear ribonucleoprotein K transcript variant								0,96	**0,40**	**0,35**
Heterogeneous nuclear ribonucleoprotein M	P52272|HNRPM_HUMAN				**1,32**	**1,54**	1,09	**0,65**	**0,18**	**0,16**
					1,22	1,33	0,93	**0,92**	**0,45**	**0,35**
Heterogeneous nuclear ribonucleoprotein Q	O60506|HNRPQ_HUMAN	0,76	0,86	1,49	**1,34**	**1,58**	1,27	0,91	0,37	0,30
								0,84	**0,46**	**0,45**
Heterogeneous nuclear ribonucleoprotein U	Q00839|HNRPU_HUMAN	0,90	1,40	**2,57**				1,23	0,21	0,15
								0,84	**0,34**	0,25
Heterogeneous nuclear ribonucleoprotein U-like protein 2	Q1KMD3|HNRL2_HUMAN				0,00	0,00	0,00	0,88	**0,31**	**0,32**
								**0,31**	**0,25**	**0,24**
Heterogeneous nuclear ribonucleoproteins A2/B1	P22626|ROA2_HUMAN	**0,79**	1,67	**3,41**	**1,48**	**2,96**	**1,86**	0,95	**0,24**	**0,12**
		0,85	1,77		1,19	2,87	**1,80**	1,04	**0,32**	**0,26**
Heterogeneous nuclear ribonucleoproteins C1/C2	P07910|HNRPC_HUMAN	0,87	1,40	**2,08**	1,14	**4,87**	**2,97**	**0,57**	**0,25**	**0,14**
					**1,80**	4,62	**2,70**	**0,73**	**0,39**	**0,32**
HEXA protein (Fragment)	Q9BVJ8|Q9BVJ8_HUMAN	0,88	**1,29**	0,97						
Hexokinase 1 isoform HKI variant (Fragment)	Q59FD4|Q59FD4_HUMAN	**1,38**	**1,96**	**2,69**	**1,40**	1,03	**0,86**	**1,14**	**1,48**	**4,46**
					1,59	1,05	0,90	**0,35**	**1,24**	**2,13**
Hexokinase 3	Q8N1E7|Q8N1E7_HUMAN				**0,74**	0,95	**0,84**			
Hexokinase 3 variant (Fragment)	Q59H08|Q59H08_HUMAN	0,95	0,88	1,05						
		1,06	1,23		0,92	1,06	1,03			
Hexokinase-2	P52789|HXK2_HUMAN				**1,20**	0,84	0,74			
High affinity immunoglobulin epsilon receptor subunit gamma precursor	P30273|FCERG_HUMAN	1,05	0,83	1,28	1,00	0,89	1,22	**1,66**	**2,22**	**2,23**
		1,54	1,46		**1,81**	0,92	1,24	1,75	1,66	2,12
	P12314|FCGR1_HUMAN									
High affinity immunoglobulin gamma Fc receptor 1 precursor		0,00	0,00							
High mobility group AT-hook 1	Q5T6U8|Q5T6U8_HUMAN							1,19		0,41
High mobility group AT-hook 1	Q5T6U9|Q5T6U9_HUMAN				1,10	**2,62**	**2,06**	1,01	**0,41**	**0,40**
					1,11	**3,40**	**4,45**	**0,86**	0,89	0,95
High mobility group nucleosome-binding domain-containing protein 3	Q15651|HMGN3_HUMAN							0,87	**0,30**	**0,27**
								1,19	0,79	**0,75**
High mobility group nucleosome-binding domain-containing protein 4	O00479|HMGN4_HUMAN							0,97	**0,62**	**0,53**
					0,46	**2,02**	2,66	1,15	**1,42**	**1,81**
High mobility group protein B3	O15347|HMGB3_HUMAN							1,01	**0,53**	**0,45**
					0,76	1,04	0,74	1,20	0,96	0,94
High-mobility group box 1	Q5T7C3|Q5T7C3_HUMAN				**0,79**	1,04	**0,77**	1,04	**1,57**	**1,40**
								**1,14**	**2,00**	**2,28**
High-mobility group box 2	Q5U071|Q5U071_HUMAN				**0,82**	0,90	**0,68**	**0,85**	**0,63**	**0,44**
		**1,21**	1,00		**0,69**	0,98	**0,71**	1,00	1,02	1,02
High-mobility group nucleosome binding domain 1	Q3B790|Q3B790_HUMAN									
		0,95	0,86							
Hippocalcin	Q5U068|Q5U068_HUMAN									
								1,39	3,33	3,46
HIST1H2AB protein	P04908|H2A1B_HUMAN							0,00	0,00	0,00
								0,00	0,00	0,00
Histamine N-methyltransferase	P50135|HNMT_HUMAN				0,00	0,00	0,00			
					0,80	0,94	0,82			
Histidine triad nucleotide-binding protein 1	P49773|HINT1_HUMAN				**0,82**	0,90	0,80			
Histidine triad nucleotide-binding protein 2	Q9BX68|HINT2_HUMAN	0,97	0,84	0,80						
		1,10	0,91							
Histidyl-tRNA synthetase	Q52NV4|Q52NV4_HUMAN				**0,81**	0,90	0,89			
Histidyl-tRNA synthetase, cytoplasmic	P12081|SYHC_HUMAN									
					0,93	1,04	1,17			
Histone cluster 1, H1e	Q4VB24|Q4VB24_HUMAN	0,86	1,10	**2,98**						
Histone cluster 2, H3, pseudogene 2	Q5TEC6|Q5TEC6_HUMAN							**0,83**	**0,64**	**0,66**
Histone deacetylase 1	Q6IT96|Q6IT96_HUMAN									
								**0,41**	**0,38**	**0,30**
Histone deacetylase 2	Q92769|HDAC2_HUMAN							1,27	0,43	0,17
Histone deacetylase complex subunit SAP18	O00422|SAP18_HUMAN							**0,83**	**0,55**	**0,25**
								1,02	**0,60**	**0,48**
Histone H1,0	P07305|H10_HUMAN							**0,88**	**0,57**	**0,60**
					1,48	**3,03**	**3,12**	1,17	1,19	**1,76**
Histone H1,1	Q02539|H11_HUMAN							0,81	**0,50**	**0,53**
								**1,28**	1,10	1,56
Histone H1,2	P16403|H12_HUMAN	0,47	1,06	**3,64**	1,21	**7,22**	**11,72**	**0,86**	**0,64**	**0,62**
		**0,57**	**0,61**		1,31	**8,11**	19,88	1,04	**1,34**	**1,85**
Histone H1,3	P16402|H13_HUMAN				0,95	**7,17**	**12,25**	**0,85**	**0,60**	**0,55**
					1,35	7,28	11,31	1,01	**1,30**	**1,75**
Histone H1,4	P10412|H14_HUMAN				1,45	**8,44**	**13,82**	**0,94**	**0,56**	**0,53**
					1,46	**5,52**	**10,64**			
Histone H1,5	P16401|H15_HUMAN	**0,73**	1,05	**2,86**	0,86	**5,83**	**9,25**	**0,83**	**0,48**	**0,49**
		**0,55**	**0,47**		**1,25**	4,98	10,01	**1,10**	**1,07**	**1,63**
Histone H1x	Q92522|H1X_HUMAN				1,09	**1,84**	2,09	0,91	**0,67**	**0,60**
					1,08	1,67	1,67	**1,10**	**1,20**	**1,67**
Histone H2A	A2RUA4|A2RUA4_HUMAN							0,00	0,00	0,00
								1,33	**1,21**	1,49
Histone H2A type 1-C	Q93077|H2A1C_HUMAN							0,00	0,00	0,00
								0,00	0,00	0,00
Histone H2A type 1-D	P20671|H2A1D_HUMAN							0,00	0,00	0,00
								0,00	0,00	0,00
Histone H2A type 1-H	Q96KK5|H2A1H_HUMAN							0,00	0,00	0,00

Histone H2A type 1-J	Q99878|H2A1J_HUMAN	 **0,48**	 **0,60**					0,00<br>0,00	0,00<br>0,00	0,00<br>0,00
Histone H2A type 2-A	Q6FI13|H2A2A_HUMAN							0,00<br>0,00	0,00<br>0,00	0,00<br>0,00
Histone H2A type 3	Q7L7L0|H2A3_HUMAN							0,00<br>0,00	0,00<br>0,00	0,00<br>0,00
Histone H2A,x	P16104|H2AX_HUMAN	**1,29**	**0,41**	**1,27**	0,72<br>**1,51**	**9,55**<br>5,34	**15,79**<br>8,56	0,84<br>1,08	**0,70**<br>1,09	0,78<br>**1,59**
Histone H2A,Z	P0C0S5|H2AZ_HUMAN							 1,07	 1,07	 **1,46**
Histone H2AV	Q71UI9|H2AV_HUMAN	1,11<br>0,00	0,65<br>0,00	1,15	0,99<br>1,08	2,54<br>**10,37**	2,47<br>**20,33**	0,96	**0,62**	**0,70**
Histone H2B type 1-H	Q93079|H2B1H_HUMAN							1,11<br>1,19	0,93<br>1,27	0,93<br>1,54
Histone H2B type 1-J	P06899|H2B1J_HUMAN							 1,07	 1,11	 1,53
Histone H2B type 1-L	Q99880|H2B1L_HUMAN	 **0,57**	 **0,45**							
Histone H2B type 1-M	Q99879|H2B1M_HUMAN	1,05	**0,33**	**1,26**	1,76<br>**1,93**	**15,16**<br>10,31	**25,87**<br>18,48			
Histone H2B type 2-C	Q6DN03|H2B2C_HUMAN	 **0,46**	 **0,46**							
Histone H2B type 2-E	Q16778|H2B2E_HUMAN							0,91	0,83	0,73
Histone H2B type 3-B	Q8N257|H2B3B_HUMAN							0,76<br>**1,29**	0,65<br>1,00	0,61<br>**1,38**
Histone H3,1t	Q16695|H31T_HUMAN				 **1,41**	 7,25	 13,78	 0,99	 **0,66**	 1,00
Histone H3,2	Q71DI3|H32_HUMAN	1,04<br>**0,58**	**0,41**<br>**0,51**	**1,19**	1,06	**7,55**	**12,26**			
Histone H3,3	P84243|H33_HUMAN							**0,89**<br>0,99	**0,69**<br>0,64	**0,63**<br>0,96
Histone H4	P62805|H4_HUMAN	**1,15**<br>**0,52**	**0,38**<br>**0,44**	**1,21**	1,19<br>1,01	**8,70**<br>4,93	**11,82**<br>8,23	**0,88**<br>1,68	**0,71**<br>0,97	**0,69**<br>1,50
Histone H4-like protein type G	Q99525|H4G_HUMAN	 0,00	 0,00		 0,00	 0,00	 0,00	1,24<br>0,00	0,79<br>0,00	**0,83**<br>0,00
Histone-binding protein RBBP4	Q09028|RBBP4_HUMAN				1,69<br>1,13	2,73<br>**1,67**	2,53<br>1,35	1,02	**0,41**	**0,44**
HIV Tat-specific factor 1	O43719|HTSF1_HUMAN							1,03<br>1,10	0,41<br>0,67	0,37<br>0,40
HLA B44 (Fragment)	A9JPE8|A9JPE8_HUMAN				 0,00	 0,00	 0,00			
HLA class II histocompatibility antigen gamma chain	P04233|HG2A_HUMAN	**0,86**<br>**0,88**	**0,38**<br>**0,81**	**0,40**				0,90	**3,29**	**4,24**
HLA class II histocompatibility antigen, DR alpha chain precursor	P01903|2DRA_HUMAN							**1,52**	**2,23**	**2,94**
HLA class II histocompatibility antigen, DRB1-9 beta chain precursor	Q9TQE0|2B19_HUMAN	1,04	0,80	0,81						
HLA-B associated transcript 1	Q5STU3|Q5STU3_HUMAN				**1,61**	**2,34**	**1,78**			
HLA-B associated transcript 5	B0UXB6|B0UXB6_HUMAN	0,86<br>0,89	1,04<br>1,14	1,28						
HLA-DMB protein (Fragment)	Q6LEU6|Q6LEU6_HUMAN	1,17	1,05	0,95	1,55	0,58	0,86	 0,98	4,74<br>**2,32**	7,86<br>**3,73**
HLA-DPA1 protein	Q95HB9|Q95HB9_HUMAN	 0,00	 0,00							
HLA-DPB2 protein	Q8WM95|Q8WM95_HUMAN			2,93						
HLA-DQA1 protein	Q6DKG8|Q6DKG8_HUMAN	 0,96	 0,94							
HLA-DRB1*13 variant (Fragment)	Q53IG1|Q53IG1_HUMAN	1,08	0,82	0,98						
HLA-drb5 (Fragment)	Q29703|Q29703_HUMAN	 1,00	 **0,84**							
HMGCL protein	Q6IBC0|Q6IBC0_HUMAN	0,90<br>**0,91**	**0,86**<br>**0,88**	**0,64**						
HMOX2 protein	Q6IBP2|Q6IBP2_HUMAN	0,86<br>1,06	1,03<br>1,28	0,96	0,00	0,00	0,00		8,37	10,96
HNRPA0 protein	Q6IB18|Q6IB18_HUMAN							1,02<br>**0,63**	**0,41**<br>**0,47**	**0,20**<br>**0,32**
HNRPL protein	Q6NTA2|Q6NTA2_HUMAN				1,98	**4,51**	2,78	1,05<br>**0,39**	**0,27**<br>**0,26**	**0,24**<br>**0,22**
HNRPR protein	Q9BV64|Q9BV64_HUMAN	 1,10	 1,23					0,72<br>**0,76**	**0,18**<br>**0,33**	**0,13**<br>**0,32**
HNRPU protein	Q96BA7|Q96BA7_HUMAN				1,06<br>1,04	**2,71**<br>**2,35**	**1,43**<br>**1,78**	**1,42**<br>**0,39**	**0,35**<br>**0,45**	**0,21**<br>**0,21**
HP protein	Q6NSB4|Q6NSB4_HUMAN	0,00	0,00	0,00	**2,06**	**2,03**	**3,70**			
HP protein	Q0VAC5|Q0VAC5_HUMAN	**1,49**	**1,63**	**3,68**						
HPCAL1 protein	Q6FGY1|Q6FGY1_HUMAN				**1,39**<br>0,90	0,99<br>0,76	1,01<br>0,74			
HSD-33	Q6W898|Q6W898_HUMAN	1,08<br>0,98	1,35<br>1,06	1,02	1,38	0,61	1,20			
HSPA9 protein (Fragment)	Q8N1C8|Q8N1C8_HUMAN				 1,84	 1,28	 1,42			
HSPC027	Q9Y6E3|Q9Y6E3_HUMAN	0,92	1,34	3,64	1,08<br>0,84	1,14<br>0,87	0,94<br>0,79			
HSPC151	Q9P012|Q9P012_HUMAN							0,93	1,03	1,29
HSPC185	Q9P0T8|Q9P0T8_HUMAN							1,05	**0,47**	0,40
HSPE1 protein (Fragment)	Q53X54|Q53X54_HUMAN	**0,87**<br>1,00	**0,82**<br>1,00	**0,63**	**1,85**<br>1,54	**1,71**<br>**1,26**	**2,97**<br>1,84	1,11<br>**1,22**	**2,77**<br>**2,44**	**2,83**<br>**2,87**
Huntingtin	P42858|HD_HUMAN				 1,09	 1,86	 1,36			
Huntingtin-interacting protein K	Q9NX55|HYPK_HUMAN				 0,58	 0,79	 0,76			
Hydrocephalus-inducing protein homolog	Q4G0P3|HYDIN_HUMAN				 0,00	 0,00	 0,00			
Hydrogen voltage-gated channel 1	Q96D96|Q96D96_HUMAN	0,75	0,52	0,60	 0,00	 0,00	 0,00	0,00	0,00	0,00
Hydroxyacyl-coenzyme A dehydrogenase, mitochondrial precursor	Q16836|HCDH_HUMAN	1,03<br>0,98	**0,87**<br>0,96	**0,63**	1,27<br>**1,65**	1,38<br>1,20	1,67<br>**1,55**	 0,60	 0,48	 0,80
Hydroxyacylglutathione hydrolase	Q16775|GLO2_HUMAN	1,97<br>0,82	1,37<br>0,75		**0,79**<br>0,97	0,99<br>0,68	0,89<br>0,64			
Hydroxyprostaglandin dehydrogenase 15-(NAD)	Q06F08|Q06F08_HUMAN				 0,00	 0,00	 0,00			
Hydroxysteroid 11-beta-dehydrogenase 1-like protein precursor	Q7Z5J1|DHI1L_HUMAN	 0,00	 0,00							
Hydroxysteroid dehydrogenase-like protein 2	Q6YN16|HSDL2_HUMAN	0,93<br>1,03	**0,78**<br>1,01	**0,63**						
Hypoxia up-regulated protein 1 precursor	Q9Y4L1|HYOU1_HUMAN	0,98<br>0,97	1,09<br>1,06	1,08	**1,83**<br>1,93	1,06<br>1,33	**1,49**<br>**1,91**	0,90<br>0,84	**2,79**<br>**3,46**	**5,71**<br>**4,86**
HZGJ	Q86YQ0|Q86YQ0_HUMAN				0,91<br>0,00	0,75<br>0,00	**0,72**<br>0,00			
IDH1 protein (Fragment)	Q6FHQ6|Q6FHQ6_HUMAN				**0,90**	0,94	0,92			
IKIP2	Q70UQ0|Q70UQ0_HUMAN							0,74	2,48	3,31

					1,33	0,84	0,60			
Immature colon carcinoma transcript 1 protein precursor	Q14197|ICT1_HUMAN	1,01<br>0,98	1,00<br>0,99	0,94						
Immediate early response 3-interacting protein 1	Q9Y5U9|IR3IP_HUMAN	0,81<br>1,04	1,09<br>0,98	0,96					5,40	7,97
IMMT protein	Q3B7X4|Q3B7X4_HUMAN	**0,64**<br>0,00	**0,80**<br>0,00	0,96						
Immunity-related GTPase family Q protein	Q8WZA9|IRGQ_HUMAN				0,60	0,67	**0,55**			
Immunoglobulin superfamily member 2 precursor	Q93033|IGSF2_HUMAN	1,01	0,95							
Immunoglobulin superfamily member 6 precursor	O95976|IGSF6_HUMAN	0,96<br>0,76	**0,49**<br>0,66	**0,55**						
Immunoglobulin-binding protein 1	P78318|IGBP1_HUMAN				0,00<br>0,54	0,00<br>**0,62**	0,00<br>**0,42**			
Induced by contact to basement membrane 1 protein	Q5TEJ8|ICB1_HUMAN				0,94	0,84	0,60			
Inhibitor-1 of protein phosphatase-2A	Q5J8L8|Q5J8L8_HUMAN				0,85	0,90	**0,60**			
Inner membrane protein COX18, mitochondrial precursor	Q8N8Q8|COX18_HUMAN	1,04	0,95							
Inner membrane protein OXA1L, mitochondrial precursor	Q15070|OXA1L_HUMAN	0,88<br>0,95	**0,82**<br>**0,83**	0,85						
Inorganic pyrophosphatase	Q15181|IPYR_HUMAN				0,99<br>0,96	1,04<br>0,94	0,86<br>0,96			
Inorganic pyrophosphatase 2, mitochondrial precursor	Q9H2U2|IPYR2_HUMAN	1,12<br>1,03	1,13<br>0,97	0,86	1,39	1,34	**1,68**			
Inosine-5'-monophosphate dehydrogenase 2	P12268|IMDH2_HUMAN	1,41	3,51	**7,50**	**0,78**<br>0,85	**0,75**<br>0,94	**0,54**<br>0,69			
Inositol 1,4,5-trisphosphate receptor type 1	Q14643|ITPR1_HUMAN	0,90<br>0,98	0,98<br>1,03	0,94						
Inositol 1,4,5-trisphosphate receptor type 2	Q14571|ITPR2_HUMAN	0,99<br>0,93	0,99<br>0,99	1,07						
Inositol monophosphatase 3	Q9NX62|IMPA3_HUMAN	1,11<br>0,87	0,62<br>1,07	0,73						
Insulin-degrading enzyme	Q5T5N2|Q5T5N2_HUMAN				0,00	0,00	0,00			
Integrator complex subunit 8	Q75QN2|INT8_HUMAN							0,00	0,00	0,00
Integrin alpha-5 precursor	P08648|ITA5_HUMAN	**1,38**<br>**1,18**	1,10<br>**1,15**	**1,32**	1,64<br>2,29	1,12<br>1,15	0,84<br>**1,69**			
Integrin alpha-L precursor	P20701|ITAL_HUMAN	**1,37**<br>1,00	0,98<br>1,06	**1,29**	**2,23**<br>1,09	1,12<br>0,85	1,41<br>0,80			
Integrin alpha-M precursor	P11215|ITAM_HUMAN	0,95	1,02							
Integrin alpha-V precursor	P06756|ITAV_HUMAN	1,25<br>1,04	1,39<br>0,86	1,06						
Integrin alpha-X precursor	P20702|ITAX_HUMAN	**1,33**<br>1,04	1,02<br>**1,08**	1,12	**2,66**	1,57	**2,11**	3,82	2,56	7,56
Integrin beta (Fragment)	Q7Z3V1|Q7Z3V1_HUMAN	**1,10**	**1,13**							
Integrin beta (Fragment)	Q6PJ75|Q6PJ75_HUMAN	**1,21**<br>0,97	**0,89**<br>0,99	0,99	**1,73**<br>**1,60**	0,93<br>1,02	**1,27**<br>1,27	**2,28**	**3,24**	**4,47**
Integrin beta-2 precursor	P05107|ITB2_HUMAN				1,78	**0,91**	1,15	**1,92**	**3,38**	**4,40**
Integrin beta-3 precursor	P05106|ITB3_HUMAN	1,03<br>0,85	0,93<br>0,94	1,08						
Integrin, alpha 2b	Q17R67|Q17R67_HUMAN	0,00	0,00	0,00						
Integrin, alpha M	Q4VAK1|Q4VAK1_HUMAN	**1,50**	**1,12**	**1,33**	**1,75**<br>**2,09**	1,11<br>1,49	**1,33**<br>**1,69**			
Integrin, beta 1	Q8WUM6|Q8WUM6_HUMAN	1,29	1,07	**1,29**						
Integrin-linked protein kinase	Q13418|ILK_HUMAN	1,06	1,37	**2,88**	**0,85**<br>**0,81**	0,99<br>0,89	**0,80**<br>**0,75**			
Intercellular adhesion molecule 1	Q5NKV8|Q5NKV8_HUMAN	**1,65**	1,33	1,37						
Intercellular adhesion molecule 1 precursor	P05362|ICAM1_HUMAN	**1,33**	1,19							
Intercellular adhesion molecule 3	Q6PD68|Q6PD68_HUMAN	0,98	1,14							
Interferon (Alpha, beta and omega) receptor 2								0,00	0,00	0,00
Interferon gamma-inducible protein 30 preproprotein								0,00	0,00	0,00
Interferon, gamma-inducible protein 30	P13284|GILT_HUMAN	1,14	**1,37**	1,32	**2,17**<br>**1,18**	1,37<br>0,95	**2,27**<br>**1,25**	0,00	0,00	0,00
ISG15 ubiquitin-like modifier	P05161|UCRP_HUMAN	1,36	**3,55**	**5,68**	**2,39**	**4,77**	**4,79**			
Interferon-induced 35 kDa protein	P80217|IN35_HUMAN				1,50<br>0,00	1,19<br>0,00	1,22<br>0,00			
Interferon-induced GTP-binding protein Mx1	P20591|MX1_HUMAN				**1,73**<br>1,78	2,85<br>2,58	2,61<br>2,13			
Interferon-induced guanylate-binding protein 1	P32445|GBP1_HUMAN				1,11<br>1,29	**1,79**<br>1,95	**1,73**<br>1,76			
Interferon-induced protein with tetratricopeptide repeats 1	P09914|IFIT1_HUMAN	1,35	1,96	3,69	**1,76**<br>**2,75**	**2,45**<br>**4,89**	**2,22**<br>**4,03**			
Interferon-induced protein with tetratricopeptide repeats 2	Q8IZ03|Q8IZ03_HUMAN			**15,75**	0,00<br>11,98	0,00<br>**15,11**	0,00<br>10,74			
Interferon-induced protein with tetratricopeptide repeats 3	O14879|IFIT3_HUMAN		**6,74**	**17,70**	**2,99**	**4,57**	**3,72**			
Interleukin enhancer binding factor 2 variant (Fragment)	Q53FG3|Q53FG3_HUMAN							1,00<br>**0,71**	**0,35**<br>**0,37**	**0,27**<br>**0,30**
Interleukin enhancer-binding factor 3	Q12906|ILF3_HUMAN				1,16	**2,69**	**2,08**	**0,92**<br>0,86	**0,49**<br>**0,64**	**0,28**<br>**0,46**
Interleukin-1 receptor antagonist protein precursor	P18510|IL1RA_HUMAN	1,84	2,28	3,09	0,92<br>0,95	**1,21**<br>0,89	1,01<br>1,15			
Interleukin-18	B0YJ28|B0YJ28_HUMAN	0,00	0,00	0,00						
Interleukin-4 induced protein-1 variant 2	Q1WMJ3|Q1WMJ3_HUMAN	1,21<br>**1,24**	**1,65**<br>**1,16**	1,44				1,07	1,34	1,65
Inverted formin-2	Q27J81|INF2_HUMAN				1,10	0,80	1,48			
Iron regulatory protein 1	Q9HBB2|Q9HBB2_HUMAN				1,04	1,15	0,94			
Iron-sulfur cluster assembly 1 homolog, mitochondrial precursor	Q9BUE6|ISCA1_HUMAN	0,00	0,00							
Iron-sulfur cluster assembly enzyme ISCU, mitochondrial precursor	Q9H1K1|ISCU_HUMAN	**0,82**<br>1,02	0,95<br>1,06	1,12	1,40	1,23	1,24			
Iron-sulfur cluster scaffold protein Nfu	Q7Z5B2|Q7Z5B2_HUMAN	1,05	1,05							
Isobutyryl-CoA dehydrogenase, mitochondrial precursor	Q9UKU7|ACAD8_HUMAN	0,92	0,91							
Isocitrate dehydrogenase [NAD] subunit alpha, mitochondrial precursor	P50213|IDH3A_HUMAN	0,88<br>1,00	0,93<br>**0,94**	**0,74**	**2,00**<br>**1,70**	**1,60**<br>1,25	**2,57**<br>**1,68**			
Isocitrate dehydrogenase [NAD] subunit beta, mitochondrial precursor	O43837|IDH3B_HUMAN	**0,57**<br>1,01	0,78<br>0,93	**0,58**	1,98	1,15	2,09			
Isocitrate dehydrogenase [NAD] subunit gamma, mitochondrial precursor	P51553|IDH3G_HUMAN	0,86<br>**0,88**	1,03<br>1,00	0,90						

Isocitrate dehydrogenase [NADP], mitochondrial precursor	P48735|IDHP_HUMAN	1,05	0,92	**0,76**	**1,73**	**1,40**	**1,79**			
		0,94	**0,90**							
Isocitrate dehydrogenase 2 (NADP+), mitochondrial variant	Q53GL5|Q53GL5_HUMAN								3,06	7,63
(Fragment)					1,91	1,36	1,84	**0,62**	1,67	3,65
Isoleucyl-tRNA synthetase, mitochondrial precursor	Q9NSE4|SYIM_HUMAN	**0,87**	0,92	**0,79**						
		0,96	1,00							
Isopentenyl-diphosphate Delta-isomerase 1	Q13907|IDI1_HUMAN									
		0,83	0,90							
Isovaleryl Coenzyme A dehydrogenase	Q53XZ9|Q53XZ9_HUMAN									
		1,01	0,94		2,77	1,64	1,55			
Isovaleryl-CoA dehydrogenase, mitochondrial precursor	P26440|IVD_HUMAN	**0,81**	**0,75**	**0,66**						
IWS1 homolog	Q96ST2|IWS1_HUMAN							1,58	0,58	**0,28**
JTB protein	Q6IB19|Q6IB19_HUMAN									
		1,12	1,02							
Jun B proto-oncogene	Q5U079|Q5U079_HUMAN							0,45	0,08	**0,07**
Junctional adhesion molecule A precursor	Q9Y624|JAM1_HUMAN									
		1,18	0,98							
Kangai 1	Q7Z5N2|Q7Z5N2_HUMAN	1,41	0,74	0,91						
		0,76	1,03							
Kappa-actin	Q9BYX7|ACTK_HUMAN	0,96	0,80	1,72						
		0,79	1,39		0,61	0,69	0,49			
Karyopherin (Importin) beta 1	Q53XN2|Q53XN2_HUMAN			6,23	0,99	1,08	1,03	1,47	1,18	1,58
					1,05	1,16	1,26	0,49	1,43	1,14
KDEL motif-containing protein 2 precursor	Q7Z4H8|KDEL2_HUMAN	0,88	1,09	1,19						
		1,14	**1,31**							
Keratin 77	Q0IIN1|Q0IIN1_HUMAN									
					0,00	0,00	0,00			
Keratin, type I cytoskeletal 10	P13645|K1C10_HUMAN	**0,69**	**0,69**	**1,26**				**5,08**	2,06	**13,19**
		**1,17**	0,78		0,55	0,98	1,81	**3,10**	**3,90**	1,04
Keratin, type I cytoskeletal 9	P35527|K1C9_HUMAN	1,05	1,19	**5,34**				**7,40**	1,57	**8,37**
					0,98	0,90	1,17	**1,58**	**4,51**	**0,73**
Keratin, type II cytoskeletal 1	P04264|K2C1_HUMAN	**0,86**	0,89	**3,04**	**0,49**	**1,48**	**1,94**	**3,56**	**0,68**	**5,77**
		**1,34**	**0,67**		**0,20**	0,30	**0,59**	**2,41**	**5,12**	0,94
Keratin, type II cytoskeletal 1b	Q7Z794|K2C1B_HUMAN				0,00	0,00	0,00	0,00	0,00	0,00
Keratin, type II cytoskeletal 2 epidermal	P35908|K22E_HUMAN				1,45	2,52	2,05	**6,70**		**22,56**
								**2,05**	**2,74**	0,89
Keratin, type II cytoskeletal 6A	P02538|K2C6A_HUMAN	0,62	0,64	0,90						
Keratin, type II cytoskeletal 72	Q14CN4|K2C72_HUMAN	1,17	1,44	1,50						
Ketosamine-3-kinase	Q9HA64|KT3K_HUMAN				0,91	1,04	0,71			
KH domain-containing, RNA-binding, signal transduction-	Q07666|KHDR1_HUMAN							**1,18**	**0,29**	**0,16**
associated protein 1								**1,18**	**0,41**	**0,32**
KIAA0023 protein	Q15010|Q15010_HUMAN									
								**0,31**	0,69	**0,35**
KIAA0103 variant (Fragment)	Q53HG5|Q53HG5_HUMAN									
					0,00	0,00	0,00	1,28	**2,32**	**2,39**
KIAA0174 protein	Q3SYM4|Q3SYM4_HUMAN							1,16	**5,16**	**5,92**
								0,99	**2,68**	**2,52**
KIAA0391	Q8N5L5|Q8N5L5_HUMAN									
		**0,84**	0,94							
KIAA0564 protein (Fragment)	A3KMH1|A3KMH1_HUMAN	**0,82**	**0,81**	**0,80**						
		0,99	0,96							
KIAA1228 protein	A4D229|A4D229_HUMAN	0,91	1,17	0,94						
		0,96	1,18		0,65	0,94	0,76			
KIAA1835 protein (Fragment)	Q96JJ2|Q96JJ2_HUMAN									
					1,18	1,08	0,89			
Kinectin	Q86UP2|KTN1_HUMAN	0,94	**0,72**	**0,74**	0,00	0,00	0,00	0,90	1,66	1,45
		1,26	1,07		1,30	1,34	1,17	0,80	**3,01**	**3,60**
Kinesin family member 1B	Q4VXC3|Q4VXC3_HUMAN				1,03	1,06	1,22			
					0,00	0,00	0,00			
Kinesin light chain 1M	Q7RTQ4|Q7RTQ4_HUMAN									
					0,00	0,00	0,00			
Kinesin-1 heavy chain	P33176|KINH_HUMAN				0,75	1,45	1,40			
					0,95	1,07	0,94			
KTEL motif-containing protein 1 precursor	Q8NBL1|KTEL1_HUMAN							0,92	**1,54**	**1,72**
Kunitz-type protease inhibitor 1 precursor	O43278|SPIT1_HUMAN									
		1,59	0,79							
Kynureninase (L-kynurenine hydrolase) variant (Fragment)	Q53F63|Q53F63_HUMAN				0,93	1,08	1,09			
Kynurenine--oxoglutarate transaminase 3	Q6YP21|KAT3_HUMAN									
		0,97	1,13							
	Q9H9P8|L2HDH_HUMAN									
L-2-hydroxyglutarate dehydrogenase, mitochondrial precursor		1,13	1,04							
LAG1 longevity assurance homolog 2	Q96G23|LASS2_HUMAN	1,12	1,15	1,05						
		0,79	0,97							
LAG1 longevity assurance homolog 6	Q6ZMG9|LASS6_HUMAN									
		0,82	0,91							
Lamin A/C	P02545|LMNA_HUMAN				1,01	**3,20**	**3,41**			
		**0,48**	**0,77**		1,64	4,23	4,36	**0,89**	0,44	0,31
Lamina-associated polypeptide 2, isoforms beta/gamma	P42167|LAP2B_HUMAN							0,93	**0,67**	**0,35**
								**1,11**	**1,15**	**0,87**
Lamina-associated polypeptide 2, isoforms beta/gamma	Q59G12|Q59G12_HUMAN	0,00	0,00	0,00	1,10	1,07	1,12	0,00	0,00	0,00
variant (Fragment)										
Lamin-B receptor	Q14739|LBR_HUMAN	0,85	0,87	1,05				1,14	1,06	0,95
		**0,84**	**0,80**					0,72	1,26	1,05
Lamin-B1	P20700|LMNB1_HUMAN	**0,52**	**0,32**	**0,39**	2,07	**8,20**	**7,71**	**1,09**	**0,51**	**0,27**
		**0,71**	0,90					**0,54**	**0,40**	**0,26**
Lamin-B2	Q03252|LMNB2_HUMAN							1,04	**0,55**	**0,28**
		0,86	0,88					0,92	**0,68**	**0,51**
Lanosterol synthase	P48449|ERG7_HUMAN	1,04	1,02	0,94						
		1,00	1,06							
La-related protein 1	Q6PKG0|LARP1_HUMAN				0,00	0,00	0,00			
					0,77	0,59	0,38	0,85	0,94	0,82
Lectin, galactoside-binding, soluble, 3	Q6NVH9|Q6NVH9_HUMAN									
					0,87	0,86	0,78	**1,63**	1,17	0,98
Lectin, galactoside-binding, soluble, 8	Q5T3Q4|Q5T3Q4_HUMAN									
		0,00	0,00							
Lectin, mannose-binding, 1 variant (Fragment)	Q53FS4|Q53FS4_HUMAN	1,00	1,00	1,05						
		0,95	1,03							
LEM domain-containing protein 2	Q8NC56|LEMD2_HUMAN							**1,18**	0,75	**0,56**
		0,93	0,95					1,03	1,30	1,19
LETM1 domain-containing protein 1	Q6P1Q0|LTMD1_HUMAN									
		0,98	1,03							
Leucine zipper-EF-hand-containing transmembrane protein 1,	O95202|LETM1_HUMAN	**0,87**	**0,86**	**0,71**				0,74	**2,37**	**3,85**
mitochondrial precursor		0,99	**0,94**					0,74	**1,65**	**2,52**
Leucine-rich PPR motif-containing protein, mitochondrial	P42704|LPPRC_HUMAN				1,06	0,97	1,20			
precursor					2,08	1,16	1,67			
Leucine-rich repeat and calponin homology domain-containing	Q96II8|LRCH3_HUMAN									
protein 3 precursor		2,02	1,69							
Leucine-rich repeat flightless-interacting protein 1	Q32MZ4|LRRF1_HUMAN				**0,63**	1,31	1,10	1,80	1,32	0,97
					0,53	0,91	**0,76**			
Leucine-rich repeat-containing protein 25 precursor	Q8N386|LRC25_HUMAN	1,31	0,92	1,01						

		1,17	0,93							
Leucine-rich repeat-containing protein 59	Q96AG4|LRC59_HUMAN	**0,84**<br>1,00	0,95<br>**1,22**	**0,89**	**1,88**	0,82	1,01	**0,78**<br>0,97	**3,21**<br>**3,19**	**3,79**<br>**4,05**
Leucine-rich repeat-containing protein 8C	Q8TDW0|LRC8C_HUMAN	1,14<br>0,87	0,88<br>0,98	1,07						
Leucocyte antigen B	Q9UIP6|Q9UIP6_HUMAN	1,14	0,95	**0,83**						
Leucyl-cystinyl aminopeptidase	Q9UIQ6|LCAP_HUMAN	0,88<br>0,76	0,87<br>0,84	0,82						
Leukemia-associated protein (Fragment)	Q5MK14|Q5MK14_HUMAN	1,85	1,17							
Leukocyte antigen-DQ beta chain precursor	Q29884|Q29884_HUMAN	1,17	1,05	0,99						
Leukocyte common antigen precursor	P08575|CD45_HUMAN	**1,46**<br>**1,09**	1,11<br>**1,13**	**1,24**	**1,71**<br>**1,91**	1,13<br>1,29	1,12<br>**1,96**			
Leukocyte elastase inhibitor	P30740|ILEU_HUMAN				0,91	1,06	0,92			
Leukocyte elastase precursor	P08246|ELNE_HUMAN	0,98	0,99							
Leukocyte immunoglobulin-like receptor subfamily A member 1 precursor	O75019|LIRA1_HUMAN	0,85	0,88							
Leukocyte immunoglobulin-like receptor subfamily B member 3 precursor	O75022|LIRB3_HUMAN	1,04<br>0,96	0,66<br>0,91	0,79						
Leukocyte immunoglobulin-like receptor subfamily B member 4 precursor	Q8NHJ6|LIRB4_HUMAN	1,13<br>0,86	0,94<br>0,73	1,32	1,80	1,28	0,93			
Leukocyte surface antigen CD47 precursor	Q08722|CD47_HUMAN	**1,42**<br>1,07	1,18<br>**1,31**	**1,37**	1,65	0,81	1,25			
Leukocyte surface antigen CD53	P19397|CD53_HUMAN	**1,78**	1,39	**1,80**						
Leukosialin precursor	P16150|LEUK_HUMAN	**2,02**<br>**2,53**	**1,40**<br>**1,73**	**1,94**	**1,68**<br>**1,27**	1,09<br>0,87	1,20<br>1,05	1,25<br>1,90	2,05<br>3,75	2,62<br>4,89
Leydig cell tumor 10 kDa protein homolog	Q9UNZ5|L10K_HUMAN							0,94	**0,65**	**0,57**
LGALS3 protein (Fragment)	Q6FGL0|Q6FGL0_HUMAN							**1,74**	**1,57**	**1,39**
LIM and senescent cell antigen-like-containing domain protein 1	P48059|LIMS1_HUMAN				**0,93**<br>0,82	1,23<br>0,93	1,20<br>0,81			
LIM and SH3 domain protein 1	Q14847|LASP1_HUMAN				**0,77**<br>**0,72**	**0,86**<br>**0,72**	**0,74**<br>**0,65**	**1,23**<br>**1,94**	**0,55**<br>**0,53**	**0,52**<br>**0,47**
LIM-only protein FHL3	Q9P100|Q9P100_HUMAN				0,00	0,00	0,00			
Lipase maturation factor 2	Q9BU23|LMF2_HUMAN	0,93	1,10							
Lipoamide acyltransferase component of branched-chain alpha-keto acid dehydrogenase complex, mitochondrial precursor	P11182|ODB2_HUMAN	**0,90**	0,94							
Lipoma HMGIC fusion partner-like 2 protein	Q6ZUX7|LHPL2_HUMAN	0,84	0,46	0,29						
Liver carboxylesterase 1 precursor	P23141|EST1_HUMAN	**0,88**	1,03	0,93	**1,83**<br>**1,99**	**1,38**<br>**1,35**	**2,57**<br>**1,91**			
Liver histone H1e	A3R0T7|A3R0T7_HUMAN							1,05	1,04	**1,42**
L-lactate dehydrogenase	Q5U077|Q5U077_HUMAN	1,00<br>1,15	1,49<br>1,12	1,31	**0,88**<br>0,91	1,00<br>0,94	0,99<br>0,94			
L-lactate dehydrogenase A chain	P00338|LDHA_HUMAN	1,19<br>**1,22**	1,50<br>**1,72**	1,15	**0,85**<br>**0,92**	0,98<br>1,00	1,01<br>1,10			
LMNB1 protein (Fragment)	Q6DC98|Q6DC98_HUMAN				1,29	3,20	3,91			
Lon protease homolog, mitochondrial precursor	P36776|LONM_HUMAN	**0,85**<br>0,96	**0,86**<br>**0,88**	**0,82**						
Long-chain fatty acid transport protein 1	Q6PCB7|S27A1_HUMAN	0,85<br>0,85	0,73<br>1,07	0,77						
Long-chain fatty acid transport protein 3	Q5K4L6|S27A3_HUMAN	1,05<br>**0,90**	**1,20**<br>**1,16**	**1,25**	1,44	0,81	0,81	1,47	0,71	0,38
Long-chain fatty acid transport protein 4	Q6P1M0|S27A4_HUMAN	0,86	0,96							
Long-chain-fatty-acid--CoA ligase 1	P33121|ACSL1_HUMAN	1,05<br>1,09	0,99<br>1,07	1,08						
Long-chain-fatty-acid--CoA ligase 3	O95573|ACSL3_HUMAN	1,12<br>0,93	1,37<br>**1,18**	**1,40**						
Long-chain-fatty-acid--CoA ligase 4	O60488|ACSL4_HUMAN	0,90	0,92	1,12						
Long-chain-fatty-acid--CoA ligase 5	Q9ULC5|ACSL5_HUMAN	0,94	0,99							
Low affinity immunoglobulin gamma Fc region receptor II-c precursor	P31995|FCG2C_HUMAN	1,25	0,95	1,18						
LRRFIP1 protein (Fragment)	Q05D04|Q05D04_HUMAN				0,83	1,15	1,00			
LSM3 protein	Q6IAH0|Q6IAH0_HUMAN				0,60	1,03	0,64	0,00<br>1,01	0,00<br>0,66	0,00<br>0,35
LSP1 protein	Q6FHM3|Q6FHM3_HUMAN				**2,24**<br>1,56	**2,52**<br>**1,73**	**3,13**<br>1,99	1,01	**0,30**	**0,18**
LTA4H protein	Q6IAT6|Q6IAT6_HUMAN	0,00<br>**2,79**	0,00<br>**2,97**	0,00	0,97<br>1,00	0,96<br>1,00	**0,74**<br>0,86			
Lung cancer oncogene 7	Q5VLR4|Q5VLR4_HUMAN				**1,38**	1,11	**1,72**			
L-xylulose reductase	Q7Z4W1|DCXR_HUMAN	1,08<br>1,00	1,13<br>0,94	1,08						
Lymphocyte antigen 75 precursor	O60449|LY75_HUMAN	**1,23**	1,08							
Lymphocyte cytosolic protein 1	Q5TBN4|Q5TBN4_HUMAN	**1,62**<br>**1,58**	**1,86**<br>**1,63**	**3,33**	**0,75**<br>0,68	**1,04**<br>**0,94**	1,02<br>0,81	1,29	**1,71**	**2,97**
Lymphocyte-specific protein 1 variant (Fragment)	Q53H48|Q53H48_HUMAN	**1,47**<br>**1,85**	1,08<br>**1,29**	**1,62**						
LYN protein (Fragment)	Q6NUK7|Q6NUK7_HUMAN	1,09<br>1,09	1,09<br>1,06	1,40	2,10	1,46	1,52	**0,46**	0,44	0,42
LYPLA1 protein	Q6IAQ1|Q6IAQ1_HUMAN	1,09<br>1,05	1,14<br>1,10	0,73	0,97<br>0,87	0,89<br>0,98	1,12<br>**0,79**			
LYR motif-containing protein 2	Q9NU23|LYRM2_HUMAN	1,41<br>0,96	1,16<br>0,97	0,52						
LYR motif-containing protein 5	Q6IPR1|LYRM5_HUMAN							0,00<br>7,33	0,00<br>2,46	0,00<br>2,16
LYR motif-containing protein 7	Q5U5X0|LYRM7_HUMAN	1,28	0,84	0,85						
Lysophosphatidylcholine acyltransferase	Q6P1A2|MBOA5_HUMAN	0,73<br>0,83	0,93<br>1,17	0,77						
Lysophosphatidylcholine acyltransferase 2	Q7L5N7|PCAT2_HUMAN	1,05<br>0,97	**1,29**<br>1,11	**1,37**	0,00	0,00	0,00		13,29	26,56
Lysophospholipase-like protein 1	Q5VWZ2|LYPL1_HUMAN	0,85	0,79							
Lysosomal acid phosphatase precursor	P11117|PPAL_HUMAN	1,11<br>**0,90**	1,17<br>0,97	0,94						
Lysosomal alpha-glucosidase precursor	P10253|LYAG_HUMAN	**1,22**<br>0,93	**1,73**<br>0,98	**1,39**	**1,77**<br>**1,99**	**1,29**<br>1,13	**2,00**<br>**1,79**			
Lysosomal alpha-mannosidase precursor	O00754|MA2B1_HUMAN	0,95	**0,94**		1,61	1,03	**1,53**			
Lysosomal membrane glycoprotein LAMP-2 homolog	Q9UD93|Q9UD93_HUMAN	0,92<br>**0,68**	1,28<br>**0,80**	1,62						
Lysosomal Pro-X carboxypeptidase precursor	P42785|PCP_HUMAN	1,14<br>**0,91**	1,55<br>0,87	**1,53**						

Lysosome-associated membrane glycoprotein 1 precursor	P11279|LAMP1_HUMAN	**1,15**<br>**0,82**	1,12<br>**0,95**	**0,88**	**1,91**<br>1,90	0,86<br>0,88	1,03<br>1,19			
Lysosome-associated membrane protein-3 variant	Q8N6Z9|Q8N6Z9_HUMAN	0,94<br>0,99	**0,78**<br>0,94	**0,69**	**2,14**<br>2,18	**0,79**<br>**0,74**	1,02<br>0,87	1,50	**2,44**	**4,21**
Lysozyme C precursor	P61626|LYSC_HUMAN	**0,80**<br>**0,88**	**0,57**<br>**0,56**	**0,43**	**1,66**<br>1,37	0,89<br>**0,71**	**1,26**<br>0,88	**0,73**<br>0,98	**0,58**<br>**0,82**	**0,57**<br>**0,86**
Lysyl-tRNA synthetase	Q9HB23|Q9HB23_HUMAN				**0,69**	1,01	0,83			
M6PR protein	Q6I9U3|Q6I9U3_HUMAN	1,00<br>0,90	0,92<br>0,92	1,28	**1,81**<br>1,86	**1,59**<br>**1,35**	**1,81**<br>**1,40**	1,50<br>1,86	3,15<br>1,64	6,60<br>3,11
MACRO domain-containing protein 1	Q9BQ69|MACD1_HUMAN	1,01	1,07							
Macrophage mannose receptor 2 precursor	Q9UBG0|MRC2_HUMAN	1,10	0,90							
Macrophage scavenger receptor types I and II	P21757|MSRE_HUMAN	0,88<br>1,00	**0,62**<br>**0,73**	**0,57**				1,06<br>**1,26**	**1,35**<br>0,98	**1,37**<br>1,25
Macrophage-capping protein	P40121|CAPG_HUMAN	1,32<br>1,16	2,19<br>1,28	**3,58**	**0,82**<br>0,74	**0,83**<br>**0,85**	**0,73**<br>0,66	1,03<br>1,13	1,05<br>0,66	0,95<br>**0,58**
Magnesium transporter MRS2 homolog, mitochondrial precursor	Q9HD23|MRS2_HUMAN	0,74<br>0,95	**0,76**<br>1,19	0,66						
Magnesium transporter protein 1 precursor	Q9H0U3|MAGT1_HUMAN							0,74	**2,22**	**2,86**
MAGUK p55 subfamily member 4	Q96JB8|MPP4_HUMAN	1,10	1,23							
Major facilitator superfamily domain-containing protein 10	Q14728|MFS10_HUMAN	0,97	1,23	0,96						
Major histocompatibility complex, class I, A	Q5SUL4|Q5SUL4_HUMAN	**1,38**	1,00	1,17						
Major histocompatibility complex, class I, C	B0S7Z6|B0S7Z6_HUMAN							1,40	**4,19**	**5,03**
Major histocompatibility complex, class II, DP beta 1	Q95HC1|Q95HC1_HUMAN	1,18<br>0,93	1,05<br>0,92	0,98	**1,42**<br>1,71	0,98<br>1,14	**1,50**<br>**1,36**	1,03	**2,29**	**2,32**
Major histocompatibility complex, class II, DP beta 1	Q5SQ72|Q5SQ72_HUMAN							1,11	**1,37**	**1,83**
Major histocompatibility complex, class II, DR alpha	Q5SP06|Q5SP06_HUMAN	0,96	**0,73**	**0,83**	**1,95**	1,10	**1,42**	1,31	1,68	**2,40**
Major histocompatibility complex, class II, DR beta 3	Q5STE0|Q5STE0_HUMAN	0,87	0,69							
Major prion protein (Fragment)	Q86XR1|Q86XR1_HUMAN	0,00<br>0,00	0,00<br>0,00	0,00				1,31	**0,46**	0,60
Major vault protein	Q14764|MVP_HUMAN	1,71	1,75	**5,79**	0,83<br>1,00	1,08<br>**1,26**	**1,37**<br>1,82	1,48	1,40	3,45
Malate dehydrogenase	A8K414|A8K414_HUMAN	**0,84**<br>**0,89**	**0,87**<br>**0,93**	**0,57**	1,84	1,43	2,04	**0,75**	1,19	**1,94**
Malate dehydrogenase (Fragment)	Q0QF37|Q0QF37_HUMAN				**2,19**	**1,81**	**2,97**			
Malate dehydrogenase, cytoplasmic	P40925|MDHC_HUMAN	1,05	1,14	0,91	**0,86**<br>**0,87**	0,98<br>1,02	0,95<br>1,02			
Malate dehydrogenase, mitochondrial precursor	P40926|MDHM_HUMAN							**1,16**	**1,79**	**3,59**
Malonyl CoA-acyl carrier protein transacylase, mitochondrial precursor	Q8IVS2|FABD_HUMAN	0,92	0,93							
Malonyl-CoA decarboxylase, mitochondrial precursor	O95822|DCMC_HUMAN	1,09<br>1,00	1,08<br>0,89	1,10						
Mannose receptor	Q0Z8D6|Q0Z8D6_HUMAN				**2,18**	1,34	1,42	1,07	1,14	1,73
Mannose receptor C type 1-like protein 1	Q5VSK2|Q5VSK2_HUMAN	**1,08**<br>**1,09**	**0,71**<br>**0,76**	**0,53**						
Mannose-1-phosphate guanyltransferase alpha	Q96IJ6|GMPPA_HUMAN				0,00	0,00	0,00			
Mannose-6-phosphate receptor-binding protein 1	O60664|M6PBP_HUMAN	**0,87**	1,14	1,37	**0,82**<br>0,67	**0,87**<br>**0,72**	**0,84**<br>**0,70**			
Mannose-P-dolichol utilization defect 1	Q9BUU8|Q9BUU8_HUMAN	0,83	0,95	0,98						
Mannosidase, alpha, class 2B, member 1 variant (Fragment)	Q59E90|Q59E90_HUMAN	**1,16**	**1,61**	**1,40**						
Mannosyl (Alpha-1,3-)-glycoprotein beta-1,2-N-acetylglucosaminyltransferase variant (Fragment)	Q59G70|Q59G70_HUMAN	1,09	0,97							
Mannosyl-oligosaccharide 1,2-alpha-mannosidase IA	P33908|MA1A1_HUMAN	1,01<br>0,95	**0,41**<br>**0,74**	**0,48**						
Mannosyl-oligosaccharide glucosidase	Q13724|GCS1_HUMAN	0,97	1,09							
MAP kinase-activated protein kinase 3	Q16644|MAPK3_HUMAN				1,00<br>1,03	0,90<br>1,00	0,73<br>0,93			
Maspardin	Q9NZD8|SPG21_HUMAN	1,16	1,20							
Mast cell-expressed membrane protein 1	P24534|EF1B_HUMAN	1,28	0,98	**1,30**				1,19	**2,03**	**1,93**
Matrin-3	P43243|MATR3_HUMAN							**0,65**	**0,27**	**0,25**
Matrix metalloproteinase-14 precursor	P50281|MMP14_HUMAN	0,92<br>**1,23**	0,86<br>1,20	1,03						
Matrix metalloproteinase-21 precursor	Q8N119|MMP21_HUMAN									
Matrix metalloproteinase-9 precursor	P14780|MMP9_HUMAN	**0,73**<br>**0,87**	**0,30**<br>**0,75**	**0,28**						
Matrix protein - Infectious hematopoietic necrosis virus (strain WRAC)								0,00	0,00	0,00
Matrix protein 1	P63233|M1_I72A2	**2,42**<br>1,25	**8,17**<br>**5,19**	**16,62**	**2,73**<br>**2,26**	**10,84**<br>8,28	**7,38**<br>6,04	**10,96**<br>**6,64**	**12,29**<br>**8,33**	**5,53**<br>**5,05**
Mature T-cell proliferation 1	Q5HYP9|Q5HYP9_HUMAN	0,64	0,79							
Megakaryoblastic leukemia (Translocation) 1	B0QY84|B0QY84_HUMAN	0,00	0,00							
Megakaryocyte-associated tyrosine-protein kinase	P42679|MATK_HUMAN	0,00	0,00	0,00						
Melanoma inhibitory activity protein 3 precursor	Q5JRA6|MIA3_HUMAN	0,88<br>1,05	0,89<br>**1,21**	0,95						
Membrane alanine aminopeptidase variant (Fragment)	Q59E93|Q59E93_HUMAN	**1,40**<br>**1,07**	**1,28**<br>1,02	**1,26**	**1,71**<br>**1,63**	1,13<br>1,13	**1,58**<br>**1,47**	**1,15**<br>0,95	**0,50**<br>**0,56**	**0,65**<br>**0,56**
Membrane-associated progesterone receptor component 2	O15173|PGRC2_HUMAN	1,12<br>1,00	**1,31**<br>1,06	**1,31**	0,00	0,00	0,00	1,35	2,18	4,47
Membrane-bound O-acyltransferase domain-containing protein 1	Q6ZNC8|MBOA1_HUMAN	0,99	0,91							
Membrane-bound O-acyltransferase domain-containing protein 7	Q96N66|MBOA7_HUMAN	0,91<br>0,83	1,11<br>0,90	1,01						
Mesoderm development candidate 2	Q14696|MESD2_HUMAN	0,95<br>0,97	1,00<br>1,05	0,89	**2,17**<br>**1,57**	1,19<br>1,06	2,63<br>**1,52**	0,97<br>1,04	**2,99**<br>**2,31**	**3,40**<br>3,67
Metalloreductase STEAP3	Q658P3|STEA3_HUMAN	1,30	0,84	0,92						
Metastasis-associated protein MTA2	O94776|MTA2_HUMAN							1,31<br>**0,31**	0,82<br>**0,30**	0,89<br>**0,29**
Metaxin 1	Q9BUU3|Q9BUU3_HUMAN	0,98<br>0,94	0,95<br>0,89	**0,82**				0,84	**1,69**	**2,24**
Metaxin 2	Q8IZ68|Q8IZ68_HUMAN	0,88<br>1,09	0,80<br>0,93	0,80				0,00	0,00	0,00
Methionine aminopeptidase 1	P53582|AMPM1_HUMAN							0,00	0,00	0,00
	Q9Y3D2|MSRB2_HUMAN									

Methionine-R-sulfoxide reductase B2, mitochondrial precursor		0,87	0,75							
Methionyl-tRNA formyltransferase, mitochondrial precursor	Q96DP5|FMT_HUMAN	0,85	1,23							
Methionyl-tRNA synthetase, cytoplasmic	P56192|SYMC_HUMAN				0,89	0,96	**0,71**			
Methionyl-tRNA synthetase, mitochondrial precursor	Q96GW9|SYMM_HUMAN	0,92	0,85							
Methyl-CpG-binding domain protein 1	Q9UIS9|MBD1_HUMAN							0,00	0,00	0,00
Methyl-CpG-binding domain protein 2	Q9UBB5|MBD2_HUMAN							0,85<br>**0,76**	**0,52**<br>0,80	0,52<br>0,82
Methyl-CpG-binding domain protein 4	O95243|MBD4_HUMAN							0,00	0,00	0,00
Methyl-CpG-binding protein 2	P51608|MECP2_HUMAN							1,11<br>1,80	0,56<br>1,41	0,59<br>1,86
Methylcrotonoyl-CoA carboxylase beta chain, mitochondrial precursor	Q9HCC0|MCCB_HUMAN	0,96<br>0,95	0,89<br>0,95	0,78						
Methylcrotonoyl-CoA carboxylase subunit alpha, mitochondrial precursor	Q96RQ3|MCCA_HUMAN	0,92<br>1,07	0,90<br>1,02	0,92						
Methylmalonic aciduria type A protein, mitochondrial precursor	Q8IVH4|MMAA_HUMAN	**0,40**<br>1,12	**0,50**<br>0,94	**0,31**						
Methylmalonyl-CoA epimerase, mitochondrial precursor	Q96PE7|MCEE_HUMAN	0,00<br>1,05	0,00<br>1,04	0,00						
Methylmalonyl-CoA mutase, mitochondrial precursor	P22033|MUTA_HUMAN	**0,85**<br>1,05	**0,84**<br>0,99	**0,64**						
Methyltransferase-like protein 7A precursor	Q9H8H3|MET7A_HUMAN	1,14	**1,12**							
Methyltransferase-like protein 7B precursor	Q6UX53|MET7B_HUMAN	1,15	1,18							
MGAT1 protein	Q6IBE3|Q6IBE3_HUMAN	1,08	**0,74**	**0,73**						
MGC70857 protein	Q6GMR2|Q6GMR2_HUMAN	0,84	0,63	0,64						
MGST1 protein (Fragment)	Q6LET6|Q6LET6_HUMAN	0,00<br>0,94	0,00<br>0,80	0,00						
MHC cell surface glycoprotein precursor	Q30118|Q30118_HUMAN	1,05	0,95		**1,77**	1,09	1,13			
MHC class I antigen	Q546L8|Q546L8_HUMAN	0,93	0,82							
MHC class I antigen	A4H284|A4H284_HUMAN	0,98	1,14							
MHC class I antigen (Fragment)	Q6F3I9|Q6F3I9_HUMAN	1,72	1,61	1,55	**1,49**	0,86	1,07			
MHC class I antigen (Fragment)	Q860B1|Q860B1_HUMAN	0,90	0,78							
MHC class I antigen (Fragment)	Q6F3H8|Q6F3H8_HUMAN	1,09	0,85	0,98						
MHC class I antigen (Fragment)	A3F720|A3F720_HUMAN				1,71	0,94	**0,72**			
MHC class I antigen (Fragment)	Q0VJ91|Q0VJ91_HUMAN				1,44	0,97	1,00			
MHC class I antigen (Fragment)	Q5JZI4|Q5JZI4_HUMAN							1,75	2,89	3,16
MHC class I antigen precursor (Fragment)	Q6R741|Q6R741_HUMAN	0,92<br>0,95	0,78<br>0,90	0,84						
MHC class II antigen	Q6YJU6|Q6YJU6_HUMAN	0,00	0,00	0,00	1,63	1,37	1,38			
MHC class II antigen	Q58ZE2|Q58ZE2_HUMAN	**0,78**	**0,90**							
MHC class II antigen	Q5Y7D6|Q5Y7D6_HUMAN	1,06	**0,86**							
MHC class II antigen (Fragment)	Q8N5A0|Q8N5A0_HUMAN	0,95	**0,76**	0,88				1,21	1,62	2,14
MHC class II antigen (Fragment)	Q5DUF5|Q5DUF5_HUMAN	0,62	0,80							
MHC class II antigen (Fragment)	Q3LA86|Q3LA86_HUMAN	1,01	0,98							
MHC class II antigen (Fragment)	Q6S5I0|Q6S5I0_HUMAN				**1,99**	0,92	1,09			
Microfibrillar-associated protein 1	Q86TG6|Q86TG6_HUMAN							**0,82**<br>1,04	**0,28**<br>0,64	**0,15**<br>0,53
Microphthalmia-associated transcription factor	O75030|MITF_HUMAN							1,01	0,28	**0,20**
Microsomal glutathione S-transferase 2	Q99735|MGST2_HUMAN	0,00<br>0,79	0,00<br>0,70	0,00				0,00	0,00	0,00
Microsomal glutathione S-transferase 3	Q13011|ECH1_HUMAN	0,92	0,99	0,93				1,89	1,53	1,95
Microsomal glutathione S-transferase 3	Q5VV89|Q5VV89_HUMAN	0,98	1,02					**0,38**	1,01	1,57
Microsomal glutathione S-transferase 3 variant (Fragment)	Q53GB9|Q53GB9_HUMAN				**2,89**	1,99	1,52			
Microtubule-associated protein 1S	Q66K74|MAP1S_HUMAN				0,55	0,98	0,71	0,69	**0,74**	**0,51**
Microtubule-associated protein 4	P27816|MAP4_HUMAN				**0,72**<br>0,56	**0,76**<br>0,69	**0,64**<br>0,57	0,94<br>**1,32**	1,04<br>**1,88**	**0,50**<br>1,40
Microtubule-associated protein RP/EB family member 1	Q15691|MARE1_HUMAN				0,88<br>**0,70**	1,01<br>**0,85**	**0,87**<br>**0,57**	1,10	**1,68**	**1,29**
Midasin	Q9NU22|MDN1_HUMAN	0,95	0,87					0,00	0,00	0,00
Migration-inducing gene 14	Q5J8M4|Q5J8M4_HUMAN							0,76	1,00	0,81
Migration-inducing protein 12	Q596K9|Q596K9_HUMAN	0,00	0,00	0,00	**0,53**<br>0,46	**0,71**<br>**0,59**	**0,59**<br>**0,43**			
Mimitin, mitochondrial precursor	Q8N183|MIMIT_HUMAN	0,94<br>**1,24**	1,13<br>1,10	**0,74**				1,19<br>0,00	4,26<br>0,00	**4,48**<br>0,00
Minichromosome maintenance protein 3 variant (Fragment)	Q53HJ4|Q53HJ4_HUMAN				0,85	0,89	0,72			
Minor histocompatibility antigen H13	Q8TCT9|HM13_HUMAN	**0,88**<br>**0,89**	1,04<br>1,05	1,06				0,84	0,97	2,61
Mitochondrial 18 kDa protein	Q9UDX5|MTP18_HUMAN	1,24<br>1,07	0,94<br>1,03	**0,69**						
Mitochondrial 28S ribosomal protein S10	P82664|RT10_HUMAN	0,89	0,89							
Mitochondrial 28S ribosomal protein S14	O60783|RT14_HUMAN	1,11	1,22							
Mitochondrial 28S ribosomal protein S2	Q9Y399|RT02_HUMAN	**1,27**<br>0,98	1,11<br>1,01	1,06						
Mitochondrial 28S ribosomal protein S22	P82650|RT22_HUMAN	**0,80**<br>**0,90**	0,75<br>**0,90**	**0,62**						
Mitochondrial 28S ribosomal protein S25	P82663|RT25_HUMAN	0,00<br>0,88	0,00<br>0,99	0,00						
Mitochondrial 28S ribosomal protein S27	Q92552|RT27_HUMAN	0,00	0,00							
Mitochondrial 28S ribosomal protein S28	Q9Y2Q9|RT28_HUMAN	1,13<br>1,07	1,62<br>0,90	1,98						
Mitochondrial 28S ribosomal protein S30	Q9NP92|RT30_HUMAN	1,01<br>1,01	0,93<br>1,00	**0,75**						
Mitochondrial 28S ribosomal protein S33	Q9Y291|RT33_HUMAN	0,00<br>**0,72**	0,00<br>0,83	0,00						

Mitochondrial 28S ribosomal protein S34	P82930|RT34_HUMAN	0,94<br>0,99	0,88<br>0,92	0,68						
Mitochondrial 28S ribosomal protein S36	P82909|RT36_HUMAN	0,83<br>1,03	1,00<br>**0,87**	0,90				2,15<br>1,65	4,39<br>2,46	5,94<br>4,51
Mitochondrial 28S ribosomal protein S6	P82932|RT06_HUMAN	0,99<br>1,02	0,82<br>0,90	**0,60**						
Mitochondrial 39S ribosomal protein L23	Q16540|RM23_HUMAN	0,80	**0,85**							
Mitochondrial 39S ribosomal protein L27	Q9P0M9|RM27_HUMAN	1,01<br>0,92	1,06<br>**1,11**	1,14						
Mitochondrial 39S ribosomal protein L39	Q9NYK5|RM39_HUMAN	1,00<br>0,91	**0,83**<br>0,96	**0,69**						
Mitochondrial 39S ribosomal protein L4	Q9BYD3|RM04_HUMAN	0,94<br>0,93	0,93<br>**0,83**	0,79						
Mitochondrial 39S ribosomal protein L49	Q13405|RM49_HUMAN	1,20<br>0,97	0,94<br>0,98	1,13						
Mitochondrial 39S ribosomal protein L50	Q8N5N7|RM50_HUMAN	0,62<br>0,80	0,78<br>0,88	0,39						
Mitochondrial aldehyde dehydrogenase 2 variant (Fragment)	Q53FB6|Q53FB6_HUMAN	0,98<br>0,96	0,94<br>**0,91**	**0,70**	2,01<br>**2,29**	2,05<br>1,44	**3,57**<br>**2,17**			
Mitochondrial antiviral-signaling protein	Q7Z434|MAVS_HUMAN	0,00	0,00		0,90	0,70	0,91			
Mitochondrial carnitine/acylcarnitine carrier protein	O43772|MCAT_HUMAN	1,10<br>0,94	0,99<br>**0,79**	0,84						
Mitochondrial carnitine/acylcarnitine carrier protein CACL	Q8N8R3|MCATL_HUMAN	0,98<br>0,94	0,89<br>**0,76**	0,84						
Mitochondrial carrier homolog 2	Q9Y6C9|MTCH2_HUMAN	**1,17**<br>**1,14**	0,91<br>**0,91**	0,90				1,36	1,09	**2,85**
Mitochondrial carrier homolog 2 variant (Fragment)	Q53G34|Q53G34_HUMAN							**0,49**	0,93	**1,26**
Mitochondrial chaperone BCS1	Q9Y276|BCS1_HUMAN	1,03	1,16							
Mitochondrial citrate transport protein (Fragment)	Q6LAP8|Q6LAP8_HUMAN	0,97	**0,89**					1,23<br>1,13	0,90<br>**1,43**	**2,35**<br>**2,33**
Mitochondrial deoxynucleotide carrier	Q9HC21|DNC_HUMAN	1,17<br>**1,07**	1,04<br>1,02	0,80						
Mitochondrial dicarboxylate carrier	Q9UBX3|DIC_HUMAN	1,16<br>1,01	1,24<br>0,93	1,25						
Mitochondrial dimethyladenosine transferase 1, mitochondrial precursor	Q8WVM0|TFB1M_HUMAN	**0,86**	0,99							
Mitochondrial dimethyladenosine transferase 2, mitochondrial precursor	Q9H5Q4|TFB2M_HUMAN	0,96	0,89							
Mitochondrial fission 1 protein	Q9Y3D6|FIS1_HUMAN	1,00	0,86							
Mitochondrial folate transporter/carrier	Q9H2D1|MFTC_HUMAN	0,95	0,88							
Mitochondrial glutamate carrier 1	Q9H936|GHC1_HUMAN	**1,40**<br>0,94	1,20<br>0,99	1,16						
Mitochondrial import inner membrane translocase subunit Tim10	P62072|TIM10_HUMAN	0,95<br>1,18	0,82<br>0,85	**0,53**				0,79	1,93	**2,67**
Mitochondrial import inner membrane translocase subunit Tim13	Q9Y5L4|TIM13_HUMAN	0,00<br>0,81	0,00<br>0,95	0,00				0,00	0,00	0,00
Mitochondrial import inner membrane translocase subunit TIM14	Q96DA6|TIM14_HUMAN	1,02<br>1,01	0,91<br>0,98	**0,76**						
Mitochondrial import inner membrane translocase subunit Tim16	Q9Y3D7|TIM16_HUMAN							0,85	1,93	1,72
Mitochondrial import inner membrane translocase subunit Tim22	Q9Y584|TIM22_HUMAN	0,97	0,84							
Mitochondrial import inner membrane translocase subunit Tim23	O14925|TIM23_HUMAN	0,00<br>0,66	0,00<br>0,78	0,00						
Mitochondrial import inner membrane translocase subunit Tim8 B	Q9Y5J9|TIM8B_HUMAN	0,79<br>0,92	0,45<br>0,92	0,30						
Mitochondrial import inner membrane translocase subunit Tim9	Q9Y5J7|TIM9_HUMAN	1,00<br>**1,22**	0,94<br>1,12	**0,59**				0,70	1,69	4,18
Mitochondrial import receptor subunit TOM20 homolog	Q15388|TOM20_HUMAN	0,84<br>**1,27**	0,91<br>1,27	0,85						
Mitochondrial import receptor subunit TOM22 homolog	Q9NS69|TOM22_HUMAN	0,00<br>**0,74**	0,00<br>1,00	0,00				0,00	0,00	0,00
Mitochondrial import receptor subunit TOM40 homolog	O96008|TOM40_HUMAN	1,22<br>1,08	0,92<br>**1,15**	0,85				1,10<br>**0,31**	1,23<br>0,72	3,33<br>1,01
Mitochondrial import receptor subunit TOM7 homolog	Q9P0U1|TOM7_HUMAN	1,00	**0,69**					0,85<br>**1,36**	1,25<br>0,95	1,07<br>1,21
Mitochondrial inner membrane protein	Q16891|IMMT_HUMAN	0,95<br>1,03	0,95<br>**1,06**	**0,88**	1,38<br>**1,66**	0,93<br>0,99	1,08<br>0,87	**0,85**<br>**0,50**	**1,26**<br>0,89	**1,75**<br>**1,25**
Mitochondrial intermediate peptidase, mitochondrial precursor	Q99797|MIPEP_HUMAN	0,88<br>1,00	0,83<br>0,97	0,64						
Mitochondrial intermembrane space import and assembly protein 40	Q8N4Q1|MIA40_HUMAN	0,97<br>1,05	0,87<br>0,87	**0,57**						
Mitochondrial PDHA1	A5YVE9|A5YVE9_HUMAN	0,96	0,97							
Mitochondrial precursor proteins import receptor	O94826|TOM70_HUMAN	0,99<br>**1,21**	0,93<br>**1,21**	0,84						
Mitochondrial Rho GTPase 1	Q8IXI2|MIRO1_HUMAN	1,43<br>1,00	0,97<br>1,07	1,28						
Mitochondrial Rho GTPase 2	Q8IXI1|MIRO2_HUMAN	1,02<br>0,92	**0,77**<br>0,93	0,88						
Mitochondrial ribosomal protein 63	Q9BQC6|RT63_HUMAN	0,81	**0,68**	**0,63**						
Mitochondrial ribosomal protein L28	Q4TT38|Q4TT38_HUMAN	0,99	1,00							
Mitochondrial ribosomal protein L43	B1AL10|B1AL10_HUMAN	0,94	0,95							
Mitochondrial ribosomal protein S11 isoform a variant (Fragment)	Q53FX9|Q53FX9_HUMAN	0,89	0,94							
Mitochondrial ribosomal protein S18A (Fragment)	Q5QPA5|Q5QPA5_HUMAN	0,87	0,82	**0,71**						
Mitochondrial ribosomal protein S21	Q5TB11|Q5TB11_HUMAN	0,00<br>0,98	0,00<br>0,83	0,00						
Mitochondrial ribosomal protein S23	Q9Y3D9|RT23_HUMAN	1,09<br>0,95	1,13<br>0,88	0,87						
Mitochondrial ribosomal protein S27	Q6P1S1|Q6P1S1_HUMAN	0,82<br>0,00	0,79<br>0,00	**0,64**						
Mitochondrial ribosomal protein S9	Q6PG40|Q6PG40_HUMAN	1,35<br>1,21	1,89<br>**2,25**	1,75						
Mitochondrial-processing peptidase subunit beta, mitochondrial precursor	O75439|MPPB_HUMAN	0,93<br>**0,89**	**0,81**<br>**0,86**	**0,54**						
Mitofusin-2	O95140|MFN2_HUMAN	1,00<br>**1,22**	0,81<br>1,12	**0,75**						
Mitogen-activated protein kinase 1	Q499G7|Q499G7_HUMAN				0,92<br>**0,87**	1,03<br>0,92	0,92<br>0,82			
Mitogen-activated protein kinase kinase 1-interacting protein 1	Q9UHA4|MK1I1_HUMAN	1,52	1,44							
Mitogen-activated protein-binding protein-interacting protein	A8K534|A8K534_HUMAN							1,62	1,92	1,87
Mitotic checkpoint protein BUB3	O43684|BUB3_HUMAN							1,79<br>0,33	0,56<br>**0,46**	0,64<br>0,37
MLL/SEPTIN6 fusion protein (Fragment)	Q8NFH9|Q8NFH9_HUMAN							1,22	**0,68**	**0,42**
Moesin	P26038|MOES_HUMAN	**1,79**	**1,57**	**1,62**	0,99	**1,13**	**1,14**	**1,63**	1,16	**1,45**

		**2,00**	**1,41**		0,97	1,24	**1,25**	1,05	1,01	1,14
Monocarboxylate transporter 4	O15427|MOT4_HUMAN	**1,41**<br>1,16	0,92<br>0,94	1,00						
MOSC domain-containing protein 1, mitochondrial precursor	Q5VT66|MOSC1_HUMAN	0,98	1,04							
Motile sperm domain-containing protein 2	Q8NHP6|MSPD2_HUMAN	0,95<br>0,95	1,02<br>1,06	1,03						
M-phase phosphoprotein 6	Q99547|MPH6_HUMAN							1,01<br>1,17	0,27<br>0,50	0,08<br>**0,39**
Mps one binder kinase activator-like 1B	Q9H8S9|MOL1B_HUMAN				0,00	0,00	0,00			
Mps one binder kinase activator-like 2A	Q96BX8|MOL2A_HUMAN				0,84	0,63	0,74			
MPST protein (Fragment)	Q6FHN9|Q6FHN9_HUMAN	0,96<br>1,07	1,00<br>0,90	1,09	0,00	0,00	0,00			
MRNA encoding beta-tubulin, (Fragment)	Q6LC01|Q6LC01_HUMAN	1,10	1,71					0,89	**2,95**	**3,13**
MRPL3 protein	Q6IBT2|Q6IBT2_HUMAN	1,17<br>0,95	1,20<br>0,96	1,29						
MRPL47 protein	Q8N5D1|Q8N5D1_HUMAN	1,02	1,08							
MTCH1 protein (Fragment)	A4FVA6|A4FVA6_HUMAN	1,22	1,20	1,38						
MTCH1 protein (Fragment)	Q8IW90|Q8IW90_HUMAN	0,90	**0,88**					0,00	0,00	0,00
Multidrug resistance-associated protein 1	P33527|MRP1_HUMAN	1,03	0,96							
Multifunctional protein ADE2	P22234|PUR6_HUMAN				**0,73**	**0,83**	**0,58**			
Multisynthetase complex auxiliary component p38	Q13155|MCA2_HUMAN				**0,83**<br>0,79	0,79<br>0,97	0,82<br>0,75			
Multisynthetase complex auxiliary component p43	Q12904|MCA1_HUMAN				1,09<br>0,65	0,82<br>1,03	0,61<br>0,61			
Muscleblind-like protein 1	Q9NR56|MBNL1_HUMAN				**1,96**<br>1,16	**1,48**<br>0,73	**2,08**<br>**1,59**	**1,25**	**1,33**	**1,20**
Myb-binding protein 1A	Q9BQG0|MBB1A_HUMAN							0,00<br>**0,20**	0,00<br>**0,40**	0,00<br>0,36
Myc box-dependent-interacting protein 1	O00499|BIN1_HUMAN				0,83<br>0,82	0,64<br>1,03	0,94<br>1,59			
Myeloid cell nuclear differentiation antigen	Q5VUU6|Q5VUU6_HUMAN	**1,27**<br>0,99	**1,68**<br>1,05	**2,31**	**1,17**<br>1,26	1,11<br>1,57	0,90<br>1,11	**1,11**<br>**0,81**	**0,57**<br>**0,76**	**0,45**<br>**0,66**
Myeloid cell surface CD33 precursor	P20138|CD33_HUMAN	1,42	1,01							
Myeloid-associated differentiation marker	Q96S97|MYADM_HUMAN	1,17<br>1,09	0,77<br>1,14	0,86						
Myeloperoxidase precursor	P05164|PERM_HUMAN	1,05<br>**0,90**	**1,55**<br>0,94	**1,17**						
MYO1C variant protein (Fragment)	Q4LE56|Q4LE56_HUMAN	1,39<br>**1,14**	1,33<br>1,05	1,60						
MYO1G variant protein	B0I1T2|B0I1T2_HUMAN	1,20<br>1,11	0,89<br>0,88	0,97						
MYO9B variant protein	B0I1T6|B0I1T6_HUMAN				0,68	0,55	0,17			
Myoferlin	Q9NZM1|MYOF_HUMAN	**1,13**<br>**0,95**	**0,95**<br>0,98	1,01	9999,00<br>2,44	1,89	9999,00<br>2,54	1,40<br>**0,55**	**4,04**<br>**3,62**	**11,25**<br>**5,08**
Myosin IE	Q4KMR3|Q4KMR3_HUMAN				0,73	0,70	0,60			
Myosin regulatory light chain	Q53X45|Q53X45_HUMAN	1,09<br>1,09	1,07<br>**1,55**	**1,91**	0,93	**0,85**	**1,16**	**3,10**<br>**1,78**	**5,41**<br>**7,05**	**4,65**<br>**6,56**
Myosin regulatory light chain MRCL3 variant (Fragment)	Q53HL1|Q53HL1_HUMAN				**0,78**	**0,72**	**0,72**			
Myosin, light chain 6, alkali, smooth muscle and non-muscle	Q561V9|Q561V9_HUMAN	1,02<br>**1,34**	**1,19**<br>**1,47**	**2,06**	**0,86**<br>0,66	**0,88**<br>**0,80**	1,07<br>**0,80**	**1,84**<br>**1,33**	**2,64**<br>**2,95**	**2,64**<br>**3,06**
Myosin-Id	O94832|MYO1D_HUMAN	1,07	0,99							
Myosin-If	O00160|MYO1F_HUMAN	**1,39**	**1,69**	**2,72**	**1,52**	**1,27**	1,27			
Myosin-IXb	Q13459|MYO9B_HUMAN				1,49	0,90	1,49			
Myosin-Va	Q9Y4I1|MYO5A_HUMAN	0,00<br>1,01	0,00<br>0,79	0,00						
Myotrophin	P58546|MPTN_HUMAN				1,06<br>0,68	1,10<br>**0,78**	1,07<br>0,74			
Myristoylated alanine-rich C-kinase substrate	P29966|MARCS_HUMAN	1,19	1,28		1,21<br>1,04	1,18<br>1,11	1,35<br>1,20			
N(4)-(beta-N-acetylglucosaminyl)-L-asparaginase precursor	P20933|ASPG_HUMAN	0,97	0,92							
Na+/K+ transporting ATPase beta 3 polypeptide (Fragment)	Q58I18|Q58I18_HUMAN				0,83	0,71	0,84			
N-acylethanolamine-hydrolyzing acid amidase precursor	Q02083|ASAHL_HUMAN	1,01	0,93							
N-acetylgalactosamine-6-sulfatase precursor	P34059|GALNS_HUMAN	1,09<br>1,04	1,12<br>0,91	1,05						
N-acetylgalactosaminyltransferase 7	Q86SF2|GALT7_HUMAN	1,12<br>1,01	**0,64**<br>0,84	**0,64**						
N-acetylglucosamine kinase	Q9UJ70|NAGK_HUMAN				**0,79**<br>1,03	0,89<br>1,09	0,84<br>1,43			
N-acetylglucosamine-1-phosphodiester alpha-N-acetylglucosaminidase precursor	Q9UK23|NAGPA_HUMAN	0,00<br>1,09	0,00<br>**0,79**	0,00						
N-acetylglucosamine-1-phosphotransferase subunit gamma precursor	Q9UJJ9|GNPTG_HUMAN	0,95	**0,51**	0,67						
N-acetylglucosamine-1-phosphotransferase subunits alpha/beta precursor	Q3T906|GNPTA_HUMAN	1,00<br>**1,11**	**0,70**<br>0,94	0,79						
N-acetylglucosamine-6-sulfatase precursor	P15586|GNS_HUMAN	1,07<br>0,94	1,17<br>**0,86**	0,97	**1,40**<br>**1,56**	0,96<br>1,02	**1,38**<br>1,08			
N-acylethanolamine-hydrolyzing acid amidase precursor	Q02083|ASAHL_HUMAN	1,08	1,05	0,89						
N-acylneuraminate cytidylyltransferase	Q8NFW8|NEUA_HUMAN				1,92	**2,05**	2,17	0,00<br>0,39	0,00<br>**0,47**	0,00<br>**0,40**
N-acylsphingosine amidohydrolase (Acid ceramidase) 1 preproprotein isoform a variant (Fragment)	Q53H01|Q53H01_HUMAN				1,70	0,89	1,15			
NACHT, LRR and PYD domains-containing protein 6	P59044|NALP6_HUMAN	0,00	0,00							
NAD-dependent deacetylase sirtuin-5	Q9NXA8|SIRT5_HUMAN	0,98	1,19							
NAD-dependent malic enzyme, mitochondrial precursor	P23368|MAOM_HUMAN	**0,88**<br>1,02	**0,80**<br>1,01	**0,63**	2,69	2,24	**3,66**			
NADH dehydrogenase	Q549M5|Q549M5_HUMAN	0,98	0,98					1,11	**1,17**	**1,77**
NADH dehydrogenase (Ubiquinone) 1 beta subcomplex, 8, 19kDa	Q5W145|Q5W145_HUMAN							1,22	1,20	2,54
NADH dehydrogenase (Ubiquinone) flavoprotein 2, 24kDa	Q6IPW4|Q6IPW4_HUMAN	0,94	0,96							
NADH dehydrogenase (Ubiquinone) Fe-S protein 2, 49kDa	Q5VTW0|Q5VTW0_HUMAN	1,01<br>**0,79**	0,95<br>**0,75**	0,85						
NADH dehydrogenase (Ubiquinone) Fe-S protein 3, 30kDa (NADH-coenzyme Q reductase) variant (Fragment)	Q53FM7|Q53FM7_HUMAN	0,93	1,04	0,94				0,00<br>**1,73**	0,00<br>1,17	0,00<br>**1,96**
NADH dehydrogenase (Ubiquinone) Fe-S protein 8, 23kDa (NADH-coenzyme Q reductase) variant (Fragment)	Q53G17|Q53G17_HUMAN							1,05	1,07	1,60

NADH dehydrogenase (Ubiquinone) flavoprotein 3, 10kDa	Q8WU60|Q8WU60_HUMAN	0,78	**0,75**	**0,69**				0,99	1,17	**1,57**
		1,00	**0,88**		**1,81**	1,00	1,39	**1,81**	**2,28**	**4,85**
NADH dehydrogenase [ubiquinone] 1 alpha subcomplex	O95299|NDUAA_HUMAN									
subunit 10, mitochondrial precursor		0,87	**0,90**							
NADH dehydrogenase [ubiquinone] 1 alpha subcomplex	Q9UI09|NDUAC_HUMAN	0,77	**0,79**	**0,72**				0,94	0,97	**1,57**
subunit 12		1,05	0,93					0,00	0,00	0,00
NADH dehydrogenase [ubiquinone] 1 alpha subcomplex	Q9P0J0|NDUAD_HUMAN	0,90	**0,76**	**0,72**				0,96	1,00	**1,27**
subunit 13		0,98	0,97							
NADH dehydrogenase [ubiquinone] 1 alpha subcomplex	O43678|NDUA2_HUMAN	0,95	**0,73**	**0,68**				1,16	0,90	1,60
subunit 2		0,98	0,96					1,93	1,09	2,22
NADH dehydrogenase [ubiquinone] 1 alpha subcomplex	O00483|NDUA4_HUMAN	0,90	**0,70**	**0,51**						
subunit 4		1,09	0,96					**1,71**	0,90	**2,14**
NADH dehydrogenase [ubiquinone] 1 alpha subcomplex	Q16718|NDUA5_HUMAN									
subunit 5		1,01	0,99					**1,99**	**1,72**	**2,97**
NADH dehydrogenase [ubiquinone] 1 alpha subcomplex	P51970|NDUA8_HUMAN	1,00	**0,72**	**0,72**						
subunit 8		**0,91**	1,03							
NADH dehydrogenase [ubiquinone] 1 alpha subcomplex	Q16795|NDUA9_HUMAN	0,90	**0,76**	0,78						
subunit 9, mitochondrial precursor		0,98	0,96							
NADH dehydrogenase [ubiquinone] 1 beta subcomplex subunit	O75438|NDUB1_HUMAN									
1		**1,10**	**1,11**							
NADH dehydrogenase [ubiquinone] 1 beta subcomplex subunit	O96000|NDUBA_HUMAN	0,68	0,72	0,50				0,92	1,17	**1,63**
10		1,01	0,98					1,80	1,68	**3,18**
NADH dehydrogenase [ubiquinone] 1 beta subcomplex subunit	Q9NX14|NDUBB_HUMAN	0,00	0,00	0,00						
11, mitochondrial precursor					0,00	0,00	0,00			
NADH dehydrogenase [ubiquinone] 1 beta subcomplex subunit	O95178|NDUB2_HUMAN	1,05	0,69	0,64						
2, mitochondrial precursor										
NADH dehydrogenase [ubiquinone] 1 beta subcomplex subunit	O95168|NDUB4_HUMAN	1,01	0,92	0,82				1,27	1,23	1,39
4		1,03	0,96							
NADH dehydrogenase [ubiquinone] 1 beta subcomplex subunit	Q49SH3|Q49SH3_HUMAN	1,04	0,99	**0,74**				1,11	**1,58**	**2,10**
6										
NADH dehydrogenase [ubiquinone] 1 beta subcomplex subunit	O43674|NDUB5_HUMAN									
5, mitochondrial precursor		**0,86**	1,00							
NADH dehydrogenase [ubiquinone] 1 beta subcomplex subunit	O95139|NDUB6_HUMAN									
6		1,03	1,03					**1,71**	**2,17**	**3,40**
NADH dehydrogenase [ubiquinone] 1 beta subcomplex subunit	O95169|NDUB8_HUMAN	0,93	0,81	0,83				1,08	0,85	1,33
8, mitochondrial precursor		**1,21**	1,05							
NADH dehydrogenase [ubiquinone] 1 beta subcomplex subunit	Q9Y6M9|NDUB9_HUMAN	0,78	**0,67**	**0,62**						
9		1,04	**0,84**					0,00	0,00	0,00
NADH dehydrogenase [ubiquinone] iron-sulfur protein 3,	O75489|NDUS3_HUMAN									
mitochondrial precursor		1,07	0,88							
NADH dehydrogenase [ubiquinone] iron-sulfur protein 4,	O43181|NDUS4_HUMAN	0,92	1,02	0,84				0,83	**1,18**	1,51
mitochondrial precursor		0,94	0,89					**2,15**	1,51	**3,33**
NADH:ubiquinone oxidoreductase PSST subunit	Q7LD69|Q7LD69_HUMAN									
		0,97	0,97							
NADH-cytochrome b5 reductase 1	Q9UHQ9|NB5R1_HUMAN	0,95	1,02	0,92				1,17	1,00	1,14
		1,06	1,00					0,00	0,00	0,00
NADH-cytochrome b5 reductase 3	P00387|NB5R3_HUMAN	1,02	0,94	0,99	1,12	1,11	1,01	1,65	2,76	4,91
		0,98	1,05					0,00	0,00	0,00
NADH-ubiquinone oxidoreductase 75 kDa subunit,	P28331|NDUS1_HUMAN	0,92	0,87	**0,80**						
mitochondrial precursor		**0,88**	**0,83**							
NADH-ubiquinone oxidoreductase chain 3	A4ZN04|A4ZN04_HUMAN									
		0,00	0,00							
NADH-ubiquinone oxidoreductase chain 4	Q305G4|Q305G4_HUMAN	1,05	1,22	0,84						
NADP-dependent malic enzyme	P48163|MAOX_HUMAN				1,02	0,91	1,04			
					0,98	1,21	1,18			
Nascent polypeptide-associated complex subunit alpha	Q13765|NACA_HUMAN							0,00	0,00	0,00
					0,00	0,00	0,00			
NCF4 protein	Q6FGM9|Q6FGM9_HUMAN									
					0,62	**0,60**	0,64			
NCK-associated protein 1-like	Q52LW0|Q52LW0_HUMAN									
								1,00	0,92	0,89
NDUFA3 protein	Q6FGG4|Q6FGG4_HUMAN	1,03	**0,73**	**0,64**						
		0,88	0,89							
NDUFA6 protein (Fragment)	Q6FGW0|Q6FGW0_HUMAN	0,86	0,88	**0,64**						
		1,05	0,95							
NDUFA7 protein	Q6IB89|Q6IB89_HUMAN									
								**2,15**	1,61	**2,85**
NDUFA7 protein (Fragment)	P52815|RM12_HUMAN							**1,29**	**1,24**	**1,82**
NDUFA7 protein (Fragment)	Q32Q14|Q32Q14_HUMAN									
		1,09	1,00							
NDUFB3 protein	Q6IB80|Q6IB80_HUMAN	1,08	**0,77**	0,70				0,94	1,17	**1,60**
		1,05	0,93					**3,94**	1,25	**3,36**
NDUFB5 protein	Q68DI0|Q68DI0_HUMAN	0,93	0,96	0,80				1,04	**1,89**	**2,11**
								**3,75**	1,40	**3,97**
NDUFB7 protein (Fragment)	Q6ICN9|Q6ICN9_HUMAN	0,86	**0,67**	**0,62**				0,90	**0,55**	0,77
		1,03	1,07							
NDUFC2 protein	Q6FIH8|Q6FIH8_HUMAN	0,99	1,05	0,82						
NDUFS5 protein	Q6ICQ4|Q6ICQ4_HUMAN	0,86	**0,68**	**0,71**				1,18	1,27	**1,92**
NDUFS5 protein	Q6IBA0|Q6IBA0_HUMAN									
		0,85	0,98							
NDUFS6 protein	Q6IBC4|Q6IBC4_HUMAN	1,09	1,03	0,99				0,85	0,89	1,18
		0,99	**0,86**							
NDUFV1 protein	Q6IBR3|Q6IBR3_HUMAN	1,00	0,93	0,79						
		0,96	1,00							
NEDD8 precursor	Q15843|NEDD8_HUMAN									
					0,82	0,88	0,89			
NEDD8-activating enzyme E1 regulatory subunit	Q13564|ULA1_HUMAN				0,00	0,00	0,00			
NEDD8-conjugating enzyme Ubc12	P61081|UBC12_HUMAN									
					**0,60**	0,81	0,41			
NEDD9-interacting protein with calponin homology and LIM	Q8TDZ2|MICA1_HUMAN				1,03	1,99	1,29			
domains										
Neighbor of COX4	Q53Y03|Q53Y03_HUMAN							1,44	**2,51**	**3,75**
		1,02	1,19					0,68	1,88	2,83
Nesprin-1	Q8NF91|SYNE1_HUMAN				1,11	1,01	0,94			
		1,10	**1,19**							
Nestin	P48681|NEST_HUMAN				**1,68**	**1,49**	**1,59**			
					**1,91**	1,60	**1,90**			
Neurabin-2	Q96SB3|NEB2_HUMAN							0,87	0,88	0,58
								1,11	0,40	0,87
Neuraminidase	P03483|NRAM_I72A2	**5,47**	**11,93**	**10,54**				**3,71**	**3,86**	**7,23**
		**1,84**	**5,34**		**3,04**	**6,44**	8,86	**9,93**	**12,07**	**12,89**
Neuroblast differentiation-associated protein AHNAK	Q09666|AHNK_HUMAN	**1,70**	**1,18**	**1,82**	**1,64**	**1,07**	0,97	**1,30**	**0,49**	**0,44**
		1,91	**1,33**		1,16	0,93	0,86	1,75	**0,94**	**1,13**
Neurofilament, medium polypeptide 150kDa	Q4QRK6|Q4QRK6_HUMAN									
								1,26	0,88	0,66
Neurogranin	Q92686|NEUG_HUMAN				**0,73**	0,78	**0,42**			
					0,65	0,51	0,17			
Neurolysin, mitochondrial precursor	Q9BYT8|NEUL_HUMAN	**0,80**	0,60	**0,37**						
		0,95	0,88							
Neuronal protein	Q7Z4X2|Q7Z4X2_HUMAN									
		1,05	0,86					1,74	**1,90**	2,36
Neuropathy target esterase	Q8IY17|PLPL6_HUMAN	1,11	1,10	1,16						
		1,07	**1,34**					0,99	1,39	1,55
Neuropilin 1	Q5T7F3|Q5T7F3_HUMAN	1,07	**0,80**	0,90						

		1,17	0,90							
Neuroplastin precursor	Q9Y639|NPTN_HUMAN	1,09	0,88	0,93	1,62	0,80	1,40	0,00	0,00	8,56<br>0,00
Neutral alpha-glucosidase AB precursor	Q14697|GANAB_HUMAN	**1,13**<br>**0,88**	**1,15**<br>1,06	**1,13**	**1,89**<br>**2,49**	1,30<br>**1,57**	**2,29**<br>**2,37**			
Neutral amino acid transporter	Q71UA6|Q71UA6_HUMAN	1,26<br>1,03	1,22<br>0,83	**1,33**						
Neutrophil cytosol factor 2	P19878|NCF2_HUMAN	1,03	**4,08**	**8,80**	0,88	**0,80**	**0,52**			
Neutrophil cytosol factor 2 variant (Fragment)	Q59F14|Q59F14_HUMAN				**0,86**	0,77	**0,49**			
Neutrophil cytosolic factor 3; NCF-3 (Fragments)	Q9UDI2|Q9UDI2_HUMAN	0,00	0,00							
NHP2L1 protein	Q6FHM6|Q6FHM6_HUMAN	0,00<br>0,75	0,00<br>0,91	0,00				0,96<br>0,76	**0,80**<br>**0,73**	**0,45**<br>**0,65**
Nicalin precursor	Q969V3|NCLN_HUMAN	1,01<br>**0,80**	1,04<br>0,97	1,01				0,90	**3,58**	**6,86**
Nicastrin precursor	Q92542|NICA_HUMAN	**0,71**<br>0,94	0,79<br>0,95	0,86						
Nicotinamide nucleotide transhydrogenase	Q2TB59|Q2TB59_HUMAN	0,99<br>0,97	**0,91**<br>**0,94**	**0,83**	1,14	1,12	1,13			
Nicotinate phosphoribosyltransferase	Q6XQN6|PNCB_HUMAN				0,00	0,00	0,00			
Niemann-Pick disease, type C1 variant (Fragment)	Q59GR1|Q59GR1_HUMAN	0,99<br>0,97	1,01<br>0,95	0,89						
Niemann-Pick disease, type C2 variant (Fragment)	Q53HV6|Q53HV6_HUMAN							0,96	**1,77**	1,45
Nitrilase homolog 2	Q9NQR4|NIT2_HUMAN	1,02	1,02		0,90	1,07	0,99			
NLR family member X1	Q86UT6|NLRX1_HUMAN	0,83	0,64	0,44						
Nodal modulator 1 precursor	Q15155|NOMO1_HUMAN							1,02	**2,42**	**7,44**
Nodal modulator 2 precursor	Q5JPE7|NOMO2_HUMAN							0,69	**1,91**	**2,89**
Nodal modulator 3 precursor	P69849|NOMO3_HUMAN	1,02<br>0,95	0,97<br>1,10	0,88						
NOGO-interacting mitochondrial protein	Q96RL8|Q96RL8_HUMAN	0,82	0,81							
NOL1 protein	Q3KQS4|Q3KQS4_HUMAN							0,85<br>1,11	**0,62**<br>1,06	**0,42**<br>0,99
Non-histone chromosomal protein HMG-14	P05114|HMGN1_HUMAN				0,86<br>0,53	**1,88**<br>2,35	**1,65**<br>2,70	1,05<br>**1,24**	**0,45**<br>1,06	**0,35**<br>1,07
Non-histone chromosomal protein HMG-17	P05204|HMGN2_HUMAN				0,88	3,41	**4,18**	0,78<br>**1,19**	**0,28**<br>0,96	**0,23**<br>0,99
Non-muscle myosin heavy polypeptide 9	Q60FE2|Q60FE2_HUMAN	1,00<br>**1,20**	**1,15**<br>1,64	**1,75**	**1,11**<br>1,26	**1,12**<br>1,44	**1,36**<br>1,76	**2,54**<br>**0,63**	**6,30**<br>5,18	**7,50**<br>4,52
Non-structural protein 1	P03495|NS1_I72A2	**1,96**<br>**1,69**	**7,36**<br>**5,84**	**9,43**	**3,16**<br>2,70	**10,23**<br>7,25	**6,47**<br>4,22	**18,22**<br>**10,96**	**10,22**<br>**3,70**	**3,71**<br>**3,28**
Normal mucosa of esophagus-specific gene 1 protein	Q9C002|NMES1_HUMAN	0,97<br>0,97	1,06<br>0,92	0,71						
Novel enoyl-CoA hydratase/isomerase family protein (Fragment)	Q5TEF9|Q5TEF9_HUMAN	1,08	1,13							
Novel protein	Q5JTA8|Q5JTA8_HUMAN	1,05<br>0,93	1,02<br>1,08	1,01				1,14	**1,79**	**2,95**
Novel protein similar to ATP synthase	Q5VTU8|Q5VTU8_HUMAN	0,87	0,99	0,91				0,60	3,51	**5,74**
Novel protein similar to prothymosin alpha PTMA	Q5VYG3|Q5VYG3_HUMAN				**0,68**	**0,50**	**0,36**			
Novel protein similar to ribosomal protein 26 RPS26	Q5JVH5|Q5JVH5_HUMAN	0,81	1,28					0,89	0,32	0,64
Novel protein similar to ribosomal protein L29 RPL29 (Fragment)	Q5T1D1|Q5T1D1_HUMAN	0,00	0,00		1,01	0,96	1,06			
NS5ATP1	Q546Z2|Q546Z2_HUMAN	0,97<br>1,06	0,92<br>0,98	**0,67**	1,71	1,36	1,43			
NSFL1 cofactor p47	Q9UNZ2|NSF1C_HUMAN				0,71	0,79	0,66			
NSFL1 (P97) cofactor	Q5JXA4|Q5JXA4_HUMAN				0,00	0,00	0,00			
N-terminal acetyltransferase complex ARD1 subunit homolog A	P41227|ARD1A_HUMAN				0,73	0,91	0,90			
Nuclear cap-binding protein subunit 2	P52298|NCBP2_HUMAN							0,81<br>0,89	**0,48**<br>0,68	**0,23**<br>**0,38**
Nuclear export protein	Q0A2H1|NEP_I59A0					88,55	46,43			
Nuclear export protein	P69258|NEP_I72A2	1,65	3,73	3,89	4,34	**12,25**	**6,09**	**9,43**	**6,24**	**4,34**
Nuclear fragile X mental retardation-interacting protein 2	Q7Z417|NUFP2_HUMAN							0,88	0,60	0,47
Nuclear migration protein nudC	Q9Y266|NUDC_HUMAN				0,94<br>**0,61**	**0,83**<br>0,64	0,90<br>**0,61**			
Nuclear pore complex protein Nup205	Q92621|NU205_HUMAN							1,36	1,21	2,12
Nuclear pore complex protein Nup88	Q99567|NUP88_HUMAN							0,40	0,91	0,84
Nuclear pore complex protein Nup93	Q8N1F7|NUP93_HUMAN							**0,46**	**0,72**	**0,63**
Nuclear pore complex protein Nup98-Nup96 precursor	P52948|NUP98_HUMAN							0,91<br>**0,68**	**0,58**<br>1,14	**0,38**<br>**0,72**
Nuclear pore glycoprotein p62	P37198|NUP62_HUMAN							0,83	1,24	0,72
Nuclear pore membrane glycoprotein 210 precursor	Q8TEM1|PO210_HUMAN	1,11<br>**0,81**	1,02<br>**0,85**	1,10						
Nuclear protein Hcc-1	P82979|HCC1_HUMAN				0,00	0,00	0,00	1,01<br>**1,37**	**0,25**<br>**0,39**	**0,17**<br>**0,37**
Nuclear protein localization protein 4 homolog	Q8TAT6|NPL4_HUMAN				0,00	0,00	0,00			
Nuclear protein NP60	Q49A26|NP60_HUMAN							0,94	0,48	**0,43**
Nuclear receptor coactivator 5	Q9HCD5|NCOA5_HUMAN							0,49	0,33	**0,19**
Nuclear transport factor 2	P61970|NTF2_HUMAN				0,95	1,04	1,00			
Nuclear ubiquitous casein and cyclin-dependent kinases substrate	Q9H1E3|NUCKS_HUMAN				**0,69**<br>**0,41**	1,00<br>0,94	0,87<br>0,86		8,49<br>10,22	7,48<br>12,00
Nuclease-sensitive element-binding protein 1	Q7KZ24|Q7KZ24_HUMAN				0,00	0,00	0,00	0,99	1,26	1,15
Nuclease-sensitive element-binding protein 1	P67809|YBOX1_HUMAN	1,05	1,28					0,91	**1,58**	**1,33**
Nucleobindin 1 variant (Fragment)	Q53GX6|Q53GX6_HUMAN	1,05<br>**1,21**	**0,57**<br>**0,90**	**0,63**	1,33<br>**1,74**	1,02<br>1,12	**1,67**<br>1,26			
Nucleobindin-2 precursor	P80303|NUCB2_HUMAN	**1,28**	0,89	1,04						
Nucleolar and coiled-body phosphoprotein 1	Q9BUV3|Q9BUV3_HUMAN				1,06	1,28	1,41	0,93<br>**1,15**	**0,55**<br>1,06	**0,24**<br>**0,73**
Nucleolar complex protein 2 homolog	Q9Y3T9|NOC2L_HUMAN							0,38	0,71	0,50
Nucleolar complex protein 3 homolog	Q8WTT2|NOC3L_HUMAN							**0,72**	0,83	0,79

Nucleolar protein 5	Q9Y2X3|NOL5_HUMAN	0,00	0,00	0,00				0,66	0,46	0,46
								**0,52**	0,59	0,45
Nucleolar protein 5A	O00567|NOL5A_HUMAN							0,90	**0,63**	**0,51**
								**0,49**	**0,73**	**0,62**
Nucleolar protein 6	Q9H6R4|NOL6_HUMAN									
								0,22	**0,41**	**0,34**
Nucleolar RNA helicase 2	Q9NR30|DDX21_HUMAN							0,91	0,69	0,87
								**0,34**	**0,62**	**0,57**
Nucleolar transcription factor 1	P17480|UBF1_HUMAN							**0,83**	**0,44**	**0,37**
Nucleolin	P19338|NUCL_HUMAN	0,98	1,03	**0,88**	**1,14**	**1,42**	**1,19**	1,03	**0,41**	**0,26**
		**1,49**	1,15		1,01	1,38	1,07	**1,49**	**0,73**	**0,65**
Nucleophosmin	P06748|NPM_HUMAN	1,03	**1,16**	**1,92**	**1,51**	**1,86**	**1,31**	**0,78**	**0,47**	**0,32**
		1,04	**1,30**		1,31	1,99	1,48	**0,89**	**0,84**	**0,67**
Nucleoporin 153kDa	Q7Z743|Q7Z743_HUMAN									
								**0,52**	0,98	**0,33**
Nucleoporin 54kDa variant (Fragment)	Q53FD7|Q53FD7_HUMAN							0,79	0,63	0,36
Nucleoporin NUP53	Q8NFH5|NUP53_HUMAN									
								**0,58**	0,93	**0,39**
Nucleoporin-like protein RIP	P52594|AGFG1_HUMAN							0,00	0,00	0,00
					0,88	1,03	**0,71**			
Nucleoprotein	P06827|NCAP_I72A2	**1,72**	**6,74**	**12,25**	**1,86**	**9,25**	**8,92**	**12,80**	**6,45**	**7,08**
		**1,24**	**5,02**		2,28	7,19	7,15	**9,26**	**7,71**	**10,10**
Nucleoside diphosphate kinase	Q32Q12|Q32Q12_HUMAN	0,72	1,36	1,84	0,85	0,83	0,82	0,79	**1,63**	**1,70**
		2,24	2,18			0,89	0,85	**0,82**	**1,39**	**1,79**
Nucleoside diphosphate kinase	Q86XQ2|Q86XQ2_HUMAN				0,00	0,00	0,00			
		2,05	1,75							
Nucleoside diphosphate kinase (Fragment)	Q9NUF9|Q9NUF9_HUMAN	1,19	**1,13**	1,77						
		0,95	0,88					0,00	0,00	0,00
Nucleoside diphosphate kinase (Fragment)	Q53HM5|Q53HM5_HUMAN									
		1,19	1,03							
Nucleoside diphosphate-linked moiety X motif 6	P53370|NUDT6_HUMAN									
		0,90	0,81							
Nucleosome assembly protein 1-like 1	P55209|NP1L1_HUMAN				**0,78**	**0,83**	0,89			
Nucleosome assembly protein 1-like 4	Q99733|NP1L4_HUMAN				0,93	0,93	0,93			
					0,66	**0,74**	0,80			
Nucleotide exchange factor SIL1 precursor	Q9H173|SIL1_HUMAN	0,97	0,70	0,50						
Nudix (Nucleoside diphosphate linked moiety X)-type motif 1	Q8IV95|Q8IV95_HUMAN				0,87	0,69	0,66			
					0,67	0,75	0,62			
NUMA1 variant protein (Fragment)	Q4LE64|Q4LE64_HUMAN							0,88	**0,72**	**0,38**
					0,72	0,85	1,02	**0,65**	**0,70**	**0,68**
Nurim	Q8IXM6|NRM_HUMAN							0,79	1,03	0,55
OAT protein	Q6IAV9|Q6IAV9_HUMAN	**0,80**	**0,80**	**0,64**						
		**0,88**	0,96							
Obg-like ATPase 1	Q9NTK5|OLA1_HUMAN									
					0,00	0,00	0,00			
OCIA domain-containing protein 1	Q9NX40|OCAD1_HUMAN	**0,72**	0,80	0,88				0,90	1,14	**1,36**
		0,94	0,97					1,12	1,33	1,74
OGFR protein	Q05BV5|Q05BV5_HUMAN				0,00	0,00	0,00			
					0,00	0,00	0,00			
Optic atrophy 3 protein	Q9H6K4|OPA3_HUMAN	0,99	1,01	0,92						
		0,96	0,99							
ORM1-like protein 2	Q53FV1|ORML2_HUMAN	1,25	1,41	1,09						
Osteoclast-stimulating factor 1	Q92882|OSTF1_HUMAN				0,87	0,89	**0,77**			
					1,00	**0,87**	0,72			
Osteopetrosis-associated transmembrane protein 1 precursor	Q86WC4|OSTM1_HUMAN	1,17	1,35	0,90				1,65	**6,40**	**7,73**
OS-9 protein	Q6IBL2|Q6IBL2_HUMAN									
		0,98	1,10							
OTTHUMP00000016816	Q5TCG3|Q5TCG3_HUMAN									
					**1,73**	**1,37**	1,24			
Overexpressed breast tumor protein	Q96B49|OBTP_HUMAN									
		0,00	0,00							
OXCT protein	Q6IAV5|Q6IAV5_HUMAN									
					0,00	0,00	0,00			
Oxidoreductase HTATIP2	Q9BUP3|HTAI2_HUMAN	1,16	1,25	1,17						
		0,97	0,92							
Oxidoreductase NAD-binding domain-containing protein 1 precursor	Q96HP4|OXND1_HUMAN									
		1,03	0,95							
Oxysterol-binding protein-related protein 8	Q9BZF1|OSBL8_HUMAN	1,09	**1,44**	**1,82**						
p180/ribosome receptor	A7BI36|A7BI36_HUMAN	**0,60**	0,96	**1,30**	**1,91**	0,90	1,08	0,88	**1,79**	**2,03**
		**1,17**	**1,20**		1,48	**0,87**	**0,82**	0,91	**1,73**	**1,77**
P2X purinoceptor 4	Q99571|P2RX4_HUMAN	1,01	1,04	1,03						
		0,98	0,99							
P2X purinoceptor 7	Q99572|P2RX7_HUMAN									
		1,11	1,10							
P37 AUF1	Q12771|Q12771_HUMAN				**1,33**	**1,83**	1,32	0,00	0,00	0,00
					1,18	1,20	1,04			
PA2G4 protein (Fragment)	Q05D08|Q05D08_HUMAN				0,85	0,97	**0,79**			
					0,89	**0,87**	**0,66**	**0,48**	0,86	**0,73**
PAFAH1B2 protein	Q6IBR6|Q6IBR6_HUMAN				0,88	0,98	0,97			
PAFAH1B3 protein (Fragment)	Q53X88|Q53X88_HUMAN									
					0,71	0,74	0,62			
PAPSS1 protein	Q6IAX6|Q6IAX6_HUMAN				0,90	**1,24**	0,96			
					**0,86**	1,20	0,79			
Paraplegin	Q9UQ90|SPG7_HUMAN	0,83	1,09	0,90						
		1,01	0,85							
Paraspeckle component 1	Q8WXF1|PSPC1_HUMAN							1,03	**0,27**	**0,16**
								**1,28**	**0,40**	**0,27**
Parathymosin	P20962|PTMS_HUMAN				1,04	1,17	0,90			
					**0,70**	**0,82**	**0,72**			
PC cell-derived growth factor	Q540U8|Q540U8_HUMAN	1,04	0,90	0,76				0,84	0,75	1,44
		0,79	0,61							
PC4 and SFRS1-interacting protein	O75475|PSIP1_HUMAN							**0,74**	**0,45**	**0,33**
								1,05	0,89	0,82
PC4 protein	Q6IBA2|Q6IBA2_HUMAN									
								**1,31**	**3,86**	**5,63**
PCK2 protein	Q6IB91|Q6IB91_HUMAN	0,96	**0,86**	**0,63**	**2,54**	**1,82**	**2,85**			
		1,02	**0,83**		**1,81**	1,15	**1,83**			
PDCD6IP protein	Q6NUS1|Q6NUS1_HUMAN	0,99	1,39	**2,29**						
		0,99	1,12		0,86	0,96	0,98			
PDZ and LIM domain protein 4	P50479|PDLI4_HUMAN									
					0,65	0,78	**0,60**			
PDZ and LIM domain protein 5	Q96HC4|PDLI5_HUMAN									
					0,54	**0,60**	0,38			
PDZ and LIM domain protein 7	Q9NR12|PDLI7_HUMAN							0,83	0,63	0,18
					0,75	0,88	**0,59**	0,80	0,88	**0,55**
PEA15 protein	Q6FHL9|Q6FHL9_HUMAN				**0,79**	**0,53**	**0,38**			
PECI protein	Q6IBN4|Q6IBN4_HUMAN	1,54	**1,29**	1,34				0,83	1,65	**3,18**
		**0,80**	0,95							
Peflin	Q9UBV8|PEF1_HUMAN	0,00	0,00	0,00	0,92	1,21	1,52	2,19	1,92	1,67

					1,29	1,02	1,00	1,44	2,93	3,23
Pentatricopeptide repeat-containing protein 3, mitochondrial precursor	Q96EY7|PTCD3_HUMAN	0,85<br>0,92	0,94<br>**0,91**	0,82						
Peptidase (Mitochondrial processing) alpha	Q5SXM9|Q5SXM9_HUMAN	0,88<br>**0,87**	0,86<br>**0,84**	**0,67**						
Peptide/histidine transporter	Q8N697|Q8N697_HUMAN	0,92<br>0,83	0,97<br>0,86	1,25						
Peptide methionine sulfoxide reductase	Q9UJ68|MSRA_HUMAN	1,09	1,07		**0,72**	0,93	**0,54**			
Peptidyl-prolyl cis-trans isomerase	Q9BWS9|CHID1_HUMAN	0,96	1,05	**0,93**	0,94	0,95	0,64	**0,92**	**1,59**	**1,84**
Peptidyl-prolyl cis-trans isomerase	Q2NKQ6|Q2NKQ6_HUMAN	0,95	1,14	1,24	**1,90**	**1,22**	**2,10**	0,78	0,51	0,25
Peptidyl-prolyl cis-trans isomerase	Q53XJ5|Q53XJ5_HUMAN	0,97	0,93	0,90						
Peptidyl-prolyl cis-trans isomerase	Q6IBH5|Q6IBH5_HUMAN				**1,70**	1,15	**1,84**			
Peptidyl-prolyl cis-trans isomerase	Q6FGM6|Q6FGM6_HUMAN				0,78	0,67	**0,47**			
Peptidyl-prolyl cis-trans isomerase	Q0VDC6|Q0VDC6_HUMAN				**0,59**	0,88	0,50			
Peptidyl-prolyl cis-trans isomerase	A8K534|A8K534_HUMAN	0,99	**1,19**							
Peptidyl-prolyl cis-trans isomerase	Q71V99|Q71V99_HUMAN	**1,21**	**1,13**					**0,72**	0,96	**0,67**
Peptidyl-prolyl cis-trans isomerase	Q53XJ5|Q53XJ5_HUMAN	0,79	0,97							
Peptidyl-prolyl cis-trans isomerase, mitochondrial precursor	P30405|PPIF_HUMAN	0,99	1,04							
Peptidyl-prolyl cis-trans isomerase A	P62937|PPIA_HUMAN				**0,78**<br>0,68	0,89<br>0,79	**0,82**<br>0,67	**1,43**	1,51	1,34
Peptidyl-prolyl cis-trans isomerase B precursor	P23284|PPIB_HUMAN							0,95	**1,94**	**2,56**
Peptidyl-prolyl cis-trans isomerase G	Q13427|PPIG_HUMAN							**0,66**	**0,58**	0,45
Peptidyl-prolyl cis-trans isomerase-like 3	Q9H2H8|PPIL3_HUMAN				0,91	1,05	1,65			
Peptidyl-prolyl cis-trans isomerase, mitochondrial precursor	P30405|PPIF_HUMAN	**0,82**	**0,81**	**0,48**	**1,91**	**1,66**	**2,18**	0,94<br>**0,70**	0,98<br>0,95	1,18<br>1,07
Peripheral benzodiazepine receptor	Q13850|Q13850_HUMAN	**0,82**<br>0,93	0,96<br>**0,45**	**0,83**						
Periphilin-1	Q8NEY8|PPHLN_HUMAN							0,86<br>1,06	**0,22**<br>**0,35**	**0,12**<br>**0,34**
Peroxiredoxin 3 isoform a variant (Fragment)	Q53HC2|Q53HC2_HUMAN							**1,05**	3,87	**7,40**
Peroxiredoxin-1	Q06830|PRDX1_HUMAN	**1,33**<br>**1,98**	**2,24**<br>**1,67**	**3,91**	**0,84**<br>0,76	**0,83**<br>0,81	**0,80**<br>0,76	**2,82**<br>**1,62**	**1,65**<br>**0,86**	0,97<br>**0,60**
Peroxiredoxin-2	P32119|PRDX2_HUMAN				0,87<br>0,91	0,93<br>0,87	0,94<br>1,01			
Peroxiredoxin-4	Q13162|PRDX4_HUMAN	1,07<br>0,97	**1,23**<br>1,04	**1,26**						
Peroxiredoxin-5, mitochondrial precursor	P30044|PRDX5_HUMAN	0,82<br>1,01	0,92<br>0,90	0,70	0,00<br>0,90	0,00<br>0,91	0,00<br>0,63			
Peroxiredoxin-6	P30041|PRDX6_HUMAN				**0,80**<br>0,88	0,90<br>1,07	**0,81**<br>0,93			
Peroxisomal bifunctional enzyme	Q08426|ECHP_HUMAN							0,00<br>0,00	0,00<br>0,00	0,00<br>0,00
Peroxisomal long-chain acyl-coA thioesterase variant (Fragment)	Q53EK4|Q53EK4_HUMAN	0,88<br>1,05	**0,75**<br>**0,93**	**0,45**						
Peroxisomal membrane protein 11B	O96011|PX11B_HUMAN	1,05<br>**0,81**	0,96<br>0,99	0,81						
Peroxisomal membrane protein PEX14	O75381|PEX14_HUMAN	0,80<br>0,95	0,86<br>1,05	0,71				0,60	0,78	0,65
Peroxisomal membrane protein PEX16	Q9Y5Y5|PEX16_HUMAN	0,74	0,84	0,63						
Peroxisomal multifunctional enzyme type 2	P51659|DHB4_HUMAN	0,97<br>**0,94**	0,88<br>0,98	**0,73**	**1,67**<br>**1,60**	1,15<br>1,11	**1,62**<br>**1,39**	**1,43**<br>**0,56**	**2,43**<br>**2,03**	**4,58**<br>**3,07**
PERQ amino acid-rich with GYF domain-containing protein 2	Q6Y7W6|PERQ2_HUMAN				2,99	1,51	2,36			
PEST proteolytic signal-containing nuclear protein	Q8WW12|PCNP_HUMAN				0,00<br>0,96	0,00<br>0,97	0,00<br>0,94	1,00<br>1,12	0,91<br>1,53	0,62<br>1,86
PET112-like variant (Fragment)	Q53GP4|Q53GP4_HUMAN	1,22<br>0,84	0,90<br>0,89	0,99						
PEX3 protein (Fragment)	Q6FGP5|Q6FGP5_HUMAN	0,95	1,10							
PGRMC1 protein	Q6IB11|Q6IB11_HUMAN	0,85<br>1,02	1,00<br>1,06	1,02				1,15	**4,68**	**7,62**
PGS1 protein (Fragment)	Q8TA85|Q8TA85_HUMAN	0,84	0,93							
PHB protein (Fragment)	Q6FHP5|Q6FHP5_HUMAN	0,96	**0,92**	**0,84**						
PHD finger-like domain-containing protein 5A	Q7RTV0|PHF5A_HUMAN							0,87<br>**0,75**	**0,50**<br>**0,69**	**0,31**<br>**0,52**
Phenylalanyl-tRNA synthetase alpha chain	Q9Y285|SYFA_HUMAN				1,12	1,14	1,22	0,68	**1,27**	0,96
Phenylalanyl-tRNA synthetase beta chain	Q9NSD9|SYFB_HUMAN				**0,80**	1,06	1,13			
Phenylalanyl-tRNA synthetase, mitochondrial precursor	O95363|SYFM_HUMAN	0,00<br>0,84	0,00<br>0,93	0,00						
Phosphatase and actin regulator 3	B1AKX0|B1AKX0_HUMAN	1,00	0,95							
Phosphatidate cytidylyltransferase 2	O95674|CDS2_HUMAN	1,14<br>0,86	1,13<br>1,20	1,10						
Phosphatidylethanolamine-binding protein 1	P30086|PEBP1_HUMAN				**0,74**<br>0,82	0,94<br>0,94	0,89<br>0,77			
Phosphatidylinositide phosphatase SAC1	Q9NTJ5|SAC1_HUMAN	1,14<br>1,00	**1,27**<br>1,05	**1,37**						
Phosphatidylinositol 4-kinase type 2-alpha	Q9BTU6|P4K2A_HUMAN	0,97<br>**0,88**	**0,88**<br>**0,88**	**0,79**						
Phosphatidylinositol binding clathrin assembly protein	Q6GMQ6|Q6GMQ6_HUMAN				0,81	1,03	0,81	**0,70**	**0,61**	**0,51**
Phosphatidylinositol-5-phosphate 4-kinase type-2 alpha	P48426|PI42A_HUMAN	3,92<br>1,38	1,00	5,24						
Phosphatidylserine decarboxylase proenzyme	Q9UG56|PISD_HUMAN	0,91	0,93							
Phosphatidylserine synthase 2	Q9BVG9|PTSS2_HUMAN	0,00	0,00	0,00						
Phosphofructokinase, platelet	Q5VSR7|Q5VSR7_HUMAN				**0,81**	0,87	**0,53**			
Phosphofructokinase-P (Fragment)	O14943|O14943_HUMAN	2,14	1,98	2,66						
Phosphoglucomutase-1	P36871|PGM1_HUMAN				0,92<br>1,11	0,90<br>1,11	1,03<br>1,10			
Phosphoglucomutase-2	Q96G03|PGM2_HUMAN				1,03<br>0,90	**1,14**<br>1,09	1,00<br>1,14			
Phosphoglycerate dehydrogenase	Q5SZU1|Q5SZU1_HUMAN				1,18	0,93	0,52			
Phosphoglycerate kinase 1	P00558|PGK1_HUMAN	1,27<br>**1,61**	**1,69**<br>**1,55**	**2,13**	**0,82**<br>0,78	0,98<br>1,03	**0,92**<br>0,95	1,12<br>**0,68**	1,34<br>0,90	1,71<br>**0,82**

Phosphoglycerate mutase 1	Q6P6D7|Q6P6D7_HUMAN				**0,87**	**0,87**	0,92			
Phosphoglycerate mutase 2	A4D2J6|A4D2J6_HUMAN				1,07	1,08	1,05			
Phosphoglycerate mutase family member 5 precursor	Q96HS1|PGAM5_HUMAN	1,09<br>0,91	0,99<br>**0,88**	0,85				0,87	1,63	1,31
Phosphoglycolate phosphatase	A6NDG6|PGP_HUMAN				0,97<br>1,01	0,77<br>**0,67**	0,87<br>0,78			
Phospholipase A2, group VII	Q5VTT1|Q5VTT1_HUMAN	0,00	0,00	0,00						
Phospholipase D1 variant (Fragment)	Q59EA4|Q59EA4_HUMAN	0,93	0,94							
Phospholipase D3	Q8IV08|PLD3_HUMAN	1,00<br>0,94	**0,76**<br>**0,71**	**0,58**	**2,07**<br>1,36	0,74<br>**0,52**	**0,75**<br>**0,70**			
Phosphomannomutase 2	O15305|PMM2_HUMAN				0,66	0,82	0,74			
Phosphopantothenate--cysteine ligase	Q9HAB8|PPCS_HUMAN				0,96<br>0,53	1,12<br>0,76	1,22<br>0,88			
Phosphoprotein associated with glycosphingolipid-enriched microdomains 1	Q9NWQ8|PAG1_HUMAN							0,00	0,00	0,00
Phosphoprotein enriched in astrocytes 15	B1AKZ3|B1AKZ3_HUMAN				**0,51**	**0,30**	0,20			
Phosphoribosylglycinamide formyltransferase, phosphoribosylglycinamide synthetase,	Q59HH3|Q59HH3_HUMAN	0,91	1,30	2,49	0,95	0,87	**0,67**			
Phosphorylase (Fragment)	Q59GM9|Q59GM9_HUMAN				0,79<br>0,96	0,90<br>1,02	0,82<br>0,95			
Phostensin	Q6NYC8|PHTNS_HUMAN				**1,37**<br>0,84	1,19<br>0,98	**1,36**<br>1,08	**1,59**	**0,60**	**0,44**
PICALM variant protein (Fragment)	Q4LE54|Q4LE54_HUMAN	0,92	**0,50**	**0,54**	0,00	0,00	0,00	0,90	**0,35**	**0,40**
PIK4CA variant protein (Fragment)	Q4LE69|Q4LE69_HUMAN	1,02	1,10							
Pinin	Q9H307|PININ_HUMAN							**0,80**<br>1,02	**0,55**<br>0,92	**0,24**<br>**0,60**
PITSLRE serine/threonine-protein kinase CDC2L1	P21127|CD2L1_HUMAN							1,06<br>1,23	0,64<br>0,82	0,50<br>0,84
Pituitary tumor-transforming gene 1 protein-interacting protein precursor	P53801|PTTG_HUMAN	1,04	0,84							
Placenta apolipoprotein B48 receptor type 2	Q9NS13|Q9NS13_HUMAN	**0,74**	0,86	1,27						
Plasma alpha-L-fucosidase precursor	Q9BTY2|FUCO2_HUMAN	1,07	0,99							
Plasma glutamate carboxypeptidase precursor	Q9Y646|PGCP_HUMAN	1,36<br>0,82	1,75<br>0,84	1,60	2,29	2,00	2,27			
Plasma membrane calcium-transporting ATPase 1	P20020|AT2B1_HUMAN	1,10<br>1,17	1,09<br>**1,27**	**1,23**						
Plasminogen activator inhibitor 1 RNA-binding protein	Q8NC51|PAIRB_HUMAN				0,97<br>0,74	**0,79**<br>0,75	**0,84**<br>**0,77**	**0,80**<br>1,21	0,96<br>**1,32**	**0,62**<br>1,07
Platelet endothelial cell adhesion molecule precursor	P16284|PECA1_HUMAN	1,01	0,76	0,61	1,84	0,88	0,93			
Platelet glycoprotein 4	P16671|CD36_HUMAN	**1,26**	**0,75**	**0,83**						
Platelet receptor Gi24 precursor	Q9H7M9|GI24_HUMAN	1,16	1,05	1,30						
Pleckstrin homology domain-containing family O member 2	Q8TD55|PKHO2_HUMAN	1,36	**2,55**	**4,24**	0,97<br>**0,71**	1,00<br>**0,77**	**0,84**<br>**0,61**	1,44	**8,33**	**6,30**
Pleckstrin homology, Sec7 and coiled-coil domains 4 (Fragment)	B1AHI1|B1AHI1_HUMAN				0,00	0,00	0,00			
Plectin 10	Q6S377|Q6S377_HUMAN	**1,15**	**1,23**							
Plectin 6	Q6S380|Q6S380_HUMAN				**1,96**<br>2,09	**1,70**<br>1,89	**1,80**<br>1,90	**1,16**	**0,38**	**0,45**
Plectin-1	Q15149|PLEC1_HUMAN	0,89	**1,34**	**2,26**				0,29	0,34	0,30
Pleiotropic regulator 1	O43660|PLRG1_HUMAN							0,53	**0,28**	0,15
PLEK protein (Fragment)	Q6FGQ1|Q6FGQ1_HUMAN	**1,55**	**2,72**	**5,42**	**0,79**<br>**0,79**	0,92<br>1,07	**0,82**<br>1,01			
Plexin domain-containing protein 2 precursor	Q6UX71|PXDC2_HUMAN	1,13<br>1,04	0,90<br>0,70	1,19						
Plexin-B2 precursor	O15031|PLXB2_HUMAN	1,15<br>1,12	**0,78**<br>0,99	**0,88**						
PLS1 protein	Q8NEG6|Q8NEG6_HUMAN				0,72	1,27	1,33			
PML-RARA-regulated adapter molecule 1	Q96QH2|PRAM_HUMAN				0,96<br>0,71	**0,68**<br>0,78	**0,48**<br>0,69	1,12<br>1,19	0,91<br>1,48	**0,37**<br>0,85
POLDIP3 protein (Fragment)	Q96DI9|Q96DI9_HUMAN							**1,34**	0,90	0,56
POLR2C protein	Q6FGR6|Q6FGR6_HUMAN							1,14	0,54	0,36
Poly [ADP-ribose] polymerase 1	P09874|PARP1_HUMAN							1,01<br>0,45	0,92<br>0,76	1,01<br>0,92
Poly(A) RNA polymerase, mitochondrial precursor	Q9NVV4|PAPD1_HUMAN	1,03	0,96							
Poly(ADP-ribose) glycohydrolase ARH3	Q9NX46|ARHL2_HUMAN	0,90	0,89							
Poly(RC) binding protein 2	Q6IPF4|Q6IPF4_HUMAN	1,68	**6,85**	**12,75**	1,01	0,80	**0,52**	0,00	0,00	0,00
Poly(RC)-binding protein 2 isoform b variant (Fragment)	Q59HD4|Q59HD4_HUMAN				0,84	0,63	0,67	1,06	0,70	0,53
Poly(U)-binding-splicing factor PUF60	Q9UHX1|PUF60_HUMAN							1,09<br>1,12	**0,44**<br>0,82	**0,27**<br>0,73
Polyadenylate-binding protein 1	P11940|PABP1_HUMAN	1,10	**3,08**	**6,35**	**1,48**<br>1,52	**1,13**<br>1,05	0,93<br>1,09	0,99<br>0,91	1,22<br>0,84	0,86<br>0,74
Polyadenylate-binding protein 2	Q86U42|PABP2_HUMAN							0,95<br>**1,35**	**0,14**<br>**0,28**	**0,11**<br>**0,30**
Polyglutamine-binding protein 1	O60828|PQBP1_HUMAN							0,91<br>0,00	**0,28**<br>0,00	**0,21**<br>0,00
Polymerase acidic protein	Q3HM39|PA_I18A0				1,37	2,00	1,16			
Polymerase acidic protein	O89752|PA_I97A1	1,33	1,97							
Polymerase acidic protein	Q1K9E2|PA_I72A2	**2,43**	**5,24**	**8,34**	**1,83**	**3,26**	**3,42**			
Polymerase basic protein 2	Q8QPG7|PB2_I00A0	1,61	2,34	2,38						
Polymerase basic protein 2	Q67296|PB2_I72A2							5,70	0,93	1,31
Polymerase delta-interacting protein 2	Q9Y2S7|PDIP2_HUMAN	1,01	**0,85**							
Polymerase delta-interacting protein 3	Q9BY77|PDIP3_HUMAN							1,21	**0,36**	**0,16**
Polypeptide N-acetylgalactosaminyltransferase 1	Q10472|GALT1_HUMAN	0,00	0,00	0,00						
Polypeptide N-acetylgalactosaminyltransferase 12	Q8IXK2|GLT12_HUMAN	1,16<br>0,98	0,72<br>0,95	0,90						
Polypeptide N-acetylgalactosaminyltransferase 2	Q10471|GALT2_HUMAN	**1,33**<br>1,03	**0,62**<br>1,02	**0,77**						
Polypyrimidine tract binding protein 1	Q9BUQ0|Q9BUQ0_HUMAN	0,81	1,44	**3,33**				**0,86**	**0,31**	**0,20**

					**1,19**	**1,63**	1,23	**1,36**	**0,35**	**0,39**
Polyribonucleotide nucleotidyltransferase 1, mitochondrial precursor	Q8TCS8|PNPT1_HUMAN	1,29<br>1,05	**1,30**<br>1,01	1,10				0,86<br>**0,38**	0,75<br>1,35	0,90<br>**1,27**
Porphobilinogen deaminase	P08397|HEM3_HUMAN				1,19	0,99	0,86			
Possible J 56 gene segment (Fragment)	A0N4V7|A0N4V7_HUMAN				1,15	0,65	1,72			
PPM1G protein	Q6IAU5|Q6IAU5_HUMAN				0,49	0,80	0,64			
PPM2C protein (Fragment)	Q6P1N1|Q6P1N1_HUMAN	1,14	0,96							
PPT1 protein (Fragment)	Q6FGQ4|Q6FGQ4_HUMAN	1,10<br>**0,89**	1,11<br>**0,76**	**0,85**	**1,60**<br>**1,56**	**0,79**<br>0,80	**0,66**<br>**0,59**			
PQ-loop repeat-containing protein 1	Q8N2U9|PQLC1_HUMAN			1,08						
PRA1 family protein 3	O75915|PRAF3_HUMAN	**0,69**<br>1,04	1,09<br>**1,46**	1,37	**2,07**	1,23	**1,41**	1,00<br>1,22	**8,12**<br>**4,62**	**7,02**<br>**6,20**
Prefoldin subunit 1	O60925|PFD1_HUMAN				0,85	0,72	0,85			
Prefoldin subunit 2	Q9UHV9|PFD2_HUMAN				0,90	0,87	0,81			
Prefoldin subunit 5	Q99471|PFD5_HUMAN				0,93<br>0,60	0,80<br>0,66	0,83<br>0,69			
Prefoldin subunit 6	A2AB88|A2AB88_HUMAN				1,02<br>0,00	0,94<br>0,00	1,11<br>0,00			
Pre-mRNA 3'-end-processing factor FIP1	Q6UN15|FIP1_HUMAN							0,87	**0,39**	0,25
Pre-mRNA cleavage complex 2 protein Pcf11	O94913|PCF11_HUMAN							0,77<br>0,00	0,28<br>0,00	0,13<br>0,00
Pre-mRNA-processing factor 19	Q9UMS4|PRP19_HUMAN							**0,22**	**0,30**	**0,29**
Pre-mRNA-processing factor 40 homolog A	O75400|PR40A_HUMAN							**0,83**<br>**0,75**	**0,62**<br>**0,60**	**0,41**<br>**0,54**
Pre-mRNA-processing factor 6	O94906|PRP6_HUMAN							0,97<br>**0,33**	0,48<br>**0,35**	0,48<br>**0,25**
Pre-mRNA-processing-splicing factor 8	Q6P2Q9|PRP8_HUMAN							**1,46**<br>**0,20**	0,92<br>**0,46**	1,04<br>**0,35**
Pre-mRNA-splicing regulator WTAP	Q15007|FL2D_HUMAN							0,99	**0,33**	**0,25**
Prenylcysteine oxidase 1 precursor	Q9UHG3|PCYOX_HUMAN	1,06	1,34							
Prenylated Rab acceptor protein 1	Q9UI14|PRAF1_HUMAN	1,27	0,65	0,70						
Presenilin 2	B1AP22|B1AP22_HUMAN	1,27	1,09							
Presenilins-associated rhomboid-like protein, mitochondrial precursor	Q9H300|PARL_HUMAN	0,83	**0,78**							
Presequence protease, mitochondrial precursor	Q5JRX3|PREP_HUMAN	0,92<br>0,99	**0,85**<br>0,96	**0,66**						
Probable 10-formyltetrahydrofolate dehydrogenase ALDH1L2	Q3SY69|AL1L2_HUMAN	0,93	**0,81**							
Probable alanyl-tRNA synthetase, mitochondrial precursor	Q5JTZ9|SYAM_HUMAN	0,99<br>1,01	1,12<br>0,91	1,19						
Probable arginyl-tRNA synthetase, mitochondrial precursor	Q5T160|SYRM_HUMAN	0,97	0,95							
Probable asparaginyl-tRNA synthetase, mitochondrial precursor	Q96I59|SYNM_HUMAN	0,95	0,99							
Probable ATP-dependent RNA helicase DDX17	Q92841|DDX17_HUMAN				1,21	1,19	1,15			
Probable ATP-dependent RNA helicase DDX23	Q9BUQ8|DDX23_HUMAN							0,87<br>**0,52**	0,76<br>**0,55**	0,86<br>**0,41**
Probable ATP-dependent RNA helicase DDX28	Q9NUL7|DDX28_HUMAN	0,00<br>**0,83**	0,00<br>0,84	0,00						
Probable ATP-dependent RNA helicase DDX46	Q7L014|DDX46_HUMAN							**0,61**	**0,50**	**0,40**
Probable ATP-dependent RNA helicase DDX5	P17844|DDX5_HUMAN	0,97<br>1,07	1,27<br>**1,37**	**1,75**	1,01<br>0,97	1,09<br>1,44	**0,66**<br>0,82	**1,32**<br>**0,65**	**0,35**<br>**0,48**	**0,29**<br>**0,36**
Probable cation-transporting ATPase 13A1	Q9HD20|AT131_HUMAN	0,94<br>0,99	1,09<br>1,07	1,00						
Probable cysteinyl-tRNA synthetase, mitochondrial precursor	Q9HA77|SYCM_HUMAN	0,91<br>0,99	0,84<br>**0,91**	0,83						
Probable D-lactate dehydrogenase, mitochondrial precursor	Q86WU2|LDHD_HUMAN	0,86<br>**0,86**	**0,75**<br>**0,86**	**0,70**						
Probable E3 ubiquitin-protein ligase HERC2	O95714|HERC2_HUMAN	0,92	0,94							
Probable ergosterol biosynthetic protein 28	Q9UKR5|ERG28_HUMAN	**0,85**	**0,65**							
Probable histidyl-tRNA synthetase, mitochondrial precursor	P49590|SYHM_HUMAN	0,94<br>**1,11**	0,97<br>0,98	0,76						
Probable hydrolase PNKD	Q8N490|PNKD_HUMAN	0,90<br>1,04	0,91<br>1,05	0,80						
Probable leucyl-tRNA synthetase, mitochondrial precursor	Q15031|SYLM_HUMAN	0,93	0,97							
Probable peptidyl-tRNA hydrolase	Q86Y79|PTH_HUMAN	1,06	0,91							
Probable protein BRICK1	Q8WUW1|BRK1_HUMAN							0,79<br>0,79	**1,77**<br>**1,60**	**1,45**<br>**1,81**
Probable RNA-binding protein 25	P49756|RBM25_HUMAN							0,92	**0,56**	**0,38**
Probable rRNA-processing protein EBP2	Q99848|EBP2_HUMAN							**0,72**<br>0,94	**0,76**<br>0,95	**0,62**<br>1,21
Probable saccharopine dehydrogenase	Q8NBX0|SCPDH_HUMAN	0,88<br>0,96	0,91<br>1,04	0,97						
Probable serine carboxypeptidase CPVL precursor	Q9H3G5|CPVL_HUMAN	**1,33**<br>0,90	**1,60**<br>0,95	**1,32**	0,00<br>0,00	0,00<br>0,00	0,00<br>0,00	1,09	1,08	1,00
Procollagen-lysine,2-oxoglutarate 5-dioxygenase 1 precursor	Q02809|PLOD1_HUMAN	1,53<br>0,85	0,93<br>1,02	1,26						
Procollagen-lysine,2-oxoglutarate 5-dioxygenase 3 precursor	O60568|PLOD3_HUMAN	1,08<br>0,98	1,11<br>1,05	1,14				1,08	**1,75**	**4,89**
Procollagen-proline, 2-oxoglutarate 4-dioxygenase (Proline 4-hydroxylase), alpha polypeptide I	Q5VSQ6|Q5VSQ6_HUMAN	0,81<br>1,03	0,88<br>1,08	0,93						
Profilin 1	P07737|PROF1_HUMAN	0,00<br>1,01	0,00<br>1,05	0,00	0,96<br>1,06	1,11<br>**0,82**	1,16<br>1,39	0,00	0,00	0,00
Progerin	Q6UYC3|Q6UYC3_HUMAN	**0,58**	**0,31**	**0,40**				**0,95**	**0,38**	**0,18**
Programmed cell death 6	Q2YDC2|Q2YDC2_HUMAN				1,21	1,13	**1,79**	1,31	**1,31**	1,16
Programmed cell death 6-interacting protein	Q8WUM4|PDC6I_HUMAN				**0,87**	**0,82**	**0,74**			
Programmed cell death protein 10	Q9BUL8|PDC10_HUMAN				0,61	0,78	0,61			
Programmed cell death protein 4	Q53EL6|PDCD4_HUMAN				**0,66**	0,77	**0,70**			
Programmed cell death protein 5	O14737|PDCD5_HUMAN				0,59<br>0,55	0,79<br>**0,69**	0,68<br>**0,64**			
Programmed cell death protein 6	O75340|PDCD6_HUMAN							1,50	**1,64**	1,45
Prohibitin	P35232|PHB_HUMAN	**0,91**	**0,93**							

Prohibitin variant (Fragment)	Q53FV0|Q53FV0_HUMAN							0,92	**3,51**	**5,54**
					2,86	**1,56**	**1,58**	**1,50**	**2,81**	**5,52**
Prohibitin-2	Q99623|PHB2_HUMAN	1,00	0,95	**0,86**	1,45	1,02	1,04	0,81	**2,65**	**3,20**
		1,00	1,01		**2,36**	1,10	1,43	1,10	**2,09**	**3,88**
Prolactin regulatory element-binding protein	Q9HCU5|PREB_HUMAN									
		**0,76**	0,85							
Proliferating cell nuclear antigen	P12004|PCNA_HUMAN				0,91	1,03	0,82			
					0,91	0,91	**0,61**			
Proliferation-associated protein 2G4	Q9UQ80|PA2G4_HUMAN							0,96	**1,10**	**1,37**
Proline synthetase co-transcribed bacterial homolog protein	O94903|PROSC_HUMAN	0,00	0,00	0,00						
		0,87	0,84		0,79	1,03	0,85			
Proline-, glutamic acid- and leucine-rich protein 1	Q8IZL8|PELP1_HUMAN									
								0,21	0,39	0,28
Proline-serine-threonine phosphatase-interacting protein 1	O43586|PPIP1_HUMAN	0,76	0,82	1,07						
	Q07954|LRP1_HUMAN	**1,09**	**0,71**	**0,74**				0,96	0,68	0,86
Prolow-density lipoprotein receptor-related protein 1 precursor		0,98	**0,82**		0,84	1,04	0,75			
Prolyl 3-hydroxylase 1 precursor	Q32P28|P3H1_HUMAN									
		0,99	**1,27**							
Prolyl endopeptidase	Q9UM02|Q9UM02_HUMAN				0,65	0,86	0,52			
					1,47	1,17	1,22			
Propionyl Coenzyme A carboxylase, beta polypeptide variant	Q59GV0|Q59GV0_HUMAN									
(Fragment)		0,85	0,85							
Propionyl-CoA carboxylase alpha subunit	Q8WXQ7|Q8WXQ7_HUMAN	1,00	1,24	1,21						
		0,89	0,98							
	P05166|PCCB_HUMAN	0,73	0,59	**0,52**						
Propionyl-CoA carboxylase beta chain, mitochondrial precursor										
Prosaposin	B1AVU8|B1AVU8_HUMAN									
		0,98	0,99							
Prosaposin variant (Fragment)	Q59EN5|Q59EN5_HUMAN	0,96	0,88	**0,72**	**1,84**	0,90	**1,22**	1,03	**1,29**	1,32
					1,34	0,55	1,12			
Prostaglandin E synthase 2	Q9H7Z7|PGES2_HUMAN	0,92	**0,79**	**0,69**						
		1,07	1,04		0,00	0,00	0,00			
Prostaglandin E synthase 3	Q15185|TEBP_HUMAN				0,82	**0,82**	**0,60**			
					**0,70**	**0,67**	**0,59**			
Prostaglandin G/H synthase 1 precursor	P23219|PGH1_HUMAN	0,93	1,08	1,02						
		0,91	1,03							
Protease serine 1 (Fragment)	Q86W19|Q86W19_HUMAN	0,00	0,00	0,00				0,00	0,00	0,00
Protease serine 2 isoform B	Q7Z5F3|Q7Z5F3_HUMAN	**0,68**	1,03	1,10						
								1,19	1,37	**1,65**
Protease serine 2 preproprotein	A6XMV9|A6XMV9_HUMAN							**1,52**	**1,72**	1,48
Protease, serine, 3	Q5VXV0|Q5VXV0_HUMAN				**1,52**	**1,50**	**1,68**			
Proteasome (Prosome, macropain) 26S subunit, ATPase, 1	Q6NW36|Q6NW36_HUMAN				0,92	0,89	**0,79**			
					0,89	0,96	**0,74**			
Proteasome (Prosome, macropain) 26S subunit, non-ATPase,	Q5VWC4|Q5VWC4_HUMAN									
4 (Fragment)					0,93	0,88	**0,51**			
Proteasome 26S ATPase subunit 5 variant (Fragment)	Q59GS3|Q59GS3_HUMAN									
					1,02	0,96	1,19			
Proteasome 26S non-ATPase subunit 11 variant (Fragment)	Q53FT5|Q53FT5_HUMAN									
					**0,58**	0,96	**0,67**			
Proteasome 26S non-ATPase subunit 2 variant (Fragment)	Q59EG8|Q59EG8_HUMAN	1,05	**2,63**	**8,80**	0,85	0,90	0,75			
					0,90	1,42	**1,21**			
Proteasome activator complex subunit 2	Q9UL46|PSME2_HUMAN				0,91	1,06	1,09			
					**0,61**	1,05	1,19			
Proteasome beta 1 subunit variant (Fragment)	Q53FT8|Q53FT8_HUMAN				0,95	**1,15**	**1,35**			
					1,17	1,49	2,01			
Proteasome subunit alpha type	Q5U0A0|Q5U0A0_HUMAN				1,07	1,32	**1,84**			
					0,92	0,95	1,13			
Proteasome subunit alpha type	Q53XP2|Q53XP2_HUMAN				1,06	**1,30**	**1,60**			
					1,11	1,05	1,50			
Proteasome subunit alpha type	Q6IB71|Q6IB71_HUMAN				1,06	1,03	**1,39**			
Proteasome subunit alpha type (Fragment)	Q6ICS6|Q6ICS6_HUMAN									
					1,07	**1,33**	1,18			
Proteasome subunit alpha type (Fragment)	Q05DH1|Q05DH1_HUMAN				1,01	1,11	**1,47**			
					1,00	1,12	1,33			
Proteasome subunit alpha type-1	P25786|PSA1_HUMAN				0,97	1,01	**1,26**			
					0,93	1,08	**1,26**			
Proteasome subunit alpha type-6	P60900|PSA6_HUMAN				1,23	0,93	1,06			
					1,05	1,07	1,61			
Proteasome subunit beta type	Q5JNW6|Q5JNW6_HUMAN				1,06	**1,35**	**1,61**			
					0,98	1,24	1,11			
Proteasome subunit beta type	Q6IB22|Q6IB22_HUMAN				0,89	1,07	**1,32**			
					0,86	**1,50**	**1,73**			
Proteasome subunit beta type	Q6IAT9|Q6IAT9_HUMAN									
					0,00	0,00	0,00			
Proteasome subunit beta type-2	P49721|PSB2_HUMAN				1,18	**1,20**	**1,41**			
Proteasome subunit beta type-7 precursor	Q99436|PSB7_HUMAN									
					0,98	1,11	1,49			
Protein ADRM1	Q16186|ADRM1_HUMAN				1,01	1,28	0,89			
					0,86	0,78	0,70	0,56	1,26	1,19
Protein ARMET precursor	P55145|ARMET_HUMAN	1,03	1,12	0,99				0,91	**1,54**	**1,84**
		1,02	1,13					1,04	**2,93**	**4,00**
Protein bassoon	Q9UPA5|BSN_HUMAN									
		0,72	0,96							
Protein beta-actin-like	Q562R1|ACTBL_HUMAN									
					0,00	0,00	0,00			
Protein BUD31 homolog	P41223|BUD31_HUMAN							0,00	0,00	0,00
Protein C14orf166	Q9Y224|CN166_HUMAN							1,07	**0,69**	**0,36**
					0,81	0,80	0,82	1,27	**1,31**	**0,74**
Protein canopy homolog 2 precursor	Q9Y2B0|CNPY2_HUMAN	0,93	0,95	0,93				1,30	**8,71**	**11,09**
		1,01	1,18		0,00	0,00	0,00			
Protein canopy homolog 3 precursor	Q9BT09|CNPY3_HUMAN	0,97	1,11	1,28	2,24	1,12	2,44	0,85	5,20	6,76
		0,94	0,93		**1,84**	1,23	1,72			
Protein CASP	Q13948|CASP_HUMAN									
		1,09	1,09							
Protein cornichon homolog 4	Q9P003|CNIH4_HUMAN									
		0,00	0,00							
Protein CWC15 homolog	Q9P013|CWC15_HUMAN							1,02	0,45	0,30
								1,36	**0,43**	0,48
Protein DEK	P35659|DEK_HUMAN							0,87	**0,35**	**0,40**
					1,04	1,60	1,35	**1,26**	**0,83**	0,92
Protein disulfide-isomerase A3 precursor	P30101|PDIA3_HUMAN	**0,94**	0,97	0,95	**1,66**	1,06	**1,99**	0,97	**4,23**	**8,48**
		**0,92**	**1,12**		1,75	**1,15**	1,51	**1,21**	**4,07**	**7,60**
Protein disulfide-isomerase A5 precursor	Q14554|PDIA5_HUMAN	0,91	1,16	0,76						
		0,96	1,00							
Protein disulfide-isomerase precursor	P07237|PDIA1_HUMAN	**0,92**	1,00	**0,90**	**1,64**	**1,13**	**2,14**	0,85	**1,78**	**3,25**
		0,97	**1,18**		1,77	**1,22**	1,71	**0,70**	**1,68**	**3,09**
Protein disulfide-isomerase TXNDC10 precursor	Q96JJ7|TXD10_HUMAN	0,87	1,22	1,18						
		1,02	1,08							
Protein DJ-1	Q99497|PARK7_HUMAN	0,99	1,02	**0,74**	**0,85**	**0,92**	**0,92**			
		1,08	0,92		**0,74**	0,90	0,92			
Protein dpy-19 homolog 2	Q6NUT2|D19L2_HUMAN				0,00	0,00	0,00			

Protein dpy-30 homolog	Q9C005|DPY30_HUMAN							0,96	**0,38**	**0,28**
								1,24	0,95	0,60
Protein EMI5 homolog, mitochondrial precursor	Q9NX18|EMI5_HUMAN									
		0,95	1,01							
Protein FAM105A	Q9NUU6|F105A_HUMAN									
		**0,89**	**1,13**							
Protein FAM134C	Q86VR2|F134C_HUMAN	0,00	0,00	0,00						
					2,26	0,91	1,08			
Protein FAM136A	Q96C01|F136A_HUMAN									
		0,98	**0,89**							
Protein FAM14A precursor	Q9H2X8|FA14A_HUMAN									
		0,92	0,93							
Protein FAM3C precursor	Q92520|FAM3C_HUMAN	1,02	**0,65**	**0,77**				1,33	**2,23**	**4,08**
		1,02	1,02							
Protein FAM49B	Q9NUQ9|FA49B_HUMAN	**1,39**	1,09	1,16						
		**1,36**	1,00		**0,83**	**0,86**	**0,69**			
Protein FAM82A	Q96LZ7|FA82A_HUMAN	1,08	1,06	1,10						
Protein FAM82B	Q96DB5|FA82B_HUMAN	0,73	**0,44**	0,42						
		0,95	**0,77**							
Protein FAM82C	Q96TC7|FA82C_HUMAN	0,84	0,87	1,50						
		1,02	0,93							
Protein FAM91A1	Q658Y4|F91A1_HUMAN									
					0,00	0,00	0,00			
Protein GPR108 precursor	Q9NPR9|GP108_HUMAN	1,28	0,86	0,98						
Protein jagunal homolog 1	Q8N5M9|JAGN1_HUMAN	0,00	0,00	0,00				0,00	0,00	0,00
		0,00	0,00					0,00	0,00	0,00
Protein KIAA1524	Q8TCG1|K1524_HUMAN									
					0,66	0,65	0,73			
Protein kinase C and casein kinase substrate in neurons	Q9UNF0|PACN2_HUMAN				**0,64**	**0,80**	**0,77**	0,92	1,61	1,60
protein 2					**0,72**	**0,86**	0,87	0,95	**1,83**	1,70
Protein kinase C beta type	P05771|KPCB_HUMAN	0,00	0,00	0,00						
Protein kinase C, delta	Q86XJ6|Q86XJ6_HUMAN	0,95	**1,61**	**3,18**						
		**1,45**	**1,46**		0,83	0,81	0,60			
Protein LAP4	Q14160|LAP4_HUMAN									
					1,03	0,87	0,89			
Protein lunapark	Q9C0E8|LNP_HUMAN									
		1,29	1,70							
Protein LYRIC	Q86UE4|LYRIC_HUMAN	0,82	0,91	1,24	1,77	0,89	1,10	0,94	**1,64**	**1,58**
		1,12	0,98		**2,04**	1,15	1,18	1,15	**2,60**	**3,15**
Protein LZIC	Q8WZA0|LZIC_HUMAN				0,68	0,69	**0,77**			
					**0,63**	0,80	0,60			
Protein mago nashi homolog 2	Q96A72|MGN2_HUMAN							**0,75**	**0,34**	**0,15**
Protein MANBAL	Q9NQG1|MANBL_HUMAN									
		0,00	0,00							
Protein Niban	Q9BZQ8|NIBA_HUMAN									
					**0,80**	0,91	0,85			
Protein NipSnap homolog 1	Q9BPW8|NIPS1_HUMAN	1,00	1,14	1,06						
		0,98	0,95							
Protein NipSnap homolog 3A	Q9UFN0|NPS3A_HUMAN	0,00	0,00	0,00						
		0,00	0,00							
Protein ODR4 homolog	Q5SWX8|ODR4_HUMAN									
		1,06	1,19							
Protein phosphatase 1A	P35813|PPM1A_HUMAN									
					0,00	0,00	0,00			
Protein phosphatase 1 regulatory subunit 7	Q15435|PP1R7_HUMAN				0,66	0,91	0,95			
Protein phosphatase inhibitor 2	P41236|IPP2_HUMAN									
					0,55	0,95	0,83			
Protein PPRC1	Q96M27|PRRC1_HUMAN				0,87	0,93	0,76			
					0,86	1,34	0,75			
Protein QIL1	Q5XKP0|QIL1_HUMAN	0,91	0,94	0,83						
		1,07	1,20							
Protein quaking	Q96PU8|QKI_HUMAN							**1,32**	0,33	0,32
Protein RCC2	Q9P258|RCC2_HUMAN	0,00	0,00	0,00	0,89	1,67	0,87			
					**1,31**	1,49	1,04	0,20	0,43	0,31
Protein RFT1 homolog	Q96AA3|RFT1_HUMAN	0,87	0,97	1,32						
		0,92	1,00							
Protein S100-A10	P60903|S10AA_HUMAN	**1,32**	**1,52**	**2,55**	**1,58**	**1,27**	**1,83**	**1,20**	**1,25**	**1,27**
		**2,03**	**1,34**		1,45	1,16	1,76	**1,52**	**1,95**	**2,06**
Protein S100-A11	P31949|S10AB_HUMAN	**2,09**	**2,10**	**3,33**	**1,33**	**1,05**	**1,40**	**1,89**	**2,09**	**1,44**
		**3,15**	**2,62**		0,97	0,91	**1,15**	**0,72**	1,05	0,93
Protein S100-A13	Q99584|S10AD_HUMAN				0,94	0,94	1,10	1,08	**1,74**	**1,78**
					**0,71**	0,91	0,95	1,15	2,60	2,69
Protein S100-A4	P26447|S10A4_HUMAN	**1,95**	**5,25**	**13,15**	**0,90**	0,95	**1,37**	1,19	**2,53**	1,78
		**1,53**	**2,30**		0,71	0,90	1,14	**1,16**	**3,18**	**2,30**
Protein S100-A6	P06703|S10A6_HUMAN	1,23	**1,84**	**3,00**	0,96	**1,07**	**1,26**	**1,59**	**3,75**	**2,86**
		1,20	**1,44**		0,68	**0,78**	0,88	**0,77**	**2,56**	**2,35**
Protein S100-A8	P05109|S10A8_HUMAN	1,31	**5,39**	**9,35**	**0,73**	0,96	**0,88**	1,21	**13,25**	**12,58**
		**1,45**	**2,30**		0,58	0,87	**0,75**	1,13	**12,37**	**10,67**
Protein S100-A9	P06702|S10A9_HUMAN	0,00	0,00	0,00	**0,84**	1,04	**0,90**	1,31	**10,61**	**10,20**
		**2,79**	**5,38**		**0,63**	**0,80**	**0,66**	1,41	**9,89**	**8,72**
Protein SDA1 homolog	Q9NVU7|SDA1_HUMAN									
		0,00	0,00					1,07	1,32	1,37
Protein sel-1 homolog 1 precursor	Q9UBV2|SE1L1_HUMAN	0,80	1,03	0,97						
		1,03	1,10							
Protein Shroom3	Q8TF72|SHRM3_HUMAN				1,23	1,45	1,50			
Protein spinster homolog 1	Q9H2V7|SPNS1_HUMAN	1,10	1,07	0,83						
		1,05	0,96							
Protein TBRG4	Q969Z0|TBRG4_HUMAN	0,87	0,93	0,82						
		1,11	1,03							
Protein translocation complex beta variant (Fragment)	Q53FA5|Q53FA5_HUMAN									
								0,99	**2,94**	**3,84**
Protein transport protein Sec24C	P53992|SC24C_HUMAN									
					0,82	1,33	1,09			
Protein transport protein Sec31A	O94979|SC31A_HUMAN				0,97	1,08	0,85			
					1,01	1,02	1,02			
Protein transport protein Sec61 subunit alpha isoform 1	P61619|S61A1_HUMAN	0,98	1,04	0,97				1,54	**2,25**	**4,66**
		1,01	0,97					**0,31**	1,48	**2,06**
Protein transport protein Sec61 subunit beta	P60468|SC61B_HUMAN	1,07	1,17	1,24				**0,60**	**1,90**	**2,22**
Protein tweety homolog 3	Q9C0H2|TTYH3_HUMAN	0,72	0,53	0,50						
		0,92	0,90							
Protein tyrosine phosphatase, non-receptor type 6	Q53XS4|Q53XS4_HUMAN							1,09	1,01	**1,28**
Protein tyrosine phosphatase-like protein PTPLAD1	Q9P035|PTAD1_HUMAN	1,03	0,80	0,79						
		0,00	0,00							
Protein unc-84 homolog B	Q9UH99|UN84B_HUMAN							0,62	0,50	0,20
								0,58	0,66	0,45
Protein wibg homolog	Q9BRP8|WIBG_HUMAN				0,00	0,00	0,00	0,96	0,80	**0,44**
					0,70	0,78	0,45	1,18	1,39	0,76
Protein Wiz	O95785|WIZ_HUMAN									
								1,10	3,89	6,54

Protein XRP2	O75695|XRP2_HUMAN	**1,37**<br>**1,23**	1,12<br>**1,14**	1,28						
Protein YIPF3	Q9GZM5|YIPF3_HUMAN	0,81<br>0,88	0,64<br>0,69	0,71						
Protein YIPF4	Q9BSR8|YIPF4_HUMAN	1,17	**0,69**	**0,77**						
Protein YIPF5	Q969M3|YIPF5_HUMAN	0,91	1,19							
Protein YIPF6	Q96EC8|YIPF6_HUMAN	1,14	0,97							
Protein-L-isoaspartate(D-aspartate) O-methyltransferase	P22061|PIMT_HUMAN	0,84	0,93							
Protein-tyrosine phosphatase mitochondrial 1, mitochondrial precursor	Q8WUK0|PTPM1_HUMAN	0,86<br>1,05	**0,62**<br>0,87	0,61						
Protein-tyrosine phosphatase-like member B	Q6Y1H2|Q6Y1H2_HUMAN	1,05	1,13							
Protein-tyrosine sulfotransferase 2	O60704|TPST2_HUMAN	1,10<br>0,95	0,75<br>1,01	1,02						
Proteolipid protein 2	Q04941|PLP2_HUMAN	1,43<br>0,00	0,94<br>0,00	1,83	0,00<br>3,05	0,00<br>1,57	0,00<br>0,00	0,00	0,00	0,00
Proteolysis inducing factor	Q53YJ2|Q53YJ2_HUMAN				1,22	2,56	2,26			
Prothymosin alpha	Q15202|Q15202_HUMAN				**0,45**	**0,41**	0,31			
Prothymosin alpha precursor	Q15254|Q15254_HUMAN	0,00	0,00	0,00						
Proto-oncogene tyrosine-protein kinase FGR	P09769|FGR_HUMAN	1,07<br>**1,35**	1,05<br>**1,37**	**1,46**				0,60	**0,72**	0,76
Protoporphyrinogen oxidase	Q5VTW8|Q5VTW8_HUMAN	1,05	0,85							
PRP3 pre-mRNA processing factor 3 homolog	Q5VT54|Q5VT54_HUMAN							0,99	**0,40**	**0,24**
PRP4 pre-mRNA processing factor 4 homolog B	Q8IVC3|Q8IVC3_HUMAN							**0,76**	**0,70**	**0,61**
PRSS3 protein	A1A508|A1A508_HUMAN	0,49	**0,80**	1,02				**1,60**<br>**1,30**	**1,67**<br>**1,70**	**1,59**<br>**2,20**
P-selectin glycoprotein ligand 1 precursor	Q14242|SELPL_HUMAN	0,00	0,00							
PSMB4 protein	Q6IBI4|Q6IBI4_HUMAN				0,00<br>1,65	0,00<br>2,30	0,00<br>3,23			
PSMD3 protein	Q6IBN0|Q6IBN0_HUMAN	1,25<br>1,03	1,60<br>1,05	3,05						
PSME1 protein	Q6IBM2|Q6IBM2_HUMAN				**0,69**	1,03	1,00			
Pterin-4-alpha-carbinolamine dehydratase 2	Q9H0N5|PHS2_HUMAN	1,14	1,04	0,99						
Puromycin-sensitive aminopeptidase	P55786|PSA_HUMAN				0,87<br>0,97	0,92<br>1,01	0,92<br>0,93			
Putative adenosylhomocysteinase 2	O43865|SAHH2_HUMAN	1,36	1,86	4,05	0,94	1,03	0,98			
Putative ATP-dependent Clp protease proteolytic subunit, mitochondrial precursor	Q16740|CLPP_HUMAN	1,13<br>0,95	1,12<br>1,02	0,76						
Putative ATP-dependent RNA helicase DHX29	Q7Z478|DHX29_HUMAN				0,00	0,00	0,00			
Putative ATP-dependent RNA helicase DHX30	Q7L2E3|DHX30_HUMAN	1,02	1,01							
Putative eukaryotic translation initiation factor 1A	O75642|IF1AH_HUMAN				0,95	**0,79**	0,79			
Putative GTP-binding protein 6	O43824|GTPB6_HUMAN	0,72<br>0,95	0,50<br>0,88	0,60						
Putative metalloprotease C21orf57	P58557|CU057_HUMAN	**1,15**	0,99							
Putative methyltransferase NSUN4	Q96CB9|NSUN4_HUMAN	0,95	1,12							
Putative phospholipase B-like 1 precursor	Q6P4A8|PLBL1_HUMAN	**1,19**	**1,56**	**1,52**	1,74	0,98	**2,22**	0,99<br>**0,84**	**1,89**<br>**1,90**	**2,16**<br>**2,23**
Putative phospholipase B-like 2 precursor	Q8NHP8|PLBL2_HUMAN	1,05<br>0,92	**1,41**<br>**0,83**	1,18	**1,76**<br>**1,54**	0,92<br>1,07	**1,53**<br>**1,50**			
Putative pre-mRNA-splicing factor ATP-dependent RNA helicase DHX15	O43143|DHX15_HUMAN				**1,40**	**1,49**	1,41	**0,23**	**0,38**	**0,29**
Putative RNA-binding protein 15	Q96T37|RBM15_HUMAN							1,29	1,01	1,10
Putative RNA-binding protein 3	P98179|RBM3_HUMAN							0,97<br>**1,32**	**0,40**<br>**0,53**	**0,16**<br>**0,33**
Putative RNA-binding protein Luc7-like 2	Q9Y383|LC7L2_HUMAN							**1,18**<br>1,10	0,70<br>0,93	**0,53**<br>**0,87**
Putative S-adenosyl-L-methionine-dependent methyltransferase METT5D1	A6NJ78|ME5D1_HUMAN	0,00	0,00							
Putative sodium-coupled neutral amino acid transporter 10	Q9HBR0|S38AA_HUMAN	0,97	0,96							
Putative transferase C1orf69, mitochondrial precursor	Q5T440|CA069_HUMAN	1,00	0,98							
Putative ubiquitin-conjugating enzyme E2 N-like	Q5JXB2|UE2NL_HUMAN				0,78	0,79	0,76			
Putative uncharacterized protein	Q86Z22|Q86Z22_HUMAN	1,53<br>1,12	**1,53**<br>**1,35**	**1,67**	**0,62**<br>0,56	**0,78**<br>0,70	**0,63**<br>0,58	**0,83**<br>1,33	**1,25**<br>1,47	1,03<br>1,13
Putative uncharacterized protein	Q9Y520|Q9Y520_HUMAN	1,07	1,39		0,93	0,87	0,70	0,91	**0,77**	**0,57**
Putative uncharacterized protein	Q504R3|Q504R3_HUMAN							**0,71**<br>1,23	0,39<br>1,11	0,14<br>0,87
Putative uncharacterized protein (Fragment)	Q9BU08|Q9BU08_HUMAN	1,13	1,59	3,97						
Putative uncharacterized protein (Fragment)	Q8TBW1|Q8TBW1_HUMAN				1,09	0,84	**0,77**			
Putative uncharacterized protein (Fragment)	Q96C20|Q96C20_HUMAN				**0,73**	0,54	**0,46**	1,65	**8,33**	**5,15**
Putative uncharacterized protein (Fragment)	Q59ED7|Q59ED7_HUMAN	**1,18**	**1,32**		1,92	1,07	1,08			
Putative uncharacterized protein (Fragment)	Q8TBT6|Q8TBT6_HUMAN	0,97	0,97							
Putative uncharacterized protein (Fragment)	Q9UEL4|Q9UEL4_HUMAN	1,16	1,82							
Putative uncharacterized protein DKFZp451D234	Q86TB8|Q86TB8_HUMAN				1,16<br>**0,86**	1,19<br>1,12	1,34<br>0,95			
Putative uncharacterized protein DKFZp547A0616 (Fragment)	Q5JQ44|Q5JQ44_HUMAN	1,14	0,91	0,97						
Putative uncharacterized protein DKFZp547L106	Q9H023|Q9H023_HUMAN				0,81	0,58	0,37			
Putative uncharacterized protein DKFZp564E242	Q659F9|Q659F9_HUMAN				0,72<br>0,97	0,77<br>0,78	0,79<br>0,83			
Putative uncharacterized protein DKFZp564G0422	Q5JXL8|Q5JXL8_HUMAN							1,18	**1,88**	**3,01**
Putative uncharacterized protein DKFZp564H1664	Q659E8|Q659E8_HUMAN				**2,01**	1,33	1,53			
Putative uncharacterized protein DKFZp566H1924 (Fragment)	Q9UFM8|Q9UFM8_HUMAN	1,07	1,12							
Putative uncharacterized protein DKFZp586G1517 (Fragment)	Q9UFU8|Q9UFU8_HUMAN	1,01	0,99							
Putative uncharacterized protein DKFZp666O168	Q658R4|Q658R4_HUMAN				0,76	**0,84**	0,56			

Guanine nucleotide-binding protein G(I)/G(S)/G(O) subunit gamma-2	P59768|GBG2_HUMAN	0,00	0,00							
Putative uncharacterized protein DKFZp686B04128	Q68D08|Q68D08_HUMAN				0,81	0,99	0,92			
Putative uncharacterized protein DKFZp686D0880 (Fragment)	Q6N0B1|Q6N0B1_HUMAN				1,02<br>1,78	1,06<br>**1,57**	**1,41**<br>1,98			
Putative uncharacterized protein DKFZp686E12175	Q68DQ7|Q68DQ7_HUMAN				0,79<br>**0,74**	0,58<br>0,84	0,85<br>0,84			
Putative uncharacterized protein DKFZp686E2459	Q7Z3D7|Q7Z3D7_HUMAN							0,84<br>1,06	0,54<br>0,79	0,41<br>0,75
Putative uncharacterized protein DKFZp686G04235	Q63HL4|Q63HL4_HUMAN	0,97	**1,27**	**1,65**						
Mitochondrial 28S ribosomal protein S29	P51398|RT29_HUMAN	0,99<br>0,90	0,99<br>1,00	0,89						
IgG receptor FcRn large subunit p51 precursor	P55899|FCGRN_HUMAN	0,84<br>0,94	0,66<br>**0,79**	0,86						
Putative uncharacterized protein DKFZp686I0746	Q6MZZ3|Q6MZZ3_HUMAN	1,07	0,86		1,07	1,10	1,04			
Putative uncharacterized protein DKFZp686J1372	Q5HYB6|Q5HYB6_HUMAN	**1,71**	**2,04**					**2,09**	**2,82**	**3,37**
Putative uncharacterized protein DKFZp686K08109	Q7Z3S1|Q7Z3S1_HUMAN	0,96	0,98							
Putative uncharacterized protein DKFZp686L0869	Q7Z3U5|Q7Z3U5_HUMAN	1,52	1,28	**3,60**	0,89	1,08	0,91			
Putative uncharacterized protein DKFZp686M09245	Q6MZV5|Q6MZV5_HUMAN				**0,62**<br>0,44	1,04<br>0,69	1,02<br>0,58	1,57<br>0,94	2,99<br>2,04	4,62<br>3,30
Isocitrate dehydrogenase [NADP] cytoplasmic	O75874|IDHC_HUMAN				**0,84**	0,98	**0,83**			
Putative uncharacterized protein DKFZp686M1669	Q68E10|Q68E10_HUMAN	1,03	1,57	3,33	**1,35**	0,83	0,76	1,25<br>0,00	**1,78**<br>0,00	**4,51**<br>0,00
Putative uncharacterized protein DKFZp686M22160 (Fragment)	Q7Z349|Q7Z349_HUMAN	0,79<br>1,08	1,00<br>0,99	1,31						
Putative uncharacterized protein DKFZp686N1815 (CASP3)	Q5HYI3|Q5HYI3_HUMAN				0,00	0,00	0,00			
Putative uncharacterized protein DKFZp779B2258	Q68D48|Q68D48_HUMAN				0,67	**0,81**	0,78			
Putative uncharacterized protein DKFZp779I1858	Q68D50|Q68D50_HUMAN	0,91	0,78	**0,68**						
Putative uncharacterized protein DKFZp779L0468	Q68DQ4|Q68DQ4_HUMAN	0,93	0,83	1,63	0,91	0,96	0,96			
Putative uncharacterized protein DKFZp779P1227	Q68DW4|Q68DW4_HUMAN							1,47		1,63
Putative uncharacterized protein DKFZp781B1032	Q68DD1|Q68DD1_HUMAN	1,06	0,93	1,00				0,72<br>0,95	1,85<br>1,91	**4,09**<br>2,49
Putative uncharacterized protein DKFZp781C0423 (Fragment)	Q68DD9|Q68DD9_HUMAN							0,98<br>**0,73**	0,59<br>**0,44**	0,51<br>**0,41**
Beta-hexoaminidase alpha chain precursor	P06865|HEXA_HUMAN	1,03	**0,78**		**1,70**	1,01	1,11			
Putative uncharacterized protein DKFZp781K0743	P42765|THIM_HUMAN							1,16	0,92	**2,10**
Putative uncharacterized protein DKFZp781N1372	Q68CQ5|Q68CQ5_HUMAN				0,89	0,90	0,95			
Protein disulfide-isomerase A4 precursor	P13667|PDIA4_HUMAN	0,94<br>**0,94**	1,04<br>**1,12**	**0,91**	**1,59**<br>2,02	**1,15**<br>1,43	**2,00**<br>1,91	0,76<br>0,95	1,10<br>**1,83**	1,69<br>**2,69**
Glycerol-3-phosphate dehydrogenase, mitochondrial precursor	P43304|GPDM_HUMAN							0,77<br>0,85	**2,95**<br>1,25	**6,40**<br>**2,13**
General transcription factor II-I	P78347|GTF21_HUMAN							0,94<br>**0,75**	**0,25**<br>**0,46**	**0,20**<br>**0,37**
Putative uncharacterized protein HDLBP	Q53QU2|Q53QU2_HUMAN				1,38	0,90	0,79			
Putative uncharacterized protein ITGA6	Q53RX7|Q53RX7_HUMAN	1,00	**0,54**	**0,63**						
Putative uncharacterized protein LOC552891	Q96BN9|Q96BN9_HUMAN	0,00	0,00	0,00						
U6 snRNA-associated Sm-like protein LSm6	P62312|LSM6_HUMAN							0,84<br>1,15	**0,44**<br>**0,52**	**0,29**<br>**0,49**
Mitochondrial 28S ribosomal protein S5	P82675|RT05_HUMAN	0,90<br>1,07	1,06<br>**0,91**	0,95						
Putative uncharacterized protein Nbla03646	Q3LIE7|Q3LIE7_HUMAN							0,26	2,08	**3,26**
26S protease regulatory subunit 7	P35998|PRS7_HUMAN				0,88	0,92	0,65			
Protein disulfide-isomerase A6 precursor	Q15084|PDTA6_HUMAN	0,99<br>**0,88**	0,95<br>1,05	0,96	**1,63**<br>1,84	0,98<br>1,16	**1,77**<br>**1,69**	1,21<br>**1,29**	**2,73**<br>**3,01**	**8,23**<br>**8,79**
Poly(rc)-binding protein 1	Q15365|PCBP1_HUMAN	0,82	2,81	4,97	**0,73**<br>0,88	**0,80**<br>0,58	**0,57**<br>**0,60**	0,90<br>0,67	**0,49**<br>0,63	**0,45**<br>**0,42**
Putative uncharacterized protein PSMC2 (Fragment)	Q75L23|Q75L23_HUMAN	2,15	**5,71**	**16,54**						
Putative uncharacterized protein PTPN18	Q53P42|Q53P42_HUMAN				0,00	0,00	0,00			
Ras-related protein Rab-10	P61026|RAB10_HUMAN							**0,31**	**1,73**	**2,43**
Alpha-synuclein	P37840|SYUA_HUMAN				0,83<br>0,68	0,71<br>0,80	0,49<br>0,64			
Putative uncharacterized protein UNQ6490/PRO21339 precursor	Q6UXU1|YC002_HUMAN	0,00	0,00							
Putative uncharacterized protein WASPIP	Q53TA9|Q53TA9_HUMAN				0,67	**0,66**	0,67	1,01	1,04	0,66
ATP-dependent DNA helicase 2 subunit 2	P13010|KU86_HUMAN				1,05	1,71	**1,77**	**0,63**	**1,43**	**1,50**
Pyridoxal kinase	O00764|PDXK_HUMAN				**0,82**	0,96	0,79			
Pyrroline-5-carboxylate reductase 2	Q96C36|P5CR2_HUMAN	1,01	0,88							
Pyruvate carboxylase, mitochondrial precursor	P11498|PYC_HUMAN	0,93<br>0,94	**0,88**<br>**0,89**	**0,80**	0,00	0,00	0,00			
Pyruvate dehydrogenase (Lipoamide) alpha 1	Q5JPT8|Q5JPT8_HUMAN	**0,89**	**0,89**	**0,76**						9999,00
Pyruvate dehydrogenase E1 component subunit beta, mitochondrial precursor	P11177|ODPB_HUMAN	0,94<br>0,99	0,94<br>0,94	**0,74**	0,00	0,00	0,00			
Pyruvate dehydrogenase phosphatase regulatory subunit, mitochondrial precursor	Q8NCN5|PDPR_HUMAN	1,07	1,09							
Pyruvate dehydrogenase protein X component, mitochondrial precursor	O00330|ODPX_HUMAN	**0,85**<br>1,01	0,95<br>1,00	**0,78**						
Pyruvate kinase	A6NFK3|A6NFK3_HUMAN					0,86	1,03			
Pyruvate kinase isozymes M1/M2	P14618|KPYM_HUMAN	1,21<br>**1,40**	**1,58**<br>**1,59**	**2,85**	0,00<br>0,99	0,00<br>1,18	0,00<br>1,24	**1,92**<br>0,37	**2,61**<br>1,07	**5,72**<br>0,96
QARS protein (Fragment)	Q9BUZ3|Q9BUZ3_HUMAN				0,83	1,08	**0,78**			
Quinone oxidoreductase	Q08257|QOR_HUMAN	0,95	0,89							
Rab11 family-interacting protein 1	Q6WKZ4|RFIP1_HUMAN	0,80<br>1,05	1,04<br>0,90	0,99	1,30<br>1,47	1,18<br>1,16	0,99<br>1,02	**0,77**	**0,47**	**0,28**
RAB11B protein	Q6FI42|Q6FI42_HUMAN	0,95<br>1,00	0,95<br>1,00	0,96	**1,56**<br>1,48	**1,26**<br>1,10	**1,34**<br>1,30	1,24<br>1,40	**3,59**<br>1,62	**3,73**<br>3,53
RAB13 protein (Fragment)	Q504R6|Q504R6_HUMAN	1,01<br>0,99	1,02<br>1,09	**1,29**						

<table>
<tr><th></th><th></th><th></th><th></th><th></th><th></th><th></th><th></th><th></th><th></th><th></th></tr>
<tr><td>RAB1A, member RAS oncogene family</td><td>Q5U0I6|Q5U0I6_HUMAN</td><td>0,93
0,99</td><td>0,85
1,14</td><td>0,97</td><td>**1,65**
1,54</td><td>**1,33**
1,20</td><td>1,17
1,01</td><td>1,45
0,77</td><td>3,38
3,01</td><td>**7,32**
6,20</td></tr>
<tr><td>RAB27A protein</td><td>Q6IAS8|Q6IAS8_HUMAN</td><td>
1,02</td><td>
1,24</td><td></td><td></td><td></td><td></td><td></td><td></td><td></td></tr>
<tr><td>RAB31, member RAS oncogene family variant (Fragment)</td><td>Q53EY4|Q53EY4_HUMAN</td><td></td><td></td><td></td><td>1,36</td><td>0,79</td><td>0,77</td><td></td><td></td><td></td></tr>
<tr><td>RAB5A protein</td><td>Q6FI44|Q6FI44_HUMAN</td><td>0,00
1,09</td><td>0,00
1,28</td><td>0,00</td><td>
1,60</td><td>
1,19</td><td>
1,27</td><td></td><td></td><td></td></tr>
<tr><td>RAB5B protein</td><td>Q6FI54|Q6FI54_HUMAN</td><td></td><td></td><td></td><td>
0,00</td><td>
0,00</td><td>
0,00</td><td></td><td></td><td></td></tr>
<tr><td>RAB5C protein</td><td>Q6FH55|Q6FH55_HUMAN</td><td>0,99
0,94</td><td>1,19
1,26</td><td>1,33</td><td>**2,37**
1,50</td><td>**1,42**
0,95</td><td>1,33
0,95</td><td>1,10
0,00</td><td>1,15
0,00</td><td>0,90
0,00</td></tr>
<tr><td>RAB6A, member RAS oncogene family isoform a variant (Fragment)</td><td>Q53ET8|Q53ET8_HUMAN</td><td>**1,14**</td><td>**0,81**</td><td>**0,84**</td><td></td><td></td><td></td><td></td><td></td><td></td></tr>
<tr><td>RAB8A protein</td><td>Q6FHV5|Q6FHV5_HUMAN</td><td>1,10
1,23</td><td>0,87
1,32</td><td>1,47</td><td></td><td></td><td></td><td></td><td></td><td></td></tr>
<tr><td>RABGGTB protein</td><td>Q6IB63|Q6IB63_HUMAN</td><td></td><td></td><td></td><td>
0,00</td><td>
0,00</td><td>
0,00</td><td></td><td></td><td></td></tr>
<tr><td>Rab GTPase-binding effector protein 2</td><td>Q9H5N1|RABE2_HUMAN</td><td></td><td></td><td></td><td>
0,00</td><td>
0,00</td><td>
0,00</td><td></td><td></td><td></td></tr>
<tr><td>Radical S-adenosyl methionine domain-containing protein 2</td><td>Q8WXG1|RSAD2_HUMAN</td><td>**4,71**</td><td>**7,63**</td><td>**8,41**</td><td></td><td></td><td></td><td></td><td></td><td></td></tr>
<tr><td>RAN binding protein 1 variant (Fragment)</td><td>Q53EY3|Q53EY3_HUMAN</td><td></td><td></td><td></td><td>1,01
0,00</td><td>1,10
0,00</td><td>0,79
0,00</td><td></td><td></td><td></td></tr>
<tr><td>RAN binding protein 5</td><td>Q86XC7|Q86XC7_HUMAN</td><td></td><td></td><td></td><td>
0,81</td><td>
0,85</td><td>
0,76</td><td></td><td></td><td></td></tr>
<tr><td>RAP1, GTP-GDP dissociation stimulator 1</td><td>Q9BUX6|Q9BUX6_HUMAN</td><td></td><td></td><td></td><td>
0,71</td><td>
0,89</td><td>
0,37</td><td></td><td></td><td></td></tr>
<tr><td>RAP1A, member of RAS oncogene family</td><td>Q5U0C3|Q5U0C3_HUMAN</td><td>0,00
1,11</td><td>0,00
1,10</td><td>0,00</td><td></td><td></td><td></td><td>**1,58**</td><td>**2,93**</td><td>**4,00**</td></tr>
<tr><td>Ras GTPase-activating protein-binding protein 2</td><td>Q9UN86|G3BP2_HUMAN</td><td></td><td></td><td></td><td>2,05
2,06</td><td>1,24
1,12</td><td>0,87
1,15</td><td>**0,70**</td><td>**0,43**</td><td>**0,23**</td></tr>
<tr><td>Ras GTPase-activating-like protein IQGAP1</td><td>P46940|IQGA1_HUMAN</td><td>**1,24**
1,74</td><td>0,96
1,15</td><td>**1,67**</td><td>**0,89**
1,09</td><td>1,05
1,22</td><td>0,94
1,04</td><td>**1,19**
0,38</td><td>**0,82**
0,62</td><td>1,18
0,56</td></tr>
<tr><td>Ras GTPase-activating-like protein IQGAP2</td><td>Q13576|IQGA2_HUMAN</td><td>1,31
1,07</td><td>**2,33**
0,94</td><td>**4,77**</td><td>
0,86</td><td>
1,10</td><td>
0,96</td><td></td><td></td><td></td></tr>
<tr><td>Ras homolog gene family, member A variant (Fragment)</td><td>Q53HM4|Q53HM4_HUMAN</td><td>1,04
1,46</td><td>**1,22**
1,53</td><td>**1,69**</td><td>1,06
1,04</td><td>**0,82**
0,87</td><td>**0,75**
0,59</td><td></td><td></td><td></td></tr>
<tr><td>Ras-related C3 botulinum toxin substrate 1</td><td>A4D2P0|A4D2P0_HUMAN</td><td></td><td></td><td></td><td></td><td></td><td></td><td>
0,53</td><td>
1,55</td><td>
1,75</td></tr>
<tr><td>Ras-related C3 botulinum toxin substrate 1 precursor</td><td>P63000|RAC1_HUMAN</td><td>
1,13</td><td>
0,85</td><td></td><td></td><td></td><td></td><td></td><td></td><td></td></tr>
<tr><td>Ras-related C3 botulinum toxin substrate 2 precursor</td><td>P15153|RAC2_HUMAN</td><td>0,95
1,22</td><td>**1,23**
1,40</td><td>**1,78**</td><td>1,24
1,32</td><td>0,88
0,69</td><td>**0,66**
0,60</td><td>**1,38**</td><td>**2,37**</td><td>**4,63**</td></tr>
<tr><td>Ras-related GTP-binding protein C</td><td>Q9HB90|RRAGC_HUMAN</td><td>3,55</td><td>6,36</td><td>6,85</td><td></td><td></td><td></td><td></td><td></td><td></td></tr>
<tr><td>Ras-related protein Rab-10</td><td>P61026|RAB10_HUMAN</td><td>1,08
1,02</td><td>1,10
1,04</td><td>1,00</td><td>1,72
1,84</td><td>1,15
1,17</td><td>1,35
1,24</td><td>1,37</td><td>1,82</td><td>**2,99**</td></tr>
<tr><td>Ras-related protein Rab-14</td><td>P61106|RAB14_HUMAN</td><td>1,06
1,09</td><td>1,16
1,07</td><td>**1,46**</td><td>
1,79</td><td>
1,32</td><td>
1,16</td><td>1,88</td><td>**3,18**</td><td>**6,54**</td></tr>
<tr><td>Ras-related protein Rab-18</td><td>Q9NP72|RAB18_HUMAN</td><td>0,96
0,93</td><td>0,93
0,89</td><td>1,15</td><td></td><td></td><td></td><td></td><td></td><td></td></tr>
<tr><td>Ras-related protein Rab-1B</td><td>Q9H0U4|RAB1B_HUMAN</td><td>1,03
1,28</td><td>1,07
1,28</td><td>**1,46**</td><td>1,27
1,58</td><td>0,90
1,06</td><td>0,83
0,90</td><td>1,13
1,24</td><td>**2,87**
3,24</td><td>**5,60**
5,38</td></tr>
<tr><td>Ras-related protein Rab-21</td><td>Q9UL25|RAB21_HUMAN</td><td>1,13
0,95</td><td>1,20
1,16</td><td>0,95</td><td></td><td></td><td></td><td></td><td></td><td></td></tr>
<tr><td>Ras-related protein Rab-24</td><td>Q969Q5|RAB24_HUMAN</td><td>
1,04</td><td>
0,83</td><td></td><td></td><td></td><td></td><td></td><td></td><td></td></tr>
<tr><td>Ras-related protein Rab-27A</td><td>P51159|RB27A_HUMAN</td><td>1,04</td><td>1,17</td><td>1,41</td><td></td><td></td><td></td><td></td><td></td><td></td></tr>
<tr><td>Ras-related protein Rab-2A</td><td>P61019|RAB2A_HUMAN</td><td>1,09
1,00</td><td>0,84
1,01</td><td>0,79</td><td></td><td></td><td></td><td></td><td></td><td></td></tr>
<tr><td>Ras-related protein Rab-31</td><td>Q13636|RAB31_HUMAN</td><td>0,89
1,07</td><td>**0,83**
1,05</td><td>1,09</td><td></td><td></td><td></td><td></td><td></td><td></td></tr>
<tr><td>Ras-related protein Rab-32</td><td>Q13637|RAB32_HUMAN</td><td>0,98
1,03</td><td>1,00
1,08</td><td>0,86</td><td></td><td></td><td></td><td></td><td></td><td></td></tr>
<tr><td>Ras-related protein Rab-35</td><td>Q15286|RAB35_HUMAN</td><td>1,17
1,08</td><td>1,00
1,12</td><td>1,38</td><td></td><td></td><td></td><td></td><td></td><td></td></tr>
<tr><td>Ras-related protein Rab-3D</td><td>O95716|RAB3D_HUMAN</td><td>1,16</td><td>1,23</td><td>1,97</td><td></td><td></td><td></td><td></td><td></td><td></td></tr>
<tr><td>Ras-related protein Rab-4B</td><td>P61018|RAB4B_HUMAN</td><td>1,28</td><td>1,37</td><td>**1,22**</td><td></td><td></td><td></td><td></td><td></td><td></td></tr>
<tr><td>Ras-related protein Rab-5B</td><td>P61020|RAB5B_HUMAN</td><td>0,84
1,02</td><td>1,54
0,74</td><td>**2,84**</td><td></td><td></td><td></td><td></td><td></td><td></td></tr>
<tr><td>Ras-related protein Rab-6A</td><td>P20340|RAB6A_HUMAN</td><td>
1,10</td><td>
0,98</td><td></td><td></td><td></td><td></td><td></td><td></td><td></td></tr>
<tr><td>Ras-related protein Rab-7a</td><td>P51149|RAB7A_HUMAN</td><td>0,99
1,01</td><td>0,97
1,07</td><td>0,93</td><td>**1,81**
2,07</td><td>0,83
1,13</td><td>1,08
1,18</td><td>0,83
0,83</td><td>**4,42**
2,83</td><td>**6,31**
5,36</td></tr>
<tr><td>Ras-related protein Rab-8B</td><td>Q92930|RAB8B_HUMAN</td><td>1,14
1,15</td><td>1,10
1,10</td><td>1,04</td><td></td><td></td><td></td><td></td><td></td><td></td></tr>
<tr><td>Ras-related protein Rab-9A</td><td>P51151|RAB9A_HUMAN</td><td>
0,82</td><td>
0,99</td><td></td><td></td><td></td><td></td><td></td><td></td><td></td></tr>
<tr><td>Ras-related protein Ral-A precursor</td><td>P11233|RALA_HUMAN</td><td>1,06
1,05</td><td>0,96
1,18</td><td>0,98</td><td></td><td></td><td></td><td>1,31</td><td>1,90</td><td>2,49</td></tr>
<tr><td>Ras-related protein Ral-B precursor</td><td>P11234|RALB_HUMAN</td><td>1,81
1,10</td><td>**1,50**
0,99</td><td>1,82</td><td></td><td></td><td></td><td></td><td></td><td></td></tr>
<tr><td>Ras-related protein Rap-1b precursor</td><td>P61224|RAP1B_HUMAN</td><td>1,20
1,30</td><td>1,25
1,24</td><td>1,56</td><td>**1,83**
2,45</td><td>**0,87**
1,25</td><td>0,89
1,19</td><td>
1,13</td><td>
1,93</td><td>
2,74</td></tr>
<tr><td>Ras-related protein Rap-2b precursor</td><td>P61225|RAP2B_HUMAN</td><td>
1,21</td><td>
1,04</td><td></td><td></td><td></td><td></td><td></td><td></td><td></td></tr>
<tr><td>RBM25 protein (Fragment)</td><td>A0PJL9|A0PJL9_HUMAN</td><td></td><td></td><td></td><td></td><td></td><td></td><td>
0,84</td><td>
0,74</td><td>
0,62</td></tr>
<tr><td>Receptor expression-enhancing protein 5</td><td>Q00765|REEP5_HUMAN</td><td>**0,63**
1,08</td><td>0,90
1,19</td><td>1,01</td><td>**1,72**
2,11</td><td>0,89
1,23</td><td>0,98
1,24</td><td></td><td></td><td></td></tr>
<tr><td>Receptor-type tyrosine-protein phosphatase eta precursor</td><td>Q12913|PTPRJ_HUMAN</td><td>
1,18</td><td>
1,12</td><td></td><td></td><td></td><td></td><td></td><td></td><td></td></tr>
<tr><td>Regulator of G-protein signaling 10</td><td>O43665|RGS10_HUMAN</td><td></td><td></td><td></td><td>0,85</td><td>1,03</td><td>0,76</td><td></td><td></td><td></td></tr>
<tr><td>Regulator of G-protein signaling 14</td><td>O43566|RGS14_HUMAN</td><td></td><td></td><td></td><td>
0,72</td><td>
0,61</td><td>
0,47</td><td></td><td></td><td></td></tr>
<tr><td>Renin receptor precursor</td><td>O75787|RENR_HUMAN</td><td>0,83
0,83</td><td>0,80
0,91</td><td>0,81</td><td></td><td></td><td></td><td>1,54
1,92</td><td>**3,08**
2,39</td><td>**3,70**
3,86</td></tr>
<tr><td>Replication protein A 70 kDa DNA-binding subunit</td><td>P27694|RFA1_HUMAN</td><td></td><td></td><td></td><td>0,85</td><td>0,98</td><td>0,89</td><td></td><td></td><td></td></tr>
<tr><td>RER1 protein</td><td>Q9P0H9|Q9P0H9_HUMAN</td><td>1,45
1,02</td><td>1,05
0,87</td><td>1,11</td><td></td><td></td><td></td><td></td><td></td><td></td></tr>
<tr><td>Ret finger protein-like 4A</td><td>A6NLU0|RFPLA_HUMAN</td><td></td><td></td><td></td><td>0,80</td><td>0,85</td><td>0,87</td><td></td><td></td><td></td></tr>
<tr><td>Reticulocalbin 2, EF-hand calcium binding domain</td><td>Q53XN8|Q53XN8_HUMAN</td><td>
0,98</td><td>
1,02</td><td></td><td></td><td></td><td></td><td></td><td></td><td></td></tr>
<tr><td>Reticulocalbin-1 precursor</td><td>Q15293|RCN1_HUMAN</td><td>0,92
1,03</td><td>0,95
1,14</td><td>0,88</td><td>
1,19</td><td>
1,28</td><td>
1,95</td><td></td><td></td><td></td></tr>
<tr><td>Reticulon-1</td><td>Q16799|RTN1_HUMAN</td><td>1,00
1,04</td><td>1,30
1,03</td><td>**1,71**</td><td>**2,10**
1,82</td><td>1,48
1,31</td><td>1,54
1,39</td><td></td><td></td><td></td></tr>
<tr><td>Reticulon-3</td><td>O95197|RTN3_HUMAN</td><td>0,70
0,93</td><td>0,86
1,39</td><td>1,04</td><td></td><td></td><td></td><td>**0,70**</td><td>2,10</td><td>**2,75**</td></tr>
<tr><td>Reticulon-4</td><td>Q9NQC3|RTN4_HUMAN</td><td>0,88
1,14</td><td>**1,35**
1,25</td><td>**1,92**</td><td>**2,44**
1,64</td><td>1,18
0,92</td><td>1,58
1,46</td><td>**2,71**
0,99</td><td>1,84
1,56</td><td>**2,30**
2,43</td></tr>
<tr><td>Retinal dehydrogenase 2</td><td>O94788|AL1A2_HUMAN</td><td></td><td></td><td></td><td>**0,82**</td><td>**1,30**</td><td>**1,22**</td><td></td><td></td><td></td></tr>
</table>

					1,01	**1,25**	**1,24**			
Retinoblastoma binding protein 7	Q5JP00|Q5JP00_HUMAN							0,64	**0,47**	0,47
Retinoblastoma-binding protein 6	Q7Z6E9|RBBP6_HUMAN							0,93<br>**1,36**	**0,36**<br>**0,66**	**0,26**<br>0,73
Retinoid-inducible serine carboxypeptidase precursor	A7BI36|A7BI36_HUMAN	0,96	**1,42**	**1,28**	**1,76**<br>**1,52**	0,94<br>1,07	**1,19**<br>1,10	1,06	1,86	2,04
Retinoid-inducible serine carboxypeptidase precursor	Q9HB40|RISC_HUMAN	1,07	**0,84**							
Retinol dehydrogenase 11	Q8TC12|RDH11_HUMAN	**0,73**<br>1,10	0,95<br>**1,27**	1,16						
Retinol dehydrogenase 13	Q8NBN7|RDH13_HUMAN	0,69<br>1,10	0,80<br>0,98	**0,55**						
Retinol dehydrogenase 14	Q9HBH5|RDH14_HUMAN	0,91<br>0,89	0,78<br>0,87	0,80						
RFTN1 protein (Fragment)	Q8N5I0|Q8N5I0_HUMAN	0,00<br>1,08	0,00<br>1,28	0,00						
RGS19 protein	Q6I9S5|Q6I9S5_HUMAN	0,00<br>1,30	0,00<br>1,13	0,00				1,86<br>1,20	0,54<br>0,62	0,34<br>0,88
Rho GDP-dissociation inhibitor 2	P52566|GDIR2_HUMAN	1,38<br>1,50	3,61<br>2,08	6,78	**0,92**<br>0,91	**0,83**<br>0,79	**0,85**<br>0,98	0,81	2,31	3,30
Rho GTPase-activating protein 1	Q07960|RHG01_HUMAN	2,65		4,66	0,64<br>0,79	**0,66**<br>0,85	**0,52**<br>**0,51**			
Rho GTPase-activating protein 17	Q68EM7|RHG17_HUMAN				**0,67**	0,93	1,06			
Rho GTPase-activating protein 18	Q8N392|RHG18_HUMAN				0,86<br>0,81	**0,70**<br>**0,72**	**0,59**<br>0,44			
Rho guanine nucleotide exchange factor 1	Q92888|ARHG1_HUMAN				**0,77**	0,75	**0,52**			
Rhomboid domain-containing protein 2	Q6NTF9|RHBD2_HUMAN	1,28<br>0,99	0,91<br>**0,82**	1,11						
Ribonuclease inhibitor	P13489|RINI_HUMAN				0,93	0,90	0,83			
Ribonuclease T2	A6XND5|A6XND5_HUMAN	**1,34**<br>1,01	1,86<br>0,90	1,43						
Ribophorin I variant (Fragment)	Q53EP4|Q53EP4_HUMAN	0,98<br>**0,92**	1,00<br>**1,13**	0,98				**1,20**<br>**0,53**	**2,29**<br>**1,92**	**5,87**<br>**2,86**
Ribophorin II	Q5JYR6|Q5JYR6_HUMAN							0,92	**2,49**	**4,60**
Ribosomal L1 domain-containing protein 1	O76021|RL1D1_HUMAN							**0,85**<br>**0,56**	**0,59**<br>**0,76**	**0,68**<br>0,81
Ribosomal protein L10 (Fragment)	Q5HY50|Q5HY50_HUMAN	1,25	**1,91**		1,03<br>1,18	**0,81**<br>**0,83**	**0,56**<br>**0,62**	0,93	**0,63**	0,64
Ribosomal protein L11	Q5VVD0|Q5VVD0_HUMAN				1,10<br>**1,21**	0,96<br>1,03	**0,72**<br>**1,22**	0,93<br>**0,68**	**0,80**<br>**0,66**	**0,55**<br>**0,54**
Ribosomal protein L12 variant (Fragment)	Q59FI9|Q59FI9_HUMAN	0,80<br>1,19	1,13<br>1,26	**1,45**	0,99<br>1,03	**0,84**<br>0,92	1,00<br>1,00	**0,81**	1,21	**1,42**
Ribosomal protein L14 variant (Fragment)								0,00	0,00	0,00
Ribosomal protein L15	Q642I1|Q642I1_HUMAN	0,95<br>**1,28**	1,22<br>1,33	**1,45**	1,35	0,83	1,22			
Ribosomal protein L15 (Fragment)	Q8N6E1|Q8N6E1_HUMAN				0,64	0,75	0,65	1,21<br>**0,33**	1,52<br>0,86	1,94<br>1,02
Ribosomal protein L18 (Fragment)	Q0QEW2|Q0QEW2_HUMAN				1,28	0,92	0,66			
Ribosomal protein L18a variant (Fragment)	Q53HD3|Q53HD3_HUMAN				1,18<br>1,38	1,02<br>1,14	1,12<br>1,00	1,48<br>**0,29**	1,38<br>0,65	**2,02**<br>0,77
Ribosomal protein L19 variant (Fragment)	Q53G49|Q53G49_HUMAN				**1,41**	1,01	1,13			
Ribosomal protein L27a	Q6NZ52|Q6NZ52_HUMAN	0,97	1,12	1,74	1,10	0,93	**0,75**	0,79	0,73	**0,52**
Ribosomal protein L29	Q6IPI1|Q6IPI1_HUMAN	0,72	1,22	**2,48**	0,95	**0,75**	0,86	0,95<br>0,99	1,03<br>1,24	0,81<br>**1,61**
Ribosomal protein L3	Q96QL0|Q96QL0_HUMAN	**0,79**	**1,29**	**1,90**	**1,19**	0,96	0,86	**1,49**<br>**0,33**	**1,50**<br>**0,84**	**2,07**<br>1,00
Ribosomal protein L4 variant (Fragment)	Q59GY2|Q59GY2_HUMAN	**0,81**	**1,23**	**1,85**	**0,86**<br>**1,23**	**0,70**<br>1,04	**0,63**<br>0,87	1,00<br>**0,42**	**1,39**<br>0,86	**1,58**<br>1,08
Ribosomal protein L5 variant (Fragment)	Q59GX9|Q59GX9_HUMAN	0,93<br>**1,58**	1,14<br>1,06	1,15	1,06<br>1,11	0,96<br>1,01	**0,81**<br>0,90	1,06<br>**0,43**	**1,28**<br>**0,70**	**1,47**<br>**0,67**
Ribosomal protein L7a	Q5T8U4|Q5T8U4_HUMAN	**0,78**<br>1,14	1,01<br>**1,39**	1,61	1,01<br>1,24	**0,73**<br>1,02	0,87<br>0,91	0,90<br>0,92	**1,57**<br>0,97	**1,30**<br>**1,15**
Ribosomal protein S18	Q5SUJ3|Q5SUJ3_HUMAN	1,17<br>1,26	**1,65**<br>1,50	**4,04**	**1,23**<br>1,16	0,90<br>0,88	**1,12**<br>1,03	**0,88**<br>**1,28**	**0,92**<br>1,02	**0,54**<br>1,00
Ribosomal protein S19 (Fragment)	Q8WVX7|Q8WVX7_HUMAN	1,04<br>1,28	1,25<br>1,38	**3,40**	**1,24**<br>1,05	**0,90**<br>1,02	1,00<br>1,21	0,95<br>**1,37**	**1,15**<br>**1,57**	**0,80**<br>**1,41**
Ribosomal protein S2	Q6IPX5|Q6IPX5_HUMAN	0,85	1,20		**1,32**	1,07	**1,23**			
Ribosomal protein S27	Q5T4L6|Q5T4L6_HUMAN	1,54<br>1,46	1,80<br>1,58	4,49	1,25	1,00	0,77	0,81<br>**0,51**	0,41<br>0,48	**0,44**<br>**0,44**
Ribosomal protein S27a	Q5RKT7|Q5RKT7_HUMAN							**0,84**	**0,18**	**0,11**
Ribosomal protein S3A	Q6NXR8|Q6NXR8_HUMAN	0,94	**1,78**	**3,88**	**1,27**<br>**1,12**	0,93<br>1,02	1,09<br>1,13	1,06<br>0,96	**1,22**<br>**1,23**	0,93<br>1,04
Ribosomal protein S4, X-linked X isoform variant (Fragment)	Q53HV1|Q53HV1_HUMAN	**0,82**	**1,38**							
Ribosomal protein S6	Q96DV6|Q96DV6_HUMAN	1,28<br>1,03	**2,33**<br>1,37	**7,70**	**1,36**<br>**1,44**	0,93<br>0,98	**0,85**<br>0,97	**0,58**	0,84	0,96
Ribosomal protein S6 kinase alpha-1	Q15418|KS6A1_HUMAN	1,16	2,40	5,46	0,66	0,51	0,25			
Ribosomal protein S6 kinase alpha-3	P51812|KS6A3_HUMAN				0,96	0,99	0,74			
Ribosomal protein S8	Q5JR95|Q5JR95_HUMAN	0,87	1,35	2,06	**1,26**<br>1,62	0,95<br>1,16	1,22<br>1,83	**0,44**	**0,50**	0,90
Ribosome recycling factor, mitochondrial precursor	Q96E11|RRFM_HUMAN	0,91<br>1,00	0,85<br>0,97	**0,64**	0,00	0,00	0,00	0,92<br>1,15	**0,70**<br>**1,24**	**0,65**<br>**1,36**
RIG homolog (Fragment)	Q9UDC2|Q9UDC2_HUMAN				1,12	0,91	1,13	0,94<br>1,10	0,90<br>**0,73**	**0,64**<br>0,81
RING and PHD-finger domain-containing protein KIAA1542	Q9P1Y6|K1542_HUMAN							0,87	0,69	0,63
RING finger and transmembrane domain-containing protein PTD016	Q5M7Z0|PTD16_HUMAN	1,70	1,54							
RING finger protein 170	Q96K19|RN170_HUMAN	0,00	0,00	0,00						
RING finger protein 214	Q8ND24|RN214_HUMAN				0,00	0,00	0,00			
RNA (guanine-9-)-methyltransferase domain-containing protein 1, mitochondrial precursor	Q7L0Y3|RG9D1_HUMAN	0,88<br>0,94	0,86<br>**0,89**	**0,69**						
RNA binding protein, autoantigenic (Fragment)	Q5QPL9|Q5QPL9_HUMAN							**0,53**<br>0,93	**0,16**<br>**0,33**	**0,08**<br>**0,23**
RNA methyltransferase-like protein 1	Q9HC36|RMTL1_HUMAN	1,16<br>1,04	0,68<br>**0,90**	0,65				0,00	0,00	0,00
RNA polymerase-associated protein LEO1	Q8WVC0|LEO1_HUMAN							1,11	0,54	0,28
RNA-binding motif protein X-linked-like 1	Q96E39|Q96E39_HUMAN							0,69<br>0,85	**0,59**<br>**0,55**	**0,27**<br>**0,36**
RNA-binding protein 14	Q96PK6|RBM14_HUMAN							0,97<br>0,95	**0,38**<br>0,92	**0,24**<br>0,67
RNA-binding protein 26	Q5T8P6|RBM26_HUMAN				0,00	0,00	0,00	0,96<br>1,41	0,40<br>0,85	**0,23**<br>0,53

RNA-binding protein 27	Q9P2N5|RBM27_HUMAN							0,96	0,36	0,19
RNA-binding protein 28	Q9NW13|RBM28_HUMAN							0,50	0,28	**0,32**
RNA-binding protein 42	Q9BTD8|RBM42_HUMAN							1,21<br>1,22	**0,43**<br>0,80	**0,26**<br>0,61
RNA-binding protein 4B	Q9BQ04|RBM4B_HUMAN							0,73	**0,17**	0,14
RNA-binding protein 8A	Q9Y5S9|RBM8A_HUMAN				0,80<br>0,94	1,25<br>**1,58**	1,17<br>1,59	0,95<br>**1,21**	**0,29**<br>**0,41**	**0,18**<br>**0,37**
RNA-binding protein Nova-2	Q9UNW9|NOVA2_HUMAN							**0,58**	**0,27**	**0,30**
RNA-binding protein Raly	Q9UKM9|RALY_HUMAN	0,00	0,00	0,00				1,03	**0,20**	0,20
RNA-binding protein with serine-rich domain 1	Q15287|RNPS1_HUMAN							1,08<br>1,83	0,82<br>1,44	**0,28**<br>1,27
RNA-directed RNA polymerase catalytic subunit	Q9WLS3|RDRP_I97A1	0,00	0,00	0,00						
RNA-directed RNA polymerase catalytic subunit	Q0A461|RDRP_I68A3	1,34	1,99	3,64						
RNF13 protein	Q6IBJ9|Q6IBJ9_HUMAN	0,00	0,00							
RNH1 protein (Fragment)	Q96FD7|Q96FD7_HUMAN				**0,77**	**0,84**	**0,65**			
RNP24 protein	Q6FHT8|Q6FHT8_HUMAN	**1,23**<br>**0,84**	1,01<br>0,98	1,07				0,83	**5,25**	**5,97**
Rootletin	Q5TZA2|CROCC_HUMAN				1,03	0,85	1,03	1,01	0,85	1,58
RPL13A protein (Fragment)	Q9BSQ6|Q9BSQ6_HUMAN	0,86	1,28	**1,98**	1,09	0,87	**0,75**	0,95<br>**0,77**	**1,47**<br>**0,76**	0,92<br>0,81
RPL14 protein	Q6IPH7|Q6IPH7_HUMAN	1,03	1,30	1,42	0,00<br>0,00	0,00<br>0,00	0,00<br>0,00	1,02	1,02	1,09
RPL7 protein	Q3KQU0|Q3KQU0_HUMAN	**1,18**	**1,22**		1,02	**0,80**	**0,78**	**0,81**<br>0,95	**1,53**<br>**0,73**	0,92<br>0,97
RPLP1 protein (Fragment)	Q59FV6|Q59FV6_HUMAN							0,88	**2,09**	**1,91**
RPLP2 protein	Q6FG96|Q6FG96_HUMAN	0,90<br>**1,16**	0,94<br>**1,33**	0,81	1,16<br>0,78	**0,81**<br>0,68	1,15<br>0,80	**0,79**<br>**1,32**	**1,75**<br>**2,73**	**1,35**<br>**2,89**
RPN1 protein	Q6IBR0|Q6IBR0_HUMAN				2,96<br>**2,23**	1,16<br>1,22	1,23<br>**1,39**			
RPS21 protein	Q8WVC2|Q8WVC2_HUMAN				**1,24**<br>0,92	1,01<br>**0,69**	**1,21**<br>0,82	**0,88**<br>**1,29**	0,87<br>1,09	**0,51**<br>**0,81**
RPS4X protein (Fragment)	Q96IR1|Q96IR1_HUMAN	0,97	**1,56**	**3,06**	1,53	1,01	**1,35**	0,90<br>**0,84**	**0,69**<br>**0,51**	**0,56**<br>**0,60**
RRAS protein	Q6FH12|Q6FH12_HUMAN	1,01	1,07							
RRBP1 protein	P54577|SYYC_HUMAN							0,68	2,38	2,20
rRNA 2'-O-methyltransferase fibrillarin	P22087|FBRL_HUMAN							**1,18**<br>**0,56**	**0,89**<br>0,96	**0,67**<br>0,91
RRP1 protein (Fragment)	Q6PJJ2|Q6PJJ2_HUMAN							0,88<br>1,00	0,38<br>0,46	0,16<br>0,38
RRP12-like protein	Q5JTH9|RRP12_HUMAN							1,17<br>0,73	1,03<br>0,96	1,06<br>0,73
RRP1-like protein B	Q14684|RRP1B_HUMAN							**0,72**<br>1,31	**0,37**<br>**2,06**	**0,13**<br>0,93
RSU1 protein	Q6FI17|Q6FI17_HUMAN				**0,76**<br>0,73	0,87<br>1,00	0,86<br>0,87	1,08	1,16	**0,79**
RSU1 protein (Fragment)	Q32Q10|Q32Q10_HUMAN							0,00	0,00	0,00
RuvB-like 2	Q9Y230|RUVB2_HUMAN				0,76	0,92	0,70	0,82<br>0,84	**0,63**<br>0,69	**0,51**<br>0,76
S100 calcium binding protein A1	Q5T7Y6|Q5T7Y6_HUMAN				0,75	**1,38**	1,60			
S-adenosylmethionine synthetase isoform type-2	P31153|METK2_HUMAN				0,92<br>0,97	**1,13**<br>**1,53**	0,96<br>**1,39**			
SAFB-like transcription modulator	Q9NWH9|SLTM_HUMAN							**0,79**<br>**0,85**	**0,37**<br>**0,52**	**0,24**<br>**0,47**
SAM domain and HD domain-containing protein 1	Q9Y3Z3|SAMH1_HUMAN	1,10<br>**1,20**	**2,13**<br>**1,35**	**3,62**	0,92<br>**0,79**	0,97<br>0,94	**0,58**<br>0,50	0,86<br>**0,47**	1,26<br>**1,29**	**1,75**<br>0,94
SAPK substrate protein 1	Q04323|SAKS1_HUMAN				0,78<br>0,61	0,71<br>0,48	0,67<br>0,32			
SAPS domain family member 1	Q9UPN7|SAPS1_HUMAN				1,06	1,03	1,09			
Sarcoplasmic/endoplasmic reticulum calcium ATPase 2	P16615|AT2A2_HUMAN	0,94<br>0,95	1,11<br>**1,12**	1,09	1,78	1,01	1,23			
Sarcosine dehydrogenase, mitochondrial precursor	Q9UL12|SARDH_HUMAN	0,90	**0,88**	**0,73**						
Scaffold attachment factor B	A0AV56|A0AV56_HUMAN							**0,71**	**0,40**	**0,15**
Scaffold attachment factor B1	Q15424|SAFB1_HUMAN							**0,75**	**0,50**	**0,36**
Scaffold attachment factor B2	Q14151|SAFB2_HUMAN							0,74<br>**0,70**	**0,31**<br>**0,43**	**0,19**<br>**0,35**
SCAMP1 protein (Fragment)	Q6FG23|Q6FG23_HUMAN	1,27	1,21	1,24						
SCAMP2 protein	Q6IBK3|Q6IBK3_HUMAN	1,06<br>1,07	1,04<br>0,98	1,04	0,00	0,00	0,00			
Scavenger receptor class B, member 2	Q53Y63|Q53Y63_HUMAN	0,95<br>0,98	1,04<br>1,03	**0,89**	**2,08**	0,87	1,13			
Scavenger receptor cysteine-rich type 1 protein M130 precursor	Q86VB7|C163A_HUMAN	1,17<br>1,02	0,90<br>**0,81**	0,79						
SCO1 protein homolog, mitochondrial precursor	O75880|SCO1_HUMAN	0,88<br>1,00	0,86<br>0,97	**0,75**						
SCO2 protein homolog, mitochondrial precursor	O43819|SCO2_HUMAN	1,16<br>0,86	0,80<br>0,90	0,84						
Sec23 homolog B	Q503A9|Q503A9_HUMAN				0,00<br>0,00	0,00<br>0,00	0,00<br>0,00			
Secretory carrier-associated membrane protein 4	Q969E2|SCAM4_HUMAN	1,24<br>1,04	0,81<br>0,91	0,99						
Secretory phospholipase A2 receptor precursor	Q13018|PLA2R_HUMAN	0,00	0,00	0,00						
Selenoprotein H	Q8IZQ5|SELH_HUMAN							0,00	0,00	0,00
Selenoprotein N precursor	Q9NZV5|SELN_HUMAN	1,21<br>0,00	0,81<br>0,00	0,75						
Sepiapterin reductase	P35270|SPRE_HUMAN	1,04<br>1,00	0,93<br>0,96	0,81						
Septin	Q3LIE9|Q3LIE9_HUMAN							**0,48**	**0,45**	**0,30**
Septin (Fragment)	A4GYY8|A4GYY8_HUMAN				0,92	1,23	**1,46**			
Septin 7	Q8TC62|Q8TC62_HUMAN							0,98	**0,51**	**0,32**
Septin-11	Q9NVA2|SEP11_HUMAN							0,47	0,43	0,31
Septin-2	Q15019|SEPT2_HUMAN				1,10	**1,52**	**1,77**	0,97<br>1,20	**0,52**<br>0,61	**0,42**<br>**0,52**
Septin-7	Q16181|SEPT7_HUMAN				**0,77**	**1,31**	**1,65**			

Septin-9	Q9UHD8|SEPT9_HUMAN				0,80<br>0,53	1,11<br>0,90	0,87<br>0,85	**0,86**<br>0,96	**0,41**<br>**0,72**	**0,26**<br>**0,51**
Serine beta-lactamase-like protein LACTB, mitochondrial precursor	P83111|LACTB_HUMAN	**0,87**<br>**0,93**	0,95<br>**0,86**	**0,82**				0,94<br>**0,45**	1,08<br>0,93	**1,54**<br>**1,24**
Serine hydroxymethyltransferase	Q8N1A5|Q8N1A5_HUMAN	0,96	0,91	**0,73**	**1,72**<br>2,15	**1,47**<br>**1,42**	**2,60**<br>**2,04**			
Serine hydroxymethyltransferase (Fragment)	Q5BJF5|Q5BJF5_HUMAN							1,23<br>0,71	**1,69**<br>**1,90**	**3,18**<br>**2,62**
Serine hydroxymethyltransferase (Fragment)	Q53ET4|Q53ET4_HUMAN	0,95	0,96							
Serine incorporator 1	Q9NRX5|SERC1_HUMAN	0,00<br>0,00	0,00<br>0,00	0,00						
Serine palmitoyltransferase 1	O15269|SPTC1_HUMAN	1,09<br>0,92	0,99<br>1,11	0,94						
Serine protease HTRA2, mitochondrial precursor	O43464|HTRA2_HUMAN	0,90	0,99							
Serine/arginine repetitive matrix protein 2	Q9UQ35|SRRM2_HUMAN				1,18	1,67	2,03	**0,79**<br>**1,24**	**0,59**<br>**1,59**	**0,37**<br>1,13
Serine/threonine kinase 10	Q6NSK0|Q6NSK0_HUMAN	1,49	1,81	2,63				**1,25**<br>0,97	**2,24**<br>**2,24**	**1,48**<br>3,02
Serine/threonine kinase 4 variant (Fragment)	Q59FZ4|Q59FZ4_HUMAN				1,04	0,87	0,79			
Serine/threonine protein phosphatase<br>(protein phosphatase 2, catalytic subunit, alpha isozyme)	Q6I9T8|Q6I9T8_HUMAN				0,69	**0,90**	0,75			
Serine/threonine protein phosphatase	Q07161|Q07161_HUMAN				0,89	0,91	0,71			
Serine/threonine protein phosphatase	Q8WZ56|Q8WZ56_HUMAN				0,97	1,09	1,06			
Serine/threonine protein phosphatase	A8K3B7|A8K3B7_HUMAN				0,93	0,94	0,91			
Serine/threonine protein phosphatase	A8W6Z7|A8W6Z7_HUMAN				0,73	0,60	0,59			
Serine/threonine-protein phosphatase PP1-alpha catalytic subunit	P62136|PP1A_HUMAN	1,81	**1,35**							
Serine/threonine protein phosphatase (Fragment)	Q9BPW0|Q9BPW0_HUMAN				1,03<br>**0,82**	0,98<br>**0,86**	0,95<br>1,02			
Serine/threonine-protein kinase 10	O94804|STK10_HUMAN				0,75<br>**0,79**	**0,65**<br>0,85	**0,59**<br>0,76			
Serine/threonine-protein kinase 3	Q13188|STK3_HUMAN				0,00	0,00	0,00			
Serine/threonine-protein kinase MST4	Q9P289|MST4_HUMAN				0,68	1,36	0,75			
Serine/threonine-protein kinase N1	Q16512|PKN1_HUMAN				0,78	1,05	0,96			
Serine/threonine-protein kinase OSR1	O95747|OXSR1_HUMAN				0,75	0,80	0,76			
Serine/threonine-protein kinase PAK 2	Q13177|PAK2_HUMAN				0,73<br>**0,70**	0,88<br>**0,82**	**0,69**<br>**0,58**			
Serine/threonine-protein kinase VRK1	Q99986|VRK1_HUMAN							0,91<br>0,86	**0,30**<br>0,70	**0,21**<br>0,70
Serine/threonine-protein phosphatase 1 regulatory subunit 10	Q96QC0|PP1RA_HUMAN							0,92<br>1,03	**0,38**<br>0,93	**0,16**<br>**0,55**
Serine/threonine-protein phosphatase 2A 65 kDa regulatory subunit A alpha isoform	P30153|2AAA_HUMAN				**0,84**<br>0,75	0,90<br>**0,88**	1,00<br>**0,79**			
Serine/threonine-protein phosphatase PP1-alpha catalytic subunit	P62136|PP1A_HUMAN				**1,26**	**1,30**	**1,33**	1,28	**0,54**	**0,54**
Serine-threonine kinase receptor-associated protein	Q9Y3F4|STRAP_HUMAN				0,70	0,88	0,71			
Serpin H1 precursor	P50454|SERPH_HUMAN	0,98<br>1,13	1,01<br>1,08	1,00				0,00	0,00	0,00
Serum albumin	Q56G89|Q56G89_HUMAN				3,00	1,78	8,68			
Serum albumin precursor	P02768|ALBU_HUMAN	**1,15**<br>**1,36**	**0,87**<br>**1,44**	**1,64**	**2,72**	**1,44**	**6,76**			
Serum response factor-binding protein 1	Q8NEF9|SRFB1_HUMAN							0,89	**0,33**	**0,22**
Seryl-tRNA synthetase, mitochondrial precursor	Q9NP81|SYSM_HUMAN	0,98<br>0,92	1,02<br>0,94	0,84						
Ses2 protein (Fragment)	Q9BU04|Q9BU04_HUMAN	1,20	0,86							
SET domain-containing protein 3	Q86TU7|SETD3_HUMAN				0,00<br>1,16	0,00<br>1,05	0,00<br>1,09			
SET translocation	Q5VXV3|Q5VXV3_HUMAN				0,81<br>0,74	1,06<br>1,56	0,78<br>0,59	**1,99**<br>**1,84**	**4,44**<br>**7,30**	**2,65**<br>**5,21**
SF3B2 protein (Fragment)	Q9BWD2|Q9BWD2_HUMAN				1,79<br>0,00	3,35<br>0,00	**2,59**<br>0,00	**0,91**<br>1,00	**0,31**<br>**0,51**	**0,21**<br>**0,43**
S-formylglutathione hydrolase	P10768|ESTD_HUMAN				0,89	1,07	1,00			
SFT2 domain containing 2	Q5TIH2|Q5TIH2_HUMAN				0,00	0,00	0,00			
SGTA protein	Q6FIA9|Q6FIA9_HUMAN				0,51	0,85	0,55			
SH3 domain-binding glutamic acid-rich-like protein	O75368|SH3L1_HUMAN				**0,82**<br>0,64	0,98<br>0,77	**0,82**<br>**0,72**	1,21	2,52	2,90
SH3 domain-binding protein 1	Q9Y3L3|3BP1_HUMAN				**0,64**	0,71	0,73			
SH3 protein expressed in lymphocytes homolog	O75995|SLY_HUMAN				**0,75**	0,77	0,68			
Shootin-1	A0MZ66|SHOT1_HUMAN				**0,71**	**0,75**	**0,55**			
Short-chain specific acyl-CoA dehydrogenase, mitochondrial precursor	P16219|ACADS_HUMAN	**0,79**<br>0,98	**0,75**<br>0,94	0,80						
Sialate O-acetylesterase precursor	Q9HAT2|SIAE_HUMAN	1,08	0,99							
Sialic acid synthase	Q9NR45|SIAS_HUMAN				0,85	**0,81**	0,82			
Sialic acid-binding Ig-like lectin 7 precursor	Q9Y286|SIGL7_HUMAN	0,00<br>0,00	0,00<br>0,00	0,00						
Sialic acid-binding Ig-like lectin 9 precursor	Q9Y336|SIGL9_HUMAN	0,00<br>0,98	0,00<br>0,81	0,00						
Sialin	Q9NRA2|S17A5_HUMAN	1,05	0,99							
Sialidase-1 precursor	Q99519|NEUR1_HUMAN	0,00<br>1,00	0,00<br>0,88	0,00						
Sideroflexin-1	Q9H9B4|SFXN1_HUMAN	0,85<br>0,92	0,81<br>**0,89**	0,80						
Sideroflexin-2	Q96NB2|SFXN2_HUMAN	0,98	0,96							
Sideroflexin-3	Q9BWM7|SFXN3_HUMAN	0,95<br>1,02	**0,87**<br>**0,85**	**0,76**						
Sideroflexin-4	Q6P4A7|SFXN4_HUMAN	1,01	1,08							
Sideroflexin-5	Q8TD22|SFXN5_HUMAN	1,07<br>0,98	0,96<br>0,94	0,84						
SIDT2 protein	Q24JR2|Q24JR2_HUMAN	0,97	0,94							
Sigma 1-type opioid receptor	Q99720|OPRS1_HUMAN	**0,45**	1,10	1,15						

Signal peptidase complex SPC-18	Q7Z4Z6|Q7Z4Z6_HUMAN							**1,99**<br>1,44	**3,64**<br>1,74	**3,78**<br>**3,17**
Signal peptidase complex subunit 1	Q9Y6A9|SPCS1_HUMAN	0,00<br>1,17	0,00<br>1,03	0,00						
Signal peptidase complex subunit 2	Q15005|SPCS2_HUMAN	1,00<br>0,98	**1,18**<br>**1,15**	1,12				1,10<br>1,04	**3,76**<br>**2,75**	**5,54**<br>**4,37**
Signal peptidase complex subunit 3	P61009|SPCS3_HUMAN	0,94<br>0,79	0,80<br>1,17	**0,78**				0,94	**2,35**	**2,50**
Signal peptide peptidase beta	Q15K36|Q15K36_HUMAN				**1,81**	0,97	0,98			
Signal recognition particle 14 kDa protein	P37108|SRP14_HUMAN	0,91	1,10	1,32						
Signal recognition particle 14kD	Q96Q14|Q96Q14_HUMAN				1,16<br>0,92	1,14<br>1,06	1,14<br>1,01	**1,37**<br>**1,62**	**1,50**<br>**1,37**	1,12<br>**1,55**
Signal recognition particle 19 kDa protein	P09132|SRP19_HUMAN							0,00	0,00	0,00
Signal recognition particle 9 kDa protein	P49458|SRP09_HUMAN	1,24	1,49		0,00<br>0,66	0,00<br>0,87	0,00<br>0,81	1,18<br>**2,10**	1,12<br>1,57	0,99<br>**1,82**
Signal recognition particle receptor subunit beta	Q9Y5M8|SRPRB_HUMAN	1,11<br>0,96	1,29<br>1,06	1,40	0,00	0,00	0,00	0,95<br>0,77	1,32<br>0,24	1,18<br>0,29
Signal-regulatory protein alpha	A2A2E1|A2A2E1_HUMAN	1,20<br>1,21	0,74<br>0,98	0,66	1,33	**0,78**	0,84			
Signal transducer and activator of transcription 1-alpha/beta	P42224|STAT1_HUMAN				0,93<br>1,12	1,05<br>1,13	0,84<br>1,21			
Similar to 60S ribosomal protein L35	A4D2M5|A4D2M5_HUMAN				1,10	0,95	0,83	**1,49**	0,92	1,05
Similar to AFG3 ATPase family gene 3-like 2 (Fragment)	Q8TA92|Q8TA92_HUMAN	**0,83**<br>1,02	0,95<br>0,95	0,90						
Similar to Chain , Heat-Shock Cognate 70kd Protein	A4D111|A4D111_HUMAN				0,00	0,00	0,00			
Similar to ribosomal protein L23 (Fragment)	Q9BTQ7|Q9BTQ7_HUMAN	1,01	**2,62**	**4,97**	1,30	1,30	0,96	0,83	**0,47**	**0,33**
Similar to ribosomal protein L23 (Fragment)	Q9BTQ7|Q9BTQ7_HUMAN				1,32	0,77	0,58			
Similar to ribosomal protein S8 (Fragment)	Q9BS10|Q9BS10_HUMAN	0,97	1,38					0,94	1,10	1,36
Similar to RIKEN cDNA C030006K11 gene	Q6P2Q7|Q6P2Q7_HUMAN	1,02	0,95							
Similar to Splicing factor, arginine/serine-rich, 46kD	A4D2F6|A4D2F6_HUMAN	0,84	1,07	1,34						
Single-stranded DNA binding protein 1	Q567R6|Q567R6_HUMAN	**0,74**	**0,72**	**0,63**	**2,24**	1,37	**1,66**	**1,94**	**2,32**	**3,17**
Single-stranded DNA-binding protein, mitochondrial precursor	Q04837|SSB_HUMAN	**0,93**	**0,83**					**1,61**	**2,52**	**2,51**
Sirtuin (Silent mating type information regulation 2 homolog) 5	Q5T295|Q5T295_HUMAN	0,00	0,00	0,00						
Sister chromatid cohesion protein PDS5 homolog A	Q29RF7|PDS5A_HUMAN							**0,77**<br>**1,31**	**0,55**<br>1,23	**0,37**<br>0,76
Sister chromatid cohesion protein PDS5 homolog B	Q9NTI5|PDS5B_HUMAN							0,84<br>0,90	0,58<br>0,99	0,60<br>1,01
Sjogren syndrome antigen B	Q53XJ4|Q53XJ4_HUMAN				1,02<br>0,89	**1,58**<br>**1,26**	**1,30**<br>1,13	1,23	1,26	0,92
SLAM family member 5 precursor	Q9UIB8|SLAF5_HUMAN	1,15<br>**1,21**	0,58<br>0,97	0,59						
SLC25A11 protein	Q6IBH0|Q6IBH0_HUMAN	1,01<br>0,92	0,98<br>**0,91**	0,90				0,00	0,00	0,00
SLC25A5 protein (Fragment)	Q6NVC0|Q6NVC0_HUMAN				**2,28**<br>2,37	**1,17**<br>**1,24**	1,15<br>**1,23**	**0,83**	**1,50**	**3,04**
SLC4A2 protein	Q8TAG3|Q8TAG3_HUMAN	**1,23**	1,07							
Small EDRK-rich factor 2	P84101|SERF2_HUMAN				0,64<br>0,53	0,42<br>0,50	0,34<br>0,48	**0,64**<br>0,85	**0,21**<br>0,56	**0,18**<br>0,57
Small nuclear ribonucleoprotein component	Q8IXJ3|Q8IXJ3_HUMAN				0,00	0,00	0,00			
Small nuclear ribonucleoprotein E	P62304|RUXE_HUMAN							0,00<br>2,02	0,00	0,00<br>0,32
Small nuclear ribonucleoprotein F	P62306|RUXF_HUMAN							0,65	0,08	0,04
Small nuclear ribonucleoprotein polypeptide A' variant (Fragment)	Q53G61|Q53G61_HUMAN							0,91<br>1,01	**0,40**<br>**0,50**	**0,24**<br>**0,39**
Small nuclear ribonucleoprotein polypeptide C	Q5TAL4|Q5TAL4_HUMAN							0,94<br>1,82	**0,36**<br>0,52	**0,13**<br>0,61
Small nuclear ribonucleoprotein Sm D2	P62316|SMD2_HUMAN				1,01<br>1,39	2,09<br>3,73	2,10<br>2,71	1,01<br>1,24	**0,49**<br>**0,51**	**0,23**<br>**0,46**
Small nuclear ribonucleoprotein Sm D3	P62318|SMD3_HUMAN							**0,77**	**0,54**	0,41
Small ubiquitin-related modifier 1 precursor	P63165|SUMO1_HUMAN							0,86<br>**1,29**	**0,36**<br>**0,65**	0,20<br>**0,30**
Small ubiquitin-related modifier 2 precursor	P61956|SUMO2_HUMAN				1,00<br>**0,66**	0,96<br>**0,78**	0,84<br>**0,67**	1,10<br>**1,38**	**0,45**<br>0,93	**0,13**<br>**0,64**
Small ubiquitin-related modifier 3 precursor	P55854|SUMO3_HUMAN				0,59	0,76	0,56			
SMARCA4 isoform 2	Q9HBD4|Q9HBD4_HUMAN							0,00	0,00	0,00
Sn1-specific diacylglycerol lipase beta	Q8NCG7|DGLB_HUMAN	0,84<br>0,91	0,85<br>1,03	0,79						
Snare-associated protein Snapin	O95295|S25BP_HUMAN	1,16	0,99							
SNRPB protein	Q6IB35|Q6IB35_HUMAN							**0,80**	**0,39**	**0,18**
SNRPB protein	Q6PKB4|Q6PKB4_HUMAN							**2,22**	**0,64**	0,54
SNRPG protein	Q6IB86|Q6IB86_HUMAN							**0,70**	**0,34**	**0,19**
SNW1 protein	Q6I9S2|Q6I9S2_HUMAN							**0,59**<br>0,91	**0,24**<br>**0,47**	**0,13**<br>**0,34**
SNX5 protein (Fragment)	Q6P5V6|Q6P5V6_HUMAN				0,51	0,66	0,58			
Sodium/hydrogen exchanger 7	Q96T83|SL9A7_HUMAN	1,01	1,04							
Sodium/potassium-transporting ATPase subunit alpha-1 precursor	P05023|AT1A1_HUMAN	**1,42**<br>1,02	**1,10**<br>**1,08**	**1,30**	**1,95**<br>**2,22**	**1,60**<br>**1,40**	1,36<br>**2,06**			
Sodium/potassium-transporting ATPase subunit beta-3	Q32Q14|Q32Q14_HUMAN	**1,44**	1,12	**1,27**	**1,48**	0,99	1,13	1,44	1,57	1,83
Sodium/potassium-transporting ATPase subunit beta-3	P54709|AT1B3_HUMAN	0,99	**1,13**							
Solute carrier family 1, member 4 variant (Fragment)	Q53F03|Q53F03_HUMAN	**1,32**	1,00	1,25						
Solute carrier family 12 (Potassium/chloride transporters), member 6	A0AV76|A0AV76_HUMAN	0,96	0,93	0,94						
Solute carrier family 12 member 6	Q9UHW9|S12A6_HUMAN	1,09	1,02							
Solute carrier family 12 member 7	Q9Y666|S12A7_HUMAN	1,52<br>1,04	2,12<br>0,97	4,57						
Solute carrier family 16, member 1	Q5T8R6|Q5T8R6_HUMAN	2,48<br>1,08	2,15<br>1,16	2,23						
Solute carrier family 16, member 3 variant (Fragment)	Q1HE25|Q1HE25_HUMAN				1,61	1,21	**1,53**			
Solute carrier family 2, facilitated glucose transporter member	P11169|GTR3_HUMAN	1,14	1,28	1,22						

3		1,06	0,97							
Solute carrier family 2, facilitated glucose transporter member 6	Q9UGQ3|GTR6_HUMAN	0,98	0,84							
Solute carrier family 22 member 18	Q96BI1|S22AI_HUMAN	0,83<br>0,94	1,12<br>1,06	1,18						
Solute carrier family 25 (Mitochondrial carrier, Aralar), member 12	Q96AM8|Q96AM8_HUMAN	0,89<br>0,90	1,07<br>0,97	0,89						
Solute carrier family 25 member 35	Q3KQZ1|S2535_HUMAN	0,86<br>0,82	0,76<br>0,76	0,62						
Solute carrier family 25 member 40	Q8TBP6|S2540_HUMAN	1,13<br>0,97	1,19<br>0,81	0,83						
Solute carrier family 25 member 46	Q96AG3|S2546_HUMAN	0,86	0,84							
Solute carrier family 28 member 3	Q9HAS3|S28A3_HUMAN	1,14	0,58							
Solute carrier family 35 member E1	Q96K37|S35E1_HUMAN	0,00	0,00							
Solute carrier family 43 member 3	Q8NBI5|S43A3_HUMAN	1,40	1,10	1,58	0,00<br>1,47	0,00<br>1,03	0,00<br>1,07			
Solute carrier family 6 (Neurotransmitter transporter, taurine), member 6 variant (Fragment)	Q59GD7|Q59GD7_HUMAN	0,97	0,85							
Solute carrier family 7 (Cationic amino acid transporter, y+ system), member 5	Q8IV97|Q8IV97_HUMAN	1,52<br>1,16	1,12<br>0,91	1,07						
SON protein	P18583|SON_HUMAN							0,82<br>0,84	**0,46**<br>0,77	**0,32**<br>**0,53**
Sorbitol dehydrogenase	Q00796|DHSO_HUMAN				0,99<br>0,87	0,87<br>0,85	0,72<br>0,82			
Sorcin	P30626|SORCN_HUMAN				0,99	1,00	**1,60**			
Sortilin precursor	Q99523|SORT_HUMAN	1,05<br>**0,79**	**0,67**<br>**0,76**	**0,70**						
Sorting and assembly machinery component 50 homolog	Q9Y512|SAM50_HUMAN	**1,34**<br>0,95	1,11<br>**1,07**	0,93						
Sorting nexin 1 isoform a variant (Fragment)	Q53HL9|Q53HL9_HUMAN				0,66	0,89	0,61			
Sorting nexin 2 variant (Fragment)	Q53GG3|Q53GG3_HUMAN				**0,72**<br>0,85	**0,78**<br>0,79	**0,81**<br>**0,70**			
Sorting nexin 3	Q4TT31|Q4TT31_HUMAN				0,79<br>0,71	0,66<br>0,85	0,58<br>0,71			
Sorting nexin 5 variant (Fragment)	Q53FH8|Q53FH8_HUMAN				**0,61**	0,72	0,61			
Sorting nexin-12	Q9UMY4|SNX12_HUMAN				0,92	0,85	0,72			
Sorting nexin-6	Q9UNH7|SNX6_HUMAN	0,00	0,00	0,00	0,91<br>0,00	0,93<br>0,00	1,03<br>0,00			
SPARC protein	Q6IBK4|Q6IBK4_HUMAN	**0,78**	0,90							
Spatacsin	Q96JI7|SPTCS_HUMAN	0,94	0,93							
SPC18 protein	Q6IAM7|Q6IAM7_HUMAN	0,94<br>0,98	0,83<br>0,96	0,90						
Spectrin SH3 domain binding protein 1								0,00	0,00	0,00
Spermatogenesis-associated protein 20 precursor	Q8TB22|SPT20_HUMAN	0,94	1,01							
Spermine synthase	P52788|SPSY_HUMAN				1,17	1,19	0,93			
S-phase kinase-associated protein 1	P63208|SKP1_HUMAN				0,79	**0,74**	0,66	0,86	**0,45**	**0,44**
Sphingosine-1-phosphate lyase 1	O95470|SGPL1_HUMAN	0,95<br>1,02	0,85<br>1,13	1,05				0,98	**2,29**	**5,27**
Sphingosine-1-phosphate phosphatase 1	Q9BX95|SGPP1_HUMAN	0,00	0,00	0,00						
Spleen tyrosine kinase	Q5T6N7|Q5T6N7_HUMAN	**1,27**	**2,03**	3,40	0,92	**0,79**	0,43	0,46	0,77	0,71
Spliceosome RNA helicase BAT1	Q13838|UAP56_HUMAN				1,11	**1,82**	1,48	**1,19**<br>**0,38**	**0,26**<br>**0,28**	**0,23**<br>**0,22**
Splicing factor 1	Q15637|SF01_HUMAN				1,20	1,50	1,04	1,22<br>**1,45**	**0,29**<br>0,83	**0,20**<br>**0,70**
Splicing factor 3 subunit 1	Q15459|SF3A1_HUMAN							**0,84**<br>0,97	**0,32**<br>**0,47**	**0,19**<br>**0,37**
Splicing factor 3A subunit 2	Q15428|SF3A2_HUMAN							0,91<br>1,70	**0,34**<br>**0,58**	**0,19**<br>**0,46**
Splicing factor 3B subunit 1	O75533|SF3B1_HUMAN							0,97<br>**0,51**	**0,48**<br>**0,50**	**0,41**<br>**0,34**
Splicing factor 3B subunit 5	Q9BWJ5|SF3B5_HUMAN							1,01<br>1,08	**0,46**<br>**0,51**	**0,28**<br>**0,49**
Splicing factor 3b, subunit 4, 49kDa	Q5SZ63|Q5SZ63_HUMAN							0,84	0,54	0,60
Splicing factor 3b, subunit 4 variant (Fragment)	Q53FG6|Q53FG6_HUMAN							0,00	0,00	0,00
Splicing factor 45	Q96I25|SPF45_HUMAN							0,87<br>**1,40**	**0,21**<br>0,83	**0,12**<br>0,84
Splicing factor proline/glutamine-rich	Q5SZ71|Q5SZ71_HUMAN	0,54	0,73	2,18	**1,37**	2,77	2,41	0,97<br>**1,17**	**0,28**<br>**0,39**	**0,17**<br>**0,27**
Splicing factor proline/glutamine-rich	Q86VG2|Q86VG2_HUMAN				0,98	**1,78**	1,25			
Splicing factor U2AF 35 kDa subunit	Q01081|U2AF1_HUMAN							**0,83**<br>**0,74**	**0,24**<br>**0,29**	**0,17**<br>**0,26**
Splicing factor, arginine/serine-rich 1	Q07955|SFRS1_HUMAN							0,99<br>1,07	**0,34**<br>**0,51**	**0,21**<br>**0,46**
Splicing factor, arginine/serine-rich 10 (Transformer 2 homolog, Drosophila) variant (Fragment)	Q59GA1|Q59GA1_HUMAN							**0,64**<br>1,05	**0,20**<br>**0,34**	**0,12**<br>**0,29**
Splicing factor, arginine/serine-rich 11	Q8IWE6|Q8IWE6_HUMAN							0,97<br>0,99	**0,41**<br>0,76	**0,45**<br>0,75
Splicing factor, arginine/serine-rich 12	Q8WXA9|SFR12_HUMAN							0,97<br>**1,21**	**0,30**<br>**0,49**	**0,24**<br>**0,35**
Splicing factor, arginine/serine-rich 16	Q8N2M8|SFR16_HUMAN									
Splicing factor, arginine/serine-rich 18	Q8TF01|SFR18_HUMAN							0,96<br>0,00	**0,37**<br>0,00	**0,25**<br>0,00
Splicing factor, arginine/serine-rich 2 variant (Fragment)	Q53FN0|Q53FN0_HUMAN				1,30<br>1,27	**1,83**<br>**1,30**	1,28<br>0,97	**1,21**<br>**1,55**	**0,60**<br>**1,24**	**0,27**<br>**0,87**
Splicing factor, arginine/serine-rich 3	P84103|SFRS3_HUMAN	0,78<br>0,90	1,22<br>**1,55**	**2,19**				1,00<br>**1,06**	**0,48**<br>**0,50**	**0,28**<br>**0,41**
Splicing factor, arginine/serine-rich 4	Q08170|SFRS4_HUMAN	0,81	1,24	**2,27**	1,62	1,74	1,41	1,05	**0,51**	**0,26**
Splicing factor, arginine/serine-rich 5	Q13243|SFRS5_HUMAN							**1,29**<br>**1,64**	**0,56**<br>0,71	**0,41**<br>0,74
Splicing factor, arginine/serine-rich 6	Q13247|SFRS6_HUMAN							0,96	**0,38**	**0,23**
Splicing factor, arginine/serine-rich 9	Q13242|SFRS9_HUMAN							**0,77**<br>0,89	**0,21**<br>**0,31**	**0,15**<br>**0,27**
SPRY domain-containing protein 4	Q8WW59|SPRY4_HUMAN	1,15<br>1,12	1,28<br>1,26	1,21						
SPTLC1 protein	Q6NUL7|Q6NUL7_HUMAN	0,00	0,00	0,00						
SRA stem-loop-interacting RNA-binding protein, mitochondrial precursor	Q9GZT3|SLIRP_HUMAN	**0,86**<br>0,94	**0,74**<br>1,01	**0,58**	1,53	0,88	0,75	1,90	**7,22**	**7,63**

Src homology 3 domain-containing protein HIP-55	A4D2I9|A4D2I9_HUMAN				**0,81**<br>0,00	**0,83**<br>0,00	**0,79**<br>0,00			
Src kinase-associated phosphoprotein 2	O75563|SKAP2_HUMAN							1,04	0,80	0,21
Signal recognition particle receptor subunit alpha	P08240|SRPR_HUMAN	0,92<br>1,01	**1,42**<br>**1,29**	**1,84**				1,41<br>0,71	1,58<br>0,88	1,65<br>1,47
Staphylococcal nuclease domain-containing protein 1	Q7KZF4|SND1_HUMAN	0,94<br>**1,65**	1,02<br>**1,24**	**2,02**	1,28<br>1,81	1,16<br>**1,31**	1,03<br>**1,28**			
StAR-related lipid transfer protein 3	Q14849|STAR3_HUMAN	1,05	1,23	0,95						
Stathmin	P16949|STMN1_HUMAN				0,95<br>0,58	0,86<br>0,65	**0,64**<br>0,52			
Steroid receptor RNA activator 1	Q9HD15|SRA1_HUMAN				0,00	0,00	0,00			
Sterol carrier protein 2	Q5VVZ1|Q5VVZ1_HUMAN	1,03<br>1,03	0,89<br>1,01	**0,80**	**1,49**<br>**1,40**	1,15<br>1,06	**1,61**<br>1,19	1,16	**3,27**	**3,88**
Sterol O-acyltransferase 1	P35610|SOAT1_HUMAN	0,88<br>0,91	0,93<br>1,12	1,00	 2,56	 **0,77**	 1,01	1,77<br>**0,38**	4,49<br>**2,10**	9,37<br>**3,27**
Sterol-4-alpha-carboxylate 3-dehydrogenase, decarboxylating	Q15738|NSDHL_HUMAN	0,99<br>0,99	1,08<br>**1,22**	1,21						
Steryl-sulfatase precursor	P08842|STS_HUMAN	0,00	0,00	0,00						
Stomatin-like protein 2	Q9UJZ1|STML2_HUMAN	0,84<br>1,10	0,88<br>0,95	**0,75**	 1,43	 0,94	 0,90	1,53	**4,42**	**5,08**
Stress-induced-phosphoprotein 1	Q5TZU9|Q5TZU9_HUMAN				**0,80**<br>0,62	**0,93**<br>0,82	0,97<br>**0,89**	**1,89**	**2,55**	**3,01**
Striatin	O43815|STRN_HUMAN	0,90	0,58	0,82						
Stromal cell-derived factor 2 precursor	Q99470|SDF2_HUMAN	0,92<br>**0,79**	**1,17**<br>0,90	1,06				0,86	**1,95**	**2,30**
Stromal interaction molecule 1	Q8N382|Q8N382_HUMAN	0,97<br>**1,19**	1,14<br>**1,29**	1,29						
Structural maintenance of chromosomes protein 1A	Q14683|SMC1A_HUMAN							1,08<br>**0,57**	1,04<br>0,89	0,83<br>1,16
Structural maintenance of chromosomes protein 3	Q9UQE7|SMC3_HUMAN							0,68<br>**0,43**	0,55<br>**0,40**	0,60<br>**0,42**
STXBP2 protein	Q9BU65|Q9BU65_HUMAN				0,00	0,00	0,00			
Succinate dehydrogenase [ubiquinone] flavoprotein subunit, mitochondrial precursor	P31040|DHSA_HUMAN	 1,00	 0,99							
Succinate dehydrogenase [ubiquinone] iron-sulfur subunit, mitochondrial precursor	P21912|DHSB_HUMAN	1,01<br>1,04	1,03<br>0,96	**0,88**	 1,74	 1,02	 1,17			
Succinate dehydrogenase complex, subunit A, flavoprotein variant (Fragment)	Q59GW8|Q59GW8_HUMAN	0,97	0,96	**0,84**						
Succinate dehydrogenase cytochrome b560 subunit, mitochondrial precursor	Q99643|C560_HUMAN	 1,19	 1,37							
Succinic semialdehyde dehydrogenase precursor	Q546H9|Q546H9_HUMAN	**0,69**<br>0,95	0,82<br>**0,90**	0,58						
Succinyl-CoA ligase [ADP-forming] beta-chain, mitochondrial precursor	Q9P2R7|SUCB1_HUMAN	**0,86**<br>**1,12**	0,93<br>0,98	**0,81**						
Succinyl-CoA ligase [GDP-forming] beta-chain, mitochondrial precursor	Q96I99|SUCB2_HUMAN	**0,92**<br>**1,26**	0,95<br>**1,07**	**0,70**	 2,01	 **1,42**	 **1,88**			
Succinyl-CoA:3-ketoacid-coenzyme A transferase 1, mitochondrial precursor	P55809|SCOT_HUMAN	0,97<br>1,06	0,99<br>**1,08**	0,89						
SUCLG1 protein	Q6IAL5|Q6IAL5_HUMAN	1,01<br>1,06	0,89<br>1,06	**0,79**	1,77<br>1,99	**1,25**<br>1,35	2,09<br>1,55			
Sugar phosphate exchanger 2	Q8TED4|SPX2_HUMAN	**0,63**<br>0,89	**0,69**<br>**1,23**	0,86						
Sulfatase-modifying factor 1 precursor	Q8NBK3|SUMF1_HUMAN	0,00<br>0,99	0,00<br>1,07	0,00						
Sulfatase-modifying factor 2 precursor	Q8NBJ7|SUMF2_HUMAN	0,97<br>0,82	1,06<br>1,01	1,04						
Sulfide:quinone oxidoreductase, mitochondrial precursor	Q9Y6N5|SQRD_HUMAN	1,07<br>**0,94**	1,00<br>0,99	**0,88**	**1,95**<br>**1,80**	1,13<br>1,12	1,34<br>**1,27**	1,04<br>**0,47**	**2,23**<br>**2,43**	**4,75**<br>**3,82**
Sulfotransferase family, cytosolic, 1A, phenol-preferring, member 2	Q14CJ7|Q14CJ7_HUMAN				 **0,71**	 0,78	 0,68			
SUMO-activating enzyme subunit 1	Q9UBE0|SAE1_HUMAN				1,01<br>0,00	1,12<br>0,00	1,10<br>0,00			
SUMO-activating enzyme subunit 2	Q9UBT2|SAE2_HUMAN				0,94<br>0,00	0,87<br>0,00	0,88<br>0,00			
Superoxide dismutase [Cu-Zn]	P00441|SODC_HUMAN	 0,91	 1,14		0,83<br>0,67	1,03<br>0,98	1,02<br>0,99			
Superoxide dismutase [Mn], mitochondrial precursor	P04179|SODM_HUMAN	**0,90**<br>**1,07**	0,95<br>**1,20**	**0,63**	**2,00**<br>**1,83**	**1,81**<br>**1,64**	**3,16**<br>**2,19**			
Surfeit 1	Q5T8T3|Q5T8T3_HUMAN	1,16<br>0,98	0,95<br>**0,84**	**0,83**						
Surfeit 4	Q5T8U6|Q5T8U6_HUMAN	0,95<br>1,03	**1,59**<br>1,27	**1,81**						
Surfeit 5 (Fragment)	Q5T8T8|Q5T8T8_HUMAN							0,74	0,35	0,14
Survival of motor neuron-related-splicing factor 30	O75940|SPF30_HUMAN							0,77<br>0,81	0,51<br>1,03	**0,27**<br>0,70
SWI/SNF-related matrix-associated actin-dependent regulator of chromatin c2 isoform b variant (Fragment)	Q59GV3|Q59GV3_HUMAN							**0,77**	**0,17**	**0,14**
SWI/SNF-related matrix-associated actin-dependent regulator of chromatin subfamily A member 5	O60264|SMCA5_HUMAN							 **0,23**	 **0,35**	 **0,31**
SWI/SNF-related matrix-associated actin-dependent regulator of chromatin subfamily C member 2	Q8TAQ2|SMRC2_HUMAN							 **0,78**	 **0,30**	 **0,30**
SWI/SNF-related matrix-associated actin-dependent regulator of chromatin subfamily E member 1	Q969G3|SMCE1_HUMAN							0,91<br>1,08	**0,19**<br>**0,26**	**0,15**<br>**0,26**
Synaptic glycoprotein SC2	Q9NZ01|GPSN2_HUMAN	0,96<br>0,89	1,13<br>1,06	1,06				1,21<br>0,69	2,09<br>**1,83**	**3,29**<br>**2,36**
Synaptic vesicle membrane protein VAT-1 homolog	Q99536|VAT1_HUMAN	0,85<br>1,02	1,30<br>1,05	1,23	 1,01	 1,12	 1,16			
Synaptogyrin-1	O43759|SNG1_HUMAN	1,04<br>0,71	0,81<br>0,44	**0,52**						
Synaptogyrin-2	O43760|SNG2_HUMAN	1,09<br>0,85	0,97<br>**0,78**	0,92						
Synaptophysin-like protein 1	Q16563|SYPL1_HUMAN	1,14<br>0,91	1,01<br>1,00	1,10						
Synaptosomal-associated protein 23	O00161|SNP23_HUMAN	1,26<br>1,01	0,95<br>1,04	0,98	1,65	0,94	1,42	**0,77**<br>0,66	**0,17**<br>0,85	**0,14**<br>0,74
Synaptosomal-associated protein 29	O95721|SNP29_HUMAN	 1,25	 0,90							
SYNGR2 protein	Q3KQZ2|Q3KQZ2_HUMAN				 2,58	 1,28	 2,77			
Syntaxin 3A	Q53YE2|Q53YE2_HUMAN	0,00<br>1,04	0,00<br>1,04	0,00						
Syntaxin binding protein 2 variant (Fragment)	Q53GF4|Q53GF4_HUMAN	 **1,26**	 1,06		0,90	0,97	0,94			
Syntaxin-12	Q86Y82|STX12_HUMAN	0,85<br>0,99	**0,69**<br>1,03	**0,65**	 1,68	 1,86	 1,48			
Syntaxin-5	Q13190|STX5_HUMAN							0,72	1,12	0,95
Syntaxin-7	O15400|STX7_HUMAN	1,00<br>1,09	0,67<br>1,00	**0,38**	 **1,55**	 1,18	 1,06			
Syntaxin-8	Q9UNK0|STX8_HUMAN	1,03<br>0,98	**1,17**<br>1,10	1,34						
Syntaxin-binding protein 2	Q15833|STXB2_HUMAN	1,24	1,04	1,22						

					1,04	0,98	0,90			
Syntaxin-binding protein 3	O00186|STXB3_HUMAN	1,15<br>1,27	0,87<br>1,18	1,09						
Syntenin-1	O00560|SDCB1_HUMAN	0,84	**0,81**	**0,80**	0,85	**0,42**	**0,33**			
SYT protein (Fragment)	Q8TDQ9|Q8TDQ9_HUMAN							0,77	0,26	0,14
TAGLN2 protein	Q6FGI1|Q6FGI1_HUMAN				**0,85**<br>0,58	0,93<br>0,86	**0,84**<br>0,68			
Talin-1	Q9Y490|TLN1_HUMAN	**1,39**<br>1,18	**1,24**<br>**1,17**	**2,44**	**0,90**<br>**1,05**	**1,08**<br>1,12	1,03<br>**1,16**	0,85<br>0,73	**1,44**<br>1,19	**2,10**<br>0,94
TAP2 protein (Fragment)	Q9UQ60|Q9UQ60_HUMAN				1,08	0,73	1,10			
TAP binding protein	A2ABC0|A2ABC0_HUMAN	0,92<br>0,94	0,97<br>1,05	1,06						
Tapasin precursor	O15533|TPSN_HUMAN				**2,13**	1,02	1,01	**0,81**	21,82<br>4,99	31,45<br>7,13
Tapasin-related protein precursor	Q9BX59|TPSNR_HUMAN	0,00<br>0,84	0,00<br>1,25	0,00						
TAR DNA binding protein variant (Fragment)	Q53H27|Q53H27_HUMAN							**0,44**	**0,33**	**0,29**
Target of myb1	Q86X74|Q86X74_HUMAN				0,92	0,99	**0,71**			
Target of Myb protein 1	O60784|TOM1_HUMAN				0,84	0,92	0,87			
Tartrate-resistant acid phosphatase type 5 precursor	P13686|PPA5_HUMAN	1,05<br>0,96	0,82<br>**0,76**	0,75	**1,66**	**0,56**	**0,81**	**0,81**<br>**0,73**	**1,22**<br>0,77	1,14<br>0,91
TATA-binding protein-associated factor 2N	Q92804|RBP56_HUMAN				1,17	2,20	**1,86**	1,03<br>**1,26**	**0,19**<br>**0,31**	**0,10**<br>**0,27**
TBC1 domain family member 15	Q8TC07|TBC15_HUMAN	0,00	0,00	0,00						
TBCA protein	Q6FGD7|Q6FGD7_HUMAN				**0,76**<br>0,56	**0,86**<br>**0,73**	**0,75**<br>0,60			
T-cell surface glycoprotein CD1b precursor	P29016|CD1B_HUMAN	0,00	0,00	0,00						
T-cell, immune regulator 1, ATPase, H+ transporting, lysosomal V0 subunit A3	Q8WVC5|Q8WVC5_HUMAN	1,09<br>**0,91**	1,13<br>0,95	**1,18**				1,43<br>0,67	**6,03**<br>**1,74**	**7,10**<br>**2,85**
T-complex protein 1 subunit alpha	P17987|TCPA_HUMAN				**0,80**<br>0,82	1,19<br>1,01	**1,21**<br>0,90			
T-complex protein 1 subunit beta	P78371|TCPB_HUMAN	1,17<br>1,16	0,94<br>1,16	1,39	0,86<br>**0,67**	1,05<br>0,98	1,12<br>0,91			
T-complex protein 1 subunit delta	P50991|TCPD_HUMAN				**0,74**<br>0,90	1,01<br>1,04	1,06<br>1,20			
T-complex protein 1 subunit eta	Q99832|TCPH_HUMAN				**0,75**<br>0,94	1,13<br>**1,27**	**1,16**<br>**1,22**			
T-complex protein 1 subunit theta	P50990|TCPQ_HUMAN	**1,66**<br>1,08	**2,30**<br>1,17	**5,72**	**0,74**<br>**0,86**	1,07<br>1,11	1,02<br>**1,23**			
T-complex protein 1 subunit zeta	P40227|TCPZ_HUMAN				**0,79**	1,05	1,09			
Telomeric repeat-binding factor 2	Q15554|TERF2_HUMAN							**0,88**<br>0,00	0,37<br>0,00	**0,25**<br>0,00
Testin	Q9UGI8|TES_HUMAN				0,96<br>0,94	0,94<br>0,95	1,00<br>1,24			
Testis-expressed sequence 264 protein precursor	Q9Y6I9|TX264_HUMAN	0,90<br>0,85	1,06<br>1,20	1,02	1,93	1,00	1,06			
Tetratricopeptide repeat domain 19	Q2M248|Q2M248_HUMAN	0,87	0,83							
Tetratricopeptide repeat protein 35	Q15006|TTC35_HUMAN	1,00<br>0,93	1,22<br>1,05	1,13				0,90	**2,43**	**2,56**
TFAM protein (Fragment)	Q6LES8|Q6LES8_HUMAN	0,90<br>**1,12**	0,90<br>1,01	**0,84**				**0,66**<br>**1,16**	1,05<br>**1,87**	**1,14**<br>**2,77**
Thioesterase superfamily member 2	Q9NPJ3|THEM2_HUMAN	0,82<br>0,91	0,82<br>**0,84**	**0,51**	0,00	0,00	0,00	0,94<br>0,62	**1,41**<br>1,24	**1,49**<br>1,56
Thiopurine S-methyltransferase	P51580|TPMT_HUMAN				1,00<br>0,89	0,95<br>0,84	0,97<br>0,65			
Thioredoxin delta 3 (Fragment)	O60744|O60744_HUMAN				**0,72**	0,90	**0,85**			
Thioredoxin domain-containing protein 1 precursor	Q9H3N1|TXND1_HUMAN	1,15<br>0,96	1,12<br>1,05	**1,21**	**1,56**	0,95	0,94	0,37	**10,29**<br>2,99	**19,90**<br>7,34
Thioredoxin domain-containing protein 12 precursor	O95881|TXD12_HUMAN	0,00<br>0,97	0,00<br>**1,21**	0,00						
Thioredoxin domain-containing protein 13 precursor	Q9H1E5|TXD13_HUMAN	0,87<br>0,97	1,04<br>0,93	1,08						
Thioredoxin domain-containing protein 14 precursor	Q9Y320|TXD14_HUMAN	0,90<br>1,04	1,08<br>1,69	1,10						
Thioredoxin domain-containing protein 17	Q9BRA2|TXD17_HUMAN				0,52	0,76	0,46			
Thioredoxin domain-containing protein 4 precursor	Q9BS26|TXND4_HUMAN	1,15<br>0,95	1,05<br>**0,93**	1,06	**1,75**<br>1,67	0,96<br>1,18	**1,87**<br>**1,52**	1,03<br>1,08	**2,65**<br>**2,37**	**5,75**<br>**4,30**
Thioredoxin domain-containing protein 5 precursor	Q8NBS9|TXND5_HUMAN	0,90<br>0,95	1,02<br>1,06	0,93				**2,35**	**2,72**	**16,79**
Thioredoxin reductase 2 isoform 1 variant (Fragment)	Q59ET7|Q59ET7_HUMAN	0,00	0,00							
Thioredoxin reductase 2, mitochondrial precursor	Q9NNW7|TRXR2_HUMAN	1,04<br>0,94	0,86<br>1,13	0,63						
Thioredoxin, mitochondrial precursor	Q99757|THIOM_HUMAN	**0,86**<br>0,99	**0,77**<br>1,00	**0,58**	2,00<br>1,08	2,08<br>0,90	2,27<br>1,15			
Thioredoxin-dependent peroxide reductase, mitochondrial precursor	P30048|PRDX3_HUMAN	**0,90**<br>**1,14**	**0,87**<br>0,98	**0,74**	**1,80**<br>1,92	**1,42**<br>**1,38**	**2,35**<br>1,96			
Thioredoxin-like 1 variant (Fragment)	Q59G46|Q59G46_HUMAN				0,00<br>0,71	0,00<br>1,07	0,00<br>0,45			
Thioredoxin-like 3	Q5JV01|Q5JV01_HUMAN				1,09	1,12	0,66			
THO complex 2	Q5JZ12|Q5JZ12_HUMAN							0,78<br>**0,60**	0,30<br>0,62	**0,19**<br>0,49
THO complex subunit 1	Q96FV9|THOC1_HUMAN							0,47	0,72	0,69
THO complex subunit 4	Q86V81|THOC4_HUMAN	0,85<br>**0,88**	0,88<br>0,99	0,78	1,08	1,22	0,99	1,05<br>**1,43**	**0,35**<br>**0,51**	**0,18**<br>**0,47**
THO complex subunit 6 homolog	Q86W42|THOC6_HUMAN							0,00	0,00	0,00
THO complex subunit 7 homolog	Q6I9Y2|THOC7_HUMAN							0,82	**0,40**	**0,30**
THRAP3 protein (Fragment)	Q6PJV4|Q6PJV4_HUMAN							0,00<br>1,04	0,00<br>0,85	0,00<br>0,65
Threonyl-tRNA synthetase variant (Fragment)	Q53GX7|Q53GX7_HUMAN				1,13	0,97	1,14			
Threonyl-tRNA synthetase, mitochondrial precursor	Q9BW92|SYTM_HUMAN	1,02	1,05							
Thromboxane A synthase 1 (Platelet, cytochrome P450, family 5, subfamily A) isoform TXS-I variant (Fragment)	Q53F23|Q53F23_HUMAN	0,90<br>1,04	**1,13**<br>**1,13**	1,05						
Thymidine phosphorylase precursor	P19971|TYPH_HUMAN				1,02<br>**0,84**	1,02<br>0,96	1,11<br>0,94			
Thymocyte nuclear protein 1	Q9P016|THYN1_HUMAN							1,04<br>0,94	**0,45**<br>0,59	**0,38**<br>**0,55**
Thymosin-like 4	Q5T4B6|Q5T4B6_HUMAN	**2,37**	**2,15**					0,97	**0,42**	**0,24**
Thyroid hormone receptor-associated protein 3	Q9Y2W1|TR150_HUMAN							0,98<br>**1,12**	**0,65**<br>1,20	**0,32**<br>**0,74**

TIM21-like protein, mitochondrial precursor	Q9BVV7|TI21L_HUMAN	1,11	1,15	0,95						
		0,97	1,05							
Tissue alpha-L-fucosidase precursor	P04066|FUCO_HUMAN									
		1,09	0,90							
Titin	Q8WZ42|TITIN_HUMAN	**0,86**	0,99	**1,19**	**1,50**	**1,19**	**1,62**	1,07	1,07	0,91
		0,96	1,04		1,07	0,91	0,92	1,12	1,16	1,40
TMED5 protein	Q49AG2|Q49AG2_HUMAN							1,13	**2,88**	**4,50**
TMSB4X protein (Fragment)	Q0P5Q0|Q0P5Q0_HUMAN	1,10	1,23	1,30	**0,60**	**0,75**	**0,64**			
					0,49	0,58	0,48			
TNF receptor-associated protein 1 variant (Fragment)	Q59EK6|Q59EK6_HUMAN									
					0,00	0,00	0,00			
Toll-like receptor 2 precursor	O60603|TLR2_HUMAN									
		1,11	0,97							
Toll-like receptor 8 precursor	Q9NR97|TLR8_HUMAN	1,07	0,93	0,98						
		0,98	**0,80**							
Torsin family protein C9orf167	Q9NXH8|CI167_HUMAN	0,88	0,84	0,79						
		1,00	0,95							
Torsin-1A precursor	O14656|TOR1A_HUMAN									
		1,02	1,27							
Torsin-1A-interacting protein 1	Q5JTV8|TOIP1_HUMAN	0,94	1,19	**1,37**						
		0,94	1,07		0,00	0,00	0,00	**1,28**	1,08	1,11
Torsin-1A-interacting protein 2	Q8NFQ8|TOIP2_HUMAN	0,91	0,91	0,74						
		1,07	**1,28**							
Torsin-1B precursor	O14657|TOR1B_HUMAN									
		0,00	0,00							
TPD52L2 protein	Q6FGS1|Q6FGS1_HUMAN	0,63	0,75	0,54						
					0,62	0,80	0,46			
TP53-regulated inhibitor of apoptosis 1	O43715|TRIA1_HUMAN									
		1,18	0,94							
TPMsk3 (Fragment)	Q8TCG3|Q8TCG3_HUMAN	**2,33**	**3,84**	**10,36**						
TraB domain-containing protein	Q9H4I3|TRABD_HUMAN	1,04	0,98	0,90						
		1,00	1,16							
TRAM1 protein	Q6FHL3|Q6FHL3_HUMAN			1,19						
TRAM1 protein (Fragment)	Q6FHU7|Q6FHU7_HUMAN							0,00	0,00	0,00
								0,00	0,00	0,00
Trans-2-enoyl-CoA reductase, mitochondrial precursor	Q9BV79|MECR_HUMAN	0,81	0,93	0,90						
		0,91	0,95							
Transaldolase	P37837|TALDO_HUMAN				**0,84**	1,01	**0,94**			
					**0,87**	**1,14**	0,89			
Transcription elongation factor B polypeptide 1	Q15369|ELOC_HUMAN				0,95	1,08	0,81	0,94	0,51	0,25
					0,00	0,00	0,00			
Transcription elongation factor B polypeptide 2	Q15370|ELOB_HUMAN				0,89	0,87	0,87	1,01	0,85	0,44
					0,80	0,83	**0,72**			
Transcription elongation factor B polypeptide 3	Q14241|ELOA1_HUMAN							1,23	0,54	0,33
								1,81	1,23	0,66
Transcription elongation factor SPT5	O00267|SPT5H_HUMAN									
								**0,45**	0,67	0,53
Transcription elongation regulator 1	O14776|TCRG1_HUMAN							**0,77**	**0,33**	**0,28**
								0,84	**0,44**	**0,42**
Transcription factor BTF3	P20290|BTF3_HUMAN				0,84	0,82	0,89	0,64	1,66	1,29
								1,17	1,95	2,01
Transcription factor BTF3 homolog 4	Q96K17|BT3L4_HUMAN				1,00	**0,73**	**0,78**	0,95	**2,18**	**2,37**
Transcription factor MafF	Q9ULX9|MAFF_HUMAN							0,90	0,08	0,04
Transcription factor PU,1	P17947|SPI1_HUMAN							**0,63**	**0,19**	**0,20**
								**0,84**	**0,46**	**0,40**
Transcription initiation factor TFIID subunit 9	Q16594|TAF9_HUMAN							0,00	0,00	0,00
								0,00	0,00	0,00
Transcription intermediary factor 1-beta	Q13263|TIF1B_HUMAN							0,74	0,36	0,40
					1,46	1,79	1,20	**0,56**	0,43	**0,26**
Transcriptional regulator	Q6IEH8|Q6IEH8_HUMAN									
								1,01	0,69	0,62
Transcriptional repressor p66 alpha	Q86YP4|P66A_HUMAN							**0,74**	**0,16**	**0,12**
								1,08	**0,50**	**0,39**
Transcriptional repressor p66 beta	Q8WXI9|P66B_HUMAN							1,07	0,34	0,26
								1,09	0,35	0,20
Transcriptional repressor protein YY1	P25490|TYY1_HUMAN							0,81	**0,29**	**0,23**
								0,82	0,33	0,36
Transducin beta-like 2 protein	Q9Y4P3|TBL2_HUMAN	0,90	0,88	0,83						
		**0,91**	1,07							
Transferrin	Q06AH7|Q06AH7_HUMAN									
		1,04	1,06							
Transferrin receptor	Q1HE24|Q1HE24_HUMAN	0,94	**0,48**	**0,54**						
					**1,88**	1,01	**1,35**			
Transferrin variant (Fragment)	Q53H26|Q53H26_HUMAN	1,19	**0,81**	0,87	**1,67**	1,00	**1,34**			
					**1,58**	1,08	1,23	0,00	0,00	0,00
Transforming growth factor beta-1 precursor	P01137|TGFB1_HUMAN									
		0,90	1,00							
Transglutaminase 2	Q6B838|Q6B838_HUMAN	**2,11**	**3,79**	**9,17**	0,95	1,09	**0,80**			
Trans-Golgi network integral membrane protein 2 precursor	O43493|TGON2_HUMAN	1,23	0,85	0,79						
		1,15	0,92		1,64	0,70	0,62			
Transient receptor potential cation channel subfamily V member 2	Q9Y5S1|TRPV2_HUMAN	1,20	0,87	0,94						
		1,07	1,00							
Transketolase variant (Fragment)	Q53EM5|Q53EM5_HUMAN				**0,91**	**1,10**	**0,93**			
		**1,36**	1,60		1,07	1,26	1,06			
Translation initiation factor eIF-2B subunit alpha	Q14232|EI2BA_HUMAN				1,05	0,71	0,98			
					0,00	0,00	0,00			
Translation initiation factor IF-2, mitochondrial precursor	P46199|IF2M_HUMAN									
		1,11	**1,25**							
Translocase of inner mitochondrial membrane 44 homolog (Fragment)	Q53G69|Q53G69_HUMAN	1,03	1,05	0,83						
		1,01	0,94							
Translocase of inner mitochondrial membrane 50 homolog	Q0VAB1|Q0VAB1_HUMAN	**0,82**	**0,82**	**0,70**						
		1,02	1,01							
	Q6P0M2|Q6P0M2_HUMAN							0,79	0,90	1,51
Translocase of outer mitochondrial membrane 70 homolog A								**0,61**	1,19	**1,48**
Translocated promoter region	Q5SWY0|Q5SWY0_HUMAN							**0,69**	**0,35**	**0,24**
								**0,63**	**0,49**	**0,39**
Translocation protein SEC62	Q99442|SEC62_HUMAN	1,02	1,14	1,14	2,19	0,93	1,18	1,17	**2,98**	**5,94**
		1,05	**1,21**					1,11	**3,69**	**7,02**
Translocation protein SEC63 homolog	Q9UGP8|SEC63_HUMAN	0,91	1,04	1,05						
		1,01	1,08					0,36	3,01	4,91
Translocon-associated protein subunit alpha precursor	P43307|SSRA_HUMAN	0,97	1,09	1,03				0,88	**2,55**	**3,07**
		1,01	**1,18**		1,39	1,01	0,70			
Translocon-associated protein subunit delta precursor	P51571|SSRD_HUMAN	1,28	1,29	1,18				1,29	**2,65**	**3,30**
		1,13	1,14					0,00	0,00	0,00
Translocon-associated protein subunit gamma	Q9UNL2|SSRG_HUMAN	0,93	0,95	0,82						
		0,79	1,08					1,07	**1,61**	**2,36**
Transmembrane 7 superfamily member 3 precursor	Q9NS93|TM7S3_HUMAN									
		1,12	0,89							
Transmembrane 9 superfamily member 1 precursor	O15321|TM9S1_HUMAN	0,00	0,00	0,00						
Transmembrane 9 superfamily member 2 precursor	Q99805|TM9S2_HUMAN	1,25	0,75	0,85						
		0,97	**0,80**							
Transmembrane 9 superfamily member 3 precursor	Q9HD45|TM9S3_HUMAN	1,17	**0,62**	**0,69**						

		1,05	0,90							
Transmembrane 9 superfamily member 4 precursor	Q92544|TM9S4_HUMAN	0,99	**0,56**	**0,59**						
		1,04	0,99							
Transmembrane and coiled-coil domain-containing protein 1	Q9UM00|TMCO1_HUMAN	**1,21**	1,19	**1,32**	1,73	1,09	0,66	0,97	**1,78**	**2,12**
		1,00	**1,35**					1,05	1,81	1,92
Transmembrane and coiled-coil domain-containing protein 3 precursor	Q6UWJ1|TMCO3_HUMAN	1,09	1,16	1,20						
		1,16	0,95							
Transmembrane emp24 domain-containing protein 1 precursor	Q13445|TMED1_HUMAN	1,12	1,06	**1,24**				1,73	**7,69**	**10,93**
		**0,70**	0,93							
Transmembrane emp24 domain-containing protein 10 precursor	P49755|TMEDA_HUMAN									
		1,00	1,06							
Transmembrane emp24 domain-containing protein 3 precursor	Q9Y3Q3|TMED3_HUMAN	0,00	0,00	0,00						
		0,92	0,94							
Transmembrane emp24 domain-containing protein 4 precursor	Q7Z7H5|TMED4_HUMAN	1,27	0,88	1,09				1,04	**4,76**	**6,25**
		0,98	0,93							
Transmembrane emp24 domain-containing protein 5 precursor	Q9Y3A6|TMED5_HUMAN	1,25	1,09	1,18						
		**0,85**	0,93							
Transmembrane emp24 domain-containing protein 7 precursor	Q9Y3B3|TMED7_HUMAN	1,07	0,79	0,82				0,63	**4,94**	2,25
		0,96	**0,76**							
Transmembrane emp24 domain-containing protein 9 precursor	Q9BVK6|TMED9_HUMAN	1,13	1,04	**1,36**				1,06	**2,96**	**4,28**
		0,98	**1,18**					**1,57**	**3,57**	**6,49**
Transmembrane glycoprotein NMB precursor	Q14956|GPNMB_HUMAN				**1,54**	**0,76**	1,08			
		1,03	0,95		**1,77**	**0,60**	1,03	**1,58**	**2,27**	**3,31**
Transmembrane protein 109 precursor	Q9BVC6|TM109_HUMAN	0,81	1,04	0,96	1,89	0,65	0,79	1,04	**3,71**	**4,13**
		0,95	**1,16**		**1,90**	0,84	0,89	1,01	2,16	2,72
Transmembrane protein 11	P17152|TMM11_HUMAN	0,87	0,92	0,93						
		0,92	0,99							
Transmembrane protein 111	Q9P0I2|TM111_HUMAN	0,77	0,76	0,71						
		0,78	0,85							
Transmembrane protein 126A	Q9H061|T126A_HUMAN	1,13	1,13	0,81						
		1,05	0,93							
Transmembrane protein 126B	Q8IUX1|T126B_HUMAN	0,00	0,00	0,00						
Transmembrane protein 160	Q9NX00|TM160_HUMAN	0,37	0,75	0,70				0,00	0,00	0,00
		0,83	0,55							
Transmembrane protein 165	Q9HC07|TM165_HUMAN	3,46	0,91	1,66						
Transmembrane protein 16F	Q4KMQ2|TM16F_HUMAN	1,02	**0,87**	1,05						
		0,95	1,06							
Transmembrane protein 16K	Q9NW15|TM16K_HUMAN	**1,19**	1,00	1,04						
Transmembrane protein 173	Q86WV6|TM173_HUMAN									
		0,85	0,66							
Transmembrane protein 19	Q96HH6|TMM19_HUMAN									
		1,07	1,14							
Transmembrane protein 192	Q8IY95|TM192_HUMAN	0,00	0,00	0,00						
		1,17	1,25							
Transmembrane protein 205	Q6UW68|TM205_HUMAN	0,98	1,21	1,19				0,00	0,00	0,00
		1,02	0,92					0,00	0,00	0,00
Transmembrane protein 32 precursor	Q8N4V1|TMM32_HUMAN							0,87	**2,89**	**2,57**
		0,85	0,66							
Transmembrane protein 33	P57088|TMM33_HUMAN	0,94	1,01	1,02						
		**0,87**	0,90		1,78	0,88	0,72			
Transmembrane protein 41A precursor	Q96HV5|TM41A_HUMAN									
		**0,82**	1,10							
Transmembrane protein 43	Q9BTV4|TMM43_HUMAN	**0,81**	1,02	1,03				1,72	4,99	5,71
		0,97	**1,19**		1,68	1,13	1,24			
Transmembrane protein 63A	O94886|TM63A_HUMAN	1,24	**0,88**	0,67						
		0,91	0,98							
Transmembrane protein 65	Q6PI78|TMM65_HUMAN									
		1,16	0,93							
Transmembrane protein 70	Q9BUB7|TMM70_HUMAN	0,00	0,00	0,00						
		0,52	0,72							
Transmembrane protein 85	Q5J8M3|TMM85_HUMAN	1,75	1,71	0,79					5,32	8,31
		0,97	1,07							
Transmembrane protein 93	Q9BV81|TMM93_HUMAN	1,20	0,91	0,93						
		1,23	1,26							
Transporter 1, ATP-binding cassette, sub-family B	Q96CP4|Q96CP4_HUMAN	1,06	1,76	1,56						
Transporter 1 ATP-binding cassette sub-family B	B1AZV3|B1AZV3_HUMAN									
		0,96	1,31							
Transportin-1	Q92973|TNPO1_HUMAN									
					0,79	0,91	0,59			
TRAP1 protein (Fragment)	Q9BV61|Q9BV61_HUMAN	0,93	**0,89**	**0,64**						
Treacle protein	Q13428|TCOF_HUMAN							1,03	**0,49**	**0,26**
					0,82	4,17	7,96	**1,50**	1,15	1,08
Tricarboxylate transport protein, mitochondrial precursor	P53007|TXTP_HUMAN	1,00	0,92	**0,79**						
Trifunctional enzyme subunit alpha, mitochondrial precursor	P40939|ECHA_HUMAN	**0,92**	**0,88**	**0,75**	**2,08**	**1,51**	1,74	**1,48**	**3,30**	**9,46**
		0,99	0,99		**2,04**	1,16	**1,63**	**0,48**	**2,45**	**4,07**
Trifunctional enzyme subunit beta, mitochondrial precursor	P55084|ECHB_HUMAN	**0,92**	**0,90**	**0,79**	**2,24**	**1,26**	**1,79**	1,10	**2,29**	**3,89**
		**0,94**	**0,94**		**2,35**	**1,27**	**1,47**	**0,74**	**2,32**	**3,87**
Trifunctional purine biosynthetic protein adenosine-3	P22102|PUR2_HUMAN									
					1,09	1,09	1,06			
Trimeric intracellular cation channel type B	Q9NVV0|TM38B_HUMAN	0,00	0,00	0,00						
Triosephosphate isomerase (Fragment)	Q6FHP9|Q6FHP9_HUMAN	2,09	2,87	3,01	**0,87**	0,95	**0,89**			
					0,74	**0,84**	0,71			
Triosephosphate isomerase (Fragment)	Q53HE2|Q53HE2_HUMAN									
		0,84	0,97							
TRIP12 protein	Q14CA3|Q14CA3_HUMAN							1,23	1,11	1,00
								1,27	1,25	1,05
Tripartite motif protein TRIM19 kappa	Q9BZX7|Q9BZX7_HUMAN							0,00	0,00	0,00
								**0,52**	0,73	**0,37**
Tripeptidyl-peptidase 1 precursor	O14773|TPP1_HUMAN	**1,17**	**1,30**	1,10	**2,11**	0,86	1,05			
		0,91	**0,94**		1,78	0,89	0,68	1,61	**1,97**	0,95
Tripeptidyl-peptidase I variant (Fragment)	Q53HP2|Q53HP2_HUMAN							2,09	**2,78**	1,49
tRNA pseudouridine synthase A	Q9Y606|TRUA_HUMAN									
		1,02	1,05							
tRNA-nucleotidyltransferase 1, mitochondrial precursor	Q96Q11|TRNT1_HUMAN									
		0,98	0,97							
Tropomodulin-3	Q9NYL9|TMOD3_HUMAN	1,05	1,16	2,63	**0,73**	0,98	1,04	**1,31**	**2,64**	**2,39**
					**0,65**	**0,83**	**0,70**	1,22	**4,12**	**3,95**
Tropomyosin 3	Q5VU59|Q5VU59_HUMAN				1,01	1,06	1,04			
					0,64	0,72	**0,67**	**1,49**	**4,04**	**4,85**
Tropomyosin alpha-4 chain	P67936|TPM4_HUMAN	1,39	2,02	3,56	0,93	0,98	0,98	**1,93**	**2,96**	**3,33**
		1,71	**1,71**		**0,73**	**0,80**	**0,82**	1,28	**2,71**	**3,36**
Tropomyosin isoform	Q15657|Q15657_HUMAN									
					0,84	0,83	1,12			
Trypsin-1 precursor	P07477|TRY1_HUMAN				1,55	**1,51**	**1,91**			
		1,11	1,24							
TST protein	Q6IB06|Q6IB06_HUMAN	**0,81**	**0,86**	**0,78**						
		1,08	0,95							
TUBB6 protein	Q2NKY5|Q2NKY5_HUMAN				0,00	0,00	0,00			
Tubulin alpha-1A chain	Q71U36|TBA1A_HUMAN	1,34	**4,15**	**8,16**	0,99	0,82	0,47			

Tubulin alpha-1C chain	Q9BQE3|TBA1C_HUMAN	**1,29**	**2,65**		0,89	0,84	0,58	0,67	**3,31**	**3,71**
Tubulin alpha-4A chain	P68366|TBA4A_HUMAN	0,00	0,00	0,00	0,55<br>0,81	0,66<br>0,85	0,50<br>0,91			
Tubulin beta-4 chain	P04350|TBB4_HUMAN	0,99	**1,39**	2,17		0,65	0,28			
Tubulin beta-6 chain	Q9BUF5|TBB6_HUMAN				0,74	0,96	0,54			
Tubulin folding cofactor B	Q99426|TBCB_HUMAN				**0,76**	0,85	0,72			
Tubulin, alpha 2	Q1ZYQ1|Q1ZYQ1_HUMAN							1,54	**2,76**	**4,97**
Tubulin, beta	Q5SU16|Q5SU16_HUMAN	1,23	2,56	**8,01**	**0,67**<br>0,81	**0,68**<br>0,61	**0,58**<br>**0,58**			
Tubulin, beta 2C	Q8N6N5|Q8N6N5_HUMAN				0,66	0,84	0,67	0,73	2,62	4,19
Tubulin--tyrosine ligase-like protein 12	Q14166|TTL12_HUMAN				0,76	0,74	**0,43**			
Tumor necrosis factor receptor superfamily member 5 precursor	P25942|TNR5_HUMAN	**1,40**<br>1,05	1,11<br>1,04	1,01				1,08<br>1,20	0,88<br>1,46	1,03<br>2,29
Tumor necrosis factor, alpha-induced protein 8	O95379|TFIP8_HUMAN				0,83<br>**0,59**	0,82<br>0,79	0,81<br>0,65			
Tumor protein, translationally-controlled 1	Q5W0H4|Q5W0H4_HUMAN				0,94	1,03	0,90			
Tumor rejection antigen (Gp96) 1	Q5CAQ5|Q5CAQ5_HUMAN	**0,91**<br>0,98	1,03<br>**1,14**	1,04	**2,05**<br>2,28	**1,29**<br>1,33	**2,16**<br>1,67	0,89<br>**0,71**	**2,19**<br>**2,18**	**5,78**<br>**3,86**
Twinfilin-2	Q6IBS0|TWF2_HUMAN				**0,75**<br>0,84	0,98<br>0,98	0,95<br>1,09			
TXN protein (Fragment)	Q53X69|Q53X69_HUMAN	1,52<br>0,90	2,31<br>1,51	**3,85**	**0,64**	0,79	0,79	0,90<br>1,44	**4,41**<br>**3,99**	**4,70**<br>**3,81**
Type-1 angiotensin II receptor-associated protein	Q6RW13|ATRAP_HUMAN	1,06<br>0,73	0,91<br>**0,66**	1,26						
TYROBP protein	Q6FGA5|Q6FGA5_HUMAN	1,13	1,10							
Tyrosine-protein kinase HCK	P08631|HCK_HUMAN	1,15	1,65	2,08						
Tyrosine-protein kinase receptor	A6P4V4|A6P4V4_HUMAN				0,83<br>0,96	0,97<br>1,16	0,91<br>**1,41**	0,89	**1,48**	1,39
Tyrosine-protein kinase receptor	Q8TDJ5|Q8TDJ5_HUMAN				0,95<br>0,77	0,89<br>0,96	1,04<br>1,15			
Tyrosine-protein phosphatase non-receptor type 1	P18031|PTN1_HUMAN	0,75	1,22	**2,06**						
Tyrosine-protein phosphatase non-receptor type 12	Q05209|PTN12_HUMAN				0,95	1,13	1,00			
Tyrosine-protein phosphatase non-receptor type 23	Q9H3S7|PTN23_HUMAN				0,00	0,00	0,00			
Tyrosine-protein phosphatase non-receptor type substrate 1 precursor	P78324|SHPS1_HUMAN	1,23<br>1,22	0,64<br>0,79	**0,67**	0,00	0,00	0,00			
Tyrosyl-tRNA synthetase, cytoplasmic	P54577|SYYC_HUMAN	1,14	1,84	**6,24**	**0,80**<br>0,95	**0,80**<br>0,91	**0,62**<br>**0,76**	1,24<br>0,56	**1,71**<br>1,07	**2,19**<br>0,80
Tyrosyl-tRNA synthetase, mitochondrial precursor	Q9Y2Z4|SYYM_HUMAN	0,94<br>1,04	**0,79**<br>1,03	**0,65**						
U1 small nuclear ribonucleoprotein 70 kDa	P08621|RU17_HUMAN							0,92	**0,25**	**0,17**
U1 small nuclear ribonucleoprotein A	P09012|SNRPA_HUMAN				1,11<br>0,73	**2,71**<br>**1,85**	**1,97**<br>1,24	**1,13**<br>**1,52**	**0,46**<br>**0,62**	**0,29**<br>**0,60**
U2 small nuclear ribonucleoprotein B''	P08579|RU2B_HUMAN				0,00	0,00	0,00	1,02<br>**1,26**	**0,49**<br>**0,63**	**0,31**<br>**0,58**
U2 small nuclear RNA auxiliary factor 2	Q96HC5|Q96HC5_HUMAN				1,36	**3,31**	**2,64**	1,04<br>**1,11**	**0,27**<br>**0,49**	**0,19**<br>**0,48**
U2-associated protein SR140	O15042|SR140_HUMAN							1,02<br>**0,58**	0,83<br>0,63	0,60<br>0,72
U3 small nucleolar RNA-interacting protein 2	O43818|U3IP2_HUMAN							0,00	0,00	0,00
U4/U6 small nuclear ribonucleoprotein Prp31	Q8WWY3|PRP31_HUMAN							0,69	0,29	0,08
U4/U6,U5 tri-snRNP-associated protein 1	O43290|SNUT1_HUMAN							0,92<br>1,08	**0,26**<br>**0,54**	**0,12**<br>**0,35**
U4/U6,U5 tri-snRNP-associated protein 2	Q53GS9|SNUT2_HUMAN							**0,42**	0,90	0,60
U5 small nuclear ribonucleoprotein 200 kDa helicase	O75643|U520_HUMAN							1,01<br>**0,30**	**0,57**<br>**0,51**	0,58<br>**0,46**
U5-116KD protein	Q6IBM8|Q6IBM8_HUMAN							**0,31**	**0,41**	**0,44**
U6 snRNA-associated Sm-like protein LSm4	Q9Y4Z0|LSM4_HUMAN							1,12<br>1,01	**0,49**<br>0,42	**0,30**<br>**0,35**
U6 snRNA-associated Sm-like protein LSm7	Q9UK45|LSM7_HUMAN							0,88<br>1,03	**0,49**<br>0,70	**0,29**<br>0,39
U6 snRNA-associated Sm-like protein LSm8	O95777|LSM8_HUMAN				0,00	0,00	0,00	0,84<br>1,17	**0,47**<br>**0,54**	**0,26**<br>**0,28**
Ubiquilin-2	Q9UHD9|UBQL2_HUMAN				0,00	0,00	0,00			
Ubiquinol-cytochrome c reductase complex chaperone CBP3 homolog	Q9NVA1|UQCC_HUMAN	0,88<br>0,98	0,97<br>0,77	1,10						
Ubiquinone biosynthesis methyltransferase COQ5, mitochondrial precursor	Q5HYK3|COQ5_HUMAN	0,96	0,73							
Ubiquinone biosynthesis monooxygenase COQ6	Q9Y2Z9|COQ6_HUMAN	0,89	0,79							
Ubiquinone biosynthesis protein COQ9, mitochondrial precursor	O75208|COQ9_HUMAN	1,02<br>**0,75**	1,03<br>**0,84**	0,99						
Ubiquitin A-52 residue ribosomal protein fusion product 1	Q3MIH3|Q3MIH3_HUMAN	0,97	1,01	1,05	**0,94**<br>**0,87**	**0,91**<br>**0,80**	**0,67**<br>0,60	**1,19**	0,33	0,25
Ubiquitin carboxyl-terminal hydrolase 15	Q9Y4E8|UBP15_HUMAN				0,88	1,26	1,06			
Ubiquitin carboxyl-terminal hydrolase 5	P45974|UBP5_HUMAN				0,83<br>1,05	1,05<br>**1,12**	0,88<br>0,96			
Ubiquitin-associated protein 2-like	Q14157|UBP2L_HUMAN				1,13<br>0,89	0,84<br>0,78	0,97<br>0,76	**0,70**<br>0,92	**0,29**<br>**0,71**	**0,23**<br>**0,53**
Ubiquitin-conjugating enzyme E2 J1	Q9Y385|UB2J1_HUMAN	1,02	1,11							
Ubiquitin-conjugating enzyme E2 K	P61086|UBE2K_HUMAN				0,83	**0,82**	0,63			
Ubiquitin-conjugating enzyme E2 L3	P68036|UB2L3_HUMAN				**0,77**<br>**0,64**	0,89<br>**0,79**	**0,77**<br>**0,68**			
Ubiquitin-conjugating enzyme E2 N	P61088|UBE2N_HUMAN				**0,87**	0,80	0,59			
Ubiquitin-conjugating enzyme E2 variant 1	Q13404|UB2V1_HUMAN				**0,81**	1,00	0,92			
Ubiquitin-conjugating enzyme E2 variant 2	Q15819|UB2V2_HUMAN				0,97	0,97	0,98			
Ubiquitin-fold modifier 1	Q5VXS0|Q5VXS0_HUMAN				0,90	1,33	1,06			
Ubiquitin-like modifier activating enzyme 1	Q5JRR8|Q5JRR8_HUMAN	1,60<br>1,11	1,53<br>1,32	2,64	**0,80**<br>0,94	0,93<br>1,08	**0,88**<br>**0,82**			
Ubiquitin-like modifier activating enzyme 7	Q9BRB2|Q9BRB2_HUMAN				0,89<br>1,01	**1,05**<br>0,94	0,67<br>0,84			
Ubiquitin-like modifier-activating enzyme 6	A0AVT1|UBA6_HUMAN				0,97	1,08	1,00			
Ubiquitin-like protein 5	Q9BZL1|UBL5_HUMAN									

					0,00	0,00	0,00			
UBX domain-containing protein 8	Q96CS3|UBXD8_HUMAN	1,05	1,27							
UDP galactose 4'-epimerase	Q38G75|Q38G75_HUMAN				0,00	0,00	0,00			
UDP-glucose:glycoprotein glucosyltransferase 1 precursor	Q9NYU2|UGGG1_HUMAN	1,03<br>**0,93**	**1,14**<br>**1,09**	1,08				1,60	1,70	4,84
UDP-glucuronic acid decarboxylase 1	Q8NBZ7|UXS1_HUMAN	1,18	1,06							
UDP-N-acetylglucosamine--dolichyl-phosphate N-acetylglucosaminephosphotransferase	Q9H3H5|GPT_HUMAN	0,98<br>0,90	1,13<br>1,11	1,02						
UMP-CMP kinase	P30085|KCY_HUMAN				0,75<br>0,84	0,86<br>0,86	**0,66**<br>0,75			
UNC84B protein	Q2T9F7|Q2T9F7_HUMAN	0,00<br>0,00	0,00<br>0,00	0,00						
Uncharacterized aarF domain-containing protein kinase 1 precursor	Q86TW2|ADCK1_HUMAN	1,43<br>0,77	1,55<br>0,79	1,27						
Uncharacterized aarF domain-containing protein kinase 4	Q96D53|ADCK4_HUMAN	1,01	0,82							
Uncharacterized aarF domain-containing protein kinase 5	Q3MIX3|ADCK5_HUMAN	0,74	0,87							
Uncharacterized C7orf55 protein	Q96HJ9|CG055_HUMAN	0,00	0,00	0,00						
Uncharacterized metallophosphoesterase CSTP1	Q9BRF8|CSTP1_HUMAN				0,86<br>**0,85**	0,99<br>1,06	0,92<br>0,81			
Uncharacterized MFS-type transporter C19orf28	Q6NUT3|CS028_HUMAN	1,60	1,41	2,29						
Uncharacterized protein ACAA2 (Fragment)	A8MVT9|A8MVT9_HUMAN	1,04	**0,92**							
Uncharacterized protein AHCTF1P	A8MSG9|A8MSG9_HUMAN							0,93<br>**1,47**	0,58<br>**1,66**	0,61<br>**1,50**
Uncharacterized protein AKAP13 (Fragment)	A8MVN0|A8MVN0_HUMAN				0,81	1,09	1,18			
Uncharacterized protein ANXA6 (Fragment)	A6NN80|A6NN80_HUMAN	**1,58**	**1,92**	**2,98**	**1,39**<br>1,49	1,05<br>1,10	**1,81**<br>1,72	**1,69**<br>1,10	**0,63**<br>**0,82**	**0,74**<br>**0,67**
Uncharacterized protein ATP2A3	A8MYK9|A8MYK9_HUMAN	1,14<br>0,89	1,33<br>1,05	1,34						
Uncharacterized protein BCAP29	A6NFI0|A6NFI0_HUMAN	0,86<br>0,98	1,11<br>1,06	1,28						
Uncharacterized protein C10orf58 precursor	Q9BRX8|CJ058_HUMAN	**0,74**<br>1,08	0,92<br>1,07	1,04	**2,14**	**1,47**	1,47	0,92	3,78	5,43
Uncharacterized protein C12orf62	Q96I36|CL062_HUMAN	0,68	0,74	0,50						
Uncharacterized protein C17orf25	Q9Y3E8|Q9Y3E8_HUMAN				0,81<br>0,84	0,93<br>0,99	0,98<br>0,89			
Uncharacterized protein C17orf37	Q9BRT3|CQ037_HUMAN				0,63	0,96	0,85			
Uncharacterized protein C17orf42	Q96QE5|Q96QE5_HUMAN	0,89	1,02							
Uncharacterized protein C17orf62	Q9BQA9|CQ062_HUMAN	1,09<br>0,89	1,40<br>0,81	1,29						
Uncharacterized protein C17orf85	Q53F19|CQ085_HUMAN							0,62	0,23	0,14
Uncharacterized protein C17orf89	A1L188|CQ089_HUMAN	0,00<br>0,82	0,00<br>0,93	0,00						
Uncharacterized protein C18orf19	Q96ND0|CR019_HUMAN	1,18<br>0,86	1,10<br>0,86	1,05						
Uncharacterized protein C18orf32	Q8TCD1|CR032_HUMAN	0,00	0,00	0,00						
Uncharacterized protein C18orf8	Q96DM3|MIC1_HUMAN	0,00<br>0,87	0,00<br>1,07	0,00						
Uncharacterized protein C19orf43	Q9BQ61|CS043_HUMAN							0,95	**0,57**	**0,18**
Uncharacterized protein C19orf52	Q9BSF4|CS052_HUMAN	0,84	0,96							
Uncharacterized protein C19orf62	Q9NWV8|CS062_HUMAN				0,86<br>0,63	0,90<br>0,90	1,12<br>1,10			
Uncharacterized protein C1orf123	Q9NWV4|CA123_HUMAN				0,76	0,91	0,81			
Uncharacterized protein C1orf135	Q9H7T9|CA135_HUMAN	0,00	0,00							
Uncharacterized protein C1orf151	A8MPU7|A8MPU7_HUMAN	**1,39**	**1,31**	**1,25**						
Uncharacterized protein C1orf31	Q5JTJ3|CA031_HUMAN	0,00	0,00							
Uncharacterized protein C1orf77	Q9Y3Y2|CA077_HUMAN							0,84<br>1,21	**0,50**<br>0,87	**0,31**<br>0,70
Uncharacterized protein C20orf116 precursor	Q96HY6|CT116_HUMAN	1,04<br>1,22	1,13<br>1,09	1,27				1,14<br>**1,48**	**2,46**<br>**3,24**	**2,22**<br>**3,32**
Uncharacterized protein C20orf72	Q9BQP7|CT072_HUMAN	0,94	0,93							
Uncharacterized protein C2orf18 precursor	Q8N357|CB018_HUMAN	0,93<br>0,86	0,92<br>0,85	0,92	0,00	0,00	0,00	0,00	0,00	0,00
Uncharacterized protein C2orf33	Q9GZY8|CB033_HUMAN	0,94	0,76							
Uncharacterized protein C2orf47, mitochondrial precursor	Q8WWC4|CB047_HUMAN	1,04<br>**1,11**	0,96<br>1,00	0,99						
Uncharacterized protein C3orf1	Q9NPL8|CC001_HUMAN	0,84<br>1,05	**0,81**<br>1,12	**0,67**						
Uncharacterized protein C3orf60	Q9BU61|CC060_HUMAN	**0,74**<br>0,83	0,64<br>0,92	0,78						
Uncharacterized protein C3orf68	Q7Z7K0|CC068_HUMAN	0,84	**0,87**							
Uncharacterized protein C4orf14	Q8NC60|CD014_HUMAN	1,04<br>1,06	1,27	0,90						
Uncharacterized protein C4orf32	Q8N8J7|CD032_HUMAN	1,01	1,22							
Uncharacterized protein C6orf125	Q9BRT2|CF125_HUMAN	0,84	1,06	0,86						
Uncharacterized protein C6orf203	Q9P0P8|CF203_HUMAN	**0,85**	**0,80**							
Uncharacterized protein C7orf24 (gamma-glutamylcyclotransferase)	O75223|CG024_HUMAN				0,90	1,08	0,84			
Uncharacterized protein C7orf28	O95766|CG028_HUMAN	1,04	0,91							
Uncharacterized protein C7orf30	Q96EH3|CG030_HUMAN	1,14	0,91							
Uncharacterized protein C9orf105	Q8N4H5|CI105_HUMAN	0,92<br>1,03	**0,80**<br>**0,90**	**0,76**				0,97<br>**1,25**	**1,34**<br>0,94	**1,50**<br>**1,45**
Uncharacterized protein C9orf46	Q9HBL7|CI046_HUMAN	**1,24**<br>0,96	1,01<br>1,02	**0,77**				0,99<br>0,00	**2,33**<br>0,00	1,32<br>0,00
Uncharacterized protein C9orf80	Q9UDY4|DNJB4_HUMAN							1,14	**2,46**	**2,22**
Uncharacterized protein CAMK2D	A8MVS8|A8MVS8_HUMAN	0,00	0,00	0,00						
Uncharacterized protein CAPZA2	A8MW68|A8MW68_HUMAN	0,90	1,16							
Uncharacterized protein CD97 (Fragment)	A8MZ46|A8MZ46_HUMAN	**1,33**	1,03	1,11						

Uncharacterized protein DDTL	A6NHG4|A6NHG4_HUMAN				0,82	0,92	0,91			
Uncharacterized protein DIAPH1 (Fragment)	A6NF18|A6NF18_HUMAN				**0,82**	0,94	1,03			
Uncharacterized protein DKC1	A8MUT5|A8MUT5_HUMAN							0,96<br>**0,33**	**0,75**<br>**0,51**	**0,65**<br>**0,58**
Uncharacterized protein ENSP00000249480	A6NK07|A6NK07_HUMAN							0,75	**1,71**	**1,60**
Uncharacterized protein ENSP00000275524	A6NC28|A6NC28_HUMAN							1,42	0,82	0,90
Uncharacterized protein ENSP00000281581	P0C221|YN004_HUMAN							0,82	3,42	4,57
Uncharacterized protein ENSP00000329640	A6NMY6|A6NMY6_HUMAN				0,00	0,00	0,00			
Uncharacterized protein ENSP00000330892 (Fragment)	A6NN96|A6NN96_HUMAN				0,95	1,18	1,31			
Uncharacterized protein ENSP00000331275 (Fragment)	A6NNT3|A6NNT3_HUMAN				0,00	0,00	0,00			
Uncharacterized protein ENSP00000343430 (Fragment)	A6NK33|A6NK33_HUMAN							0,82	0,32	0,29
Uncharacterized protein ENSP00000344759	A6NL28|A6NL28_HUMAN				0,96	0,98	0,98			
Uncharacterized protein ENSP00000347049	A8MSU5|A8MSU5_HUMAN				0,99	**0,80**	**0,70**	**0,86**	**0,60**	**0,75**
Uncharacterized protein ENSP00000350479	A6NIH1|A6NIH1_HUMAN	**1,36**	**1,19**							
Uncharacterized protein ENSP00000351543 (Fragment)	A6NI39|A6NI39_HUMAN				1,49	1,46	**2,18**	0,89	**0,76**	1,04
Uncharacterized protein ENSP00000368180	A6NLS3|A6NLS3_HUMAN				0,00	0,00	**0,00**			
Uncharacterized protein ENSP00000373067	A6NCB7|A6NCB7_HUMAN							1,01	**0,26**	**0,12**
Uncharacterized protein ENSP00000381615 (Fragment)	A8MYH7|A8MYH7_HUMAN	0,00	0,00		1,34	3,05	2,13			
Uncharacterized protein ENSP00000382692								0,00	0,00	0,00
Uncharacterized protein ERO1L	A8MYW1|A8MYW1_HUMAN	0,94	1,02							
Uncharacterized protein FAM129A (Fragment)	A8MYQ4|A8MYQ4_HUMAN				0,86	0,90	0,97			
Uncharacterized protein FUBP1	A6NL74|A6NL74_HUMAN				**1,31**	**1,60**	**1,65**	**1,41**	**0,38**	**0,39**
Uncharacterized protein HCFC1	A6NEM2|A6NEM2_HUMAN							0,65	0,59	0,62
Uncharacterized protein HNRNPL	A6ND69|A6ND69_HUMAN	0,00	0,00	0,00	0,00	0,00	0,00			
Uncharacterized protein HVCN1 (Fragment)	A8MQ37|A8MQ37_HUMAN							0,00	0,00	0,00
Uncharacterized protein KIAA0090 precursor	Q8N766|K0090_HUMAN	0,94<br>**0,84**	0,92<br>0,98	**0,85**						
Uncharacterized protein KIAA0152 precursor	Q14165|K0152_HUMAN	0,93<br>1,01	0,96<br>**1,19**	0,91				1,07<br>**0,72**	**2,39**<br>**2,09**	**4,81**<br>**2,98**
Uncharacterized protein KIAA2013 precursor	Q8IYS2|K2013_HUMAN	1,04<br>1,07	**0,62**<br>1,08	0,76						
Uncharacterized protein LAIR1	A8MZ84|A8MZ84_HUMAN				0,00	0,00	0,00			
Uncharacterized protein LYRM7 (Fragment)	A8MPQ9|A8MPQ9_HUMAN	0,00<br>0,84	0,00<br>0,96	0,00						
Uncharacterized protein MATR3	A8MXP9|A8MXP9_HUMAN							**0,75**	**0,18**	**0,13**
Uncharacterized protein MRPL1	A6NG03|A6NG03_HUMAN	0,97	0,98							
Uncharacterized protein NACAP1	A8MTN7|A8MTN7_HUMAN							0,00	0,00	0,00
Uncharacterized protein PHACTR2 (Fragment)	A8MZC0|A8MZC0_HUMAN				0,00	0,00	0,00			
Uncharacterized protein PICALM	A8MW24|A8MW24_HUMAN	**1,37**	**0,69**							
Uncharacterized protein PSME1	A6NJG9|A6NJG9_HUMAN				**0,84**	0,99	**1,16**			
Uncharacterized protein SFRS2IP	A6NEU9|A6NEU9_HUMAN							1,14	**0,57**	**0,47**
Uncharacterized protein SLC4A7	A6NIA8|A6NIA8_HUMAN	1,15	1,00							
Uncharacterized protein SLCO1A2	A6NLB2|A6NLB2_HUMAN							0,50	**6,19**	**6,70**
Uncharacterized protein SRI (Fragment)	A8MTH6|A8MTH6_HUMAN				1,30	1,16	**1,61**			
Uncharacterized protein SRRM1	A9Z1X7|A9Z1X7_HUMAN							1,00<br>1,05	0,62<br>**2,83**	**0,40**<br>**2,59**
Uncharacterized protein SUMO3	A8MUA9|A8MUA9_HUMAN							1,03<br>**1,29**	**0,33**<br>0,93	**0,13**<br>0,53
Uncharacterized protein TBCB	A8MVD5|A8MVD5_HUMAN				1,08	1,02	1,46			
Uncharacterized protein TUBA4A	A6NHY4|A6NHY4_HUMAN	1,03	1,39	2,18						
Uncharacterized protein TUBA4A	A8MUB1|A8MUB1_HUMAN	0,00	0,00	0,00						
Uncharacterized protein VAMP2	A8MWB3|A8MWB3_HUMAN	0,90<br>1,05	0,89<br>**1,12**	0,80	**1,99**	1,54	1,40			
Uncharacterized protein VAPA	A6NDZ0|A6NDZ0_HUMAN	1,17	1,10		**1,86**<br>**1,95**	1,16<br>**1,32**	1,57<br>1,27	**1,21**	**2,33**	**2,43**
Uncharacterized protein ZNF326	A8MYX1|A8MYX1_HUMAN							0,83	**0,35**	0,23
Uncharacterized serine/threonine-protein kinase SgK494								0,00	0,00	0,00
UPF0195 protein FAM96A	Q9H5X1|FA96A_HUMAN				0,00	0,00	0,00			
UPF0197 protein C11orf10	P61165|CK010_HUMAN							0,59	2,19	1,76
UPF0404 protein C11orf59	Q6IAA8|CK059_HUMAN							0,00	0,00	0,00
UPF0240 protein C6orf66	Q9P032|CF066_HUMAN	0,73<br>1,03	0,71<br>1,01	0,72						
UPF0287 protein C16orf61	Q9NRP2|CP061_HUMAN	0,00<br>1,11	0,00<br>1,03	0,00						
UPF0317 protein C14orf159, mitochondrial precursor	Q7Z3D6|CN159_HUMAN	1,00	1,14	0,84						
UPF0318 protein FAM120A	Q9NZB2|F120A_HUMAN				0,81	0,74	0,75			
UPF0368 protein Cxorf26	Q9BVG4|CX026_HUMAN				0,71	0,70	0,62			
UPF0369 protein C6orf57 precursor	Q5VUM1|CF057_HUMAN	0,97	0,82	0,49						
UPF0404 protein C11orf59	Q6IAA8|CK059_HUMAN	0,92	**1,27**	**1,44**				2,10	2,47	2,84
UPF0414 transmembrane protein C20orf30	Q96A57|CT030_HUMAN							1,51	5,24	3,84
UPF0420 protein C16orf58	Q96GQ5|CP058_HUMAN									

		1,02	0,84							
UPF0465 protein C5orf33	Q4G0N4|CE033_HUMAN	0,64<br>0,98	0,65<br>0,91	0,88						
UPF0466 protein C22orf32, mitochondrial precursor	Q9H4I9|CV032_HUMAN	**0,70**	0,69	**0,58**				0,93	1,27	1,49
UPF0480 protein C15orf24 precursor	Q9NPA0|CO024_HUMAN	0,99<br>0,95	1,09<br>1,18	1,16				0,85<br>**1,35**	**2,73**<br>**2,49**	**2,59**<br>**3,33**
UPF0485 protein C1orf144	Q7Z422|CA144_HUMAN				0,00	0,00	0,00			
UPF0510 protein C19orf63 precursor	Q5UCC4|CS063_HUMAN	0,88<br>1,03	0,95<br>0,93	0,84				0,89<br>1,00	**3,55**<br>**2,60**	**3,74**<br>**3,10**
UPF0511 protein C2orf56, mitochondrial precursor	Q7L592|CB056_HUMAN	1,07	0,89							
UPF0525 protein KIAA1754 precursor	Q8IWB1|K1754_HUMAN	0,00	0,00	0,00						
UPF0535 membrane protein	Q6NUQ4|U535_HUMAN	**0,74**<br>0,87	0,71<br>0,96	**0,75**				0,38	0,83	0,80
UPF0554 protein C2orf43	Q9H6V9|CB043_HUMAN	0,80	0,86							
UPF0555 protein KIAA0776	O94874|K0776_HUMAN	0,96<br>**1,27**	1,48<br>**1,24**	**1,90**						
UPF0556 protein C19orf10 precursor	Q969H8|CS010_HUMAN	1,05<br>0,99	1,12<br>1,08	0,98						
Up-regulated during skeletal muscle growth protein 5	Q96IX5|USMG5_HUMAN	0,80	0,89							
UQCRB protein	Q6FGD1|Q6FGD1_HUMAN	0,92<br>1,05	**0,85**<br>1,04	**0,79**				1,10<br>**2,08**	0,89<br>1,16	**1,53**<br>**2,30**
UQCRH protein	Q567R0|Q567R0_HUMAN				**2,14**	1,28	1,64	**1,24**<br>**1,87**	0,84<br>1,14	**1,57**<br>**2,02**
Uridine phosphorylase 1	Q16831|UPP1_HUMAN				0,00	0,00	0,00			
Urokinase plasminogen activator surface receptor precursor	Q03405|UPAR_HUMAN	1,05	1,05					2,61	1,04	1,49
Uroporphyrinogen decarboxylase	Q53ZP6|Q53ZP6_HUMAN				1,02	1,05	1,01			
UTP--glucose-1-phosphate uridylyltransferase	Q16851|UGPA_HUMAN				0,93<br>1,09	1,01<br>1,04	**1,17**<br>**1,20**			
Utrophin	P46939|UTRO_HUMAN	1,12<br>1,11	1,37<br>1,31	1,51						
UV excision repair protein RAD23 homolog B	P54727|RD23B_HUMAN				**0,87**	1,09	0,95	0,99	**0,52**	**0,35**
UV excision repair protein RAD23 homolog B variant (Fragment)	Q53F10|Q53F10_HUMAN				0,67	**0,85**	**0,74**	1,14	0,92	0,74
Vacuolar ATP synthase catalytic subunit A	P38606|VATA_HUMAN	**1,15**<br>**0,92**	**1,69**<br>**1,09**	**1,67**	1,05<br>1,30	0,93<br>0,90	0,89<br>1,08	**1,69**<br>**0,61**	**2,94**<br>**1,84**	**7,07**<br>**2,95**
Vacuolar ATP synthase subunit B, brain isoform	P21281|VATB2_HUMAN	**1,19**<br>**0,86**	**1,55**<br>**1,08**	**1,54**	1,04<br>**1,18**	**0,85**<br>0,97	**0,86**<br>1,16	**1,57**<br>**0,60**	**3,28**<br>**1,62**	**7,42**<br>**2,58**
Vacuolar protein sorting-associated protein 13D	Q5THJ4|VP13D_HUMAN	0,91	1,04							
Vacuolar protein sorting-associated protein 16 homolog	Q9H269|VPS16_HUMAN	0,00<br>1,06	0,00<br>1,08	0,00						
Vacuolar protein sorting-associated protein 18 homolog	Q9P253|VPS18_HUMAN	0,00<br>0,90	0,00<br>1,14	0,00						
Vacuolar protein sorting-associated protein 28 homolog	Q9UK41|VPS28_HUMAN				0,90	0,94	0,72			
Vacuolar protein sorting-associated protein 29	Q9UBQ0|VPS29_HUMAN	**1,51**	1,22		1,06<br>0,78	**1,12**<br>0,99	1,26<br>1,08			
Vacuolar protein sorting-associated protein 35	Q96QK1|VPS35_HUMAN	1,26	1,05		0,80<br>0,81	1,09<br>0,99	0,98<br>1,42			
Vacuolar protein-sorting-associated protein 25	Q9BRG1|VPS25_HUMAN				0,67	0,94	1,12			
Vacuolar proton pump subunit C 1	P21283|VATC1_HUMAN	**1,30**<br>**0,87**	**1,71**<br>1,05	**1,63**	1,03<br>1,08	0,91<br>**0,82**	0,81<br>0,77	**1,73**<br>1,02	**3,58**<br>**1,64**	**6,11**<br>**2,29**
Vacuolar proton pump subunit D	Q9Y5K8|VATD_HUMAN	**1,38**<br>0,89	1,62<br>0,89	**1,48**				**1,43**<br>**1,49**	**2,79**<br>**2,09**	**3,88**<br>**2,92**
Vacuolar proton pump subunit d 1	P61421|VA0D1_HUMAN	1,01<br>1,02	1,02<br>1,06	1,01	1,28<br>2,25	0,74<br>1,02	0,94<br>**1,22**	**2,31**<br>0,80	**2,37**<br>**1,34**	**4,54**<br>**1,84**
Vacuolar proton pump subunit F	Q16864|VATF_HUMAN	1,04<br>**0,79**	**1,32**<br>1,03	1,15	1,12	0,81	0,92	1,12<br>**1,73**	**1,96**<br>**2,38**	**2,65**<br>**2,74**
Vacuolar proton pump subunit H	Q9UI12|VATH_HUMAN	**1,33**<br>**0,80**	**1,87**<br>1,17	**1,70**	1,08	1,32	0,85	**1,27**<br>0,54	1,38<br>1,38	2,79<br>2,12
Vacuolar proton translocating ATPase 116 kDa subunit a isoform 1	Q93050|VPP1_HUMAN	1,15<br>0,92	1,00<br>0,90	1,01						
Valacyclovir hydrolase precursor	Q86WA6|BPHL_HUMAN	**0,89**	0,86							
Valosin-containing protein	Q2TAI5|Q2TAI5_HUMAN	1,03<br>**1,15**	1,23<br>**1,20**	1,18	0,99<br>1,05	1,04<br>**1,16**	**1,33**<br>1,29	1,20<br>**0,57**	1,15<br>**1,33**	1,48<br>**1,47**
Valyl-tRNA synthetase	P26640|SYV_HUMAN				1,16	0,88	0,92			
Valyl-tRNA synthetase-like protein (Fragment)	Q59FI1|Q59FI1_HUMAN	0,96	1,28							
VAMP (Vesicle-associated membrane protein)-associated protein B and C	Q53XM7|Q53XM7_HUMAN							1,05<br>1,05	1,94<br>1,48	1,35<br>1,56
WARS protein	Q502Y0|Q502Y0_HUMAN				0,93	**1,17**	1,08			
Vasodilator-stimulated phosphoprotein	P50552|VASP_HUMAN	1,03<br>1,39	1,19<br>1,41	2,45	**0,80**<br>**0,75**	0,95<br>**0,80**	0,86<br>0,60	**0,82**<br>**0,66**	**0,57**<br>**0,72**	**0,35**<br>**0,37**
VAV1 protein	Q96D37|Q96D37_HUMAN	0,95	2,79	4,49						
WD repeat and FYVE domain-containing protein 1	Q8IWB7|WDFY1_HUMAN	1,17<br>**0,88**	0,84<br>**0,79**	0,82						
WD repeat-containing protein 1 isoform 1 variant (Fragment)	Q59ER5|Q59ER5_HUMAN	1,83	1,45	2,17	1,00<br>1,04	**1,24**<br>**1,25**	**1,12**<br>1,14			
Very long-chain specific acyl-CoA dehydrogenase, mitochondrial precursor	P49748|ACADV_HUMAN	**0,88**<br>**0,92**	**0,87**<br>**0,93**	**0,80**						
Vesicle transport protein GOT1B	Q9Y3E0|GOT1B_HUMAN	**1,23**<br>0,79	**1,19**<br>0,59	**1,23**						
Vesicle transport protein SFT2B	O95562|SFT2B_HUMAN	0,83	0,92	2,26						
Vesicle transport through interaction with t-SNAREs homolog 1B	Q9UEU0|VTI1B_HUMAN	1,18<br>1,17	1,05<br>1,25	1,10				1,20	1,15	1,48
Vesicle-associated membrane protein 2	Q9BUC2|Q9BUC2_HUMAN							1,21<br>1,14	3,06<br>**2,74**	**3,67**<br>**3,88**
Vesicle-associated membrane protein 7	P51809|VAMP7_HUMAN	0,96	0,95	0,79						
Vesicle-associated membrane protein 8	Q9BV40|VAMP8_HUMAN	1,07<br>0,92	1,02<br>0,97	1,17	1,52<br>2,46	0,81<br>1,33	1,07<br>0,73	0,91<br>1,22	**2,27**<br>**2,91**	**2,67**<br>**4,20**
Vesicle-associated membrane protein-associated protein A	Q9P0L0|VAPA_HUMAN	**0,79**	0,91	1,10				**0,74**	**1,85**	**2,24**
Vesicle-fusing ATPase	P46459|NSF_HUMAN	**1,08**<br>**1,24**	0,97<br>1,07	**1,29**	1,09	1,32	0,87			
Vesicle-trafficking protein SEC22b	O75396|SC22B_HUMAN	1,07<br>1,02	1,17<br>1,26	**1,37**	**1,75**<br>**1,85**	1,14<br>1,09	1,12<br>0,87	**1,19**<br>**1,77**	**3,41**<br>**3,41**	**3,18**<br>**3,87**
Vesicular integral-membrane protein VIP36 precursor	Q12907|LMAN2_HUMAN	**1,29**	1,10	1,11				1,14<br>0,93	**4,37**<br>**4,44**	**6,28**<br>**5,51**
Vimentin	P08670|VIME_HUMAN	**1,13**<br>**1,12**	**2,65**<br>**2,23**	**5,85**	**1,94**<br>2,20	**2,61**<br>3,05	**2,33**<br>2,72	**0,92**<br>0,45	**0,39**<br>0,29	**0,26**<br>0,23
Vinculin	P18206|VINC_HUMAN	0,00<br>1,31	0,00<br>1,20	0,00	**0,85**<br>0,80	0,97<br>1,01	1,01<br>**0,90**			

Wiskott-Aldrich syndrome protein	P42768|WASP_HUMAN				1,05<br>**0,70**	0,79<br>**0,77**	0,58<br>**0,70**			
Wiskott-Aldrich syndrome protein family member 2	Q9Y6W5|WASF2_HUMAN				0,77<br>**0,69**	0,71<br>0,81	0,76<br>0,76	0,95<br>**0,82**	1,17<br>**1,53**	1,13<br>**1,50**
Vitamin K epoxide reductase complex subunit 1-like protein 1	Q8N0U8|VKORL_HUMAN	0,85	0,92							
Wolframin	O76024|WFS1_HUMAN	0,93<br>0,93	1,08<br>1,05	0,98	0,00	0,00	0,00			
Voltage-dependent anion-selective channel protein 1	P21796|VDAC1_HUMAN	1,08<br>0,99	0,95<br>1,00	**0,85**	1,79<br>2,14	1,17<br>1,26	1,13<br>2,20	0,91<br>**1,40**	**1,45**<br>1,01	**1,33**<br>**1,35**
Voltage-dependent anion-selective channel protein 2	P45880|VDAC2_HUMAN	**1,16**<br>0,99	1,05<br>1,06	0,92	2,13	1,22	0,94	1,55<br>**0,38**	1,78<br>**0,87**	**2,61**<br>1,00
Voltage-dependent anion-selective channel protein 3	Q9Y277|VDAC3_HUMAN	**1,37**<br>1,04	1,16<br>**1,26**	0,96				**1,76**<br>0,90	1,53<br>0,76	**3,05**<br>0,88
Voltage-gated calcium channel beta 2 subunit splice variant CavB2cN2	Q6TME2|Q6TME2_HUMAN							0,00	0,00	0,00
Voltage-gated potassium channel subunit beta-2	Q13303|KCAB2_HUMAN				1,04	1,04	0,86			
V-set and immunoglobulin domain-containing protein 4 precursor	Q9Y279|VSIG4_HUMAN	0,00<br>1,22	0,00<br>0,59	0,00						
WW domain-binding protein 11	Q9Y2W2|WBP11_HUMAN							0,88<br>1,11	**0,35**<br>**0,63**	**0,23**<br>0,68
WW domain-binding protein 2	Q969T9|WBP2_HUMAN				0,56<br>0,92	0,51<br>1,49	0,38<br>0,95			
WW domain-binding protein 7	Q9UMN6|WBP7_HUMAN	0,96	1,02							
Xaa-Pro aminopeptidase 1	Q9NQW7|XPP1_HUMAN				**0,75**	0,98	0,77			
Xaa-Pro dipeptidase	P12955|PEPD_HUMAN				0,91	1,04	0,85			
XTP3-transactivated gene A protein	Q9H773|XTP3A_HUMAN				0,00	0,00	0,00			
XTP3-transactivated gene B protein precursor	Q96DZ1|XTP3B_HUMAN	0,88<br>0,83	0,88<br>1,02	1,14						
YBX1 protein (Fragment)	Q6PKI6|Q6PKI6_HUMAN	1,02	**1,84**	**2,61**	0,00	0,00	0,00			
YLP motif-containing protein 1	P49750|YLPM1_HUMAN							0,91<br>1,04	**0,26**<br>**0,56**	**0,21**<br>0,48
YTH domain family protein 3	Q7Z739|YTHD3_HUMAN							0,00	0,00	0,00
Zinc finger CCCH domain-containing protein 11A	O75152|ZC11A_HUMAN							0,55<br>0,84	0,18<br>**0,54**	0,10<br>0,46
Zinc finger CCCH domain-containing protein 13	Q5T200|ZC3HD_HUMAN				1,02	0,99	1,12			
Zinc finger CCCH domain-containing protein 15	Q8WU90|ZC3HF_HUMAN				0,86	0,60	0,53	0,49	0,74	0,86
Zinc finger CCCH domain-containing protein 18	Q86VM9|ZCH18_HUMAN							**0,83**<br>0,97	**0,47**<br>0,81	**0,27**<br>0,64
Zinc finger CCCH domain-containing protein 4	Q9UPT8|ZC3H4_HUMAN							1,28	0,80	0,86
Zinc finger CCCH type antiviral protein 1	Q7Z2W4|ZCC2_HUMAN				0,00	0,00	0,00			
Zinc finger matrin-type protein 2	Q96NC0|ZMAT2_HUMAN							0,76	0,66	0,55
Zinc finger protein 207 variant (Fragment)	Q59G94|Q59G94_HUMAN							1,09<br>1,14	**0,37**<br>1,23	**0,32**<br>1,27
Zinc finger protein 326	Q5BKZ1|ZN326_HUMAN							0,92	0,60	0,70
Zinc finger protein 638	Q14966|ZN638_HUMAN							1,05	0,71	0,90
Zinc finger protein 787	Q6DD87|ZN787_HUMAN							0,00	0,00	0,00
Zinc finger protein 791	Q3KP31|ZN791_HUMAN							1,47	0,98	0,75
Zinc finger protein 9	Q5U0E9|Q5U0E9_HUMAN				0,93	0,96	0,63			
Zinc finger protein KIAA1802	Q96JM3|ZN828_HUMAN							0,00	0,00	0,00
Zinc finger Ran-binding domain-containing protein 2	O95218|ZRAB2_HUMAN							**1,79**<br>2,94	**1,85**<br>**2,95**	**0,49**<br>2,12
Zinc finger RNA-binding protein	Q96KR1|ZFR_HUMAN							0,00	0,00	0,00
Zinc phosphodiesterase ELAC protein 2	Q9BQ52|RNZ2_HUMAN	0,97	0,88							
Zinc transporter 1	Q9Y6M5|ZNT1_HUMAN	1,53<br>1,06	0,94<br>1,09	1,03						
Zinc transporter 5	Q8TAD4|ZNT5_HUMAN	1,63	1,56							
Zinc transporter 7	Q8NEW0|ZNT7_HUMAN	0,97<br>0,93	0,63<br>**0,78**	0,70						
Zinc transporter SLC39A7	Q92504|S39A7_HUMAN	1,05<br>0,96	1,01<br>1,08	0,96				1,14<br>0,00	**2,02**<br>0,00	**3,47**<br>0,00
Zinc transporter ZIP11	Q8N1S5|S39AB_HUMAN	1,41<br>0,89	1,50<br>0,89	1,97						
Zinc-binding alcohol dehydrogenase domain-containing protein 2	Q8N4Q0|ZADH2_HUMAN	1,04	1,00							
Zyxin	Q15942|ZYX_HUMAN				**0,87**<br>0,71	0,99<br>**0,82**	**0,90**<br>0,67	0,99<br>**1,35**	0,60<br>0,79	0,62<br>**0,64**

bold *= statistically significant quantitation result (p<0,05)*
0,00 = protein identified but no quantitation result

B. Secretome

Protein name	SwissProt access	secretome<br>6h (iTRAQ 115:114)	 9h (iTRAQ 116:114)	 12h (iTRAQ 117:114)
10 kDa heat shock protein, mitochondrial	P61604|CH10_HUMAN	**1,87**<br>4,76	**4,06**<br>3,31	**4,96**<br>8,84
14-3-3 protein beta/alpha	P31946|1433B_HUMAN	1,80<br>**2,91**	**2,88**<br>**3,12**	**3,76**<br>**6,98**
14-3-3 protein epsilon	P62258|1433E_HUMAN	1,86<br>**5,03**	**2,48**<br>**4,33**	**3,12**<br>**9,64**
14-3-3 protein eta	Q04917|1433F_HUMAN	**1,69**<br>3,52	**3,07**<br>**4,52**	**3,90**<br>**11,49**
14-3-3 protein gamma	P61981|1433G_HUMAN	**1,48**<br>2,66	**2,15**<br>**3,67**	**2,95**<br>5,21
14-3-3 protein theta	P27348|1433T_HUMAN	2,58<br>19,96	4,34<br>15,18	**4,89**<br>33,98
14-3-3 protein zeta/delta	P63104|1433Z_HUMAN	**1,52**<br>**2,24**	**2,39**<br>**2,37**	**3,28**<br>**6,08**
1-phosphatidylinositol-4,5-bisphosphate phosphodiesterase beta-2	Q00722|PLCB2_HUMAN	0,00<br>0,00	0,00<br>0,00	12,78<br>8,18
26S proteasome non-ATPase regulatory subunit 13	Q9UNM6|PSD13_HUMAN	**1,79**	**3,01**	**3,43**
28 kDa heat- and acid-stable phosphoprotein	Q13442|HAP28_HUMAN	2,40<br>0,00	6,35<br>0,00	8,32<br>9,77
40S ribosomal protein S10	P46783|RS10_HUMAN	1,96	**5,50**	**7,71**

40S ribosomal protein S15	P62841|RS15_HUMAN	3,71	8,83	14,52
		0,00	7,08	31,52
40S ribosomal protein S16	P62249|RS16_HUMAN	2,02	3,16	**5,41**
40S ribosomal protein S17	P08708|RS17_HUMAN		**10,42**	**12,54**
40S ribosomal protein S19	P39019|RS19_HUMAN	1,31	**6,96**	**10,96**
		2,24	4,47	20,35
40S ribosomal protein S20	P60866|RS20_HUMAN	2,54	**3,87**	**4,48**
		1,61	2,35	6,90
40S ribosomal protein S23	P62266|RS23_HUMAN	4,08	6,88	7,35
		0,00	0,00	5,38
40S ribosomal protein S28	P62857|RS28_HUMAN	2,49	6,32	7,87
		**6,29**	**6,40**	**21,24**
40S ribosomal protein S3	P23396|RS3_HUMAN	2,26	3,05	3,60
		0,00	0,00	7,35
40S ribosomal protein S6	P62753|RS6_HUMAN	3,12	10,59	12,36
		0,00	0,00	26,68
40S ribosomal protein S7	P62081|RS7_HUMAN	1,53	4,18	**5,10**
40S ribosomal protein SA	P08865|RSSA_HUMAN			
		2,21	3,14	**11,80**
45 kDa calcium-binding protein	Q9BRK5|CAB45_HUMAN	1,24	**2,01**	**2,38**
		1,54	2,43	**3,56**
60S acidic ribosomal protein P0	P05388|RLA0_HUMAN	**1,29**	**2,90**	**3,89**
60S acidic ribosomal protein P2	P05387|RLA2_HUMAN	2,24	6,31	11,42
		**3,89**	4,11	**9,71**
60S ribosomal protein L10a	P62906|RL10A_HUMAN	1,65	**3,08**	**3,54**
		1,99	2,20	**5,03**
60S ribosomal protein L11	P62913|RL11_HUMAN	1,92	**3,58**	3,58
60S ribosomal protein L12	P30050|RL12_HUMAN	**2,19**	**3,93**	**3,76**
		0,00	0,00	15,76
60S ribosomal protein L23	P62829|RL23_HUMAN	5,08	9,32	9,59
		1,86	1,64	3,17
60S ribosomal protein L23a	P62750|RL23A_HUMAN	**3,46**	**8,52**	**12,07**
		4,21	5,88	20,20
60S ribosomal protein L5	P46777|RL5_HUMAN	**2,76**	**8,90**	**9,50**
		4,82	8,48	**21,97**
60S ribosomal protein L6	Q02878|RL6_HUMAN	4,50	**8,14**	**8,87**
		4,06	4,30	14,31
60S ribosomal protein L7a	P62424|RL7A_HUMAN	0,00	5,55	4,57
		0,00	0,00	8,76
6-phosphogluconate dehydrogenase, decarboxylating	P52209|6PGD_HUMAN	**1,59**	**2,24**	**2,59**
		**2,18**	**2,26**	**7,09**
78 kDa glucose-regulated protein	P11021|GRP78_HUMAN	**1,74**	**2,77**	**2,01**
		**2,51**	**2,31**	**3,47**
Acid ceramidase	Q13510|ASAH1_HUMAN	2,98	**5,04**	**6,60**
		3,71	3,97	**14,66**
	P39687|AN32A_HUMAN	1,96	3,13	4,46
Acidic leucine-rich nuclear phosphoprotein 32 family member A		**2,64**	**2,85**	**7,96**
	Q92688|AN32B_HUMAN	2,08	3,03	**3,78**
Acidic leucine-rich nuclear phosphoprotein 32 family member B		3,77	3,69	8,89
Actin, cytoplasmic 1	P60709|ACTB_HUMAN	**1,81**	**3,15**	**3,85**
		0,00	0,00	0,00
Actin-related protein 2	P61160|ARP2_HUMAN	2,14	3,66	**3,79**
		**3,76**	4,40	**11,04**
Actin-related protein 2/3 complex subunit 1B	O15143|ARC1B_HUMAN	**2,24**	**3,18**	**3,69**
		**2,56**	**2,59**	**7,31**
Actin-related protein 2/3 complex subunit 2	O15144|ARPC2_HUMAN	**1,93**	**2,87**	**3,51**
		**3,01**	**3,49**	**7,70**
Actin-related protein 2/3 complex subunit 3	O15145|ARPC3_HUMAN	**1,67**	**3,16**	**3,85**
		**4,31**	**4,66**	**11,46**
Actin-related protein 2/3 complex subunit 4	P59998|ARPC4_HUMAN	2,38	3,19	3,33
		**2,90**	**3,57**	**7,26**
Actin-related protein 2/3 complex subunit 5	O15511|ARPC5_HUMAN	**2,66**	**4,80**	**5,88**
		**3,23**	**3,65**	**9,09**
Actin-related protein 3	P61158|ARP3_HUMAN	**1,90**	**2,96**	**2,82**
		**3,08**	**3,23**	**6,92**
Acylamino-acid-releasing enzyme	P13798|ACPH_HUMAN	1,29	1,93	2,34
		2,93	1,84	8,17
Acyl-CoA-binding protein	P07108|ACBP_HUMAN	**1,48**	**3,26**	**4,69**
		**2,91**	**3,45**	**13,76**
Adenosine deaminase CECR1	Q9NZK5|CECR1_HUMAN	**0,66**	0,71	0,67
		1,16	1,21	**1,78**
Adenosylhomocysteinase	P23526|SAHH_HUMAN	2,66	**3,28**	**3,71**
Adenylate kinase 2, mitochondrial	P54819|KAD2_HUMAN	2,89	5,41	8,00
		0,00	7,27	**15,34**
Adenylosuccinate synthetase isozyme 2	P30520|PURA2_HUMAN	2,30	**3,53**	**3,93**
Adenylyl cyclase-associated protein 1	Q01518|CAP1_HUMAN	**2,60**	**4,30**	**5,76**
		**3,80**	**3,78**	**10,98**
ADP-ribosylation factor 3	P61204|ARF3_HUMAN	4,07	7,85	**10,35**
Afamin	P43652|AFAM_HUMAN			
		**3,64**	**3,37**	**5,19**
Alcohol dehydrogenase [NADP+]	P14550|AK1A1_HUMAN	1,88	3,36	3,22
		2,52	2,42	**6,73**
Alcohol dehydrogenase class-3	P11766|ADHX_HUMAN	**2,86**	**4,47**	**4,75**
		2,29	1,82	6,37
Aldose 1-epimerase	Q96C23|GALM_HUMAN	1,44	1,54	1,95
		1,17	2,07	7,16
Aldose reductase	P15121|ALDR_HUMAN			
		**2,02**	2,05	**6,22**
Allograft inflammatory factor 1	P55008|AIF1_HUMAN	**2,47**	**6,42**	**10,41**
		**3,13**	**4,27**	**15,49**
Alpha-1-antitrypsin	P01009|A1AT_HUMAN	**0,74**	0,93	0,94
		**1,41**	1,25	**1,70**
Alpha-1B-glycoprotein	P04217|A1BG_HUMAN	2,42	3,31	3,75
		0,00	0,00	9,81
Alpha-2-HS-glycoprotein	P02765|FETUA_HUMAN	1,53	**2,12**	1,74
		**4,25**	**4,05**	**4,59**
Alpha-2-macroglobulin	P01023|A2MG_HUMAN	**0,59**	**0,56**	**0,49**
		**1,37**	0,83	**1,32**
Alpha-actinin-1	P12814|ACTN1_HUMAN	**1,66**	**2,05**	**2,29**
		**2,07**	**2,12**	**4,49**
Alpha-actinin-4	O43707|ACTN4_HUMAN	**1,49**	**2,33**	**2,51**
		**2,28**	**2,50**	**6,17**
Alpha-endosulfine	O43768|ENSA_HUMAN	3,40	7,96	**8,69**
		0,00	0,00	0,00
Alpha-enolase	P06733|ENOA_HUMAN	**1,48**	**2,47**	**3,01**
		**2,27**	**2,53**	**6,96**
Aminopeptidase N	P15144|AMPN_HUMAN	1,18	**1,91**	**2,18**
		3,80	4,65	**6,33**
Amyloid beta A4 protein OS=Pan troglodytes	Q5IS80|A4_PANTR	1,26	1,57	**1,70**
		2,88	2,04	**3,95**
Amyloid-like protein 2	Q06481|APLP2_HUMAN	0,96	1,13	1,45

		1,56	1,28	2,29
Annexin A1	P04083|ANXA1_HUMAN	**1,52**	**1,65**	**2,06**
		**2,87**	**3,48**	**5,88**
Annexin A11	P50995|ANX11_HUMAN	**1,47**	**2,57**	**3,59**
		1,81	3,26	**12,84**
Annexin A2	P07355|ANXA2_HUMAN	1,05	1,09	**1,47**
		**1,91**	1,37	**2,66**
Annexin A4	P09525|ANXA4_HUMAN	1,17	2,00	**2,68**
Annexin A5	P08758|ANXA5_HUMAN	1,09	**0,59**	0,94
		**2,21**	**1,32**	**2,52**
Annexin A6	P08133|ANXA6_HUMAN	1,35	**1,98**	**2,43**
		3,49	4,92	**12,25**
AP-2 complex subunit alpha-1	O95782|AP2A1_HUMAN	1,37	2,13	3,15
				7,06
Apolipoprotein A-II	P02652|APOA2_HUMAN	**4,80**	**6,04**	**5,64**
		6,07	5,48	8,89
Apolipoprotein B-100 receptor	Q0VD83|AB48R_HUMAN	**1,86**	**3,49**	**4,53**
		**3,69**	3,26	**7,90**
Apolipoprotein C-I	P02654|APOC1_HUMAN	1,22	0,77	0,90
		**2,58**	**2,99**	**6,08**
Apolipoprotein C-II	P02655|APOC2_HUMAN			
		5,65	6,58	**9,39**
Apolipoprotein E	P02649|APOE_HUMAN	0,85	**1,28**	**1,41**
		**2,74**	**2,03**	**3,41**
Apoptotic chromatin condensation inducer in the nucleus	Q9UKV3|ACINU_HUMAN	1,36	**2,22**	**3,48**
Astrocytic phosphoprotein PEA-15	Q15121|PEA15_HUMAN	1,78	**3,00**	**4,77**
		**3,86**	4,12	**10,07**
ATPase inhibitor, mitochondrial	Q9UII2|ATIF1_HUMAN	3,32	6,70	6,55
		9,28	0,00	9,50
ATP-citrate synthase	P53396|ACLY_HUMAN	1,63	**2,73**	**2,51**
		0,00	0,00	7,26
ATP-dependent DNA helicase 2 subunit 1	P12956|KU70_HUMAN	**1,71**	**2,73**	**3,91**
		2,27	1,80	5,18
Band 4.1-like protein 3	Q9Y2J2|E41L3_HUMAN	2,51	4,32	5,26
		6,38	4,75	**9,23**
Basic leucine zipper and W2 domain-containing protein 1	Q7L1Q6|BZW1_HUMAN	**2,28**	**4,64**	**6,62**
		0,00	0,00	**15,50**
Basigin	P35613|BASI_HUMAN	0,97	2,01	3,91
		2,77	0,00	6,98
B-cell receptor-associated protein 31	P51572|BAP31_HUMAN	3,55	**9,70**	**17,11**
		2,35	3,14	**27,77**
Beta-2-glycoprotein 1	P02749|APOH_HUMAN	**1,67**	**2,40**	**2,25**
		**2,08**	**2,13**	**3,05**
Beta-2-microglobulin	P61769|B2MG_HUMAN	**0,67**	0,99	1,07
		**1,84**	**1,65**	**2,40**
Beta-hexosaminidase subunit beta	P07686|HEXB_HUMAN	1,16	1,60	**2,44**
		**2,57**	**2,93**	**8,39**
Beta-lactamase-like protein 2	Q53H82|LACB2_HUMAN	1,56	2,70	2,79
		3,05	2,92	7,65
Biliverdin reductase A	P53004|BIEA_HUMAN	2,08	3,24	3,55
		1,80	2,49	7,05
BolA-like protein 2	Q9H3K6|BOLA2_HUMAN	25,64	31,47	22,00
		40,37	27,05	41,20
Brain acid soluble protein 1	P80723|BASP1_HUMAN	1,27	**2,38**	**4,40**
		2,07	2,00	**5,92**
Bridging integrator 2	Q9UBW5|BIN2_HUMAN	1,63	**3,82**	**5,18**
		3,66	5,34	16,48
Cadherin-11	P55287|CAD11_HUMAN	11,37	**15,60**	**8,91**
		6,99	6,88	**11,25**
Calcineurin subunit B type 1	P63098|CANB1_HUMAN	2,41	5,01	6,50
		5,95	6,63	17,10
	Q9BRF8|CPPED_HUMAN	1,51	2,37	2,41
Calcineurin-like phosphoesterase domain-containing protein 1		0,00	0,00	14,51
Calcium-binding protein 39	Q9Y376|CAB39_HUMAN	**1,46**	**2,47**	**3,61**
		**2,99**	**3,82**	**8,14**
Calcyclin-binding protein	Q9HB71|CYBP_HUMAN	**1,93**	**3,08**	**3,86**
		**3,05**	3,28	**8,15**
Calmodulin	P62158|CALM_HUMAN	**1,55**	0,97	1,38
		**2,67**	**2,69**	**8,40**
Calpastatin	P20810|ICAL_HUMAN	1,22	**2,41**	**4,37**
		**2,97**	2,62	**7,85**
Calreticulin	P27797|CALR_HUMAN	**1,59**	**2,37**	**2,01**
		**1,81**	**1,78**	**2,33**
Calumenin	O43852|CALU_HUMAN	3,04	5,30	6,02
		**4,05**	**3,23**	**7,63**
cAMP-dependent protein kinase catalytic subunit beta	P22694|KAPCB_HUMAN	1,60	**2,78**	3,68
		0,00	0,00	13,50
	P10644|KAP0_HUMAN	**1,89**	3,15	**4,51**
cAMP-dependent protein kinase type I-alpha regulatory subunit			4,26	**7,94**
Caprin-1	Q14444|CAPR1_HUMAN	1,62	**2,87**	**3,80**
		5,65	3,65	**10,02**
Capz-interacting protein	Q6JBY9|CPZIP_HUMAN	**1,81**	**3,54**	**5,27**
		2,59	2,07	**7,99**
Carbonic anhydrase 2	P00918|CAH2_HUMAN	1,35	**2,16**	**2,92**
		**2,47**	**2,30**	**6,90**
Carbonyl reductase [NADPH] 1	P16152|CBR1_HUMAN	**2,35**	**3,50**	**3,33**
		4,33	0,00	**10,90**
Carboxypeptidase M	P14384|CBPM_HUMAN	1,13	1,59	1,91
		1,64	**2,15**	**4,36**
Catalase	P04040|CATA_HUMAN	1,97	2,67	2,32
		2,34	2,28	**5,08**
Cathepsin B	P07858|CATB_HUMAN	**0,69**	**0,64**	0,62
		**1,54**	**1,60**	**2,24**
Cathepsin D	P07339|CATD_HUMAN	0,85	0,82	1,10
		**2,40**	**2,77**	**6,73**
Cathepsin H	P09668|CATH_HUMAN	**1,84**	**2,41**	**3,17**
		2,54	3,06	8,15
Cathepsin S	P25774|CATS_HUMAN	1,15	1,23	1,46
		**1,92**	**2,00**	**5,07**
Cathepsin Z	Q9UBR2|CATZ_HUMAN	1,10	1,11	1,49
		1,92	**1,80**	**4,54**
Cation-independent mannose-6-phosphate receptor	P11717|MPRI_HUMAN	1,52	**3,34**	**3,20**
		2,18	3,33	7,58
C-C motif chemokine 24	O00175|CCL24_HUMAN	1,67	2,48	2,36
		2,72	2,58	4,61
C-C motif chemokine 8	P80075|CCL8_HUMAN	**7,66**	**7,25**	13,45
CD166 antigen	Q13740|CD166_HUMAN	1,44	**2,26**	**2,83**
		1,75	2,42	**6,22**
CD44 antigen	P16070|CD44_HUMAN	1,48	**3,01**	**4,85**
		**2,86**	**3,45**	**9,49**
CD63 antigen	P08962|CD63_HUMAN	1,32	2,40	2,92
		2,16	**2,98**	**10,82**
CD9 antigen	P21926|CD9_HUMAN	1,47	1,43	2,15
		1,87	2,13	8,95
Cell division control protein 42 homolog	P60953|CDC42_HUMAN	2,34	4,75	5,35
		1,74	3,37	17,06

Cellular nucleic acid-binding protein	P62633|CNBP_HUMAN	2,51	**4,54**	**6,75**
		3,81	4,43	12,65
Cellular retinoic acid-binding protein 2	P29373|RABP2_HUMAN	**1,62**	**2,40**	**3,39**
		3,78	4,03	**14,60**
Charged multivesicular body protein 4a	Q9BY43|CHM4A_HUMAN	1,28	2,89	5,65
		2,20	1,89	7,10
Chitotriosidase-1	Q13231|CHIT1_HUMAN	0,69	0,78	0,74
		**1,52**	1,25	1,21
Chloride intracellular channel protein 1	O00299|CLIC1_HUMAN	**2,31**	**3,87**	**4,70**
		**3,06**	**3,56**	**8,37**
Chloride intracellular channel protein 4	Q9Y696|CLIC4_HUMAN	2,00	3,31	4,15
		3,87	3,00	**11,16**
Chromobox protein homolog 3	Q13185|CBX3_HUMAN	1,23	2,93	4,00
		4,16	3,58	**11,18**
Citrate synthase, mitochondrial	O75390|CISY_HUMAN	**2,04**	**3,22**	**2,96**
		2,49	2,76	**4,97**
Clathrin heavy chain 1	Q00610|CLH1_HUMAN	**2,05**	**2,58**	**2,95**
		**2,88**	**2,73**	**7,78**
Clathrin light chain A	P09496|CLCA_HUMAN	2,83	**4,79**	**6,10**
		3,08	2,66	6,47
Clathrin light chain B	P09497|CLCB_HUMAN	**2,52**	**4,53**	**6,08**
		0,00	0,00	0,00
Cleavage and polyadenylation specificity factor subunit 5	O43809|CPSF5_HUMAN	2,47	3,95	6,34
		3,07	2,48	6,76
Coactosin-like protein	Q14019|COTL1_HUMAN	1,23	**2,01**	**2,64**
		**2,42**	**2,89**	**7,80**
Coagulation factor XIII A chain	P00488|F13A_HUMAN	**2,02**	**2,35**	**2,81**
Coatomer subunit delta	P48444|COPD_HUMAN	1,84	**3,26**	**3,81**
		2,52	2,73	**7,25**
Cofilin-1	P23528|COF1_HUMAN	**1,90**	**3,98**	**6,10**
		**2,86**	**4,76**	**15,67**
Cold shock domain-containing protein E1	O75534|CSDE1_HUMAN	2,08	**3,18**	3,00
Collagen alpha-1(I) chain	P02452|CO1A1_HUMAN	4,52	6,09	3,75
		**5,52**	5,81	**7,57**
Collagen alpha-1(V) chain	P20908|CO5A1_HUMAN	14,90	**19,99**	**12,86**
		26,97	19,93	31,20
Collagen alpha-1(XI) chain	P12107|COBA1_HUMAN	5,22	7,87	8,98
		11,00	8,63	13,78
Complement C1q subcomponent subunit B	P02746|C1QB_HUMAN	0,00	0,00	0,00
		2,31	0,00	**2,66**
Coronin-1A	P31146|COR1A_HUMAN	**2,42**	**3,86**	**4,47**
		2,56	**2,10**	**9,25**
Coronin-1B	Q9BR76|COR1B_HUMAN	**2,28**	**3,90**	**4,26**
		4,97	3,02	11,84
Coronin-1C	Q9ULV4|COR1C_HUMAN	**2,08**	**3,30**	**5,03**
		**3,56**	**3,58**	**8,83**
Cystatin-A	P01040|CYTA_HUMAN	2,21	**3,84**	**5,04**
		2,18	1,89	7,28
Cystatin-B	P04080|CYTB_HUMAN	1,45	**3,05**	**5,01**
		**2,93**	**3,08**	**10,39**
Cystatin-C	P01034|CYTC_HUMAN	0,88	1,00	0,91
		1,99	1,37	2,12
Cysteine and glycine-rich protein 1	P21291|CSRP1_HUMAN	**2,09**	**2,90**	**3,78**
		0,00	0,00	0,00
Cysteine-rich protein 1	P50238|CRIP1_HUMAN	4,14	7,98	**6,58**
Cytochrome b-245 light chain	P13498|CY24A_HUMAN	1,23	2,09	**2,95**
		2,41	2,92	8,48
Cytochrome c	P99999|CYC_HUMAN	**1,93**	**4,31**	**7,25**
		3,29	**3,32**	**9,74**
Cytoplasmic dynein 1 intermediate chain 2	Q13409|DC1I2_HUMAN	2,25	2,72	3,36
		6,73	6,18	12,39
Cytosol aminopeptidase	P28838|AMPL_HUMAN	2,02	**3,47**	**3,77**
		**2,49**	3,52	**11,04**
Cytosolic non-specific dipeptidase	Q96KP4|CNDP2_HUMAN	1,99	**3,63**	**4,38**
		1,90	2,27	**6,24**
Deoxyribonucleoside 5'-monophosphate N-glycosidase	O43598|RCL_HUMAN			
		6,87	5,65	**14,67**
Destrin	P60981|DEST_HUMAN	**7,97**	**10,30**	**10,46**
		8,00	**6,60**	**17,52**
Dihydrolipoyllysine-residue succinyltransferase component of 2-oxoglutarate dehydrogenase complex, mitochondrial	P36957|ODO2_HUMAN	2,54	3,79	3,30
		3,35	4,47	5,22
Dihydropyrimidinase-related protein 2	Q16555|DPYL2_HUMAN	**1,54**	**2,49**	**2,50**
		0,00	0,00	4,87
Dipeptidyl-peptidase 1	P53634|CATC_HUMAN	2,22	2,64	2,70
		3,85	4,10	9,58
Dipeptidyl-peptidase 3	Q9NY33|DPP3_HUMAN	1,40	**2,10**	**2,38**
		2,42	1,74	**4,96**
Disabled homolog 2	P98082|DAB2_HUMAN	**2,49**	4,15	**6,13**
		**3,67**	**2,98**	6,82
DNA-(apurinic or apyrimidinic site) lyase	P27695|APEX1_HUMAN	1,46	2,48	**2,69**
		**2,79**	**2,39**	**5,29**
DnaJ homolog subfamily B member 1	P25685|DNJB1_HUMAN	**2,65**	**4,37**	**5,33**
		4,55	4,60	9,71
DnaJ homolog subfamily C member 8	O75937|DNJC8_HUMAN	2,02	**3,40**	4,52
		4,22	4,41	**12,73**
Drebrin-like protein	Q9UJU6|DBNL_HUMAN	2,06	**3,85**	**4,34**
		**4,22**	**3,95**	**11,00**
Dual specificity mitogen-activated protein kinase kinase 1	Q02750|MP2K1_HUMAN	**2,04**	**4,54**	**5,71**
		0,00	0,00	44,13
Dynactin subunit 2	Q13561|DCTN2_HUMAN	1,30	2,63	**3,93**
		3,71	6,30	9,37
Dystroglycan	Q14118|DAG1_HUMAN	2,02	2,72	2,84
		1,97	1,86	4,30
EF-hand domain-containing protein D2	Q96C19|EFHD2_HUMAN	**2,07**	**4,51**	**6,78**
		**4,61**	**5,35**	**17,49**
Elongation factor 1-alpha 1	P68104|EF1A1_HUMAN	**2,13**	**4,46**	**4,63**
Elongation factor 1-beta	P24534|EF1B_HUMAN	**2,42**	**4,77**	**6,34**
		**5,67**	**6,29**	**15,94**
Elongation factor 1-gamma	P26641|EF1G_HUMAN	2,92	**4,91**	**5,00**
		**3,78**	**4,32**	**12,36**
Elongation factor 2	P13639|EF2_HUMAN	**2,67**	**4,11**	**4,12**
		**3,24**	**3,55**	**11,85**
EMILIN-2	Q9BXX0|EMIL2_HUMAN	**0,60**	**0,57**	0,65
		1,16	1,24	1,53
Ena/VASP-like protein	Q9UI08|EVL_HUMAN	1,18	**2,29**	**3,31**
		0,00	0,00	0,00
Endoplasmic reticulum protein ERp29	P30040|ERP29_HUMAN	**2,80**	**3,60**	4,39
		**3,06**	**3,12**	**6,75**
Endoplasmin	P14625|ENPL_HUMAN	**1,89**	**3,05**	**2,37**
		**2,76**	**2,98**	**3,99**
Endothelial differentiation-related factor 1	O60869|EDF1_HUMAN	2,74	3,80	**6,02**
		0,00	0,00	0,00
Epididymal secretory protein E1	P61916|NPC2_HUMAN	**1,76**	**2,70**	**3,16**
		**3,12**	**3,30**	**8,52**
ES1 protein homolog, mitochondrial	P30042|ES1_HUMAN	1,17	2,37	**2,60**

		4,13	2,96	5,79
Eukaryotic initiation factor 4A-I	P60842|IF4A1_HUMAN	**2,39**<br>6,43	**4,02**<br>**5,45**	**4,88**<br>**7,69**
Eukaryotic translation initiation factor 1A, X-chromosomal	P47813|IF1AX_HUMAN	1,90<br>0,00	3,83<br>0,00	5,01<br>10,45
Eukaryotic translation initiation factor 2 subunit 2-like protein PE=1 SV=1	A6NK07|IF2BL_HUMAN	1,90<br>2,07	3,52<br>1,56	**4,97**<br>4,35
Eukaryotic translation initiation factor 2A	Q9BY44|EIF2A_HUMAN	1,99<br>0,00	3,52<br>0,00	9,03<br>8,23
Eukaryotic translation initiation factor 3 subunit A	Q14152|EIF3A_HUMAN	**1,82**<br>2,78	**3,25**<br>0,00	**3,89**<br>**6,19**
Eukaryotic translation initiation factor 3 subunit C	Q99613|EIF3C_HUMAN	1,34<br>2,66	2,33<br>2,80	**3,39**<br>8,33
Eukaryotic translation initiation factor 3 subunit J	O75822|EIF3J_HUMAN	2,40<br>3,83	**5,18**<br>4,25	**7,67**<br>13,04
Eukaryotic translation initiation factor 4 gamma 1	Q04637|IF4G1_HUMAN	**1,70**<br>3,32	**3,50**<br>2,41	**4,30**<br>**9,95**
Eukaryotic translation initiation factor 4B	P23588|IF4B_HUMAN	1,98<br>0,00	**3,64**<br>0,00	**4,63**<br>**7,44**
Eukaryotic translation initiation factor 4H	Q15056|IF4H_HUMAN	2,17<br>2,73	3,63<br>**2,35**	4,19<br>**7,44**
Eukaryotic translation initiation factor 5	P55010|IF5_HUMAN	**1,98**<br>3,95	**4,13**<br>0,00	**5,56**<br>6,71
Eukaryotic translation initiation factor 5A-1	P63241|IF5A1_HUMAN	1,64<br>**2,74**	**3,25**<br>**3,03**	**4,29**<br>**9,44**
Eukaryotic translation initiation factor 5B	O60841|IF2P_HUMAN	1,55	**2,48**	**5,17**
Extracellular matrix protein 1	Q16610|ECM1_HUMAN	0,76<br>**2,23**	1,04<br>1,73	1,19<br>**2,31**
Ezrin	P15311|EZRI_HUMAN	**1,59**<br>**3,29**	**2,89**<br>**3,57**	**3,49**<br>**9,87**
F-actin-capping protein subunit alpha-1	P52907|CAZA1_HUMAN	3,31<br>4,10	3,79<br>6,28	4,15<br>16,91
F-actin-capping protein subunit beta	P47756|CAPZB_HUMAN	**2,15**<br>5,85	**3,29**<br>5,86	**3,74**<br>9,84
Far upstream element-binding protein 1	Q96AE4|FUBP1_HUMAN	**2,06**<br>4,74	**4,18**<br>5,30	**5,07**<br>15,60
Far upstream element-binding protein 2	Q92945|FUBP2_HUMAN	**1,95**<br>6,24	**3,75**<br>**7,45**	**4,70**<br>**19,95**
Farnesyl pyrophosphate synthetase	P14324|FPPS_HUMAN	**1,33**<br>1,79	**2,23**<br>2,42	**2,48**<br>**7,30**
Fascin	Q16658|FSCN1_HUMAN	**1,45**<br>3,51	**2,17**<br>3,82	**2,23**<br>**9,71**
Fatty acid synthase	P49327|FAS_HUMAN	**2,34**<br>0,00	**3,29**<br>0,00	**3,25**<br>8,40
Fatty acid-binding protein, adipocyte	P15090|FABP4_HUMAN	2,06<br>3,66	2,56<br>**3,70**	2,87<br>10,01
Fatty acid-binding protein, epidermal	Q01469|FABP5_HUMAN	2,14<br>**3,07**	**3,20**<br>**3,28**	**4,29**<br>**11,71**
Fatty acid-binding protein, heart	P05413|FABPH_HUMAN	2,68<br>**5,99**	**3,59**<br>**5,47**	**4,52**<br>**14,12**
Fermitin family homolog 3	Q86UX7|URP2_HUMAN	**2,03**<br>**4,27**	**3,02**<br>**4,47**	**3,73**<br>**12,95**
Ferritin heavy chain	P02794|FRIH_HUMAN	1,96<br>**2,75**	3,44<br>**2,56**	3,67<br>**6,16**
Ferritin light chain	P02792|FRIL_HUMAN	1,63<br>**1,95**	**2,87**<br>**2,35**	**3,76**<br>**5,54**
Fibroleukin	Q14314|FGL2_HUMAN	**1,60**<br>**3,04**	**1,91**<br>**2,55**	**2,27**<br>**6,29**
Fibronectin	P02751|FINC_HUMAN	**13,33**<br>**15,53**	**10,90**<br>**12,03**	**7,58**<br>**17,89**
Filamin-A	P21333|FLNA_HUMAN	**1,46**<br>**2,66**	**2,15**<br>**2,42**	**2,41**<br>**6,60**
Filamin-B	O75369|FLNB_HUMAN	 3,49	 4,21	 **5,60**
FK506-binding protein 3	Q00688|FKBP3_HUMAN	**2,05**<br>**6,42**	**4,48**<br>**6,30**	**7,44**<br>**18,59**
FK506-binding protein 4	Q02790|FKBP4_HUMAN	1,98	**3,75**	**4,12**
FK506-binding protein 5	Q13451|FKBP5_HUMAN	2,14<br>**4,99**	**4,19**<br>7,50	**5,41**<br>**15,83**
Follistatin-related protein 1	Q12841|FSTL1_HUMAN	**5,72**<br>**14,69**	**9,84**<br>11,90	**7,32**<br>**22,88**
Fructose-1,6-bisphosphatase 1	P09467|F16P1_HUMAN	1,31<br>**2,08**	**2,10**<br>**2,17**	**2,33**<br>**5,71**
Fructose-bisphosphate aldolase A	P04075|ALDOA_HUMAN	**1,55**<br>**2,21**	**2,22**<br>**2,54**	**2,35**<br>**6,67**
Fumarate hydratase, mitochondrial	P07954|FUMH_HUMAN	**1,78**<br>2,28	**2,40**<br>0,00	**2,63**<br>**4,69**
Galectin-1	P09382|LEG1_HUMAN	1,50<br>**2,69**	**3,63**<br>**3,78**	**6,19**<br>**11,51**
Galectin-3	P17931|LEG3_HUMAN	**1,37**<br>**3,17**	**2,16**<br>**2,90**	**2,67**<br>**7,13**
Galectin-3-binding protein	Q08380|LG3BP_HUMAN	0,67	0,75	**0,66**
Gamma-aminobutyric acid receptor-associated protein-like 2	P60520|GBRL2_HUMAN	2,56<br>1,85	5,90<br>1,90	**5,93**<br>6,84
Gamma-interferon-inducible lysosomal thiol reductase	P13284|GILT_HUMAN	1,96<br>8,88	3,21<br>4,91	2,11<br>14,20
Ganglioside GM2 activator	P17900|SAP3_HUMAN	0,70<br>**2,33**	0,99<br>**2,21**	1,30<br>**4,15**
Gelsolin	P06396|GELS_HUMAN	**1,39**<br>**2,27**	**2,35**<br>**2,46**	**2,90**<br>**7,03**
Glia maturation factor gamma	O60234|GMFG_HUMAN	**1,93**<br>**3,53**	**3,52**<br>**4,12**	**4,81**<br>**11,84**
Glucose-6-phosphate 1-dehydrogenase	P11413|G6PD_HUMAN	**3,03**<br>**3,51**	**6,23**<br>**3,91**	**7,05**<br>**12,92**
Glucose-6-phosphate isomerase	P06744|G6PI_HUMAN	**1,42**<br>**2,12**	**2,02**<br>**2,38**	**2,39**<br>**5,78**
Glucosidase 2 subunit beta	P14314|GLU2B_HUMAN	1,44	**2,96**	**2,71**
Glutaminyl-peptide cyclotransferase	Q16769|QPCT_HUMAN	0,90<br>**1,47**	0,95<br>1,25	0,84<br>1,35
Glutaredoxin-1	P35754|GLRX1_HUMAN	0,00<br>3,28	0,00<br>4,41	0,00<br>**14,45**
Glutathione reductase, mitochondrial	P00390|GSHR_HUMAN	1,34<br>2,06	**1,76**<br>**1,71**	**1,95**<br>**5,95**
Glutathione S-transferase omega-1	P78417|GSTO1_HUMAN	**1,57**<br>**2,54**	**2,76**<br>**2,93**	**3,65**<br>**8,30**
Glutathione S-transferase P	P09211|GSTP1_HUMAN	**1,58**<br>2,99	**2,74**<br>3,25	**3,57**<br>**8,37**
Glutathione synthetase	P48637|GSHB_HUMAN	1,29	**2,19**	**2,07**
Glyceraldehyde-3-phosphate dehydrogenase	P04406|G3P_HUMAN	1,21<br>**3,27**	**3,11**<br>**4,61**	**4,82**<br>**16,96**
Glycogen phosphorylase, liver form	P06737|PYGL_HUMAN	**1,93**<br>2,80	**3,36**<br>2,80	**3,04**<br>8,02
Glycylpeptide N-tetradecanoyltransferase 1	P30419|NMT1_HUMAN	1,40<br>2,72	1,90<br>2,72	**3,36**<br>8,08

Glycyl-tRNA synthetase	P41250|SYG_HUMAN			
		4,96	3,79	**13,78**
Glyoxalase domain-containing protein 4	Q9HC38|GLOD4_HUMAN	1,93	2,91	3,57
		3,43	3,74	**10,25**
G-protein coupled receptor 120	Q5NUL3|GP120_HUMAN	29,47	28,32	18,90
		51,57	33,67	79,58
Granulins	P28799|GRN_HUMAN	0,87	1,05	1,28
		3,03	1,85	3,47
Growth factor receptor-bound protein 2	P62993|GRB2_HUMAN	**1,93**	**3,74**	**4,80**
		**2,99**	**3,11**	**8,57**
GTP-binding nuclear protein Ran	P62826|RAN_HUMAN	2,29	**4,71**	**6,04**
		3,02	3,66	12,30
Guanine nucleotide-binding protein G(I)/G(S)/G(T) subunit beta-2	P62879|GBB2_HUMAN	**2,03**	**2,21**	**2,52**
		0,00	0,00	0,00
Guanine nucleotide-binding protein subunit beta-2-like 1	P63244|GBLP_HUMAN	**1,70**	**3,47**	**4,32**
		2,77	2,81	**8,82**
Haptoglobin	P00738|HPT_HUMAN	**1,57**	**1,92**	**1,34**
		**3,22**	**2,74**	**3,80**
Heat shock 70 kDa protein 1	P08107|HSP71_HUMAN	**1,66**	**3,10**	**3,43**
		**2,35**	**2,54**	**7,34**
Heat shock 70 kDa protein 4	P34932|HSP74_HUMAN	**1,75**	**2,60**	**2,95**
		**2,33**	**2,23**	**6,31**
Heat shock cognate 71 kDa protein	P11142|HSP7C_HUMAN	**1,80**	**2,90**	**3,47**
		**3,12**	**3,29**	**8,60**
Heat shock protein 105 kDa	Q92598|HS105_HUMAN	2,13	**3,04**	**3,74**
		2,29	3,05	**8,63**
Heat shock protein beta-1	P04792|HSPB1_HUMAN	**1,74**	**3,12**	**4,06**
		2,84	3,20	**8,85**
Heat shock protein HSP 90-alpha	P07900|HS90A_HUMAN	**2,21**	**3,60**	**3,99**
		**2,86**	**3,29**	**9,46**
Heat shock protein HSP 90-beta	P08238|HS90B_HUMAN	**1,75**	**3,19**	**3,64**
		**3,09**	**3,96**	**12,91**
Hemagglutinin	P03435|HEMA_I75A3	14,29	**16,56**	**18,87**
		**14,01**	**18,51**	**59,70**
Hematological and neurological expressed 1 protein	Q9UK76|HN1_HUMAN	1,69	3,73	5,10
		6,03	5,84	16,33
Hematopoietic lineage cell-specific protein	P14317|HCLS1_HUMAN	**2,04**	**3,72**	**5,80**
		**4,22**	**4,53**	**12,84**
Heme-binding protein 1	Q9NRV9|HEBP1_HUMAN	1,65	**4,02**	**5,13**
		0,00	0,00	24,57
Hemoglobin subunit alpha	P69905|HBA_HUMAN	13,02	**14,83**	8,73
		80,53	52,69	75,94
Hemoglobin subunit beta	P68871|HBB_HUMAN	18,08	19,43	11,51
		23,58	16,16	24,02
Hemopexin	P02790|HEMO_HUMAN	1,49	**1,50**	1,08
		**3,86**	**3,65**	**4,44**
Hepatitis A virus cellular receptor 2	Q8TDQ0|TIMD3_HUMAN	2,34	**3,05**	3,19
		3,15	3,24	6,73
Hepatoma-derived growth factor	P51858|HDGF_HUMAN	**1,50**	**3,19**	**4,96**
		3,04	2,60	6,46
Heterogeneous nuclear ribonucleoprotein A/B	Q99729|ROAA_HUMAN	6,47	10,38	7,74
		3,29	4,32	9,14
Heterogeneous nuclear ribonucleoprotein A3	P51991|ROA3_HUMAN	2,32	4,64	7,73
		3,73	4,09	8,68
Heterogeneous nuclear ribonucleoprotein D0	Q14103|HNRPD_HUMAN	1,65	3,35	**4,34**
		3,71	3,09	11,79
Heterogeneous nuclear ribonucleoprotein F	P52597|HNRPF_HUMAN	1,49	**2,82**	**2,87**
		2,15	2,80	6,99
Heterogeneous nuclear ribonucleoprotein G	P38159|HNRPG_HUMAN	**1,75**	**3,36**	**5,28**
		3,54	3,73	7,98
Heterogeneous nuclear ribonucleoprotein H	P31943|HNRH1_HUMAN	1,47	**3,43**	**3,59**
		0,00	0,00	9,12
Heterogeneous nuclear ribonucleoprotein K	P61978|HNRPK_HUMAN	1,86	**3,20**	**4,03**
		**6,41**	**5,18**	**11,93**
Heterogeneous nuclear ribonucleoprotein L	P14866|HNRPL_HUMAN	2,96	**6,39**	7,01
		0,00	0,00	33,85
Heterogeneous nuclear ribonucleoprotein M	P52272|HNRPM_HUMAN	2,47	4,05	4,79
		5,48	5,06	7,27
Heterogeneous nuclear ribonucleoprotein Q	O60506|HNRPQ_HUMAN	2,39	4,57	4,47
		5,24	6,00	11,37
Heterogeneous nuclear ribonucleoprotein R	O43390|HNRPR_HUMAN	3,78	6,15	5,44
		0,00	0,00	25,96
Heterogeneous nuclear ribonucleoprotein U	Q00839|HNRPU_HUMAN	**1,66**	**2,84**	**3,76**
		3,58	2,67	**8,62**
Heterogeneous nuclear ribonucleoproteins A2/B1	P22626|ROA2_HUMAN	**1,96**	**3,35**	**4,77**
		**3,44**	**3,46**	**7,63**
Heterogeneous nuclear ribonucleoproteins C1/C2	P07910|HNRPC_HUMAN	1,77	**3,36**	**4,46**
		2,81	2,45	6,04
Hexokinase-3	P52790|HXK3_HUMAN	1,48	2,45	4,14
		2,11	1,79	**6,93**
High mobility group protein B1	P09429|HMGB1_HUMAN	1,74	**5,56**	**10,52**
		2,38		6,97
High mobility group protein B2	P26583|HMGB2_HUMAN	1,13	4,03	6,46
		4,66	6,08	**15,97**
Histidine triad nucleotide-binding protein 1	P49773|HINT1_HUMAN	2,08	**4,21**	**6,43**
		**4,91**	**5,30**	**17,88**
Histone H1.3	P16402|H13_HUMAN	1,24	**2,39**	**3,60**
Histone H2A.x	P16104|H2AX_HUMAN	1,03	2,59	**5,30**
Histone H2B type 1-M	Q99879|H2B1M_HUMAN			
		**5,21**	4,57	**11,72**
Histone H2B type F-S	P57053|H2BFS_HUMAN	1,08	**2,15**	**3,01**
Histone H3.1t	Q16695|H31T_HUMAN			
		**3,91**	**2,81**	**6,64**
Histone H3.2	Q71DI3|H32_HUMAN	1,56	**2,34**	**2,88**
Histone H4	P62805|H4_HUMAN	1,81	2,59	2,84
		**5,19**	**3,92**	**8,97**
Histone-binding protein RBBP4	Q09028|RBBP4_HUMAN	2,16	3,17	**2,85**
HLA class I histocompatibility antigen, A-2 alpha chain	P01892|1A02_HUMAN	1,01	**1,68**	**1,89**
HLA class II histocompatibility antigen, DP alpha 1 chain	P20036|DPA1_HUMAN	1,86	3,21	2,39
		2,95	3,19	7,65
HLA class II histocompatibility antigen, DR alpha chain	P01903|2DRA_HUMAN	1,37	1,95	**2,72**
		1,62	**1,78**	**3,54**
HLA class II histocompatibility antigen, DR-1 beta chain	P01912|HB2B_HUMAN	1,32	1,37	1,25
		2,57	2,70	**5,93**
Hsc70-interacting protein	P50502|F10A1_HUMAN	**2,59**	**5,48**	**7,71**
		**3,27**	**3,19**	**11,50**
Hsp90 co-chaperone Cdc37	Q16543|CDC37_HUMAN	**1,96**	4,16	5,93
		**3,54**	**3,91**	**14,17**
Huntingtin-interacting protein K	Q9NX55|HYPK_HUMAN	3,28	8,08	12,98
		4,33	6,90	22,20
Importin subunit beta-1	Q14974|IMB1_HUMAN	2,05	3,15	3,36
		3,31	2,64	**9,20**
Integrin alpha-L	P20701|ITAL_HUMAN	1,25	2,04	1,92

		2,06	2,95	8,51
Integrin alpha-M	P11215|ITAM_HUMAN	0,74	0,99	1,15
		1,67	1,97	5,80
Integrin beta-2	P05107|ITB2_HUMAN	0,95	1,24	**1,58**
		**1,88**	**2,16**	**4,80**
Interferon-induced 17 kDa protein	P05161|UCRP_HUMAN	1,47	**2,82**	**4,06**
Interferon-induced guanylate-binding protein 1	P32455|GBP1_HUMAN	2,21	**4,11**	**5,20**
Interleukin enhancer-binding factor 3	Q12906|ILF3_HUMAN	**1,81**	**2,93**	**3,48**
		0,00	0,00	0,00
Interleukin-1 receptor antagonist protein	P18510|IL1RA_HUMAN	1,08	1,34	1,47
		**2,11**	1,83	3,29
Isocitrate dehydrogenase [NADP] cytoplasmic	O75874|IDHC_HUMAN	**2,11**	**3,13**	**3,51**
		**2,78**	**3,50**	**8,87**
Junctional adhesion molecule-like	Q86YT9|JAML1_HUMAN	1,36	1,52	1,66
			4,19	5,55
Keratin, type I cytoskeletal 10	P13645|K1C10_HUMAN	1,38	1,61	1,39
		2,01	1,01	1,56
Keratin, type I cytoskeletal 17	Q04695|K1C17_HUMAN	6,06	9,62	8,52
		**14,21**	**11,84**	**22,60**
Keratin, type I cytoskeletal 9	P35527|K1C9_HUMAN	1,47	1,33	1,13
		2,54	2,21	2,81
Keratin, type II cytoskeletal 1	P04264|K2C1_HUMAN	**1,60**	1,34	1,26
		**2,87**	**2,10**	2,40
Keratin, type II cytoskeletal 8	P05787|K2C8_HUMAN	**15,52**	**21,52**	15,78
		17,84	15,20	34,13
KH domain-containing, RNA-binding, signal transduction-	Q07666|KHDR1_HUMAN	1,72	2,52	3,67
associated protein 1		6,82	6,20	13,33
Kunitz-type protease inhibitor 1	O43278|SPIT1_HUMAN	1,78	**2,73**	**3,01**
		2,32	2,85	5,25
Kynureninase	Q16719|KYNU_HUMAN	**1,89**	**2,26**	**3,17**
		2,01	**2,68**	**8,80**
Lactoylglutathione lyase	Q04760|LGUL_HUMAN			
		2,90	**2,46**	8,38
Lamin-A/C	P02545|LMNA_HUMAN	**1,57**	**2,60**	**3,60**
		**2,92**	**2,65**	**7,29**
Lamina-associated polypeptide 2, isoform alpha	P42166|LAP2A_HUMAN	1,66	2,75	**3,88**
		5,45	4,58	9,24
Lamin-B1	P20700|LMNB1_HUMAN	1,41	**2,97**	**3,97**
		0,00	0,00	**7,96**
Lamin-B2	Q03252|LMNB2_HUMAN	9,93	17,60	18,01
		0,00	0,00	9,26
Leucine-rich repeat flightless-interacting protein 1	Q32MZ4|LRRF1_HUMAN	**1,57**	**3,41**	**4,90**
		**3,25**	**2,68**	**6,33**
Leukocyte common antigen	P08575|CD45_HUMAN	**1,48**	**2,12**	**2,46**
		0,00	0,00	**7,13**
Leukocyte elastase inhibitor	P30740|ILEU_HUMAN	2,10	3,13	3,26
		**2,63**	**2,54**	**6,43**
	Q8NHJ6|LIRB4_HUMAN	1,60	3,04	2,78
Leukocyte immunoglobulin-like receptor subfamily B member 4		3,53	3,30	8,11
Leukosialin	P16150|LEUK_HUMAN	0,72	**1,86**	2,23
Leukotriene A-4 hydrolase	P09960|LKHA4_HUMAN	**1,33**	**2,14**	**2,52**
		**2,25**	**2,26**	**6,57**
LIM and SH3 domain protein 1	Q14847|LASP1_HUMAN	1,78	**4,10**	**5,65**
		**2,96**	**3,28**	**10,46**
LIM domain and actin-binding protein 1	Q9UHB6|LIMA1_HUMAN	2,56	4,45	**6,40**
		0,00	0,00	0,00
L-lactate dehydrogenase A chain	P00338|LDHA_HUMAN	2,03	**3,30**	**3,60**
		**2,83**	**2,85**	**7,61**
L-lactate dehydrogenase B chain	P07195|LDHB_HUMAN	**1,75**	**2,67**	**2,63**
		**3,07**	**2,84**	**7,16**
Lupus La protein	P05455|LA_HUMAN	**1,67**	**3,41**	**4,85**
		4,02	3,84	**8,75**
Lymphocyte cytosolic protein 2	Q13094|LCP2_HUMAN	1,79	**3,30**	**5,27**
Lymphocyte-specific protein 1	P33241|LSP1_HUMAN	**1,62**	**3,29**	**5,05**
		**3,36**	**3,34**	**8,44**
Lysosomal alpha-glucosidase	P10253|LYAG_HUMAN	0,00	0,00	0,00
		5,90	**6,02**	**16,78**
Lysosomal alpha-mannosidase	O00754|MA2B1_HUMAN	0,94	1,09	1,37
		**2,39**	1,95	**5,15**
Lysozyme C	P61626|LYSC_HUMAN	**0,53**	**0,56**	0,79
		**1,63**	1,14	**1,56**
Macrophage colony-stimulating factor 1	P09603|CSF1_HUMAN	1,29	1,75	2,22
		1,96	2,16	5,05
Macrophage mannose receptor 1-like protein 1	Q5VSK2|MRC1L_HUMAN	1,07	**1,56**	**2,43**
		**1,94**	**3,41**	**10,00**
Macrophage metalloelastase	P39900|MMP12_HUMAN	1,12	1,16	1,44
		**3,15**	**2,30**	**3,99**
Macrophage migration inhibitory factor	P14174|MIF_HUMAN			
		2,12	2,94	**8,27**
Macrophage scavenger receptor types I and II	P21757|MSRE_HUMAN	1,53	**3,29**	**4,67**
		2,55	3,04	**10,00**
Macrophage-capping protein	P40121|CAPG_HUMAN	**1,94**	**3,48**	**4,32**
		**3,51**	**3,87**	**12,18**
Malate dehydrogenase, cytoplasmic	P40925|MDHC_HUMAN	**1,64**	**2,26**	**2,71**
		**2,43**	**2,61**	**7,03**
Malate dehydrogenase, mitochondrial	P40926|MDHM_HUMAN	**1,41**	**2,33**	**2,56**
		**2,40**	**2,12**	**4,55**
Matrilysin	P09237|MMP7_HUMAN			
		**3,02**	**3,00**	3,96
Matrix metalloproteinase-9	P14780|MMP9_HUMAN	**0,55**	**0,53**	**0,46**
		**1,31**	**1,24**	**1,46**
Matrix protein 1	Q9EA40|M1_I77A4	**12,21**	**20,69**	**33,79**
		8,46	9,57	**32,50**
Metalloproteinase inhibitor 2	P16035|TIMP2_HUMAN	0,91	**1,33**	**1,50**
		**2,68**	**2,18**	**2,96**
Microtubule-associated protein 4	P27816|MAP4_HUMAN	**1,77**	**4,45**	**6,53**
		**4,14**	**2,87**	**10,77**
Microtubule-associated protein RP/EB family member 1	Q15691|MARE1_HUMAN	**2,18**	**3,58**	**4,93**
		**3,23**	**3,66**	**10,02**
Mitogen-activated protein kinase 1	P28482|MK01_HUMAN	**1,87**	3,24	**4,20**
Mitogen-activated protein kinase 14	Q16539|MK14_HUMAN	4,97	**6,96**	**6,71**
Moesin	P26038|MOES_HUMAN	**1,46**	**2,32**	**2,90**
		**2,59**	**2,78**	**7,55**
Monocyte differentiation antigen CD14	P08571|CD14_HUMAN	0,96	**1,51**	**1,45**
Mucin-16	Q8WXI7|MUC16_HUMAN	122,87	128,80	52,52
		6,32	4,69	10,34
Myosin light polypeptide 6	P60660|MYL6_HUMAN	**1,78**	**3,20**	**3,85**
		**3,32**	**3,62**	**9,53**
Myosin regulatory light chain 12A	P19105|ML12A_HUMAN	2,29	3,70	5,07
		**3,66**	**3,55**	**10,83**
Myosin-9	P35579|MYH9_HUMAN	**1,63**	**2,67**	**3,76**
		**3,19**	**2,69**	**7,81**

Myotrophin	P58546|MTPN_HUMAN	2,36	4,19	5,70
		0,00	0,00	**12,77**
Myristoylated alanine-rich C-kinase substrate	P29966|MARCS_HUMAN	1,98	3,89	**5,88**
		2,72	3,31	**11,03**
N-acetyl-D-glucosamine kinase	Q9UJ70|NAGK_HUMAN	1,67	2,72	**3,70**
		1,91	2,00	**6,17**
N-acetylglucosamine-6-sulfatase	P15586|GNS_HUMAN	2,12	3,75	**5,73**
		0,00	0,00	0,00
NEDD8	Q15843|NEDD8_HUMAN	3,01	6,30	5,12
		12,50	13,26	**32,27**
Nestin	P48681|NEST_HUMAN	2,50	**3,78**	**4,18**
		2,70	3,12	4,75
Neuraminidase	Q09105|NRAM_I76AB	6,95	12,26	14,74
		0,00	8,08	23,93
Neuroblast differentiation-associated protein AHNAK	Q09666|AHNK_HUMAN	**1,74**	**4,45**	**7,35**
		**3,78**	**3,76**	**12,64**
Neurogranin	Q92686|NEUG_HUMAN	4,79	9,74	10,06
		0,00	0,00	9,74
Neuropilin-1	O14786|NRP1_HUMAN	1,00	1,10	1,10
		1,72	1,72	**3,12**
Neutral alpha-glucosidase AB	Q14697|GANAB_HUMAN	**1,86**	**2,83**	**2,31**
		0,00	0,00	5,38
Neutrophil cytosol factor 2	P19878|NCF2_HUMAN	**2,09**	**3,21**	**3,97**
		3,45	3,93	10,48
Niban-like protein 1	Q96TA1|NIBL1_HUMAN	**1,80**	**3,35**	**3,96**
		4,60	5,83	**16,92**
Non-POU domain-containing octamer-binding protein	Q15233|NONO_HUMAN	**2,36**	**3,81**	**4,21**
		4,50	2,69	**7,42**
Non-specific lipid-transfer protein	P22307|NLTP_HUMAN	1,69	**3,29**	**4,80**
		0,00	0,00	0,00
Non-structural protein 1	Q463W9|NS1_I72A3	**5,55**	**13,52**	**16,92**
		9,39	**13,80**	**49,25**
Nuclear migration protein nudC	Q9Y266|NUDC_HUMAN	2,87	**4,12**	**5,01**
		0,00	0,00	**15,22**
Nucleobindin-1	Q02818|NUCB1_HUMAN	0,89	1,25	1,41
		**2,04**	**1,63**	**3,13**
Nucleolin	P19338|NUCL_HUMAN	1,15	**2,43**	**4,67**
		**2,44**	**2,18**	**6,60**
Nucleophosmin	P06748|NPM_HUMAN	1,33	**2,30**	**3,67**
		**2,54**	**2,26**	**5,13**
Nucleoprotein	P06827|NCAP_I72A2	**9,79**	**24,90**	**32,38**
		**13,06**	**18,96**	**78,64**
Nucleoside diphosphate kinase A	P15531|NDKA_HUMAN	**2,36**	**3,76**	**4,56**
		0,00	4,47	13,23
Nucleoside diphosphate kinase B	P22392|NDKB_HUMAN			
		**4,80**	4,59	**11,30**
Nucleosome assembly protein 1-like 4	Q99733|NP1L4_HUMAN	3,25	6,48	10,91
		11,39	9,45	26,10
Obg-like ATPase 1	Q9NTK5|OLA1_HUMAN	1,93	3,20	**3,78**
		0,00	0,00	0,00
Osteoclast-stimulating factor 1	Q92882|OSTF1_HUMAN	1,36	**2,55**	**3,14**
		0,00	0,00	7,92
Osteopontin	P10451|OSTP_HUMAN	0,62	0,75	0,95
		**1,99**	1,24	**2,12**
Palmitoyl-protein thioesterase 1	P50897|PPT1_HUMAN	0,83	1,17	1,56
		2,16	4,89	12,34
Parathymosin	P20962|PTMS_HUMAN	1,43	**2,44**	**3,85**
		2,68	1,68	**6,76**
PDZ and LIM domain protein 5	Q96HC4|PDLI5_HUMAN	1,45	4,39	**6,28**
		0,00	0,00	48,77
Pentraxin-related protein PTX3	P26022|PTX3_HUMAN	1,86	1,43	1,35
		1,75	**1,67**	2,41
Peptidyl-prolyl cis-trans isomerase A	P62937|PPIA_HUMAN	**1,89**	**2,95**	**3,74**
		**3,27**	**3,64**	**10,96**
Peptidyl-prolyl cis-trans isomerase B	P23284|PPIB_HUMAN	1,29	**1,94**	**1,73**
		**2,25**	**2,17**	**3,00**
Peptidyl-prolyl cis-trans isomerase, mitochondrial	P30405|PPIF_HUMAN	**1,69**	**3,08**	**3,98**
		**4,43**	**4,79**	**8,50**
Perilipin-3	O60664|PLIN3_HUMAN	2,07	**5,59**	**9,97**
Peroxiredoxin-1	Q06830|PRDX1_HUMAN	1,76	**2,98**	**4,02**
		**2,96**	**3,29**	**9,32**
Peroxiredoxin-2	P32119|PRDX2_HUMAN	**1,95**	**4,00**	**5,55**
		5,65	6,00	13,87
Peroxiredoxin-6	P30041|PRDX6_HUMAN	2,16	3,81	**5,20**
		5,32	6,77	17,75
PEST proteolytic signal-containing nuclear protein	Q8WW12|PCNP_HUMAN	1,68	**4,32**	5,00
Phosphatidylethanolamine-binding protein 1	P30086|PEBP1_HUMAN	1,90	**3,36**	**4,27**
		5,70	5,61	**13,45**
Phosphatidylinositol transfer protein alpha isoform	Q00169|PIPNA_HUMAN	1,49	**2,65**	**4,07**
Phosphoglucomutase-1	P36871|PGM1_HUMAN	2,43	3,98	3,83
		7,28	6,28	**15,49**
Phosphoglucomutase-2	Q96G03|PGM2_HUMAN	2,04	2,38	3,03
		2,74	2,29	6,97
Phosphoglycerate kinase 1	P00558|PGK1_HUMAN	**1,60**	**2,43**	**2,81**
		**2,25**	**2,57**	**7,13**
Phosphoglycerate mutase 1	P18669|PGAM1_HUMAN	**1,96**	**2,94**	**3,72**
		**3,76**	**3,27**	**9,93**
Phospholipid hydroperoxide glutathione peroxidase, mitochondrial	P36969|GPX4_HUMAN		4,49	5,53
		0,00	0,00	45,11
Phostensin	Q6NYC8|PHTNS_HUMAN	**1,48**	**2,77**	**4,59**
		0,00	0,00	10,24
Plasma protease C1 inhibitor	P05155|IC1_HUMAN			
		2,32	2,41	**5,92**
Plasminogen activator inhibitor 1 RNA-binding protein	Q8NC51|PAIRB_HUMAN	**2,38**	**4,29**	**6,13**
		3,12	2,85	8,83
Plastin-2	P13796|PLSL_HUMAN	**1,63**	**2,71**	**3,31**
		**2,66**	**3,13**	**9,58**
Platelet glycoprotein 4	P16671|CD36_HUMAN	0,85	1,16	**1,49**
Platelet-activating factor acetylhydrolase IB subunit alpha OS=Pan troglodytes	Q5IS43|LIS1_PANTR	**2,37**	**3,08**	**3,10**
		0,00	0,00	0,00
Platelet-activating factor acetylhydrolase	Q13093|PAFA_HUMAN	**2,44**	1,96	1,40
		3,03	**3,33**	5,06
Pleckstrin	P08567|PLEK_HUMAN	2,06	2,29	3,96
		3,34	3,41	**7,47**
Plectin-1	Q15149|PLEC1_HUMAN	**1,74**	**3,11**	**3,96**
		**3,11**	**3,72**	**9,59**
Plexin domain-containing protein 2	Q6UX71|PXDC2_HUMAN	1,19	1,57	1,44
		3,73	3,71	5,91
PML-RARA-regulated adapter molecule 1	Q96QH2|PRAM_HUMAN	1,68	2,81	3,92
		3,98	3,71	**9,21**
Poly(rC)-binding protein 1	Q15365|PCBP1_HUMAN	**2,31**	**4,21**	5,76
		2,14	3,63	**11,15**
Polyadenylate-binding protein 1	P11940|PABP1_HUMAN	**2,06**	**2,97**	**3,39**
		5,03	3,02	8,92
Prefoldin subunit 2	Q9UHV9|PFD2_HUMAN	1,51	4,26	6,42

		0,00	0,00	13,53
Proactivator polypeptide	P07602|SAP_HUMAN	1,05	**1,62**	**2,50**
		**2,37**	**2,74**	**7,33**
Profilin-1	P07737|PROF1_HUMAN	**1,54**	**2,29**	**2,99**
		**2,25**	**2,51**	**6,77**
Profilin-2	P35080|PROF2_HUMAN	4,83	6,05	4,51
		5,84	0,00	8,09
Programmed cell death 6-interacting protein	Q8WUM4|PDC6I_HUMAN	**1,32**	**1,93**	**2,48**
		**4,19**	**3,93**	**9,92**
Programmed cell death protein 5	O14737|PDCD5_HUMAN	3,29	7,96	12,73
		8,41	7,86	**20,84**
Proliferating cell nuclear antigen	P12004|PCNA_HUMAN	2,04	4,05	**4,59**
		4,25	3,89	9,68
Proliferation-associated protein 2G4	Q9UQ80|PA2G4_HUMAN	**1,81**	**3,49**	**3,90**
		3,54	**4,01**	**9,86**
Prolow-density lipoprotein receptor-related protein 1	Q07954|LRP1_HUMAN	1,97	**2,98**	2,38
		2,99	3,58	**7,47**
Proteasome activator complex subunit 1	Q06323|PSME1_HUMAN	2,29	**3,84**	**4,37**
		**2,80**	**2,70**	**9,84**
Proteasome activator complex subunit 2	Q9UL46|PSME2_HUMAN	1,33	**2,92**	**4,84**
		**3,71**	**3,70**	**14,69**
Proteasome activator complex subunit 4	Q14997|PSME4_HUMAN	41,84	57,64	34,41
		194,44	151,93	231,18
Proteasome subunit alpha type-1	P25786|PSA1_HUMAN	**1,73**	**2,17**	**2,52**
		**2,31**	**2,33**	**4,99**
Proteasome subunit alpha type-2	P25787|PSA2_HUMAN	1,97	3,70	3,75
		3,56	3,24	6,39
Proteasome subunit alpha type-3	P25788|PSA3_HUMAN	**1,52**	**2,41**	**2,63**
		**1,55**	**2,07**	**4,14**
Proteasome subunit alpha type-4	P25789|PSA4_HUMAN	1,41	2,20	**2,47**
		2,05	1,85	4,32
Proteasome subunit alpha type-5	P28066|PSA5_HUMAN	2,98	3,72	5,54
		0,00	5,05	9,47
Proteasome subunit alpha type-6	P60900|PSA6_HUMAN	**2,00**	**2,53**	**2,80**
		2,77	**2,53**	**5,03**
Proteasome subunit alpha type-7	O14818|PSA7_HUMAN	**1,39**	**1,90**	**2,31**
		2,95	2,89	6,17
Proteasome subunit beta type-1	P20618|PSB1_HUMAN	1,91	3,06	3,15
		2,11	1,83	**4,31**
Proteasome subunit beta type-10	P40306|PSB10_HUMAN	1,08	1,84	2,17
		1,89	2,19	4,32
Proteasome subunit beta type-2	P49721|PSB2_HUMAN	1,44	**1,93**	**2,57**
		4,16	**3,00**	**6,41**
Proteasome subunit beta type-5	P28074|PSB5_HUMAN	1,36	2,22	1,96
		1,88	1,89	2,96
Proteasome subunit beta type-7	Q99436|PSB7_HUMAN	1,28	1,69	**2,23**
		1,68	1,49	4,45
Proteasome subunit beta type-8	P28062|PSB8_HUMAN	1,28	1,83	2,20
		2,11	**1,93**	**4,41**
Protein AHNAK2	Q8IVF2|AHNK2_HUMAN	**9999,00**	**9999,00**	**9999,00**
		25,50	20,06	22,38
Protein CDV3 homolog	Q9UKY7|CDV3_HUMAN	2,18	**4,13**	**6,09**
		4,30	3,10	**9,72**
Protein disulfide-isomerase A3	P30101|PDIA3_HUMAN	**1,51**	**2,49**	**2,21**
		**2,55**	**2,28**	**3,77**
Protein disulfide-isomerase A4	P13667|PDIA4_HUMAN	**1,79**	**2,45**	**2,04**
		**2,05**	**1,60**	**2,93**
Protein disulfide-isomerase A6	Q15084|PDIA6_HUMAN	**2,32**	**3,71**	**4,36**
		0,00	0,00	0,00
Protein disulfide-isomerase	P07237|PDIA1_HUMAN	**1,50**	**2,31**	**1,75**
		**2,14**	**2,06**	**3,07**
Protein DJ-1	Q99497|PARK7_HUMAN	1,69	**2,90**	**3,75**
		1,98	**2,42**	**7,36**
Protein FAM21B	Q5SNT6|FA21B_HUMAN	1,66	**3,06**	**5,10**
Protein kinase C and casein kinase substrate in neurons protein 2	Q9UNF0|PACN2_HUMAN	1,91	4,93	**6,48**
		7,86	13,28	38,78
Protein LAP2	Q96RT1|LAP2_HUMAN	**2,58**	4,97	6,09
Protein LYRIC	Q86UE4|LYRIC_HUMAN	1,87	**3,58**	**6,77**
		3,31	2,52	8,18
Protein LZIC	Q8WZA0|LZIC_HUMAN	1,94	**4,61**	**8,20**
Protein phosphatase inhibitor 2	P41236|IPP2_HUMAN	1,75	**4,18**	6,48
		4,91	3,48	17,53
Protein S100-A10	P60903|S10AA_HUMAN	1,26	**2,29**	**3,47**
		2,56	2,76	**6,93**
Protein S100-A11	P31949|S10AB_HUMAN	1,55	2,68	**3,86**
		**3,21**	**3,25**	**11,41**
Protein S100-A4	P26447|S10A4_HUMAN	1,40	**3,05**	**4,17**
		**2,64**	**3,11**	**10,56**
Protein S100-A6	P06703|S10A6_HUMAN	2,21	3,38	3,97
		**3,13**	**3,36**	**13,34**
Protein S100-A8	P05109|S10A8_HUMAN	**1,41**	**1,54**	**1,92**
		**2,17**	**2,37**	**4,69**
Protein S100-A9	P06702|S10A9_HUMAN	**1,54**	**1,83**	**2,68**
		**2,60**	**2,50**	**5,59**
Protein SET	Q01105|SET_HUMAN	2,00	3,27	**5,49**
		**3,08**	**2,35**	**7,44**
Protein-L-isoaspartate(D-aspartate) O-methyltransferase	P22061|PIMT_HUMAN	**1,70**	3,48	4,49
		3,14	**3,40**	5,97
Prothymosin alpha	P06454|PTMA_HUMAN	1,27	2,02	**3,12**
		1,61	1,27	2,52
Pterin-4-alpha-carbinolamine dehydratase	P61457|PHS_HUMAN	1,49	**1,87**	3,37
Purine nucleoside phosphorylase	P00491|PNPH_HUMAN	1,73	3,21	2,87
		2,82	3,70	6,91
Puromycin-sensitive aminopeptidase	P55786|PSA_HUMAN	2,07	3,38	3,67
		**2,69**	**2,51**	**6,45**
Putative elongation factor 1-alpha-like 3	Q5VTE0|EF1A3_HUMAN			
		**4,42**	**4,53**	**13,70**
Putative heterogeneous nuclear ribonucleoprotein A1-like protein 3	P0C7M2|RA1L3_HUMAN	1,49	**2,84**	**3,64**
		3,39	3,13	**7,42**
Putative nucleoside diphosphate kinase	O60361|NDK8_HUMAN	**1,78**	**3,67**	**4,72**
Putative phospholipase B-like 2	Q8NHP8|PLBL2_HUMAN	1,96	3,70	6,68
		**4,66**	**3,80**	**12,11**
Putative tropomyosin alpha-3 chain-like protein	A6NL28|TPM3L_HUMAN	**1,87**	4,26	**8,14**
		**2,89**	**3,04**	**8,86**
Pyridoxal kinase	O00764|PDXK_HUMAN	**2,04**	**2,75**	**3,01**
		4,54	3,93	**9,86**
Pyruvate kinase isozymes M1/M2	P14618|KPYM_HUMAN	**2,21**	**4,09**	**5,19**
		**3,06**	**4,23**	**13,04**
Quinone oxidoreductase PIG3	Q53FA7|QORX_HUMAN			
		0,00	0,00	**7,10**
Rab GDP dissociation inhibitor beta	P50395|GDIB_HUMAN	**1,81**	**2,57**	**2,92**
		**3,28**	**3,24**	**8,13**
Ran-specific GTPase-activating protein	P43487|RANG_HUMAN	1,75	4,40	**7,39**
		2,26	5,86	20,30

Ras GTPase-activating protein-binding protein 2	Q9UN86|G3BP2_HUMAN	1,58	**3,12**	**4,30**
Ras GTPase-activating-like protein IQGAP1	P46940|IQGA1_HUMAN	**1,81**	**3,38**	**4,37**
		**3,28**	**3,59**	**9,05**
Ras suppressor protein 1	Q15404|RSU1_HUMAN	1,27	**2,64**	**4,52**
		2,33	1,90	5,03
Ras-related C3 botulinum toxin substrate 1	P63000|RAC1_HUMAN	3,45	6,31	7,78
		3,38	5,48	18,85
Ras-related protein Rab-10	P61026|RAB10_HUMAN	0,00	7,92	11,71
		3,93	3,73	13,51
Ras-related protein Rab-11A	P62491|RB11A_HUMAN	1,95	**3,89**	**5,49**
Ras-related protein Rab-1A	P62820|RAB1A_HUMAN	2,00	3,71	**4,52**
Ras-related protein Rab-1B	Q9H0U4|RAB1B_HUMAN	0,00	0,00	5,40
		12,53	14,36	37,78
Ras-related protein Rap-2b	P61225|RAP2B_HUMAN	1,12	1,84	2,27
		0,00	0,00	27,57
Receptor expression-enhancing protein 5	Q00765|REEP5_HUMAN	2,23	**3,53**	4,76
Reticulon-1	Q16799|RTN1_HUMAN	**1,92**	**4,34**	**6,99**
		4,37	7,93	**23,27**
Retinal dehydrogenase 1	P00352|AL1A1_HUMAN	**1,83**	**2,26**	**2,44**
		0,00	0,00	9,86
Retinal dehydrogenase 2	O94788|AL1A2_HUMAN	**1,81**	**2,34**	**3,19**
		0,00	0,00	0,00
Retinol-binding protein 4	P02753|RET4_HUMAN	0,00	0,00	0,00
		**9999,00**	9999,00	**9999,00**
Rho GDP-dissociation inhibitor 1	P52565|GDIR1_HUMAN	**1,43**	**2,72**	**3,60**
		**2,93**	**3,24**	**10,18**
Rho GDP-dissociation inhibitor 2	P52566|GDIR2_HUMAN	**1,38**	**2,40**	**3,16**
		**2,08**	**2,41**	**6,47**
Rho guanine nucleotide exchange factor 1	Q92888|ARHG1_HUMAN	1,42	2,91	3,65
		1,44	1,49	3,20
Ribonuclease inhibitor	P13489|RINI_HUMAN	2,10	**3,72**	**4,87**
		3,82	4,22	**14,87**
Ribonuclease K6	Q93091|RNAS6_HUMAN	1,03	1,69	2,31
		2,65	2,03	4,25
Ribosome-binding protein 1	Q9P2E9|RRBP1_HUMAN	**1,34**	**2,72**	**3,78**
		2,71	**2,83**	**5,56**
S-adenosylmethionine synthetase isoform type-2	P31153|METK2_HUMAN	1,77	2,92	**3,06**
		0,00	0,00	28,33
Scavenger receptor cysteine-rich type 1 protein M130	Q86VB7|C163A_HUMAN	2,67	4,29	**4,29**
Septin-2	Q15019|SEPT2_HUMAN	**1,80**	**2,93**	**3,88**
Septin-7	Q16181|SEPT7_HUMAN	**2,12**	**3,32**	**3,71**
Septin-9	Q9UHD8|SEPT9_HUMAN	1,82	**3,30**	4,65
		5,48	0,00	7,76
Serglycin	P10124|SRGN_HUMAN			
		1,19	0,89	**1,56**
Serine/threonine-protein kinase 10	O94804|STK10_HUMAN	2,29	3,81	5,40
		3,31	2,64	5,49
Serine/threonine-protein kinase 4	Q13043|STK4_HUMAN	4,15	**5,33**	**6,23**
Serine/threonine-protein kinase PAK 2	Q13177|PAK2_HUMAN	2,71	**6,20**	**9,59**
		0,00	0,00	20,56
Serine/threonine-protein phosphatase 2A 65 kDa regulatory subunit A alpha isoform	P30153|2AAA_HUMAN	**1,97**	**3,51**	**4,50**
		**3,41**	**3,90**	**12,12**
Serine/threonine-protein phosphatase PP1-alpha catalytic subunit	P62136|PP1A_HUMAN	2,50	4,18	4,18
		3,86	4,42	10,68
Serotransferrin	P02787|TRFE_HUMAN	**2,43**	**2,68**	**1,72**
		**1,46**	1,41	**2,25**
Serpin B8	P50452|SPB8_HUMAN	1,67	2,82	**3,28**
Serum albumin	P02768|ALBU_HUMAN	**1,64**	**1,50**	**1,24**
		**2,25**	**1,85**	**6,70**
SH3 domain-binding glutamic acid-rich-like protein 3	Q9H299|SH3L3_HUMAN	1,38	1,38	**2,12**
		**2,93**	**3,01**	**9,37**
SH3 domain-binding glutamic acid-rich-like protein	O75368|SH3L1_HUMAN	1,42	**2,64**	**4,47**
		**2,75**	**3,41**	**10,65**
Sialic acid synthase	Q9NR45|SIAS_HUMAN	**3,56**	**5,47**	**7,03**
Signal recognition particle 14 kDa protein	P37108|SRP14_HUMAN	**1,92**	**5,26**	**5,77**
Signal-regulatory protein beta-1 isoform 3	Q5TFQ8|SIRBL_HUMAN	1,75	3,08	6,22
		2,04	3,59	10,64
Signal-regulatory protein beta-1	O00241|SIRB1_HUMAN	1,23	4,37	8,25
		2,81	5,22	**13,56**
SLAM family member 5	Q9UIB8|SLAF5_HUMAN	**1,16**	2,38	**2,79**
		1,71	2,18	6,73
Small nuclear ribonucleoprotein Sm D2	P62316|SMD2_HUMAN	1,46	1,67	2,87
		3,53	4,52	**6,52**
Small ubiquitin-related modifier 2	P61956|SUMO2_HUMAN	1,94	3,02	4,27
		2,66	2,99	8,73
Sortilin	Q99523|SORT_HUMAN	1,14	**2,20**	**3,05**
		0,00	0,00	0,00
Sorting nexin-12	Q9UMY4|SNX12_HUMAN	0,92	2,45	3,45
		5,12	3,43	13,37
Sorting nexin-2	O60749|SNX2_HUMAN	1,69	**3,63**	**6,32**
SPARC	P09486|SPRC_HUMAN	1,05	1,25	1,41
		**1,78**	**2,57**	**3,97**
Spectrin alpha chain, brain	Q13813|SPTA2_HUMAN	3,49	4,36	**4,18**
		0,00	0,00	10,44
Spectrin beta chain, brain 1	Q01082|SPTB2_HUMAN	5,30	**7,41**	**6,88**
Spliceosome RNA helicase BAT1	Q13838|UAP56_HUMAN	**3,21**	**4,57**	**4,11**
Splicing factor 1	Q15637|SF01_HUMAN	2,73	3,28	**4,85**
		6,71	0,00	6,20
Splicing factor, arginine/serine-rich 1	Q07955|SFRS1_HUMAN	**1,44**	**2,47**	**4,06**
Splicing factor, arginine/serine-rich 6	Q13247|SFRS6_HUMAN	1,23	2,11	**2,34**
Staphylococcal nuclease domain-containing protein 1	Q7KZF4|SND1_HUMAN	**1,79**	**3,69**	**3,65**
		0,00	0,00	0,00
Stathmin	P16949|STMN1_HUMAN	2,43	**4,86**	**6,34**
		**8,03**	**7,64**	**25,27**
Stress-70 protein, mitochondrial	P38646|GRP75_HUMAN	**2,12**	**3,81**	**3,23**
		2,06	2,78	8,39
Stress-induced-phosphoprotein 1	P31948|STIP1_HUMAN	**2,09**	**4,68**	**6,62**
		**4,30**	**4,97**	**15,91**
Sulfhydryl oxidase 1	O00391|QSOX1_HUMAN	1,02	1,11	1,08
		2,25	1,26	2,40
Superoxide dismutase [Cu-Zn]	P00441|SODC_HUMAN	1,50	**2,95**	**4,17**
		1,85	2,27	**7,02**
Superoxide dismutase [Mn], mitochondrial	P04179|SODM_HUMAN	**1,51**	**2,49**	**2,88**

		2,03	**2,41**	**4,78**
Switch-associated protein 70	Q9UH65|SWP70_HUMAN	1,15	2,61	**4,00**
Syndecan-4	P31431|SDC4_HUMAN	1,87	2,01	**1,95**
		0,00	0,00	8,24
Talin-1	Q9Y490|TLN1_HUMAN	**1,90**	**3,41**	**4,11**
		**3,93**	**3,39**	**9,65**
Target of Myb protein 1	O60784|TOM1_HUMAN	2,06	3,62	5,71
		9,38	13,37	32,19
T-complex protein 1 subunit alpha	P17987|TCPA_HUMAN	3,74	4,46	5,25
		5,49	7,26	**19,09**
T-complex protein 1 subunit beta	P78371|TCPB_HUMAN	**2,11**	**3,97**	**4,70**
		3,27	3,07	**10,19**
T-complex protein 1 subunit delta	P50991|TCPD_HUMAN	2,82	**5,09**	**6,15**
T-complex protein 1 subunit epsilon	P48643|TCPE_HUMAN	**1,95**	**3,85**	**4,02**
		6,20	5,65	**14,83**
T-complex protein 1 subunit gamma	P49368|TCPG_HUMAN	**3,33**	**4,91**	**5,21**
		5,53	7,36	**23,95**
T-complex protein 1 subunit theta	P50990|TCPQ_HUMAN	**2,36**	**3,91**	**4,53**
		**5,20**	4,00	**8,77**
T-complex protein 1 subunit zeta	P40227|TCPZ_HUMAN	1,54	**3,18**	**3,25**
		0,00	0,00	6,67
Testin	Q9UGI8|TES_HUMAN	0,00	**5,98**	**4,84**
		0,00	0,00	0,00
Tetratricopeptide repeat protein 28	Q96AY4|TTC28_HUMAN			
		6,38	3,18	**14,62**
Thiopurine S-methyltransferase	P51580|TPMT_HUMAN	2,62	3,11	2,68
		0,00	0,00	25,07
Thioredoxin domain-containing protein 12	O95881|TXD12_HUMAN	1,54	**2,87**	**3,05**
Thioredoxin	P10599|THIO_HUMAN	1,53	**3,15**	**5,46**
		3,19	3,23	**13,13**
Thioredoxin reductase 1, cytoplasmic	Q16881|TRXR1_HUMAN	1,23	**1,61**	**1,85**
		**1,91**	**1,67**	**4,11**
Thioredoxin-dependent peroxide reductase, mitochondrial	P30048|PRDX3_HUMAN	2,87	4,39	5,10
		0,00	9,03	14,34
Thioredoxin-like protein 1	O43396|TXNL1_HUMAN	2,00	2,76	3,19
		4,18	2,91	10,52
THO complex subunit 4	Q86V81|THOC4_HUMAN	1,53	2,86	3,76
		7,63	4,80	9,65
Thymidine phosphorylase	P19971|TYPH_HUMAN	**2,08**	**2,95**	**4,42**
		**2,60**	**3,84**	**11,87**
Thymosin beta-10	P63313|TYB10_HUMAN	2,03	4,01	2,48
		3,35		6,23
Thymosin beta-4-like protein 3	A8MW06|TMSL3_HUMAN			
		**2,59**	**1,96**	**7,85**
Transaldolase	P37837|TALDO_HUMAN	**1,44**	**2,08**	**2,80**
		**2,40**	**2,32**	**5,32**
Transcription elongation factor B polypeptide 1	Q15369|ELOC_HUMAN	3,16	4,39	11,07
		5,22	4,62	**12,01**
Transcription elongation factor B polypeptide 2	Q15370|ELOB_HUMAN	**2,05**	**3,18**	**4,26**
		0,00	0,00	19,00
Transferrin receptor protein 1	P02786|TFR1_HUMAN	**1,51**	**3,23**	**3,64**
		**2,11**	**4,57**	**11,40**
Transforming growth factor-beta-induced protein ig-h3	Q15582|BGH3_HUMAN	1,03	1,21	1,16
		**2,30**	**1,79**	**2,39**
Transgelin-2	P37802|TAGL2_HUMAN	**1,73**	**4,66**	**7,05**
		**3,71**	**5,89**	**17,05**
Transitional endoplasmic reticulum ATPase	P55072|TERA_HUMAN	**2,86**	**4,21**	**5,01**
		**3,86**	**3,49**	**8,99**
Transketolase	P29401|TKT_HUMAN	**1,52**	**2,03**	**2,29**
		**2,26**	**2,05**	**5,30**
Translationally-controlled tumor protein	P13693|TCTP_HUMAN	1,59	**2,43**	**3,70**
		**2,57**	**2,69**	**8,36**
Translin	Q15631|TSN_HUMAN	1,26	**1,91**	**2,88**
		2,69	2,09	**5,93**
Transmembrane glycoprotein NMB	Q14956|GPNMB_HUMAN	0,83	1,49	1,68
		2,02	2,44	7,19
Transmembrane protease, serine 13	Q9BYE2|TMPSD_HUMAN	1,20	1,23	1,31
		1,30	1,50	1,79
Triosephosphate isomerase	P60174|TPIS_HUMAN	**1,59**	**2,31**	**2,93**
		**3,03**	**3,11**	**7,95**
Tripeptidyl-peptidase 1 OS=Pan troglodytes	Q5IS74|TPP1_PANTR	1,90	2,93	2,88
		2,88	2,04	**3,95**
Tropomodulin-3	Q9NYL9|TMOD3_HUMAN	1,68	3,01	**3,84**
		0,00	0,00	21,92
Tropomyosin alpha-3 chain	P06753|TPM3_HUMAN	2,34	4,12	6,56
		4,80	4,06	13,60
Tropomyosin alpha-4 chain	P67936|TPM4_HUMAN	1,60	**3,45**	**5,93**
		**2,90**	**2,87**	**8,77**
Trypsin-3	P35030|TRY3_HUMAN	1,35	1,22	1,29
		1,59	1,64	1,98
Tryptophanyl-tRNA synthetase, cytoplasmic	P23381|SYWC_HUMAN	**1,41**	**2,28**	**2,68**
		**2,17**	**2,22**	**6,24**
Tubulin alpha-1A chain	Q71U36|TBA1A_HUMAN	**2,20**	**3,74**	**4,24**
Tubulin alpha-1C chain	Q9BQE3|TBA1C_HUMAN			
		**4,17**	**3,68**	**10,58**
Tubulin beta chain	P07437|TBB5_HUMAN	2,05	3,68	4,09
		3,29	3,89	11,37
Tubulin beta-2C chain	P68371|TBB2C_HUMAN	2,16	4,51	3,30
		0,00	0,00	12,81
Tubulin-specific chaperone A	O75347|TBCA_HUMAN	**1,81**	**3,85**	**5,73**
		**3,00**	**3,61**	**14,16**
Tumor protein D54	O43399|TPD54_HUMAN	4,08	7,75	**16,41**
		3,03	3,30	15,59
Twinfilin-2	Q6IBS0|TWF2_HUMAN	**1,87**	3,32	**4,43**
		**2,92**	**3,29**	**9,68**
Tyrosine-protein phosphatase non-receptor type 6	P29350|PTN6_HUMAN	**1,97**	**3,33**	**3,86**
		3,14	**3,52**	**10,22**
	P78324|SHPS1_HUMAN	**2,50**	**6,13**	**8,31**
Tyrosine-protein phosphatase non-receptor type substrate 1		6,41	5,39	15,56
Tyrosyl-tRNA synthetase, cytoplasmic	P54577|SYYC_HUMAN	2,87	3,92	6,17
		1,86	2,60	11,10
U1 small nuclear ribonucleoprotein A	P09012|SNRPA_HUMAN	2,07	3,62	5,07
		**8,90**	5,49	11,93
Ubiquitin carboxyl-terminal hydrolase isozyme L1	P09936|UCHL1_HUMAN	0,96	1,90	2,22
		0,00	0,00	8,23
Ubiquitin carboxyl-terminal hydrolase isozyme L3	P15374|UCHL3_HUMAN	**3,68**	**5,39**	**6,06**
Ubiquitin	P62988|UBIQ_HUMAN	1,93	**3,23**	**3,79**
		**2,94**	**2,55**	**7,81**
Ubiquitin-associated protein 2-like	Q14157|UBP2L_HUMAN	3,30	6,74	8,87
		8,36	6,66	22,41
Ubiquitin-conjugating enzyme E2 L3	P68036|UB2L3_HUMAN	2,53	**4,71**	**5,46**
		**5,29**	**4,55**	**13,82**
Ubiquitin-conjugating enzyme E2 N	P61088|UBE2N_HUMAN	2,70	**4,95**	**6,05**

Ubiquitin-like modifier-activating enzyme 1	P22314|UBA1_HUMAN	**1,97**	**3,41**	**3,79**
		3,08	**2,49**	**9,99**
UBX domain-containing protein 1	Q04323|UBXN1_HUMAN	2,71	4,40	**5,99**
		0,00	0,00	4,52
UMP-CMP kinase	P30085|KCY_HUMAN	**1,48**	**3,39**	**3,98**
		6,24	0,00	14,35
UPF0485 protein C1orf144	Q7Z422|CA144_HUMAN	4,38	5,17	**4,85**
UTP--glucose-1-phosphate uridylyltransferase	Q16851|UGPA_HUMAN	**1,79**	**3,59**	**4,35**
		4,33	3,11	11,42
UV excision repair protein RAD23 homolog B	P54727|RD23B_HUMAN	1,52	**2,64**	**4,25**
		**2,90**	3,20	**8,17**
Vacuolar protein sorting-associated protein 29	Q9UBQ0|VPS29_HUMAN	2,73	**3,96**	**3,94**
		2,76	2,84	5,49
WAS/WASL-interacting protein family member 1	O43516|WIPF1_HUMAN	**2,54**	6,44	7,27
Vasodilator-stimulated phosphoprotein	P50552|VASP_HUMAN	1,56	**2,39**	**3,17**
		6,28	10,31	**14,65**
WD repeat-containing protein 1	O75083|WDR1_HUMAN	**1,88**	**2,54**	**3,10**
		**2,14**	**2,29**	**6,63**
Vesicle-associated membrane protein-associated protein A	Q9P0L0|VAPA_HUMAN	2,15	4,53	7,48
		8,01	5,19	14,85
Vimentin	P08670|VIME_HUMAN	**1,47**	**4,05**	**8,17**
		**4,52**	**4,42**	**14,43**
Vinculin	P18206|VINC_HUMAN	**1,61**	**2,59**	**2,84**
		**3,56**	**3,08**	**6,77**
V-type proton ATPase catalytic subunit A	P38606|VATA_HUMAN	**2,04**	**2,91**	**2,82**
		**6,52**	**5,83**	**12,72**
V-type proton ATPase subunit B, brain isoform	P21281|VATB2_HUMAN	1,94	4,04	3,51
		0,00	0,00	20,33
V-type proton ATPase subunit C 1	P21283|VATC1_HUMAN	**2,12**	**4,01**	**4,44**
		8,18	6,80	10,08
V-type proton ATPase subunit D	Q9Y5K8|VATD_HUMAN	2,03	2,28	**4,81**
V-type proton ATPase subunit E 1	P36543|VATE1_HUMAN	1,47	**4,11**	**5,80**
		**4,14**	**4,52**	11,33
V-type proton ATPase subunit G 1	O75348|VATG1_HUMAN	1,88	4,59	4,96
		2,22	2,81	10,22
Xaa-Pro dipeptidase	P12955|PEPD_HUMAN	**1,69**	2,11	**2,58**
		**2,33**	**1,64**	**5,45**
Zyxin	Q15942|ZYX_HUMAN	**1,93**	**3,77**	**5,84**
		4,04	3,55	**12,00**

bold *= statistically significant quantitation result (p<0,05)*
0,000 = protein identified but no quantitation result
